# New Hygrocins K–U and Streptophenylpropanamide A and Bioactive Compounds from the Marine-Associated *Streptomyces* sp. ZZ1956

**DOI:** 10.3390/antibiotics11111455

**Published:** 2022-10-22

**Authors:** Wenwen Yi, Asif Wares Newaz, Kuo Yong, Mingzhu Ma, Xiao-Yuan Lian, Zhizhen Zhang

**Affiliations:** 1 Ocean College, Zhoushan Campus, Zhejiang University, Zhoushan 316021, China; 2 Zhejiang Marine Development Research Institute, Zhoushan 316000, China; 3 College of Pharmaceutical Sciences, Zhejiang University, Hangzhou 310058, China

**Keywords:** marine *Streptomyces* sp. ZZ1956, Streptomycetaceae, hygrocins K–U, streptophenylpropanamide A, structure elucidation, antiglioma activity, antibacterial activity

## Abstract

Marine-derived *Streptomyces* actinomycetes are one of the most important sources for the discovery of novel bioactive natural products. This study characterized the isolation, structural elucidation and biological activity evaluation of thirty compounds, including twelve previously undescribed compounds, namely hygrocins K–U (**5**–**13**, **17** and **18**) and streptophenylpropanamide A (**23**), from the marine-associated actinomycete *Streptomyces* sp. ZZ1956. Structures of the isolated compounds were determined by a combination of extensive NMR spectroscopic analyses, HRESIMS data, the Mosher’s method, ECD calculations, single crystal X-ray diffraction and comparison with reported data. Hygrocins C (**1**), D (**2**), F (**4**), N (**8**), Q (**11**) and R (**12**), 2-acetamide-6-hydroxy-7-methyl-1,4-naphthoquinone (**22**), echoside C (**27**), echoside A (**28**) and 11,11′-*O*-dimethylelaiophylin (**30**) had antiproliferative activity (IC_50_: 0.16–19.39 μM) against both human glioma U87MG and U251 cells with hygrocin C as the strongest active compound (IC_50_: 0.16 and 0.35 μM, respectively). The analysis of the structure–activity relationship indicated that a small change in the structures of the naphthalenic ansamycins had significant influence on their antiglioma activities. Hygrocins N (**8**), O (**9**), R (**12**), T (**17**) and U (**18**), 2-amino-6-hydroxy-7-methyl-1,4-naphthoquinone (**21**), 2-acetamide-6-hydroxy-7-methyl-1,4-naphthoquinone (**22**), 3′-methoxy(1,1′,4′,1″-terphenyl)-2′,6′-diol (**26**), echoside C (**27**) and echoside A (**28**) showed antibacterial activity against methicillin-resistant *Staphylococcus aureus* and *Escherichia coli* with MIC values of 3–48 μg/mL.

## 1. Introduction

Compound 3-amino-5-hydroxy benzoic acid (3,5-AHBA) is the precursor of a big group of natural products, including the ansamycins, the mitomycins and the unique saliniketals (degraded ansamycins) [1]. The ansamycins have two characteristic structural features: an aromatic core and a so-called ansa bridge containing a lactam moiety, whose two ends link to two nonadjacent positions of the aromatic core [2]. The ansamycins can be divided into naphthalenic or benzenic depending on the nature of their aromatic ring. The naphthalenic ansamycins include rifamycins, ansalactams, chaxamycins, divergolides, hygrocins, naphthomycins, rubradirins and streptovaricins. Their structural characteristic is a 1,4-naphtoquinone or a 1,4-hydroxynaphtalene chromophore. Benzenic ansamycins have a 1,4-benzoquinone or a 1,4-hydrobenzoquinone chromophore and include ansatrienins, cebulactams, cytotrienins, geldanamycins, herbimycins, macbecins, maytansines (ansamitocins) and tetrapetalones. Precursor feeding experiments and genetic and biochemical methods have been applied to investigate the biosynthesis of the ansamycins, demonstrating that AHBA is the source of the chromophore and the aliphatic ansa chains are derived from acetate, propionate, isobutyrate or glycolate units [1,2].

While most of the ansamycins were isolated from actinomycetes, the class of maytansines was also found in higher plants and mosses [3,4]. However, the first maytansines found in plants [4] are now known to be produced by the interplay amongst bacteria in the root system [5]. It was reported that nearly 300 ansamycins have been identified from natural sources [6] and more and more ansamycins continue to be reported, such as the recently described olimycins from the *ovmO*-inactivated mutant strain *Streptomyces olivaceus* SCSIO T05 [7] and ansaseomycins from a heterologous mutant strain of *Streptomyces seoulensis* [8]. The ansamycins exhibit diverse biological activities, such as antibacterial (naphthomycin A, rifamycin, rubradirin and streptovaricin A), anticancer (ansamitocins P-3, ansatrienins A, geldanamycin, mitomycin C, saliniketals A and B), lipoxygenase inhibitory (tetrapetalones A and B) and antiviral (divergolide O) activities [1,9]. The well-known drugs of the ansamycin family were the first-line anti-tuberculous drug rifamycin and the antibody–drug conjugate Kadcyla (emtansine).

**Figure 1:**
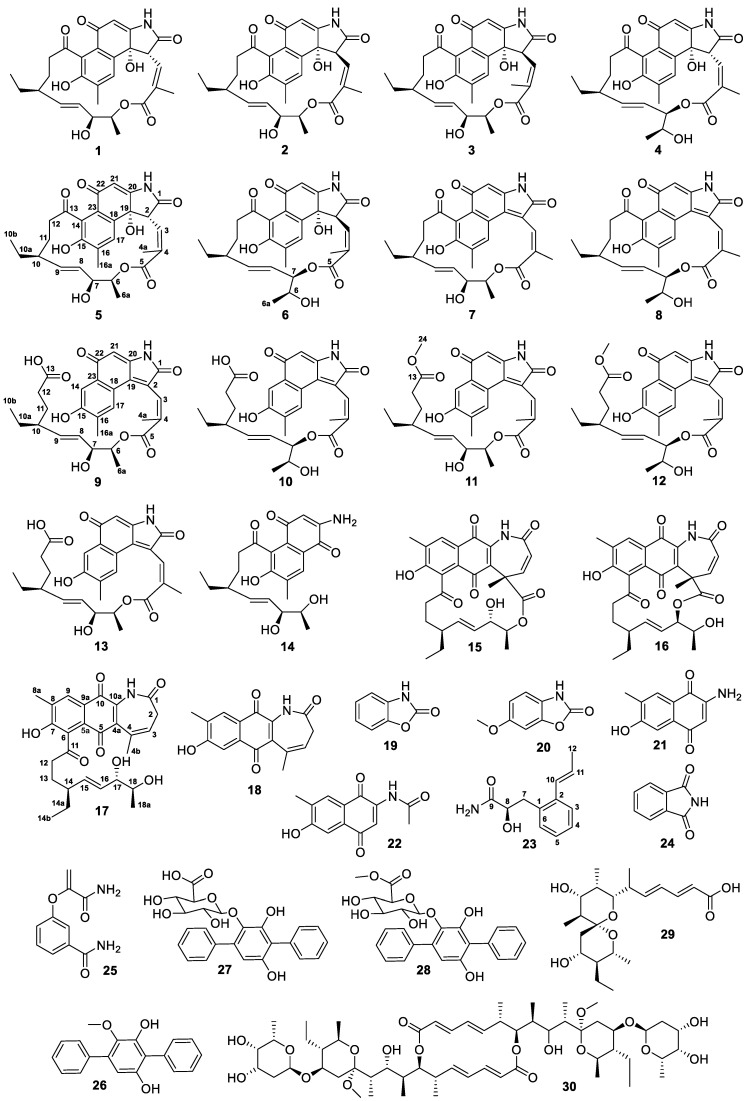
Structure of isolated compounds **1**–**30**.

As part of an ongoing project to discover novel antiglioma natural products from marine microorganisms [10,11,12,13,14,15,16,17,18,19,20], we isolated an actinomycete from a sediment sample collected from an intertidal mangrove area at the Pacific Ocean close to South Sulawesi, Indonesia. This actinomycete was assigned as *Streptomyces* sp. ZZ1956 based on its 16S rDNA sequence analysis (Figure S1 and Table S1). An extract prepared from the culture of the strain ZZ1956 in GYM liquid medium exhibited inhibitory activity against the proliferation of glioma U251 and U87MG cells with inhibition rates of over 90%. Chemical investigation of this active extract resulted in the isolation and identification of thirty compounds **1**–**30**, including eleven new naphthalenic ansamycin analogues hygrocins K–U (**5**–**13**, **17**, **18**) and one new phenylpropanamide derivative streptophenylpropanamide A (**23**) (Figure 1). Herein, we described the isolation and culture of the strain ZZ1956 as well as the isolation, structure elucidation and bioactive evaluation of these isolated compounds.

## 2. Results and Discussion

### 2.1. Structure Elucidation of the Isolated Compounds

After analyses of the NMR spectroscopic data and comparison with related data of references, eighteen known compounds were elucidated to be hygrocins C–F (**1**–**4**) [21], degrahygrocin A (**14**) [22], hygrocin B (**15**) [22], hygrocin G (**16**) [21], benzoxazolone (**19**) [23], coixol (**20**) [24], 2-amino-6-hydroxy-7-methyl-1,4-naphthoquinone (**21**) [25], 2-acetamide-6-hydroxy-7-methyl-1,4-naphthoquinone (**22**) [26], 1H-isoindole-1,3(2H)-dione (**24**) [27], 3-(3′-amino-3′-oxoprop-1′-en-2′-yl)oxy benzamide (**25**) [28], 3′-methoxy(1,1′,4′,1″-terphenyl)-2′,6′-diol (**26**) [29], echoside C (**27**) [30], echoside A (**28**) [30], pteridic acid hydrate (**29**) [31] and 11,11′-O-dimethylelaiophylin (**30**) [32]. The structure of hygrocin C (**1**) was confirmed by single crystal X-ray diffraction (Table S2). Degrahygrocin A (**14**) was previously reported as an alkaline hydrolytic product of hygrocin A [22]; while 2-acetamide-6-hydroxy-7-methyl-1,4-naphthoquinone (**22**) was an intermediate compound of chemical synthesis of ansalactam A [26]. Therefore, both compounds **14** and **22** are reported as natural products for the first time. The NMR data of these known compounds are presented in Tables S3–S14.

**Figure 2:**
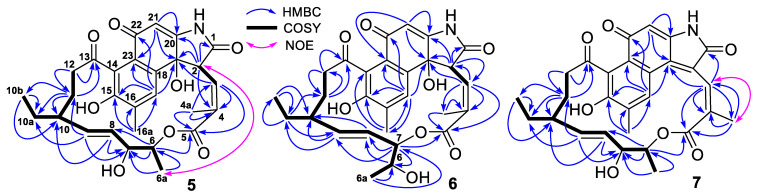
COSY, key HMBC and NOE correlations of hygrocins K–M (**5**–**7**).

Compound ***5*** had the same molecular formula C_28_H_31_NO_8_ and very similar UV characteristic absorptions as hygrocins C–F (**1**–**4**), indicating that they are isomers. Careful analyses of the ^1^H, ^13^C, HMQC, COSY, HMBC and NOESY NMR spectra of **5** demonstrated that its structure was different from those of **1**–**4** in the configurations at C-2 and C_3_-C_4_ double bond as well as the position of the lactone ring formation. The configuration at C-2 was established as *R* based on a strong NOE correlation observed between H-2 and H_3_-6a (Figure 2). A strong NOE correlation of H-2 with H_3_-6a was an indication of 2*R*-configuration in **1** and **4**, compared to the 2*S*-configuration in **2** and **3** without the NOE correlation of H-2 and H_3_-6a [21]. The chemical shift value *(δ*_C_ 13.7) of C-4a in **5** indicated a 3*E*-configuration, compared to the downfield chemical shift values (*δ*_C_ 21.1–22.2) (Tables S3 and S4) of C-4a for a 3*Z*-configuration in **1**, **2** and **4** [21]. Observed strong NOE (for **7**, **8**, **13**), no NOE (for **10**–**12**) or weak NOE (**5**, **6**, **9**) correlation between H-3 and H_3_-4a also supported the assignment of the 3*E*- or 3*Z*-configuration. In addition, the *trans*-coupling constant value of 16.0 Hz (^3^*J*_H8/H9_) indicated an 8*E*-configuration and the small vicinal coupling constant value of 2.8 Hz (^3^*J*_H6/H7_) suggested a *syn* orientation between H-6 and H-7 [21]. HMBC correlations (Figure 2) of H-6 (*δ*_H_ 4.65) with C-5 (*δ*_C_ 167.7) established the linkage of C_5_ and C_6_ through an oxygen to form the lactone ring. It is known that hygrocins C–E (**1**–**3**) have the same 6*S*, 7*S*, 10*S*, 19*R*-configuration. Therefore, we proposed compound **5** to have the same 6*S*, 7*S*, 10*S*, 19*R*-configuration as **1**–**3** based on a common biosynthetic origin. Based on the above analyses, it can be concluded that the structure of **5** is similar to that of **1** with the only difference being the configuration of the C_3_–C_4_ double bond. Therefore, the structure of **5** was elucidated as a previously undescribed member of the naphthoquinone ansamycins, named hygrocin K. Its ^13^C and ^1^H NMR data are reported in Table 1 and Table 2.

Compound ***6*** had the same molecular formula C_28_H_31_NO_8_ as **1**–**5** deduced from its HRESIMS ion peak at *m*/*z* 508.1979 [M–H]^−^ and ^13^C NMR data. Careful analyses of its 1D- and 2D-NMR spectra determined that **6** and hygrocins F (**4**) had the same planar structure. As described above for **5**, no NOE correlation between H-2 with H_3_-6a and weak NOE correlation between H-3 with H_3_-4a as well as the relative upfield shift value at δ_C_ 13.6 for C-4a indicated that **6** had 2S- and 3*E*-configurations [21]. The structure of **6** was thus identified as a previously undescribed naphthoquinone ansamycin, named hygrocin L. Its ^13^C and ^1^H NMR data (Table 1 and Table 2) were assigned based on the HMQC, COSY and HMBC correlations (Figure 2).

**Figure 3:**
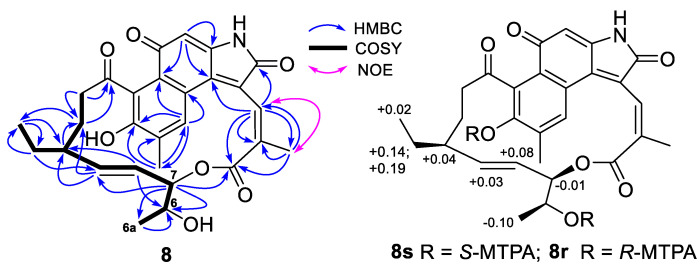
COSY and key HMBC correlations of hygrocin N (**8**) and the Δ*δ_S_*_-*R*_ values for the MTPA esters (**8s** and **8r**) of hygrocin N (**8**).

Compounds **7** and **8** had very similar UV absorptions and the same molecular formula C_28_H_29_NO_7_ deduced from their HRESIMS ion peaks at *m*/*z* 490.1875 and 490.1871 [M–H]^−^, respectively, 18 mass units lower than that of **1**–**6**, corresponding to the loss of a H_2_O molecule. Detailed analyses of the 1D- and 2D-NMR spectra of **7** and **8** as well as comparison of their NMR data with those of **1**–**6** demonstrated that the methine at C-2 and the non-protonated oxygenated carbon at C-19 in **1**–**6** were replaced by two non-protonated olefinic carbons at C-2 (*δ*_C_ 121.9 in **7** or *δ*_C_ 122.6 in **8**) and C-19 (*δ*_C_ 134.8 in **7** or *δ*_C_ 136.4 in **8**). Therefore, both **7** and **8** had a C_2_-C_19_ double bond. The downfield shift values of C-4a at *δ*_C_ 21.1 in **7** or *δ*_C_ 22.1 in **8** and the strong NOE correlation between H-3 and H_3_-4a (Figure 2 and Figure 3) indicated that they had a 3*Z*-configuration; while the *trans*-coupling constant values of 15.1 Hz in **7** and 15.6 Hz in **8** between H-8 and H-9 suggested that both **7** and **8** had an 8*E*-configuration. HMBC correlations of H-6 (*δ*_H_ 4.87) with C-5 (*δ*_C_ 167.6) in **7** and H-7 (*δ*_H_ 4.97) with C-5 (*δ*_C_ 168.3) in **8** established the position of the lactone ring formation. The Mosher’s method was used to determine the configuration at C-6 in **8**. The results (Figure 3 and Table S15) indicated a 6*S*-configuration for **8**. Therefore, compound **7** should have the same 6*S*, 7*S*-configuration as compounds **1**–**3** and **5** and compound **8** should have the same 6*S*, 7*R*-configuration as compounds **4**, **6** and **16** [21] based on their shared biogenesis, the structures of the reported compounds and the Mosher’s method results of **8**. The structures of **7** and **8** were thus elucidated as two previously unreported naphthoquinone ansamycins, named hygrocin M (**7**) and hygrocin N (**8**). The ^13^C and ^1^H NMR data (Table 1 and Table 2) of **7** and **8** were assigned based on the HMQC, COSY and HMBC correlations (Figure 2 and Figure 3).

**Figure 4:**
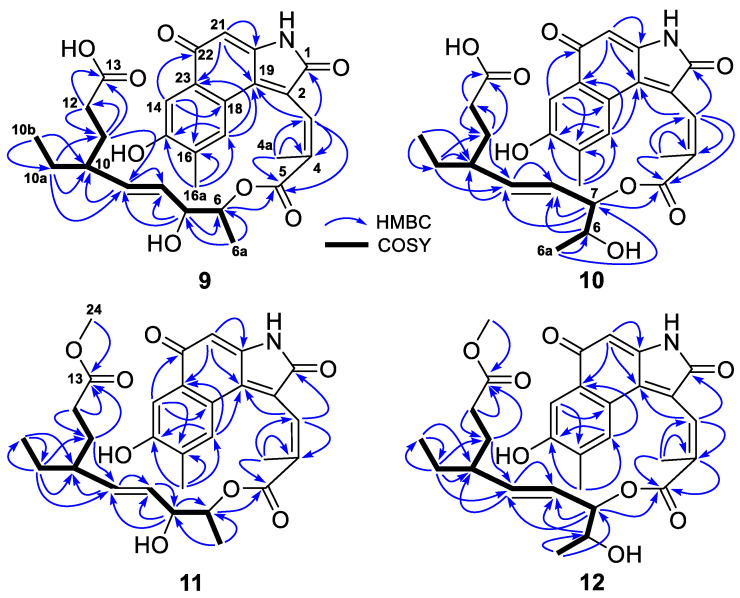
COSY and key HMBC correlations of hygrocins O–R (**9**–**12**).

Compounds **9** and **10** were obtained as a red amorphous powder and had very similar UV characteristic absorptions (around 201 and 335 nm) to those of **7** and **8**, suggesting that they were analogues. Both **9** and **10** had the same molecular formula C_28_H_31_NO_8_ deduced from their ^13^C NMR data and HRESIMS ion peaks at *m*/*z* 508.1975 [M–H]^−^ in **9** and 508.1974 [M–H]^−^ in **10**, 18 mass units higher than those of **7** and **8**. Compared to **7** and **8**, one additional aromatic hydrogen signal at *δ*_H_ 7.43 (s) in **9** or *δ*_H_ 7.42 (s) in **10** was observed in their ^1^H NMR spectra. However, the ^13^C NMR signal at *δ*_C_ 212.9 in **7** or *δ*_H_ 212.0 in **8** for the ketone group at C-13 was replaced in both **9** and **10** by upfield shifted signals at *δ*_C_ 177.7 in **9** or *δ*_C_ 177.8 in **10**. Further analyses of their HMQC, COSY and HMBC correlations (Figure 4) as well as consideration of their molecular formula and 14 degrees of unsaturation required by the molecular formula demonstrated that **9** and **10** were derivatives of **7** and **8**, respectively, with ring opening between C-13 and C-14. The chemical shift at *δ*_C_ 16.4 for C-4a and no NOE or weak NOE correlation between H-3 and H_3_-4a in **9** or **10** indicated a 3*E*-configuration, compared to the downfield shift values at *δ*_C_ 21.1 and 22.1 (Table 1) for the C-4a and the strong NOE correlation between H-3 and H_3_-4a in **7** and **8** with a 3*Z*-configuration. Therefore, the structures of **9** and **10** were elucidated as two previously reported naphthoquinone ansamycins, named hygrocin O (**9**) and hygrocin P (**10**). Their ^13^C and ^1^H NMR data are reported in Table 1 and Table 3.

Compounds **11** and **12** were also obtained as a red amorphous powder and had same molecular formula C_29_H_33_NO_8_ deduced from their ^13^C NMR data and HRESIMS ion peaks at *m*/*z* 522.2125 [M–H]^−^ in **11** and 522.2132 [M–H]^−^ in **12**, 14 mass units higher than those of **9** and **10**. Compared to the ^13^C and ^1^H NMR data of **9** and **10**, both **11** and **12** had additional NMR signals for a methoxy group at *δ*_C_ 52.1 and *δ*_H_ 3.57 (3H, s) in **11** and *δ*_C_ 52.2 and *δ*_H_ 3.65 (3H, s) in **12**. A HMBC correlation of H_3_-24 (*δ*_H_ 3.57) with C-13 (*δ*_C_ 176.0) in **11** and H_3_-24 (*δ*_H_ 3.65) with C-13 (*δ*_C_ 176.0) in **12** established the position of the methoxy group. Further analyses of their HMQC, COSY, HMBC and NOE correlations (Figure 4) demonstrated that **11** and **12** were the methyl esters of **9** and **10**, respectively. The structures of **11** and **12** were thus identified as two previously undescribed naphthoquinone ansamycins, named hygrocin Q (**11**) and R (**12**). The ^13^C and ^1^H NMR data of **11** and **12** are reported in Table 1 and Table 3. It should be noted that **11** and **12** may be the artificial products of methyl esterification of **9** and **10**, respectively, originated in the extraction and separation process.

**Figure 5:**
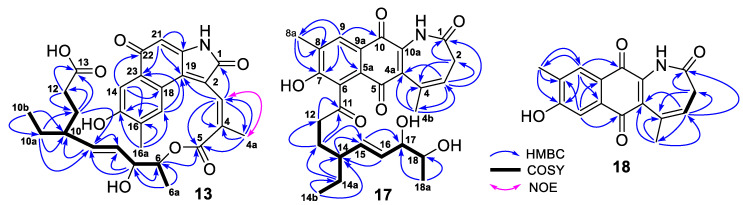
COSY and key HMBC correlations of hygrocins S–U (**13**, **17**, **18**).

The HRESIMS spectrum of compound **13** gave an ion peak at *m*/*z* 508.1975 [M–H]^−^, corresponding to a molecular formula C_28_H_31_NO_8_, which was the same as those of **9** and **10**. Detailed analyses of the 1D- and 2D-NMR spectra of **13** determined that **9** and **13** had the same planar structure and their structural difference was only the different configuration of the C_3_-C_4_ double bond. The downfield shift value at *δ*_C_ 21.1 for C-4a and a strong NOE correlation between H-3 and H_3_-4a (Figure 5) suggested a 3*Z*-configuration in **13**. The structure of **13** was thus assigned as a previously undescribed naphthoquinone ansamycin, named hygrocin S (**13**). Its ^13^C and ^1^H NMR data (Table 1 and Table 3) were assigned based on the HMQC, COSY and HMBC correlations (Figure 5).

Compound **17** was obtained as a yellow amorphous powder and its molecular formula C_27_H_31_NO_7_ was determined based on the HRESIMS ion peak at *m*/*z* 480.2024 [M–H]^−^ and ^13^C NMR data. Interpretation of the ^1^H, ^13^C and HMQC NMR spectra of **17** indicated that its twenty-seven carbons (Table 4) were assigned to four carbonyls, six pairs of double bonds, two oxymethines, one methine, four methylenes and four methyls. These carbon types of **17** were very similar to those of hygrocin B (**15**). Compared to **15**, the NMR spectra of **17** showed additional signals for one non-protonated olefinic carbon and one methylene group at *δ*_C_ 37.7 and *δ*_H_ 2.90 (2H, d, *J* = 7.2 Hz) (Table 4) and lacked the signals for one carbonyl carbon, one protonated olefinic carbon and the non-protonated carbon at *δ*_C_ 52.6 (C-4) (Table S7), which were observed in the NMR spectra of **15**. Further analyses of the HMQC, COSY and HMBC correlations (Figure 5) of **17** indicated that **17** had a C_3_-C_4_ double bond, but did not have the lactone structure existed in **15**. Therefore, compound **17** was a seco-derivative of **15**. A shared biogenesis suggested that **17** and **15** should have the same 14*S*, 17*S*, 18*S*-configuration. Analyses described above resulted in the identification of **17** as a previously undescribed naphthoquinone ansamycin, named hygrocin T. Its ^13^C and ^1^H NMR data (Table 4) were assigned based on the HMQC, COSY and HMBC correlations (Figure 5).

Compound **18** was obtained as a yellow amorphous powder and its HRESIMS gave an ion peak at *m*/*z* [M–H]^−^ 282.0770, corresponding to a molecular formula C_16_H_13_NO_4_ with eleven degrees of unsaturation. Based on the analyses of its ^1^H, ^13^C, DEPT and HMQC NMR spectra, the sixteen carbons were assigned to three carbonyls (*δ*_C_ 184.2, 177.4, 169.3), five pairs of double bonds, one methylene (*δ*_C_ 36.4) and two methyl groups (*δ*_C_ 20.8, 16.3) (Table 5). The three carbonyl and five pairs of double bonds accounted for eight out of the eleven degrees of unsaturation required by the molecular formula, suggesting that **18** had a structure with three rings. Above evidence, together with further analyses of its COSY and HMBC correlations (Figure 5), demonstrated that the core structure of **18** was similar to that of **17** with the only difference being the absence in **18** of the side chain attached. Therefore, the structure of **18** was elucidated as a previously unreported naphthoquinone ansamycin, named hygrocin U. The ^13^C and ^1^H NMR data (Table 5) assignment of **18** was made based on the HMQC, COSY and HMBC correlations (Figure 5).

**Figure 6:**
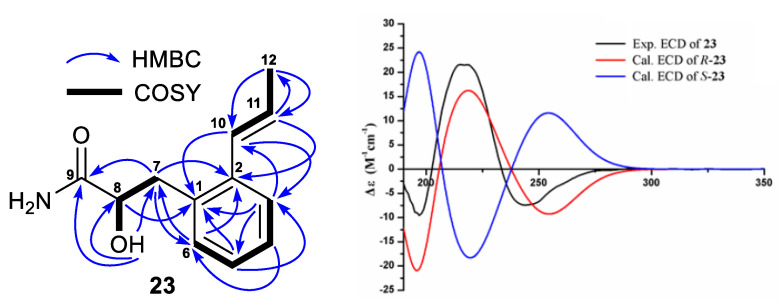
COSY and key HMBC correlations of streptobenzenepropanamide A (**23**) and the experimental ECD spectrum of streptobenzenepropanamide A (**23**) and the calculated ECD curves of the model molecules of *R*-**23** and *S*-**23** at the b3lyp/6-311+g (d, p) level.

The molecular formula C_12_H_15_NO_2_ of **23** was determined by its HRESIMS ion peaks at *m*/*z* 206.1175 [M+H]^+^ and 228.0995 [M+Na]^+^ as well as ^13^C NMR data. The twelve carbons in **23** were assigned to one carbonyl (*δ*_C_ 176.0), eight olefinic carbons, one oxymethine (*δ*_C_ 71.9), one methylene (*δ*_C_ 37.5) and one methyl group (*δ*_C_ 18.7). COSY correlations (Figure 6) of H-11 (*δ*_H_ 6.09, 1H, m) with H-10 (*δ*_H_ 6.74, 1H, dd, 15.8, 1.8 Hz) and H_3_-12 (*δ*_H_ 1.86, 3H, dd, 6.5, 1.8 Hz) as well as HMBC correlations (Figure 6) of H-12 with C-10 (*δ*_C_ 128.6) and C-11 (*δ*_C_ 126.7) indicated the existence of a 1-propen-1-yl group. Similarly, a “-CH_2_-CH(OH)-CO-” structural fragment was established based on the COSY correlations of H-8 (*δ*_H_ 3.92, 1H, ddd, 9.3, 6.2, 3.6 Hz) with H-7 (*δ*_H_ 3.08, 1H, dd, 14.1, 3.6 Hz; 2.64, 1H, dd, 14.1, 9.3 Hz) and OH-8 (*δ*_H_ 5.42, 1H, d, 6.2 Hz) as well as HMBC correlations of H-7 with C-9 (*δ*_C_ 176.0) and OH-8 with C-7 (*δ*_C_ 37.5), C-8 (*δ*_C_ 71.9) and C-9. In the downfield area (*δ*_H_ 6.09–7.60) of the ^1^H NMR spectrum of **23**, there were signals for six olefinic protons. The 1-propen-1-yl group accounted for two olefinic protons and two olefinic carbons and the remaining four olefinic protons and six olefinic carbons were assigned to an aromatic ring. HMBC correlations of H-10 with C-1 (*δ*_C_ 135.6), C-2 (*δ*_C_ 136.6) and C-3 (*δ*_C_ 125.3) and H-3 (*δ*_H_ 7.41, 1H, d, 7.5 Hz) with C-10 established the linkage of the 1-propen-1-yl at C-2. In the same way, the positioning of the “-CH_2_-CH(OH)-CO-” group at C-1 was indicated by HMBC correlations of H-6 (*δ*_H_ 7.15, 1H, m) with C-7, H-7 with C-2 and C-6 (*δ*_C_ 130.7) and H-8 with C-1. In addition, the ^1^H NMR spectrum of **23** showed two noncarbonated proton signals at *δ*_H_ 7.25 (1H, s) and 7.16 (1H, s), which were assigned to NH_2_-9. The HRESIMS data also supported a -NH_2_ group at C-9, rather than a -OH group. A *trans*-coupling constant value of 15.8 Hz (^3^*J*_H10/H11_) indicated a 10*E*-configuration, while the absolute configuration at C-8 was determined based on the results (Figure 6 and Tables S16–S19) from ECD calculations. The ECD spectrum of **23** displayed positive and negative Cotton effects at 215 and 244 nm, respectively, which closely matched those of the ECD curve calculated for 8*R*-**23**. Based on the foregoing evidence, the structure of **23** was identified as a previously undescribed phenylpropanamide analogue, named streptophenylpropanamide A. Its ^13^C and ^1^H NMR data are reported in Table 5.

### 2.2. Biological Activity Evaluation

Sulforhodamine B (SRB) assay was applied to determine the activity of all thirty isolated compounds (**1**–**30**) against the proliferation of glioma cells. Doxorubicin was used as a positive control. The results (Table 6) indicated that compounds **1**, **2**, **4**, **8**, **11**, **12**, **22** and **30** showed potent antiproliferative activity against both glioma U87MG and U251 cells with IC_50_ values ranging from 0.16 to 10.46 μM. Compounds **27** and **28** also had activity in inhibiting the proliferation of glioma U87MG and U251 cells with IC_50_ values of 11.18 and 19.39 μM, respectively. Among all the active compounds, hygrocin C (**1**) showed the strongest activity (IC_50_: 0.16 and 0.35 μM), followed by hygrocin D (**2**) (IC_50_: 0.39 and 2.63 μM). Compounds **1**–**18** were eighteen naphthoquinone ansamycins. It was noted that the active ring closed compounds hygrocins C (**1**), D (**2**) and F (**4**) had a 3*Z*-configuration, compared to the inactive ring closed compounds hygrocins E (**3**), K (**5**) and L (**6**) with a 3*E*-configuration. However, although both hygrocins M (**7**) and N (**8**) had the 3*Z*-configuration, they exhibited significantly different activities due to the different positioning of the ring closure at the C-6 or C-7 position. In addition, most of the ring open compounds hygrocins O (**9**), P (**10**), S (**13**) and T (**17**) and degrahygrocin A (**14**) were inactive. However, the ring open compounds hygrocins Q (**11**) and R (**12**), the methyl esters of hygrocins O (**9**) and P (**10**), respectively, were active. These analyses of the structure–activity relationship indicated that a small change in the structure of this class of compounds had significant influence on their antiglioma activities.

The activity of compounds **1**–**30** in inhibiting the growth of methicillin-resistant *Staphylococcus aureus* (MRSA) and *Escherichia coli* was also evaluated. The results (Table 6) showed that compounds **8**, **9**, **12**, **17**, **18**, **21**, **22** and **26**–**28** exhibited antibacterial activity against both MRSA and *E. coli* with MIC values of 3–48 μg/mL.

## 3. Experimental Section

### 3.1. General Procedures

Optical rotation (OR), ultraviolet–visible (UV), electronic circular dichroism (ECD) and infrared (IR) spectra were recorded on an Autopol I polarimeter (Rudolph Research Analytical, Hackettstown, NJ, USA), a METASH UV-8000 spectrometer (Shanghai METASH Instruments Co. Ltd., Shanghai, China), a JASCO J-815 spectropolarimeter (JASCO Co., Tokyo, Japan) and a Nicolet^TM^ IS^TM^ 10 FT-IR spectrometer (Thermo Fisher Scientific, Waltham, MA, USA), respectively. HRESIMS data were acquired on an Agilent 6230 TOF LC/MS spectrometer (Agilent Technologies Co. Ltd., Santa Clara, CA, USA). NMR spectra were obtained on a JEOL 600 spectrometer (JEOL Co. Ltd., Tokyo, Japan) using standard programs and acquisition parameters and chemical shift values were expressed in *δ* (ppm) relative to DMSO-d_6_ (*δ*_C_ 39.5, *δ*_H_ 2.50), MeOH-d_4_ (*δ*_C_ 49.15, *δ*_H_ 3.31) or acetone-*d*_6_ (*δ*_C_ 29.8, *δ*_H_ 2.05). Diaion HP-20 (Mitsubishi Chemical, Tokyo, Japan), silica gel (100–200 mesh, Qingdao Marine Chemical Co., Ltd., Qingdao, China), octadecyl-functionalized silica gel (ODS, Cosmosil 75C_18_-Prep, Nacalai Tesque Inc., Kyoto, Japan) and sephadex LH-20 (GE Healthcare, Waukesha, WI, USA) were used for column chromatography. HPLC separation was performed on a CXTH LC-3000 preparative HPLC system (Beijing Chuangxin Tongheng Science & Technology Co. Ltd., Beijing, China) with column A (CT-30, 280 × 30 mm, 10 µm, Fuji-C_18_) and an Agilent 1260 infinity HPLC system (Agilent Technologies Co. Ltd., Santa Clara, CA, USA) using Zorbax SB-C_18_ columns (column B: 250 × 9.4 mm, 5 µm or column C: 250 × 4.6 mm, 5 µm) with a DAD detector. All solvents used for this study were purchased from the Sinopharm Chemical Reagent Co. Ltd. (Shanghai, China). Glioma U87MG (JDS-2568) and U251 (XB-0439) cells used in the experiment were purchased from the Cell Bank of the Chinese Academy of Sciences (Shanghai, China). Methicillin-resistant *Staphylococcus aureus* (MRSA) ATCC 43300 and *Escherichia coli* ATCC 25922 were gifts from Dr. Zhongjun Ma and Dr. Pinmei Wang, respectively. Doxorubicin (DOX) was purchased from Solarbio Science & Technology Co. Ltd. (Beijing, China). Vancomycin and gentamicin were ordered from Meilune Biotechnology Co., Ltd. (Dalian, China). Gauze’s agar medium was ordered from the Guangdong Huankai Microbial Science and Technology Co., Ltd. (Guangzhou, China). GYM liquid medium (glucose 4 g, yeast extract 10 g, malt extract 10 g, tap water 1.0 L) was made in the authors’ laboratory.

### 3.2. Isolation and Taxonomic Identity of Streptomyces sp. ZZ1956

The *Streptomyces* sp. ZZ1956 strain was isolated from a marine mud sample collected from the mangrove area (4.15° S, 119.61° E) of Pangkep District South Sulawesi Province, Indonesia in September 2018. Briefly, the sample (1.0 g) was suspended in sterile water to make dilutions of 10^−2^, 10^−3^ and 10^−4^ g/mL. Each dilution of 200 µL was spread over the surface of solid Gauze’s medium in a Petri dish and then incubated for 10 days at 28 °C. The single ZZ1956 colony from a Petri dish with the 10^−2^ g/mL dilution was transferred to a Gauze’s agar plate. After growth for another 7 days at 28 °C, the pure strain ZZ1956 colony (Figure S2) was transferred onto Gauze′s agar slants and stored at 4 °C for later use. The 16S rDNA sequence analysis of the strain ZZ1956 was conducted by Legenomics (Hangzhou, China). The 16S rDNA sequence of the strain ZZ1956 was deposited in GenBank with an accession number of MT672495. The voucher strain of *Streptomyces* sp. ZZ1956 was preserved at the Laboratory of the Institute of Marine Biology and Pharmacology, Ocean College, Zhoushan campus, Zhejiang University, Zhoushan, China.

### 3.3. Mass Culture of the Strain ZZ1956

Colonies of the strain ZZ1956 from the Gauze’s agar plate were inoculated into 500 mL Erlenmeyer flasks, each containing 250 mL of sterile GYM liquid medium and then incubated at 28 °C for 3 days on a rotary shaker (180 rpm) to prepare the seed broth. The seed broth (10 mL) was then transferred into a 500 mL Erlenmeyer flask containing 250 mL sterilized GYM liquid medium. A total of 60 L (240 bottles) of culture was prepared for this study and incubated at 28 °C for 15 days under shaking (180 rpm) condition.

### 3.4. Extraction and Isolation of Compounds ***1**–**30***

The 60-L culture of strain ZZ1956 was centrifuged to yield supernatant and mycelia. The mycelia were extracted with MeOH three times (3 L, each time) to give a MeOH extract solution. The supernatant was applied to a Dianion HP-20 column eluted with water and then MeOH to obtain a MeOH elution. The MeOH extract solution and MeOH elution were combined and dried in vacuo to give a crude extract, which was further partitioned with EtOAc three times to give an EtOAc extract (24 g). The EtOAc extract was subjected to a column of silica gel eluted with mixtures of cyclohexane/EtOAc (10/1, 8/1, 5/1, 2/1, 1/1, *v*/*v*), EtOAc, and MeOH to give ten fractions (Frs. A–J) based on the results of TLC and HPLC analyses.

Fr. A was purified by HPLC using column C (mobile phase: MeCN/H_2_O, 65/35; flow rate: 0.8 mL/min; UV detection: 210 nm) to give **26** (4.8 mg, t_R_ 15.4 min). Fractions B and D were separated on HPLC column B (flow rate: 1 mL/min; UV detection: 210 nm) to give **24** (3.0 mg, t_R_ 25.0 min, MeOH/H_2_O, 40/60) and **22** (4.4 mg, t_R_ 24.4 min, MeOH/H_2_O, 67/37), respectively.

Fr. C was subjected to a sephadex LH-20 column eluted with 70% MeOH to yield three subfractions (Frs. C_1_–C_3_). Fr. C_1_ was further separated on column C (mobile phase: MeOH/H_2_O, 34/66; flow rate: 0.8 mL/min; UV detection: 210 nm) to give **19** (1.2 mg, t_R_ 15.7 min) and **20** (3.8 mg, t_R_ 18.6 min). Compound **18** (1.7 mg, t_R_ 25.5 min) was obtained from Fr. C_2_ through HPLC purification using column B (mobile phase: MeOH/H_2_O, 67/33; flow rate: 1 mL/min; UV detection: 280 nm).

Each of Fr. E, Fr. F and Fr. H was separated by preparative HPLC using column A (flow rate: 10 mL/min; UV detection: 210 nm). Compounds **21** (4.9 mg, t_R_ 19.5 min), **15** (21.0 mg, t_R_ 24.3 min) and **16** (7.4 mg, t_R_ 44.1 min) were obtained from Fr. E using a mobile phase (MeOH/0.1% HOAc in H_2_O, 70/30), **14** (8.0 mg, t_R_ 26.2 min, MeOH/0.1% HOAc in H_2_O, 65/35) was purified from Fr. H and five subfractions (Frs. F_1_–F_5_) were obtained from Fr. F using a gradient mobile phase (MeOH/0.1% HOAc in H_2_O, 30/70–100/0) in 40 min. Each of Frs. F_1_–F_5_ was purified by HPLC column B (flow rate: 1 mL/min; UV detection: 210 nm) to give **1** (28.0 mg, t_R_ 23.1 min, MeCN/H_2_O, 33/67), **7** (3.3 mg, t_R_ 23.7 min, MeOH/H_2_O, 55/45), **3** (6.0 mg, t_R_ 57.5 min, MeCN/H_2_O, 30/70), **2** (19.0 mg, t_R_ 26.3 min, MeCN/H_2_O, 45/55) and **8** (3.3 mg, t_R_ 33.3 min, MeOH/H_2_O, 66/34).

Fr. G was fractionated by an ODS column eluted with 65%, 75% and 100% MeOH to give three subfractions (Frs. G_1_–G_3_) based on the results of TLC and HPLC analyses. Compound **29** (4.4 mg, t_R_ 28.2 min) was obtained from Fr. G_2_ through HPLC purification using column B (mobile phase: MeCN/0.1% HOAc in H_2_O, 30/70; flow rate: 1 mL/min; UV detection: 256 nm). Fr. G_3_ was further separated by HPLC column A (mobile phase: MeOH/0.1% HOAc in H_2_O, 70/30; flow rate: 10 mL/min; UV detection: 210 nm) to give six subfractions (Frs. G_3a_–G_3f_). Fr. G_3a_ continued to be separated on the same column A with the same flow rate and UV detection to give Fr. G_3aa_ and Fr. G_3ab_ (MeOH/0.1% HOAc in H_2_O, 55/45). Further purification by HPLC column B (flow rate: 1 mL/min; UV detection: 256 nm) yielded compounds **5** (5.9 mg, t_R_ 48.8 min, MeCN/H_2_O, 27/73) from Fr. G_3aa_, **6** (2.3 mg, t_R_ 43.7 min, MeCN/H_2_O, 33/67) from Fr. G_3ab_, **23** (2.0 mg, t_R_ 21.9 min, MeOH/0.1% HOAc in H_2_O, 65/35) from Fr. G_3b_, **4** (16.0 mg, t_R_ 24.1 min, MeCN/H_2_O, 45/55) from Fr. G_3c_, **13** (3.0 mg, t_R_ 32.3 min) and **17** (8.0 mg, t_R_ 37.1 min, MeCN/0.1% HOAc in H_2_O, 39/61) from Fr. G_3d_, **9** (2.8 mg, t_R_ 30.0 min), **10** (2.8 mg, t_R_ 38.5 min), **12** (2.5 mg, t_R_ 64.2 min) and **11** (2.2 mg, t_R_ 77.1 min, MeOH/0.1% HOAc in H_2_O, 70/30) from Fr. G_3f_.

Similarly, Fr. I was also applied to an ODS column eluted with 50%, 70% and 100% MeOH to yield three subfractions (Frs. I_1_–I_3_). Fr. I_1_ was further separated on HPLC column A (flow rate: 10 mL/min; UV detection: 210 nm) with a gradient mobile phase from 40% to 100% MeOH in 40 min to give **25** (2.0 mg, t_R_ 15.8 min); while compound **30** (20.0 mg, t_R_ 32.4 min) was obtained from Fr. I_3_ by separating on column B (mobile phase: MeOH/H_2_O, 95/5; flow rate: 1 mL/min; UV detection: 256 nm).

Finally, Fr. J was fractionated on an ODS column eluted with 30%, 40%, 60%, 70% and 100% MeOH to give Fr. J_1_ and Fr. J_2_ based on the results of HPLC analyses. Fr. J_1_ was further separated on column A (flow rate: 10 mL/min; UV detection: 210 nm) with a gradient mobile phase from 40% to 100% MeOH in 40 min to give Fr. J_1a_ and Fr. J_1b_. Compounds **27** (9.0 mg, t_R_ 24.3 min) and **28** (2.0 mg, t_R_ 42.3 min) were obtained from Fr. J_1a_ through HPLC purification using column B (mobile phase: ACN/H_2_O, 33/67; flow rate: 1 mL/min; UV detection: 210 nm).

### 3.5. Compound Characterization Data

Hygrocin K (**5**): Light yellow oil; molecular formula C_28_H_31_NO_8_; [α]_D_^20^ –65.5 (*c* 0.10, MeOH); UV (MeOH) λ_max_ (log ε) 203 (4.39), 271 (4.04), 304 (4.00) nm; IR (ATR) ν_max_ 3320, 2962, 2929, 2870, 1662, 1627, 1569, 1322, 1237, 1190, 1131, 1051, 976, 857, 733 cm^−1^; ^13^C NMR data (150 MHz), Table 1, ^1^H NMR data (600 MHz), Table 2; HRESIMS *m*/*z* 508.1969 [M–H]^−^ (calcd for C_28_H_30_NO_8_^−^, 508.1971).

Hygrocin L (**6**): Light yellow oil; molecular formula C_28_H_31_NO_8_; [α]_D_^20^ +166.6 (*c* 0.10, MeOH); UV (MeOH) λ_max_ (log ε) 203 (4.21), 270 (4.04), 304 (4.03) nm; IR (ATR) ν_max_ 3314, 2963, 2928, 2874, 1698, 1657, 1627, 1567, 1239, 1188, 1108, 1029, 976, 857, 810, 735 cm^−1^; ^13^C NMR data (150 MHz), Table 1, ^1^H NMR data (600 MHz), Table 2; HRESIMS *m*/*z* 508.1979 [M–H]^−^ (calcd for C_28_H_30_NO_8_^−^, 508.1971).

Hygrocin M (**7**): Light yellow amorphous powder; molecular formula C_28_H_29_NO_7_; [α]_D_^20^ +166.7 (*c* 0.10, MeOH); UV (MeOH) λ_max_ (log ε) 201 (4.52), 333 (4.11) nm; IR (ATR) ν_max_ 3362, 2960, 2930, 2868,1714, 1655, 1626, 1559, 1446, 1346, 1285, 1134, 1044, 860 cm^−1^; ^13^C NMR data (150 MHz), Table 1, ^1^H NMR data (600 MHz), Table 2; HRESIMS *m*/*z* 490.1875 [M–H]^−^ (calcd for C_28_H_28_NO_7_^−^, 490.1866).

Hygrocin N (**8**): Light yellow amorphous powder; molecular formula C_28_H_29_NO_7_; [α]_D_^20^ +270.0 (*c* 0.10, MeOH); UV (MeOH) λ_max_ (log ε) 201 (4.55), 336 (4.26) nm; IR (ATR) ν_max_ 3275, 2963, 2927, 2877, 1709, 1650, 1617, 1567, 1466, 1338, 1282, 1249, 1132, 1079, 859, 736 cm^−1^; ^13^C NMR data (150 MHz), Table 1, ^1^H NMR data (600 MHz), Table 2; HRESIMS *m*/*z* 492.2010 [M+H]^+^ (calcd for C_28_H_30_NO_7_, 492.2022), 514.1832 [M+Na]^+^ (calcd for C_28_H_29_NNaO_7_, 514.1842) and 490.1871 [M–H]^−^ (calcd for C_28_H_28_NO_7_^−^, 490.1866).

Hygrocin O (**9**): Red amorphous powder; molecular formula C_28_H_31_NO_8_; [α]_D_^20^ –21.5 (*c* 0.10, MeOH); UV (MeOH) λ_max_ (log ε) 201 (4.65), 334 (4.33) nm; IR (ATR) ν_max_ 3257, 2959, 2926, 2872, 1697, 1652, 1600, 1580, 1343, 1257, 1204, 1135, 1022, 978 cm^−1^; ^13^C NMR data (150 MHz), Table 1, ^1^H NMR data (600 MHz), Table 3; HRESIMS *m*/*z* 508.1975 [M–H]^−^ (calcd for C_28_H_30_NO_8_^−^, 508.1971).

Hygrocin P (**10**): Red amorphous powder; molecular formula C_28_H_31_NO_8_; [α]_D_^20^ –55.5 (*c* 0.10, MeOH); UV (MeOH) λ_max_ (log ε) 201 (4.29), 334 (4.07) nm; IR (ATR) ν_max_ 3229, 2966, 2926, 2875, 1705, 1651, 1622, 1576, 1467, 1339, 1263, 1245, 1120, 1063, 1018, 976, 855, 737 cm^−1^; ^13^C NMR data (150 MHz), Table 1, ^1^H NMR data (600 MHz), Table 3; HRESIMS *m*/*z* 508.1974 [M–H]^−^ (calcd for C_28_H_30_NO_8_^−^, 508.1971).

Hygrocin Q (**11**): Red amorphous powder; molecular formula C_29_H_33_NO_8_; [α]_D_^20^ –16.0 (*c* 0.10, MeOH); UV (MeOH) λ_max_ (log ε) 201 (4.22), 335 (4.10) nm; IR (ATR) ν_max_ 3345, 2960, 2925, 2875, 1714, 1655, 1629, 1598, 1573, 1435, 1349, 1263, 1188, 1149, 1020, 978, 851, 755 cm^−1^; ^13^C NMR data (150 MHz), Table 1, ^1^H NMR data (600 MHz), Table 3; HRESIMS *m*/*z* 522.2125 [M–H]^−^ (calcd for C_29_H_32_NO_8_^−^, 522.2128).

Hygrocin R (**12**): Red amorphous powder; molecular formula C_29_H_33_NO_8_; [α]_D_^20^ –40.0 (*c* 0.10, MeOH); UV (MeOH) λ_max_ (log ε) 201 (4.14), 335 (4.09) nm; IR (ATR) ν_max_ 3376, 2959, 2927, 2872, 1696, 1653, 1596, 1578, 1441, 1339, 1261, 1202, 1136, 1064, 976, 851, 802, 728 cm^−1^; ^13^C NMR data (150 MHz), Table 1, ^1^H NMR data (600 MHz), Table 3; HRESIMS *m*/*z* 522.2132 [M–H]^−^ (calcd for C_29_H_32_NO_8_^−^, 522.2128).

Hygrocin S (**13**): Red amorphous powder; molecular formula C_28_H_31_NO_8_; [α]_D_^20^ –21.6 (*c* 0.10, MeOH); UV (MeOH) λ_max_ (log ε) 201 (4.13), 333 (4.06) nm; IR (ATR) ν_max_ 3240, 2962, 2870, 1709, 1655, 1574, 1350, 1222, 1130, 1068, 976, 855 cm^−1^; ^13^C NMR data (150 MHz), Table 1, ^1^H NMR data (600 MHz), Table 3; HRESIMS *m*/*z* 508.1975 [M–H]^−^ (calcd for C_28_H_30_NO_8_^−^, 508.1971).

Hygrocin T (**17**): Yellow amorphous powder; molecular formula C_27_H_31_NO_7_; [α]_D_^20^ –34.0 (*c* 0.10, MeOH); UV (MeOH) λ_max_ (log ε) 204 (4.35), 211 (4.35), 279 (4.36), 308 (4.10) nm; IR (ATR) ν_max_ 3324, 2961, 2925, 2874, 1694, 1655, 1567, 1457, 1339, 1261, 1188, 1151, 1012, 973, 806, 735 cm^−1^; ^13^C NMR (150 MHz) and ^1^H NMR (600 MHz) data, Table 4; HRESIMS *m*/*z* 480.2024 [M–H]^−^ (calcd for C_27_H_30_NO_7_^−^, 480.2022).

Hygrocin U (**18**): Light yellow amorphous powder; molecular formula C_16_H_13_NO_4_; UV (MeOH) λ_max_ (log ε) 201 (4.21), 211 (4.12), 280 (4.17), 307 (3.81) nm; IR (ATR) ν_max_ 3319, 2956, 2920, 2849, 1674, 1651, 1567, 1466, 1327, 1265, 1206, 1146 cm^−1^; ^13^C NMR (150 MHz) and ^1^H NMR (600 MHz) data, Table 5; HRESIMS *m*/*z* 282.0770 [M–H]^−^ (calcd for C_16_H_12_NO_4_^−^, 282.0766).

Streptobenzenepropanamide A (**23**): White amorphous powder; molecular formula C_12_H_15_NO_2_; [α]_D_^20^ +12.4 (*c* 0.10, MeOH); ECD (15 mg/L, MeOH) λ_max_ (Δε) 215 (+20.14), 244 (–6.93) nm; UV (MeOH) λ_max_ (log ε) 202 (4.16), 248 (3.91) nm; IR (ATR) ν_max_ 3333, 2963, 2925, 2852, 1665, 1578, 1447, 1406, 1380, 1261, 1094, 1074, 965, 802, 751 cm^−1^; ^13^C NMR (150 MHz) and ^1^H NMR (600 MHz) data, Table 5; HRESIMS *m*/*z* 206.1175 [M+H]^+^ (calcd for C_12_H_16_NO_2_^+^, 206.1181) and 228.0995 [M+Na]^+^ (calcd for C_12_H_15_NNaO_2_^+^, 228.1000).

### 3.6. MTPA Esterification Hygrocin N (**8**)

Hygrocin N (**8**, **3** mg) was dissolved in 2 mL anhydrous pyridine. Half of the sample solution was added either (*R*)- or (*S*)-α-methoxy-α-(trifluoromethyl)-phenylacetyl chloride (MTPA-Cl, 45 μL). The mixture was stirred at room temperature for 24 h and then added MeOH (0.5 mL) to stop the reaction. The reaction products were separated by HPLC using column B with a flow rate of 1 mL/min and UV detection of 210 nm to furnish (*S*)-MTPA ester **8s** (1.0 mg, t_R_ 22.5 min, MeOH/H_2_O, 92/8) or (*R*)-MTPA ester **8r** (1.2 mg, t_R_ 22.5 min, MeOH/H_2_O, 92/8).

(*S*)-MTPA ester **8s**: ^1^H NMR data (600 MHz, in MeOH-*d*_4_), Table S15; HRESIMS *m*/*z* 924.2815 [M+H]^+^ (calcd for C_48_H_44_F_6_NO_11_^+^, 924.2819) and 946.2640 [M+Na]^+^ (calcd for C_48_H_43_F_6_NNaO_11_^+^, 946.2638).

(*R*)-MTPA ester **8r**: ^1^H NMR data (600 MHz, in MeOH-*d*_4_), Table S15; HRESIMS *m*/*z* 924.2824 [M+H]^+^ (calcd for C_48_H_44_F_6_NO_11_^+^, 924.2819) and 946.2628 [M+Na]^+^ (calcd for C_48_H_43_F_6_NNaO_11_^+^, 946.2638).

### 3.7. ECD Calculations

The details of ECD calculations for compound **23** were described as our previous publications [19,20].

### 3.8. Sulforhodamine B (SRB) Assay

The culture of human glioma cells and the SRB assay were according to our previous reports [19,33].

### 3.9. Antibacterail Activity Determination

The antibacterial activity of the tested compounds against MRSA and *E. coli* was evaluated by the micro broth dilution method [34] using vancomycin and gentamicin as positive controls and DMSO as a negative control.

## 4. Conclusions

Marine-derived actinomycetes from the genus *Streptomyces* continue to be one of the main resources for the discovery of novel bioactive natural products. A chemical investigation of the extract prepared from a scaled-up culture of the marine-derived actinomycete *Streptomyces* sp. ZZ1956 in GYM liquid medium resulted in the isolation and identification of thirty compounds (**1**–**30**), including twelve previously undescribed compounds, namely, hygrocins K–U (**5**–**13**, **17**, **18**) and streptophenylpropanamide A (**23**). Compounds **1**–**18** were naphthalenic ansamycin derivatives and a small change in their structures significantly influenced their antiglioma activity. Hygrocins C (**1**), D (**2**) and F (**4**) structurally characterized with ring closing and 3Z-configuration exhibited potent antiproliferative activity against both human glioma U87MG and U251 cells. Hygrocins N (**8**), O (**9**), R (**12**), T (**17**) and U (**18**), 2-amino-6-hydroxy-7-methyl-1,4-naphthoquinone (**21**), 2-acetamide-6-hydroxy-7-methyl-1,4-naphthoquinone (**22**), 3′-methoxy(1,1′,4′,1″-terphenyl)-2′,6′-diol (**26**), echoside C (**27**) and echoside A (**28**) exhibited antibacterial activity against MRSA and *E. coli*. The data from this study greatly enrich the chemical and bioactive diversities of the ansamycin antibiotics.

## Figures and Tables

**Table 1:** ^13^C NMR data of compounds **5**–**13** (150 MHz, in MeOH-d_4_, *δ*_C_).

No.	5	6	7	8	9	10	11	12	13
1	177.3, C	177.1, C	172.5, C	173.1, C	171.6, C	171.6, C	171.5, C	171.7, C	171.7, C
2	56.9, CH	56.9, CH	121.9, C	122.6, C	126.8, C	126.8, C	126.8, C	126.8, C	126.6, C
3	133.1, CH	133.6, CH	128.9, CH	127.5, CH	129.7, CH	129.7, CH	129.7, CH	129.7, CH	129.7, C
4	133.9, C	133.5, C	137.5, C	137.8, C	137.6 *^a^*, C	137.7 *^a^*, C	137.6 *^a^*, C	137.7 *^a^*, C	138.3, C
4a	13.7, CH_3_	13.6, CH_3_	21.1, CH_3_	22.1, CH_3_	16.4, CH_3_	16.4, CH_3_	16.4, CH_3_	16.4, CH_3_	21.1, CH_3_
5	167.7, C	167.3, C	167.6, C	168.3, C	168.1, C	167.9, C	168.1, C	167.9, C	168.5, C
6	75.3, CH	69.5, CH	74.7, CH	68.5, CH	75.6, CH	69.9, CH	75.5, CH	69.9, CH	75.2, CH
6a	15.2, CH_3_	19.1, CH_3_	13.5, CH_3_	19.1, CH_3_	16.4, CH_3_	19.6, CH_3_	16.2, CH_3_	19.6, CH_3_	15.0, CH_3_
7	74.2, CH	77.9, CH	71.3, CH	80.6, CH	75.4, CH	81.4, CH	75.3, CH	81.2, CH	74.4, CH
8	128.2, CH	125.3, CH	128.7, CH	124.4, CH	131.5, CH	127.9, CH	131.5, CH	127.9, CH	130.7, CH
9	138.3, CH	136.5, CH	137.3, CH	139.9, CH	138.3, CH	141.3, CH	138.1, CH	140.9, CH	137.9, CH
10	41.9, CH	41.9, CH	43.8, CH	43.4, CH	45.6, CH	45.8, CH	45.6, CH	45.8, CH	45.5, CH
10a	26.3, CH_2_	26.6, CH_2_	26.6, CH_2_	29.2, CH_2_	29.2, CH_2_	29.1, CH_2_	29.2, CH_2_	29.1, CH_2_	29.1, CH_2_
10b	10.6, CH_3_	12.4, CH_3_	12.7, CH_3_	11.5, CH_3_	12.3, CH_3_	12.3, CH_3_	12.3, CH_3_	12.3, CH_3_	12.2, CH_3_
11	29.2, CH_2_	33.0, CH_2_	32.1, CH_2_	28.9, CH_2_	31.1, CH_2_	31.2, CH_2_	31.0, CH_2_	31.0, CH_2_	30.9, CH_2_
12	41.8, CH_2_	42.2, CH_2_	39.4, CH_2_	42.5, CH_2_	33.0, CH_2_	33.2, CH_2_	33.0, CH_2_	33.0, CH_2_	33.0, CH_2_
13	212.0, C	211.2, C	212.9, C	212.0, C	177.7, C	177.8, C	176.0, C	176.0, C	177.7, C
14	130.1, C	127.2, C	129.9, C	129.2, C	113.9, CH	113.9, CH	113.9, CH	113.9, CH	113.7, CH
15	153.0, C	153.7, C	158.7, C	158.0, C	159.8, C	159.8, C	159.9, C	159.9, C	159.5, C
16	133.1 *^a^*, C	133.4, C	133.3, C	133.7, C	131.9 *^b^*, C	131.9, C	131.9, C	131.9, C	132.2, C
16a	17.0, CH_3_	16.9, CH_3_	17.3, CH_3_	17.2, CH_3_	16.8, CH_3_	16.8, CH_3_	16.8, CH_3_	16.8, CH_3_	16.7, CH_3_
17	131.5, CH	132.1, CH	131.3, CH	130.7, CH	131.7 *^b^*, CH	131.6, CH	131.6, CH	131.6, CH	131.7, CH
18	133.0 *^a^*, C	134.0, C	131.2, C	130.1, C	122.8, C	122.8, C	122.7, C	122.8, C	123.2, C
19	75.4, C	75.8, C	134.8, C	136.4, C	137.4 *^a^*, C	137.4 *^a^*, C	137.4 *^a^*, C	137.4 *^a^*, C	139.6, C
20	164.6, C	164.5, C	154.8, C	155.1, C	154.0, C	154.0, C	154.0, C	154.0, C	154.5, C
21	105.1, CH	105.3 CH	106.0, CH	105.8, CH	106.3, CH	106.4, CH	106.3, CH	106.4, CH	106.0, CH
22	185.6, C	185.9, C	187.4, C	187.0, C	186.5, C	186.4, C	186.4, C	186.4, C	186.7, C
23	129.7, C	131.1, C	129.3, C	128.4, C	131.9 *^b^*, C	131.9, C	131.9, C	131.9, C	131.7, C
24	-	-	-	-	-	-	52.1, CH_3_	52.2, CH_3_	-

*^a^*^,*b*^ The data with the same labels in each column may be interchanged.

**Table 2:** ^1^H NMR data of compounds **5**–**8** (600 MHz, in MeOH-*d*_4_, *δ*_H_, multi., *J* in Hz).

No.	5	6	7	8
2	4.06, d (11.1)	4.04, d (11.1)	-	-
3	6.09, dd (11.1, 1.8)	6.10, dd (11.1, 1.4)	6.90, s	6,81, s
4a	2.06, d (1.8)	2.03, s	2.21, s	2.19, s
6	4.65, m	3.67, m	4.87 *^a^*, m	3.69, m
6a	1.15, d (6.4)	1.02, d (6.7)	0.91, d (6.0)	0.95, d (6.4)
7	3.90, dd (6.8, 2.8)	5.11, t (5.0)	3.86, m	4.97, t (5.8)
8	4.92, dd (16.0, 6.8)	5.26, dd (16.1, 5.0)	4.59, d (15.1)	4.41, dd (15.6, 6.6)
9	5.50, dd (16.0, 5.5)	5.06, dd (16.1, 6.4)	5.30, dd (15.1, 9.2)	5.36, dd (15.6, 5.8)
10	1.84, m	1.69, m	1.55, m	1.56, m
10a	1.49, m; 1.23, m	1.32, m	1.48, m; 0.92, m	1.30, m; 1.14, m
10b	0.71, t (7.3)	0.82, t (7.4)	0.69, t (7.2)	0.76, t (7.2)
11	1.45, m; 1.19, m	1.55, m; 1.38, m	1.28, m; 1.18, m	1.53, m; 1.35, m
12	2.71, m; 2.59, m	2.67, m; 2.49, m	2.75, d (11.6)	2.81, dd (16.8, 8.5); 2.43, dd (16.8, 10.0)
16a	2.29, s	2.27, s	2.21, s	2.15, s
17	7.22, s	7.28, s	7.57, s	7.65, s
21	5.87, s	5.93, s	5.85, s	5.81, s

*^a^* The data was overlapped with that of H_2_O.

**Table 3:** ^1^H NMR data of compounds **9**–**13** (600 MHz, in MeOH-*d*_4_, *δ*_H_, multi., *J* in Hz).

No.	9	10	11	12	13
3	7.53, s	7.54, s	7.51, s	7.54, d (1.4)	7.54, s
4a	1.92, s	1.92, s	1.92, s	1.93, d (1.4)	2.24, s
6	5.05, m	3.93, m	5.06, m	3.92, m	4.84, m
6a	1.34, d (6.4)	1.24, d (6.6)	1.33, d (6.4)	1.24, d (6.4)	1.14, d (6.4)
7	4.19, t (6.3)	5.23, t (6.6)	4.19, t (6.1)	5.24, t (6.3)	4.03, t (6.0)
8	5.54, dd (15.5, 6.3)	5.57, m	5.52, dd (15.5, 6.1)	5.56, m	5.30, dd (15.5, 6.0)
9	5.48, dd (15.5, 8.6)	5.57, m	5.45, dd (15.5, 8.6)	5.57, m	5.24, dd (15.5, 8.8)
10	1.95, m	1.95, m	1.90, m	1.96, m	1.71, m
10a	1.43, m; 1.28, m	1.49, m; 1.31, m	1.45, m; 1.26, m	1.48, m; 1.29, m	1.26, m; 1.18, m
10b	0.87, t (7.1)	0.86, t (7.3)	0.86, t (7.2)	0.86, t (7.4)	0.76, t (7.4)
11	1.74, m; 1.46, m	1.76, m; 1.47, m	1.72, m; 1.41, m	1.77, m; 1.52, m	1.57, m; 1.09, m
12	2.30, m; 2.21, m	2.30, m; 2.23, m	2.28, m; 2.24, m	2.30, m; 2.26, m	2.10, m; 2.02, m
14	7.43, s	7.42, s	7.43, s	7.43, s	7.46, s
16a	2.24, s	2.21, s	2.23, s	2.21, s	2.28, s
17	7.42, s	7.39, s	7.40, s	7.40, s	6.83, s
21	5.88, s	5.88, s	5.87, s	5.88, s	5.88, s
24	-	-	3.57, s	3.65, s	-

**Table 4:** ^13^C NMR (150 MHz) and ^1^H NMR (600 MHz) data of compound **17** (in DMSO-*d*_6_).

No.	*δ*_C_, Type	*δ*_H_, Multi. (*J* in Hz)	No.	*δ*_C_, Type	*δ*_H_, Multi. (*J* in Hz)
1	171.8, C	-	10	180.1, C	-
2	37.7, CH_2_	2.90, d (7.2)	10a	138.6, C	-
3	125.5, CH	5.83, td (7.2, 1.2)	11	208.5, C	-
4	136.4, C	-	12	42.5, CH_2_	2.76, m; 2.67, m
4a	130.1, C	-	13	29.7, CH_2_	1.95, m; 1.67, m
4b	21.4, CH_3_	2.13, s	14	45.2, CH	2.00, m
5	185.4, C	-	14a	29.2, CH_2_	1.49, m; 1.28, m
5a	131.8, C	-	14b	12.3, CH_3_	0.90, t (7.5)
6	124.3, C	-	15	138.6, CH	5.47, dd (15.8, 6.8)
7	158.8, C	-	16	131.3 *^a^*, CH	5.43, dd (15.8, 6.3)
8	133.0, C	-	17	78.6, CH	3.76, t (6.3)
8a	17.0, CH_3_	2.35, s	18	72.0, CH	3.53, m
9	131.3 *^a^*, CH	7.92, s	18a	19.4, CH_3_	1.05, d (6.3)
9a	131.2 *^a^*, C	-			

*^a^* Interchangeable chemical shifts.

**Table 5:** ^13^C NMR (150 MHz) and ^1^H NMR (600 MHz) data of compounds **18** and **23** (in DMSO-*d*_6_).

No.	18		No.	23	
	*δ*_C_, Type	*δ*_H_, Multi. (*J* in Hz)		*δ*_C_, Type	*δ*_H_, Multi. (*J* in Hz)
1	169.3, C	-	1	135.6, C	-
2	36.4, CH_2_	2.83, d (7.1)	2	136.6, C	-
3	123.7, CH	5.79, t (7.1)	3	125.3, CH	7.41, d (7.5)
4	134.2, C	-	4	126.4 *^b^*, CH	7.13, m
4a	127.8, C	-	5	126.3 *^b^*, CH	7.12, m
4b	20.8, CH_3_	2.09, s	6	130.7, CH	7.15, m
5	184.2, C	-	7	37.5, CH_2_	3.08, dd (14.1, 3.6); 2.64, dd (14.1, 9.3)
5a	132.9, C	-	8	71.9, CH	3.92, ddd (9.3, 6.2, 3.6)
6	112.1, CH	7.19, s	9	176.0, C	-
7	165.6 *^a^*, C	-	10	128.6, CH	6.74, dd (15.8, 1.8)
8	131.1, C	-	11	126.7, CH	6.09, dq (15.8, 6.5)
8a	16.3, CH_3_	2.19, s	12	18.7, CH_3_	1.86, dd (6.5, 1.8)
9	129.1, CH	7.75, s	OH-8	-	5.42, d (6.2)
9a	119.6 *^a^*, C	-	NH_2_-9	-	7.25, s; 7.16, s
10	177.4, C	-			
10a	137.6, C	-			
OH-7	-	9.34, 1H, s			

*^a^* The data were observed from HMBC correlations; *^b^* The data may be interchanged.

**Table 6:** Antiglioma and antibacterial activities of compounds.

Compounds	Glioma Cells (IC_50_: μM)		Microorganisms (MIC: μg/mL)	
	U87MG	U251	MRSA	*Escherichia coli*
**1**	0.16 ± 0.01	0.35 ± 0.01	NA	NA
**2**	0.39 ± 0.04	2.63 ± 0.47	NA	NA
**4**	0.57 ± 0.09	7.33 ± 0.20	NA	NA
**8**	8.17 ± 0.17	7.04 ± 0.28	15	8
**9**	NA	NA	24	20
**11**	8.81 ± 0.80	10.46 ± 0.27	NA	NA
**12**	8.32 ± 0.38	7.86 ± 0.26	9	16
**17**	NA	NA	44	25
**18**	34.68 ± 0.58	>50	3	6
**21**	NA	NA	10	16
**22**	6.18 ± 0.18	8.13 ± 0.56	3	8
**26**	NA	NA	5	12
**27**	11.18 ± 0.92	14.64 ± 1.73	6	28
**28**	19.39 ± 0.67	13.42 ± 1.71	8	48
**30**	1.64 ± 0.06	1.35 ± 0.05	NA	NA
Doxorubicin	0.43 ± 0.01	4.18 ± 0.39	NT	NT
Vancomycin	NT	NT	0.25	NT
Gentamicin	NT	NT	0.50	0.25

NA: No activity at a concentration of 50 μM or 50 μg/mL; NT: No testing.

## Data Availability

All the data in this research are presented in manuscript and supplementary material.

## Supplementary Materials

The following supporting information can be downloaded at: https://www.mdpi.com/article/10.3390/antibiotics11111455/s1, Table S1: Sequences producing significant alignments; Table S2: Crystallographic data and structure refinement parameters of hygrocin C (**1**); Tables S3–S14: ^13^C and ^1^H NMR data of the known compounds; Table S15: ^1^H NMR data of compound **8s** and **8r**; Tables S16–S19: Data of the ECD calculations of streptobenzenepropanamide A (**23**); Figure S1: 16S rDNA sequence of *Streptomyces* sp. ZZ1956; Figure S2: Colony of strain ZZ1956 cultured in GYM medium; Figures S3–S19: NMR, HRESIMS, UV and IR spectra of hygrocin K (**5**); Figures S20–S36: NMR, HRESIMS, UV and IR spectra of hygrocin L (**6**); Figures S37–S52: NMR, HRESIMS, UV and IR spectra of hygrocin M (**7**); Figures S53–S68: NMR, HRESIMS, UV and IR spectra of hygrocin N (**8**); Figures S69–S72: ^1^H NMR and HRESIMS spectra of **8s** and **8r**; Figures S73–S88: NMR, HRESIMS, UV and IR spectra of hygrocin O (**9**); Figures S89–S104: NMR, HRESIMS, UV and IR spectra of hygrocin P (**10**); Figures S105–S118: NMR, HRESIMS, UV and IR spectra of hygrocin Q (**11**); Figures S119–S132: NMR, HRESIMS, UV and IR spectra of hygrocin R (**12**); Figures S133–S146: NMR, HRESIMS, UV and IR spectra of hygrocin S (**13**); Figures S147–S160: NMR, HRESIMS, UV and IR spectra of hygrocin T (**17**); Figures S161–S169: NMR, HRESIMS, UV and IR spectra of hygrocin U (**18**); Figures S170–S182: NMR, HRESIMS, UV and IR spectra of streptobenzenepropanamide A (**23**).
