# Supplementary material for: New Hygrocins K–U and Streptophenylpropanamide A and Bioactive Compounds from the Marine-Associated Streptomyces sp. ZZ1956
Source: Antibiotics (Basel). 2022 Oct 22;11(11):1455. doi: 10.3390/antibiotics11111455 (PMC9686540; doi:10.3390/antibiotics11111455)
Supplement: Supplementary file 1 [file antibiotics-11-01455-s001.zip › antibiotics-1975886-supplementary.pdf]

SUPPLEMENTARY MATERIALS FOR

New hygrocins K–U and streptophenylpropanamide A and bioactive compounds from the marine-associated *Streptomyces* sp. ZZ1956

Wenwen Yi[1], Asif Wares Newaz[1], Kuo Yong[1], Mingzhu Ma[1,2], Xiao-Yuan Lian[3,*] and Zhizhen Zhang[1,*]

[1] Ocean College, Zhoushan Campus, Zhejiang University, Zhoushan 316021, China
[2] Zhejiang Marine Development Research Institute, Zhoushan, 316000, China

[3] College of Pharmaceutical Sciences, Zhejiang University, Hangzhou 310058, China

*****Corresponding Authors.
Email address: xylian@zju.edu.cn (X.L.) and zzhang88@zju.edu.cn (Z. Z.); *Tel.*: +86-13675859706 (Z. Z.).

CONTENT

Figure S_1. 16S rDNA sequence of *Streptomyces* sp. ZZ1956……………………………..…..6
Figure S_2. Colony of strain ZZ1956 cultured in in Gauze′s agar medium………..…..…....6
Table S_1. Sequences producing significant alignments……………………………...…...7
Table S_2. Crystallographic data and structure refinement parameters of hygrocin C (**1**)..….7
Table S_3. ^{13}C NMR (150 MHz) and ^{1}H NMR (600 MHz) data of hygrocin C (**1**)…………8
Table S_4. ^{13}C NMR data of known compounds **2**–**4** (150 MHz, in MeOH-d_4, δ_C)……..……9
Table S_5. ^{1}H NMR data of known compounds **2**–**4** (600 MHz, in MeOH-d_4, δ_C, multi., *J* in Hz)…………………………………………………………………………….……..10
Table S_6. ^{13}C NMR (150 MHz) and ^{1}H NMR (600 MHz) data of known compound **14** (in MeOH-d_4)…………………………………………………………………………....10
Table S_7. ^{13}C NMR (150 MHz) and ^{1}H NMR (600 MHz) data of known compounds **15** and **16** (in MeOH-d_4)……………………………………………………………………..…11
Table S_8. ^{13}C NMR (150 MHz) and ^{1}H NMR (600 MHz) data of known compounds **19** and **20** (in MeOH-d_4)…………………………………………………...………..………12
Table S_9. ^{13}C NMR (150 MHz) and ^{1}H NMR (600 MHz) data of known compounds **21** and **22** (in MeOH-d_4)……………………………………………………………..……………12
Table S_{10}. ^{13}C NMR (150 MHz) and ^{1}H NMR (600 MHz) data of known compounds **24** and **25** (in MeOH-d_4)………………………………………………………………….…13
Table S_{11}. ^{13}C NMR data of known compounds **26**–**28** (150 MHz, δ_C)……..……………14
Table S_{12}. ^{1}H NMR data of known compounds **26**–**28** (600 MHz, δ_H, multi., *J* in Hz)…....15
Table S_{13}. ^{13}C NMR (150 MHz) and ^{1}H NMR (600 MHz) data of known compound **29** (in MeOH-d_4)……………………………………………………………………………16
Table S_{14}. ^{13}C NMR (150 MHz) and ^{1}H NMR (600 MHz) data of known compound **30** (in DMSO-d_6)…………………………………………………………………….…17
Table S_{15}. ^{1}H NMR data of compound **8s** and **8r** (600 MHz, in MeOH-d_4) ………………17
Figures S_{3-5}. ^{1}H NMR spectra of hygrocin K (**5**)……………….…………….............18
Figures S_{6-8}. ^{13}C NMR spectra of hygrocin K (**5**)…………………………….……......19
Figures S_{9-11}. HMQC spectra of hygrocin K (**5**)……………………………….…….........21
Figure S_{12}. COSY spectrum of hygrocin K (**5**)…………………………………….....22
Figures S_{13-15}. HMBC spectra of hygrocin K (**5**)…………………………………………23
Figure S_{16}. NOESY spectrum of hygrocin K (**5**)………...…………………………..24
Figure S_{17}. HRESIMS spectrum of hygrocin K (**5**)……...………………………..25
Figure S_{18}. UV spectrum of hygrocin K (**5**)……………………………….…….25
Figure S_{19}. IR spectrum of hygrocin K (**5**)…………………………………..…25
Figures S_{20-22}. ^{1}H NMR spectra of hygrocin L (**6**)……………………………………....26
Figures S_{23-25}. ^{13}C NMR spectra of hygrocin L (**6**)………………..……………..…...27

Figures S$_{26-28}$. HMQC spectra of hygrocin L (**6**)……29
Figure S$_{29}$. COSY spectrum of hygrocin L (**6**)……30
Figures S$_{30-32}$. HMBC spectra of hygrocin L (**6**)……31
Figure S$_{33}$. NOESY spectrum of hygrocin L (**6**)……32
Figure S$_{34}$. HRESIMS spectrum of hygrocin L (**6**)……33
Figure S$_{35}$. UV spectrum of hygrocin L (**6**)……33
Figure S$_{36}$. IR spectrum of hygrocin L (**6**)……33
Figures S$_{37-39}$. ^{1}H NMR spectra of hygrocin M (**7**)……34
Figures S$_{40-42}$. ^{13}C NMR spectra of hygrocin M (**7**)……35
Figures S$_{43-45}$. HMQC spectra of hygrocin M (**7**)……37
Figure S$_{46}$. COSY spectrum of hygrocin M (**7**)……38
Figures S$_{47-48}$. HMBC spectra of hygrocin M (**7**)……39
Figures S$_{49}$. NOESY spectrum of hygrocin M (**7**)……40
Figure S$_{50}$. HRESIMS spectra of hygrocin M (**7**)……40
Figure S$_{51}$. UV spectrum of hygrocin M (**7**)……40
Figure S$_{52}$. IR spectrum of hygrocin M (**7**)……41
Figures S$_{53-55}$. ^{1}H NMR spectra of hygrocin N (**8**)……41
Figures S$_{56-58}$. ^{13}C NMR spectra of hygrocin N (**8**)……43
Figures S$_{59-61}$. HMQC spectra of hygrocin N (**8**)……44
Figure S$_{62}$. COSY spectrum of hygrocin N (**8**)……46
Figures S$_{63-64}$. HMBC spectra of hygrocin N (**8**)……46
Figure S$_{65}$. NOESY spectrum of hygrocin N (**8**)……47
Figure S$_{66}$. HRESIMS spectrum of hygrocin N (**8**)……48
Figure S$_{67}$. UV spectrum of hygrocin N (**8**)……48
Figure S$_{68}$. IR spectrum of hygrocin N (**8**)……49
Figure S$_{69}$. ^{1}H NMR spectrum of **8s** (600 MHz, in MeOH-d_4)……49
Figure S$_{70}$. HRESIMS spectrum of **8s**……50
Figure S$_{71}$. ^{1}H NMR spectrum of **8r** (600 MHz, in MeOH-d_4)……50
Figure S$_{72}$. HRESIMS spectrum of **8r**……50
Figures S$_{73-75}$. ^{1}H NMR spectra of hygrocin O (**9**)……51
Figures S$_{76-78}$. ^{13}C NMR spectra of hygrocin O (**9**)……52
Figures S$_{79-81}$. HMQC spectra of hygrocin O (**9**)……54
Figure S$_{82}$. COSY spectrum of hygrocin O (**9**)……55
Figures S$_{83-84}$. HMBC spectra of hygrocin O (**9**)……56
Figure S$_{85}$. NOESY spectrum of hygrocin O (**9**)……57
Figure S$_{86}$. HRESIMS spectrum of hygrocin O (**9**)……57
Figure S$_{87}$. UV spectrum of hygrocin O (**9**)……58

Figure S_{88}. IR spectrum of hygrocin O (**9**)……58
Figures S_{89-91} ^{1}H NMR spectra of hygrocin P (**10**)……59
Figures S_{92-94}. ^{13}C NMR spectra of hygrocin P (**10**)……60
Figures S_{95-97}. HMQC spectra of hygrocin P (**10**)……62
Figure S_{98}. COSY spectrum of hygrocin P (**10**)……63
Figures S_{99-100}. HMBC spectra of hygrocin P (**10**)……64
Figure S_{101}. NOESY spectrum of hygrocin P (**10**)……65
Figure S_{102}. HRESIMS spectrum of hygrocin P (**10**)……65
Figure S_{103}. UV spectrum of hygrocin P (**10**)……66
Figure S_{104}. IR spectrum of hygrocin P (**10**)……66
Figures $S_{105-107}$. ^{1}H NMR spectra of hygrocin Q (**11**)……67
Figures $S_{108-110}$. ^{13}C NMR spectra of hygrocin Q (**11**)……68
Figure S_{111}. HMQC spectra of hygrocin Q (**11**)……70
Figure S_{112}. COSY spectrum of hygrocin Q (**11**)……70
Figures $S_{113-114}$. HMBC spectra of hygrocin Q (**11**)……71
Figure S_{115}. NOESY spectrum of hygrocin Q (**11**)……72
Figure S_{116}. HRESIMS spectrum of hygrocin Q (**11**)……72
Figure S_{117}. UV spectrum of hygrocin Q (**11**)……73
Figure S_{118}. IR spectrum of hygrocin Q (**11**)……73
Figures $S_{119-121}$. ^{1}H NMR spectra of hygrocin R (**12**)……74
Figures $S_{122-124}$. ^{13}C NMR spectra of hygrocin R (**12**)……75
Figure S_{125}. HMQC spectrum of hygrocin R (**12**)……77
Figure S_{126}. COSY spectrum of hygrocin R (**12**)……77
Figures $S_{127-128}$. HMBC spectra of hygrocin R (**12**)……78
Figure S_{129}. NOESY spectrum of hygrocin R (**12**)……79
Figure S_{130}. HRESIMS spectrum of hygrocin R (**12**)……79
Figure S_{131}. UV spectrum of hygrocin R (**12**)……80
Figure S_{132}. IR spectrum of hygrocin R (**12**)……80
Figures $S_{133-135}$. ^{1}H NMR spectra of hygrocin S (**13**)……81
Figures $S_{136-138}$. ^{13}C NMR spectra of hygrocin S (**13**)……82
Figure S_{139}. HMQC spectrum of hygrocin S (**13**)……84
Figure S_{140}. COSY spectrum of hygrocin S (**13**)……84
Figures $S_{141-142}$. HMBC spectra of hygrocin S (**13**)……85
Figure S_{143}. NOESY spectrum of hygrocin S (**13**)……86
Figure S_{144}. HRESIMS spectrum of hygrocin S (**13**)……86
Figure S_{145}. UV spectrum of hygrocin S (**13**)……87
Figure S_{146}. IR spectrum of hygrocin S (**13**)……87

Figures $S_{147-149}$. ^{1}H NMR spectra of hygrocin T (**17**)……………………88
Figures $S_{150-152}$. ^{13}C NMR spectra of hygrocin T (**17**)……………………89
Figure S_{153}. HMQC spectrum of hygrocin T (**17**)……………………91
Figure S_{154}. COSY spectrum of hygrocin T (**17**)……………………91
Figures $S_{155-156}$. HMBC spectra of hygrocin T (**17**)……………………92
Figure S_{157}. NOESY spectrum of hygrocin T (**17**)……………………93
Figure S_{158}. HRESIMS spectrum of hygrocin T (**17**)……………………93
Figure S_{159}. UV spectrum of hygrocin T (**17**)……………………94
Figure S_{160}. IR spectrum of hygrocin T (**17**)……………………94
Figure S_{161}. ^{1}H NMR spectra of hygrocin U (**18**)……………………95
Figure S_{162}. ^{13}C NMR spectra of hygrocin U (**18**)……………………95
Figure S_{163}. HMQC spectra of hygrocin U (**18**)……………………96
Figure S_{164}. COSY spectrum of hygrocin U (**18**)……………………96
Figures $S_{165-166}$. HMBC spectra of hygrocin U (**18**)……………………97
Figure S_{167}. HRESIMS spectrum of hygrocin U (**18**)……………………98
Figure S_{168}. UV spectrum of hygrocin U (**18**)……………………98
Figure S_{169}. IR spectrum of hygrocin U (**18**)……………………98
Figures $S_{170-172}$. ^{1}H NMR spectra of streptobenzenepropanamide A (**23**)……………………99
Figures $S_{173-174}$. ^{13}C NMR spectra of streptobenzenepropanamide A (**23**)……………………100
Figures $S_{175-176}$. HMQC spectra of streptobenzenepropanamide A (**23**)……………………101
Figure S_{177}. COSY spectrum of streptobenzenepropanamide A (**23**)……………………102
Figures $S_{178-179}$. HMBC spectra of streptobenzenepropanamide A (**23**)……………………103
Figure S_{180}. HRESIMS spectrum of streptobenzenepropanamide A (**23**)……………………104
Figure S_{181}. UV spectrum of streptobenzenepropanamide A (**23**)……………………104
Figure S_{182}. IR spectrum of streptobenzenepropanamide A (**23**)……………………104
Table S_{16}. Gibbs free energies and equilibrium populations of low-energy conformers of *R*-**23**……………………105
Table S_{17}. Cartesian coordinates for the low-energy reoptimized MMFF conformers of *R*-**23** at B3LYP/6-311+G (d, p) level of theory in MeOH……………………105
Table S_{18}. Gibbs free energies and optical rotation of low-energy conformers of *S*-**23**……………………109
Table S_{19}. Cartesian coordinates for the low-energy reoptimized MMFF conformers of *S*-**23** at B3LYP/6-311+G (d, p) level of theory in MeOH……………………109

Figure S_1. 16S rDNA sequence of *Streptomyces* sp. ZZ1956

CTTACCATGCAGTCGAACGATGAACCGGTTTCGGCCGGGGATTAGTGGCGAACGGGTG
AGTAACACGTGGGCAATCTGCCCTGCACTCTGGGACAAGCCCTGGAAACGGGGTCTAA
TACCGGATATGACGCGTTCCCGCATGGGATACGTGTGGAAAGCTCCGGCGGTGCAGGA
TGAGCCCGCGGCCTATCAGCTTGTTGGTGGGGTGATGGCCTACCAAGGCGACGACGGG
TAGCCGGCCTGAGAGGGCGACCGGCCACACTGGGACTGAGACACGGCCCAGACTCCT
ACGGGAGGCAGCAGTGGGGAATATTGCACAATGGGCGCAAGCCTGATGCAGCGACGC
CGCGTGAGGGATGACGGCCTTCGGGTTGTAAACCTCTTTCAGCAGGGAAGAAGCGTG
AGTGACGGTACCTGCAGAAGAAGCGCCGGCTAACTACGTGCCAGCAGCCGCGGTAATA
CGTAGGGCGCAAGCGTTGTCCGGAATTATTGGGCGTAAAGAGCTCGTAGGCGGCTTGT
CGCGTCGGATGTGAAAGCCCGGGGCTTAACTCCGGGTCTGCATTCGATACGGGCAGGC
TAGAGTTCGGTAGGGGAGATCGGAATTCCTGGTGTAGCGGTGAAATGCGCAGATATCA
GGAGGAACACCGGTGGGCGAAGGCGGATCTCTGGGCCGATACTGACGCTGAGGAGCG
AAAGCGTGGGGAGCGAACAGGATTAGATACCCTGGTAGTCCACGCCGTAAACGTTGGG
AACTAGGTGTGGGCGACATTCCACGTTGTCCGTGCCGCAGCTAACGCATTAAGTTCCCC
GCCTGGGGAGTACGGCCGCAAGGCTAAAACTCAAAGGAATTGACGGGGGCCCGCACA
AGCGGCGGAGCATGTGGCTTAATTCGACGCAACGCGAAGAACCTTACCAAGGCTTGAC
ATACATCGGAAACATCCAGAGATGGGTGCCCCCTTGTGGTCGGTGTACAGGTGGTGCAT
GGCTGTCGTCAGCTCGTGTCGTGAGATGTTGGGTTAAGTCCCGCAACGAGCGCAACCC
TTGTCCTGTGTTGCCAGCGGGTTATGCCGGGGACTCACAGGAGACTGCCGGGGTCAAC
TCGGAGGAAGGTGGGGACGACGTCAAGTCATCATGCCCCTTATGTCTTGGGCTGCACA
CGTGCTACAATGGCCGGTACAATGAGCTGCGAAGCCGTGAGGTGGAGCGAATCTCAAA
AAGCCGGTCTCAGTTCGGATTGGGGTCTGCAACTCGACCCCATGAAGTCGGAGTCGCT
AGTAATCGCAGATCAGCATTGCTGCGGTGAATACGTTCCCGGGCCTTGTACACACCGCC
CGTCACGTCACGAAAGTCGGTAACACCCGAAGCCGGTGGCCCAACCCTTGTGGAGGG
AGCCGTCGAAGGGA (1403 bp).

Figure S_2. Colony of strain ZZ1956 cultured in Gauze′s agar medium

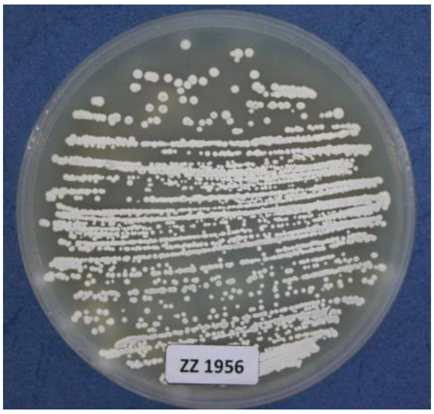

Table S1. Sequences producing significant alignments

Accession	Description	Max score	Total score	Query coverage	E value	Ident
EU603353.1	*Streptomyces* sp. MJM4686 16S ribosomal RNA gene	2575	2575	99%	0.0	99.86%
KY213669.1	*Streptomyces* sp. strain T17 16S ribosomal RNA gene	2571	2571	99%	0.0	99.86%
MN400077.1	*Streptomyces* sp. strain H4 16S ribosomal RNA gene	2569	2569	99%	0.0	99.79%
FJ532411.1	*Streptomyces malaysiensis* strain HBUM175125 16S ribosomal RNA gene	2564	2564	99%	0.0	99.79%
CP029823.1	*Streptomyces malaysiensis* strain DSM 4137 chromosome	2562	15247	99%	0.0	99.64%
KY213677.1	*Streptomyces malaysiensis* strain T44 16S ribosomal RNA gene	2562	2562	99%	0.0	99.64%

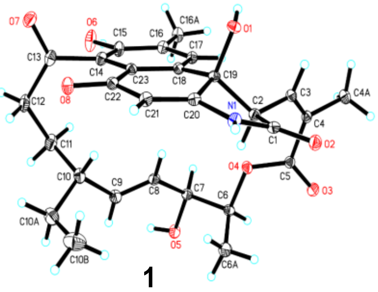

1

Table S2. Crystallographic data and structure refinement parameters of hygrocin C (**1**)

Empirical formula	$C_{28}H_{31}NO_8$	F(000)	540.0
Formula weight	509.54	Crystal size/mm^3	0.13 × 0.12 × 0.1
Temperature/K	100.0(3)	Radiation	Cu Kα (λ = 1.54184)
Crystal system	monoclinic	2Θ range for data collection/°	6.548 to 146.924
Space group	$P2_1$	Index ranges	$-11 \leq h \leq 9$, $-12 \leq k \leq 12$, $-16 \leq l \leq 16$
a/Å	9.5820(7)	Reflections collected	8536
b/Å	9.9202(6)	Independent reflections	4619 [R_{int} = 0.0295, R_{sigma} = 0.0299]
c/Å	13.4974(8)	Data/restraints/parameters	4619/1/341
α/°	90	Goodness-of-fit on F^2	1.047
β/°	90.04(4)	Final R indexes [I>=2σ (I)]	R_1 = 0.0356, wR_2 = 0.0922
γ/°	90	Final R indexes [all data]	R_1 = 0.0359, wR_2 = 0.0927
Volume/Å^3	1283.00(14)	Largest diff. peak/hole / e Å^{-3}	0.18/-0.23
Z	2	Flack/Hooft parameter	0.00(9)/0.03(7)
ρ_{calc}g/cm^3	1.319	F(000)	540.0
μ/mm^{-1}	0.802	Crystal size/mm^3	0.13 × 0.12 × 0.1

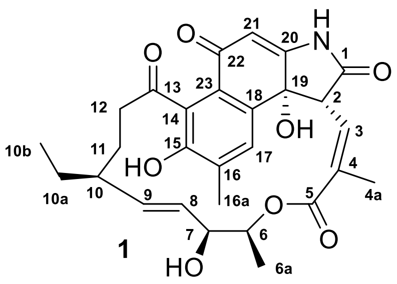

Table S3. ^{13}C NMR (150 MHz) and ^{1}H NMR (600 MHz) data of hygrocin C (**1**)

No.	**1**[a]		**1**[b]	
	δ_C, type	δ_H, multi. (J in Hz)	δ_C, type	δ_H, multi. (J in Hz)
1	174.3, C	–	177.5, C	–
2	53.5, CH	4.65, d (10.6)	55.2, CH	4.60, d (10.7)
3	130.0, CH	6.57, dd (10.6, 1.3)	131.2, CH	6.54, dd (10.7, 1.4)
4	135.1, C	–	137.0, C	–
4a	21.1, CH_3	2.19, d (1.3)	22.2, CH_3	2.20, d (1.4)
5	166.4, C	–	168.3, C	–
6	73.6, CH	4.86, qd (6.4, 4.0)	75.3, CH	4.81, qd (6.4, 4.0)
6a	13.1, CH_3	1.03, d (6.4)	14.4, CH_3	1.03, d (6.4)
7	70.0, CH	3.93, dd (5.6, 3.3)	71.9, CH	3.87, td (5.7, 3.9)
8	127.4, CH	4.18, dd (15.3, 3.3)	128.3, CH	4.14, dd (15.2, 3.9)
9	135.2, CH	5.25, ddd (15.3, 9.6, 1.8)	137.3, CH	5.24, ddd (15.2, 9.2, 1.8)
10	44.8, CH	1.43, m	45.8, CH	1.37–1.40, m
10a	26.0, CH_2	1.45, m; 0.90, m	27.1, CH_2	1.46, m; 0.93, m
10b	12.1, CH_3	0.62, t (7.1)	13.2, CH_3	0.66, t (7.4)
11	30.4, CH_2	1.40, m	31.6, CH_2	1.37–1.40, m
12	39.6, CH_2	2.83, ddd (17.3, 11.4, 2.0); 2.48, ddd (17.3, 6.6, 2.0)	40.6, CH_2	2.89, dd (17.6, 11.5); 2.60, dd (17.6, 6.1)
13	206.3, C	–	212.5, C	–
14	128.2, C	–	129.9, C	–
15	152.3, C	–	153.7, C	–
16	130.5, C	–	132.7, C	–
16a	15.9, CH_3	2.25, s	17.1, CH_3	2.22, s
17	129.7, CH	7.48, s	131.1, CH	7.41, s
18	132.8, C	–	134.3, C	–
19	72.6, C	–	73.9, C	–
20	161.6, C	–	164.0, C	–
21	102.9, CH	5.82, s	104.1, CH	5.81, s
22	183.0, C	–	185.6, C	–
23	129.3, C	–	130.2, C	–

[a,b] The data were recorded in acetone-d_6 and MeOH-d_4, respectively.

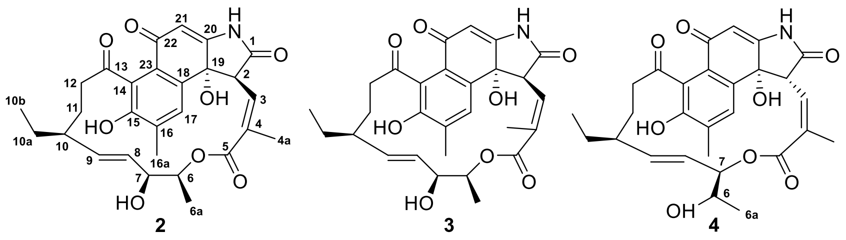

Table S4. ^{13}C NMR data of known compounds **2**–**4** (150 MHz, in MeOH-d_4, δ_C)

No.	**2**	**3**	**4**
1	177.3, C	176.9, C	178.6, C
2	56.1, CH	55.6, CH	56.3, CH
3	132.8, CH	133.0, CH	131.8, CH
4	135.5, C	134.6, C	137.3, C
4a	21.6, CH_3	13.6, CH_3	21.8, CH_3
5	168.7, C	167.4, C	168.1, C
6	76.2, CH	74.5, CH	67.4, CH
6a	13.8, CH_3	13.2, CH_3	17.1, CH_3
7	71.7, CH	71.8, CH	79.1, CH
8	128.1, CH	128.3, CH	125.9, CH
9	137.9, CH	136.4, CH	138.6, CH
10	44.2, CH	43.2, CH	42.5, CH
10a	29.7, CH_2	27.8, CH_2	29.1, CH_2
10b	12.8, CH_3	11.2, CH_3	11.9, CH_3
11	35.0, CH_2	31.6, CH_2	31.4, CH_2
12	42.9, CH_2	42.1, CH_2	43.9, CH_2
13	212.1, C	212.0, C	212.1, C
14	128.6, C	129.8, C	126.8, C
15	153.6, C	153.0, C	153.3, C
16	133.3, C	133.7, C	133.4, C
16a	17.1, CH_3	17.0, CH_3	17.1, CH_3
17	130.5, CH	132.0, CH	132.2, CH
18	134.4, C	133.0, C	134.7, C
19	73.8, C	75.3, C	75.1, C
20	164.6, C	164.7, C	164.7, C
21	103.7, CH	104.5, CH	104.1, CH
22	185.7, C	185.8, C	185.8, C
23	129.5, C	130.7, C	130.2, C

Table S5. ^{1}H NMR data of known compounds **2**–**4** (600 MHz, in MeOH-d_4, δ_H, multi., J in Hz)

No.	**2**	**3**	**4**
2	4.22, d (10.8)	4.06, d (9.2)	4.94, d (10.1)
3	6.45, d (10.8)	5.89, dd (9.2, 1.3)	6.53, d (10.1)
4a	2.19, s	2.09, d (1.3)	2.19, s
6	4.79, qd (6.1, 3.5)	4.67, qd (6.2, 4.3)	3.90, dq (6.2, 4.1)
6a	0.95 d (6.1)	0.93, d (6.2)	0.99, d (6.2)
7	3.96, d (1.4)	4.09, m	5.07, t (4.6)
8	3.84, d (15.3)	4.92, ddd (15.6, 3.2, 1.0)	4.28, dd (16.1, 5.0)
9	5.18, ddd (15.3, 9.7, 2.1)	5.51, ddd (15.6, 7.3, 1.8)	5.21, dd (16.1, 7.2)
10	1.53, 1H, m	1.83, m	1.53, m
10a	1.33, m; 1.08, m	1.45, m; 1.26, m	1.38, m; 1.10, m
10b	0.72, t (7.4)	0.78, t (7.4)	0.75, t (7.3)
11	1.76, m; 1.36, m	1.53, m; 1.29, m	1.54, m; 1.40, m
12	2.62, ddd (15.2, 11.9, 2.4); 2.46, ddd (15.2, 9.6, 2.4)	2.79, m; 2.48, m	2.84, ddd (16.2, 8.8, 2.3); 2.44, ddd (16.2, 8.8, 2.3)
16a	2.28, s	2.30, s	2.23, s
17	7.54, s	7.25, s	7.36, s
21	5.79, s	5.85, s	5.80, s

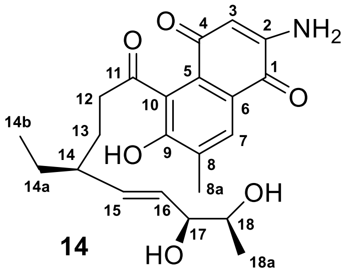

14

Table S6. ^{13}C NMR (150 MHz) and ^{1}H NMR (600 MHz) data of known compound **14** (in MeOH-d_4)

No.	δ_C, type	δ_H, multi. (J in Hz)	No.	δ_C, type	δ_H, multi. (J in Hz)
1	181.3, C	–	11	209.8, C	–
2	153.1, C	–	12	42.8, CH_2	2.68, m
3	102.1, CH	5.71, s	13	29.8, CH_2	1.65, m; 1.46, m
4	185.5, C	–	14	45.3, CH	1.96, m
5	131.7, C	–	14a	29.3, CH_2	1.91, m; 1.31, m
6	131.1, C	–	14b	12.3, CH_3	0.90, t (7.3)
7	131.2, CH	7.81, s	15	138.6, CH	5.45, m
8	132.6, C	–	16	131.7, CH	5.45, m
8a	16.9, CH_3	2.29, s	17	78.7, CH	3.76, m
9	160.4, C	–	18	72.0, CH	3.53, m
10	123.5, C	–	18a	19.3, CH_3	1.05, d (6.1)

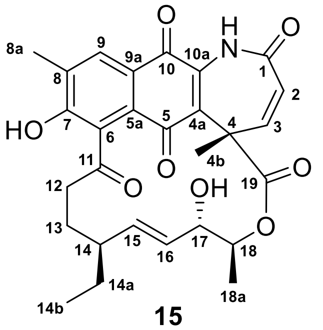

15

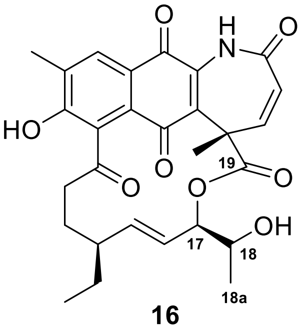

16

Table S7. ^{13}C NMR (150 MHz) and ^{1}H NMR (600 MHz) data of known compounds **15** and **16** (in MeOH-d_4)

No.	**15**		**16**	
	δ_C, type	δ_H, multi. (J in Hz)	δ_C, type	δ_H, multi. (J in Hz)
1	166.4, C	–	166.7, C	–
2	123.2, CH	6.01, d (12.3)	123.9, C	6.03, dd (12.1, 3.0)
3	146.4, CH	6.17, d (12.3)	147.6, CH	6.48, dd (12.1, 3.0)
4	52.6, C	–	51.2, C	–
4a	131.8, C	–	131.3, C	–
4b	26.0, CH_3	1.57, s	24.4, CH_3	1.51, s
5	185.1, C	–	184.0, C	–
5a	126.4, C	–	126.3, C	–
6	123.4, C	–	123.3, C	–
7	159.3, C	–	159.2, C	–
8	133.2, C	–	133.6, C	–
8a	17.1, CH_3	2.34, s	17.1, CH_3	2.34, s
9	131.9, CH	7.96, s	131.7, CH	7.95, s
9a	130.3, C	–	130.4, C	–
10	179.8, C	–	179.8, C	–
10a	137.1, C	–	137.9, C	–
11	208.8, C	–	207.5, C	–
12	41.7, CH_2	2.78, m; 2.73, m	41.7, CH_2	2.67, m; 2.35, m
13	29.8, CH_2	1.98, m; 1.50, m	38.6, CH_2	2.40, m; 1.39, m
14	44.7, CH	1.98, m	45.5, CH	2.89, m
14a	28.4, CH_2	1.49, m; 1.27, m	25.3, CH_2	1.40, m; 1.29, m
14b	12.2, CH_3	0.79, t (7.3)	12.6, CH_3	0.89, t (7.3)
15	136.9, CH	5.28, dd (15.8, 6.8)	142.8, CH	5.97, dd (15.5, 6.8)
16	132.7, CH	5.24, dd (15.8, 6.2)	125.8, CH	5.36, m
17	74.9, CH	3.98, dd (6.3, 3.5)	80.8, CH	5.36, m
18	76.8, CH	4.86, m	69.7, CH	3.79, m
18a	16.8, CH_3	1.29, d (7.3)	20.3, CH_3	1.12, dd (6.5)
19	174.4, C	–	174.6, C	–

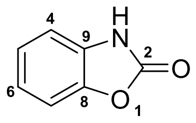

19

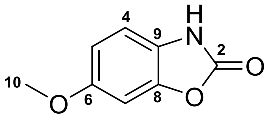

20

Table S8. ^{13}C NMR (150 MHz) and ^{1}H NMR (600 MHz) data of known compounds **19** and **20** (in DMSO-d_6)

No.	**19**		**20**	
	δ_C, type	δ_H, multi. (J in Hz)	δ_C, type	δ_H, multi. (J in Hz)
2	155.7, C	–	154.9, C	–
4	109.9, CH	7.07, d (7.8)	109.8, CH	6.98, dd (7.8, 2.7)
5	123.3, CH	7.12, t (7.8)	128.9, CH	6.69, td (7.8, 2.7)
6	121.0, CH	7.05, t (7.8)	155.5, C	–
7	109.0, CH	7.26, d (7.8)	96.8, CH	6.96, d (2.7)
8	143.9, C	–	144.3, C	–
9	132.5, C	–	124.9, C	–
10	–	–	56.8, CH_3	3.73, s

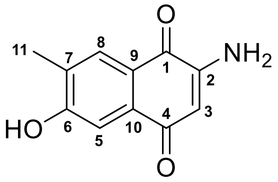

21

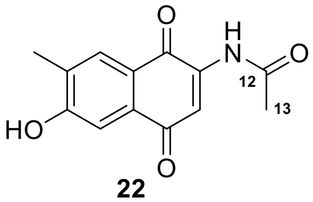

22

Table S9. ^{13}C NMR (150 MHz) and ^{1}H NMR (600 MHz) data of known compounds **21** and **22** (in DMSO-d_6)

No.	**21**		**22**	
	δ_C, type	δ_H, multi. (J in Hz)	δ_C, type	δ_H, multi. (J in Hz)
1	180.5, C	–	179.0, C	–
2	150.7, C	–	141.4, C	–
3	101.4, CH	5.67, s	115.3, CH	7.53, s
4	181.9, C	–	185.4, C	–
5	110.8, CH	7.26, s	110.7, CH	7.27, s
6	161.9, C	–	162.4, C	–
7	128.2, C	–	130.4, C	–
8	129.1, CH	7.69, s	129.5, CH	7.77, s
9	122.0, C	–	121.4, C	–
10	134.0, C	–	131.9, C	–
11	15.8, CH_3	2.19, s	16.0, CH_3	2.22, s
12	–	–	171.2, C	–
13	–	–	24.6, CH_3	2.22, s
OH-6	–	10.92, s	–	9.70, s

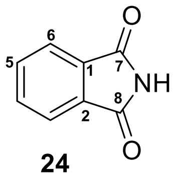

24

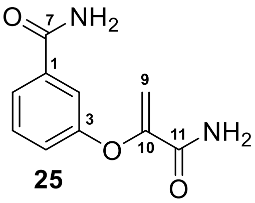

25

Table S10. ^{13}C NMR (150 MHz) and ^{1}H NMR (600 MHz) data of known compounds **24** and **25** (in MeOH-d_4)

No.	**24**		**25**	
	δ_C, type	δ_H, multi. (J in Hz)	δ_C, type	δ_H, multi. (J in Hz)
1	134.5, C	–	137.3, C	–
2	134.5, C	–	120.7, CH	7.63, t (2.0)
3	124.2, CH	7.83, m	156.7, C	–
4	135.3, CH	7.80, m	124.8, CH	7.30, dd (8.0, 2.0)
5	135.3, CH	7.80, m	131.4, CH	7.50, t (8.0)
6	124.2, CH	7.83, m	125.2, CH	7.71, td (8.0, 2.0)
7	171.2, C	–	171.3, C	–
8	171.2, C	–	–	–
9	–	–	99.5, CH_2	5.57, d (2.3); 4.59, d (2.3)
10	–	–	155.5, C	–
11	–	–	166.7, C	–

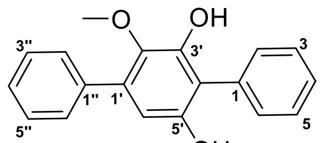

26

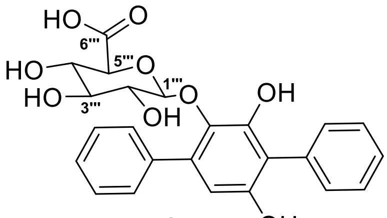

27

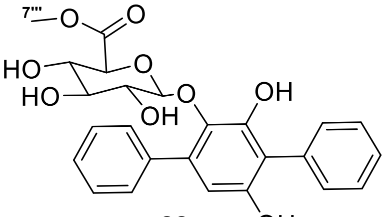

28

Table S_{11}. ^{13}C NMR data of known compounds **26**–**28** (150 MHz, δ_C)

No.	**26**^{a}	**27**^{b}	**28**^{b}
1	133.0, C	135.8, C	135.8, C
2	130.9, CH	132.3, CH	132.3, CH
3	127.3, CH	128.9, CH	128.8, CH
4	126.1, CH	127.8, CH	127.8, CH
5	127.3, CH	128.9, CH	128.8, CH
6	130.9, CH	132.3, CH	132.3, CH
1′	134.7, C	136.8, C	136.8, C
2′	137.8, C	137.4, C	137.5, C
3′	148.2, C	149.6, C	149.5, C
4′	116.2, C	118.2, C	118.1, C
5′	151.1, C	153.0, C	153.1, C
6′	106.9, CH	109.1, CH	109.0, CH
OCH_3-2′	60.3, CH_3	–	–
1″	138.2, C	139.8, C	140.0, C
2″	128.4, CH	130.8, CH	130.8, CH
3″	128.3, CH	128.8, CH	128.7, CH
4″	127.1, CH	128.2, CH	128.0, CH
5″	128.3, CH	128.8, CH	128.7, CH
6″	128.4, CH	130.8, CH	130.8, CH
1‴	–	107.6, CH	108.0, CH
2‴	–	75.0, CH	75.0, CH
3‴	–	77.1, CH	77.2, CH
4‴	–	72.9, CH	72.8, CH
5‴	–	77.3, CH	77.2, CH
6‴	–	171.9, C	170.5, C
7‴	–	–	52.9, CH_3

^{a,b} The data were recorded in DMSO-d_6 and MeOH-d_4, respectively.

Table S12. ^{1}H NMR data of known compounds **26–28** (600 MHz, δ_H, multi., *J* in Hz)

No.	**26**[a]	**27**[b]	**28**[b]
2	7.35, d (7.7)	7.41, d (7.5)	7.41, d (7.5)
3	7.35, t (7.7)	7.36, t (7.5)	7.36, t (7.5)
4	7.24, t (7.7)	7.26, t (7.5)	7.26, t (7.3)
5	7.35, t (7.7)	7.36, t (7.5)	7.36, t (7.5)
6	7.35, d (7.7)	7.41, d (7.5)	7.41, d (7.5)
6′	6.37, s	6.38, s	6.37, s
OCH_3-2′	3.27, s	–	–
2″	7.53, d (7.4)	7.48, d (7.3)	7.44, d (7.3)
3″	7.44, t (7.4)	7.32, t (7.3)	7.30, t (7.3)
4″	7.35, t (7.4)	7.26, t (7.3)	7.26, t (7.3)
5″	7.44, t (7.4)	7.32, t (7.3)	7.30, t (7.3)
6″	7.53, d (7.4)	7.48, d (7.3)	7.44, d (7.3)
1‴	–	4.36, d (7.8)	4.33, d (7.8)
2‴	–	3.36, t (9.2)	3.36, dd (9.2, 7.8)
3‴	–	3.25, t (9.2)	3.24, t (9.2)
4‴	–	3.44, t (9.2)	3.41, t (9.2)
5‴	–	3.52, d (9.8)	3.51, d (9.8)
7‴	–	–	3.65, s
OH-3′	9.08, s	–	–
OH-5′	8.59, s	–	–

[a,b] The data were recorded in DMSO-d_6 and MeOH-d_4, respectively.

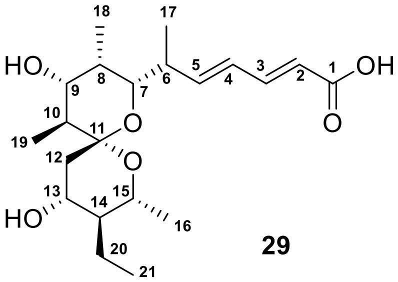

29

Table S13. ^{13}C NMR (150 MHz) and ^{1}H NMR (600 MHz) data of known compound **29** (in MeOH-d_4)

No.	δ_C, type	δ_H, multi. (*J* in Hz)	No.	δ_C, type	δ_H, multi. (*J* in Hz)
1	170.8, C	–	12	37.9, CH_2	2.26, dd (15.1, 6.0); 1.61, dd (15.1, 1.8)
2	120.8, CH	5.75, d (15.1)	13	69.9, CH	3.56, td (6.0, 1.8)
3	147.3, CH	7.20, dd (15.1, 10.7)	14	52.3, CH	1.48, m
4	129.6, CH	6.18, dd (15.1, 10.7)	15	77.9, CH	3.39, qd (10.6, 5.9)
5	149.7, CH	6.07, dd (15.1, 9.1)	16	19.9, CH_3	1.15, d (5.9)
6	40.6, CH	2.44, m	17	16.0, CH_3	0.98, d (5.8)
7	74.5, CH	3.83, dd (10.1, 1.8)	18	5.4, CH_3	0.89, d (6.7)
8	37.8, CH	1.99, m	19	12.8, CH_3	0.94, d (6.8)
9	78.2, CH	3.68, dd (11.5, 5.0)	20	24.8, CH_2	1.50, m; 1.17, m
10	42.2, CH	1.59, qd (11.5, 6.8)	21	10.4, CH_3	0.91, t (7.3)
11	103.4, C	–			

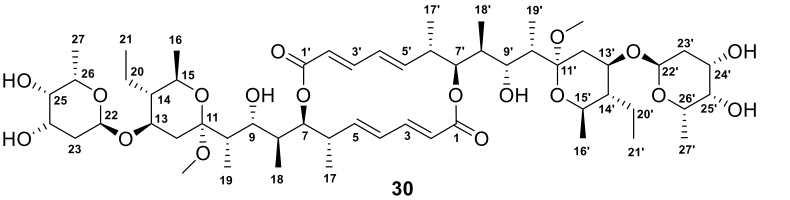

30

Table S14. ^{13}C NMR (150 MHz) and ^{1}H NMR (600 MHz) data of known compound **30** (in DMSO-d_6)

No.	δ_C, type	δ_H, multi. (J in Hz)	No.	δ_C, type	δ_H, multi. (J in Hz)
1, 1′	167.1, C	–	17,17′	15.6, CH_3	0.96, d (6.0)
2, 2′	121.3, CH	5.69, d (15.5)	18,18′	9.9, CH_3	0.83, t (7.3)
3, 3′	144.9, CH	6.81, dd (15.5, 11.1)	19,19′	7.2, CH_3	0.83, t (7.3)
4, 4′	130.4, CH	6.09, dd (15.0, 11.1)	20, 20′	18.7, CH_2	1.59, m; 1.35, m
5, 5′	145.0, CH	5.65, dd (15.0, 11.1)	21, 21′	8.8, CH_3	0.77, t (7.4)
6, 6′	41.2, CH	2.46, m	22, 22′	92.5, CH	4.90, d (4.0)
7, 7′	76.1, CH	5.12, d (10.0)	23, 23′	32.6, CH_2	1.78, td (12.0, 4.0); 1.38, dd (12.0, 5.0)
8, 8′	36.7, CH	1.70, m	24, 24′	64.9, CH	3.72, m
9, 9′	68.1, CH	3.32, dd (10.0, 7.0)	25, 25′	70.3, CH	3.37, m
10, 10′	37.5, CH	1.87, q (7.0)	26, 26′	66.3, CH	3.74, m
11, 11′	102.7, C	–	27, 27′	17.1, CH_3	1.05, d (7.5)
12, 12′	33.7, CH_2	2.37, dd (13.5, 4.5); 1.08, m	OCH_3-11, 11′	45.6, CH_3	2.94, m
13, 13′	68.2, CH	3.68, dd (11.0, 5.2)	OH-9, 9′	–	4.37, d (7.0)
14, 14′	47.1, CH	1.04, m	OH-24, 24′	–	4.52, br s
15, 15′	66.9, CH	3.40, m	OH-25, 25′	–	4.27, d (4.0)
16, 16′	18.9, CH_3	1.11, d (6.0)			

Table S15. ^{1}H NMR data of compound **8s** and **8r** (600 MHz, in MeOH-d_4, multi., J in Hz)

No.	**8s**	**8r**	$\Delta\delta_{S-R}$
3	6.85, d (1.8)	6.82, d (1.8)	+0.03
4a	2.02, d (1.8)	2.02, d (1.8)	0.00
6	4.94, m	4.96, m	–0.02
6a	1.05, d (6.3)	1.15, t (6.4)	–0.10
7	5.16, t (7.8)	5.17, dd (8.6, 6.2)	–0.01
8	4.53, dd (15.8, 7.8)	4.45, dd (15.7, 8.6)	+0.08
9	5.49, dd (15.8, 4.7)	5.46, dd (15.7, 4.4)	+0.03
10	1.50, m	1.46, m	+0.04
10a	1.43, m; 1.08, m	1.29, m; 0.89, m	+0.14; +0.19
10b	0.73, t (7.5)	0.71, t (7.5)	+0.02
11	1.29, m; 1.13, m	1.07, m; 0.99, m	+0.22; +0.14
12	2.84, dd (18.3, 6.3); 2.12, dd (18.3, 12.3)	2.80, dd (18.7, 5.8); 2.11, dd (18.7, 12.7)	+0.04; +0/01
16a	1.97, s	2.02, s	–0.05
17	7.78, s	7.79, s	–0.01
21	5.96, s	5.94, s	+0.02

Figure S3. ^{1}H NMR spectrum of hygrocin K (**5**)

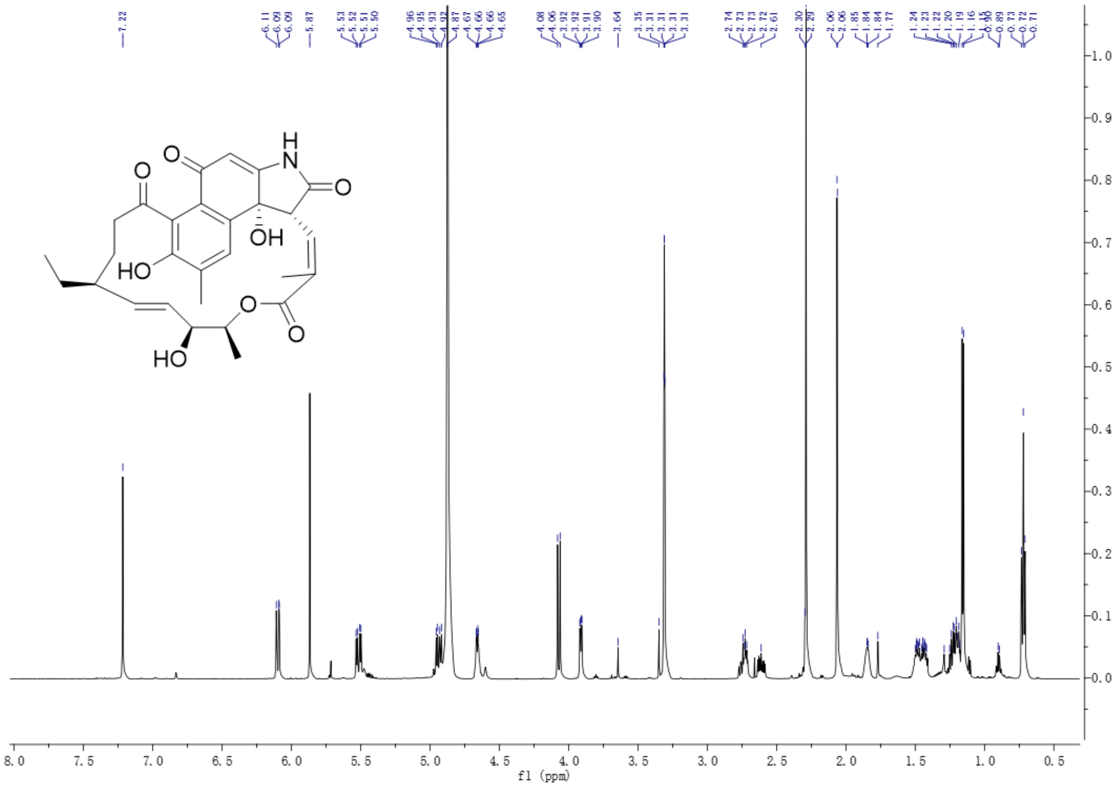

Figure S4. ^{1}H NMR spectrum of hygrocin K (**5**)

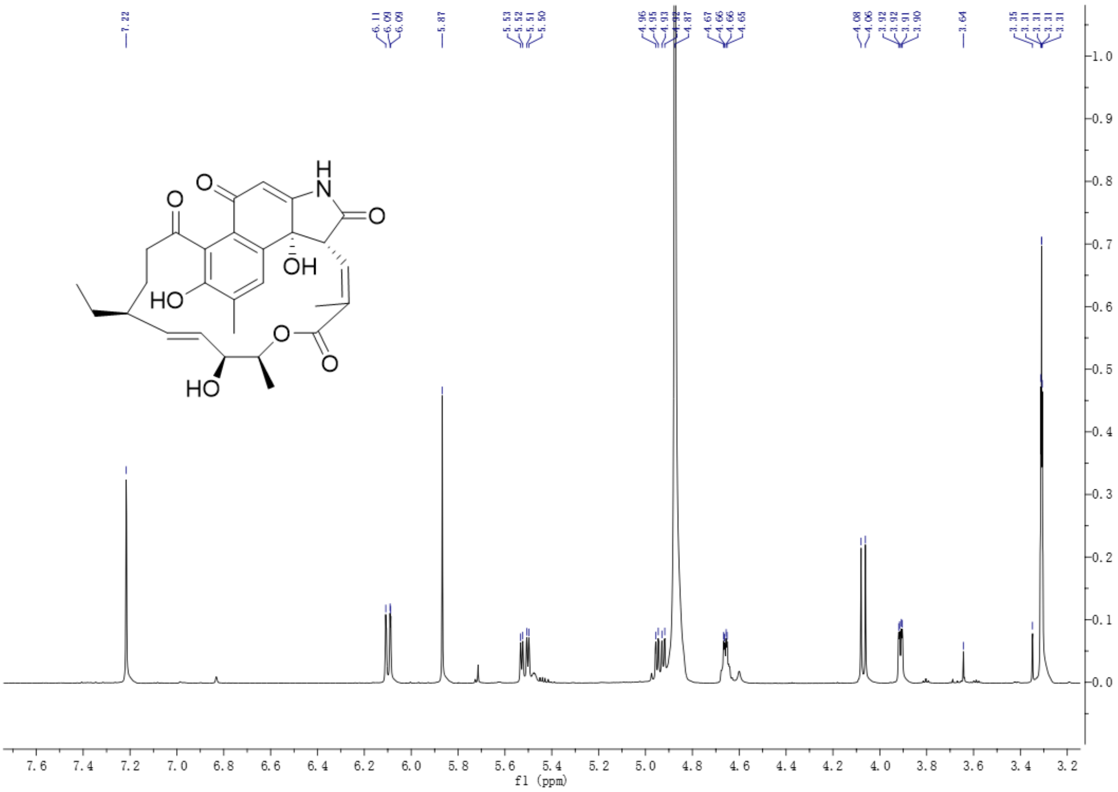

Figure S5. ^{1}H NMR spectrum of hygrocin K (**5**)

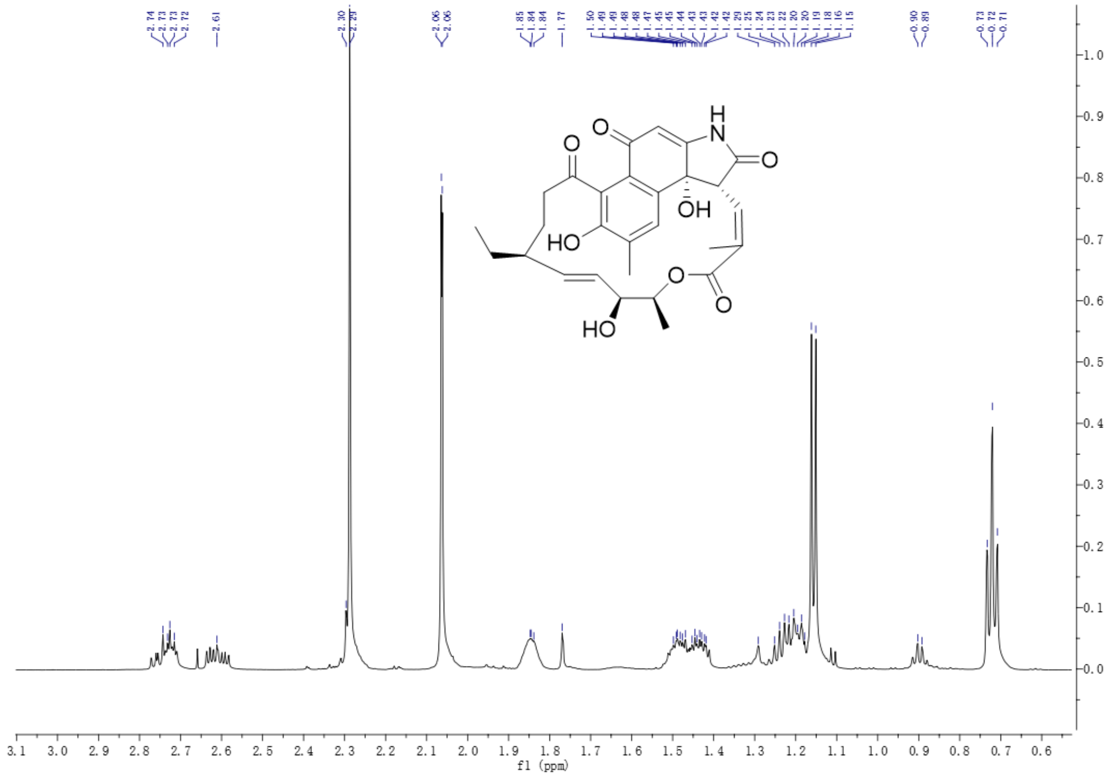

Figure S6. ^{13}C NMR spectrum of hygrocin K (**5**)

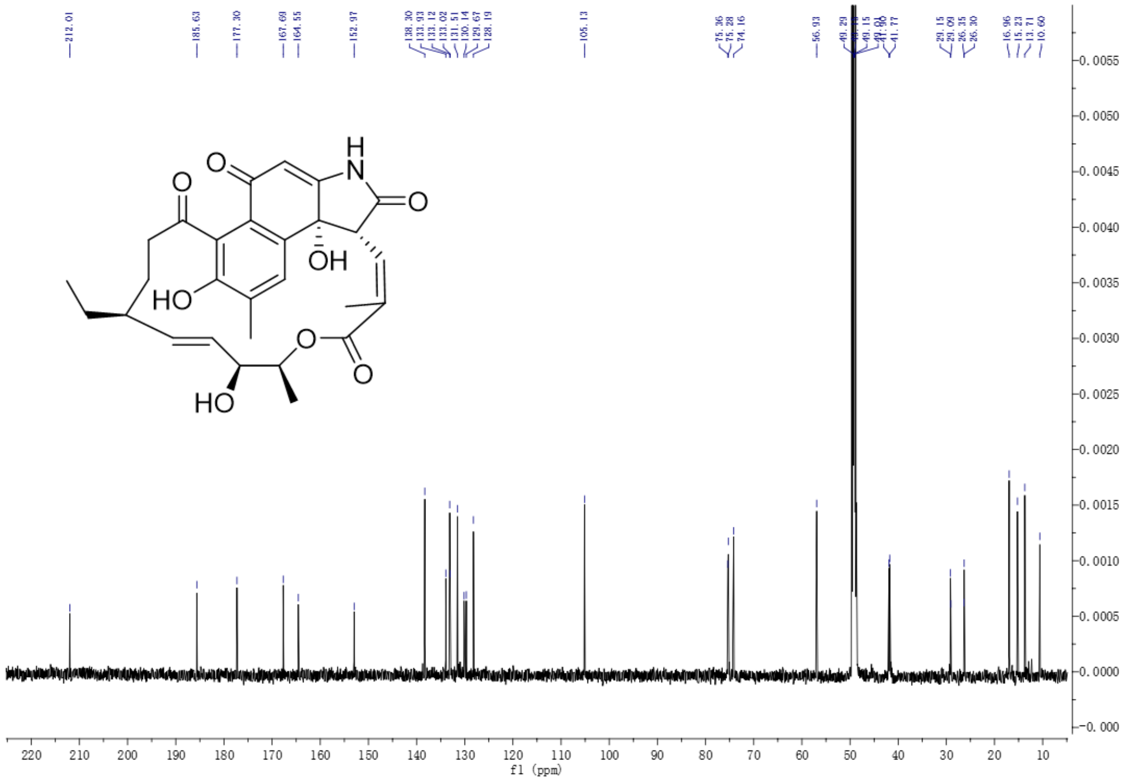

Figure S7. ^{13}C NMR spectrum of hygrocin K (**5**)

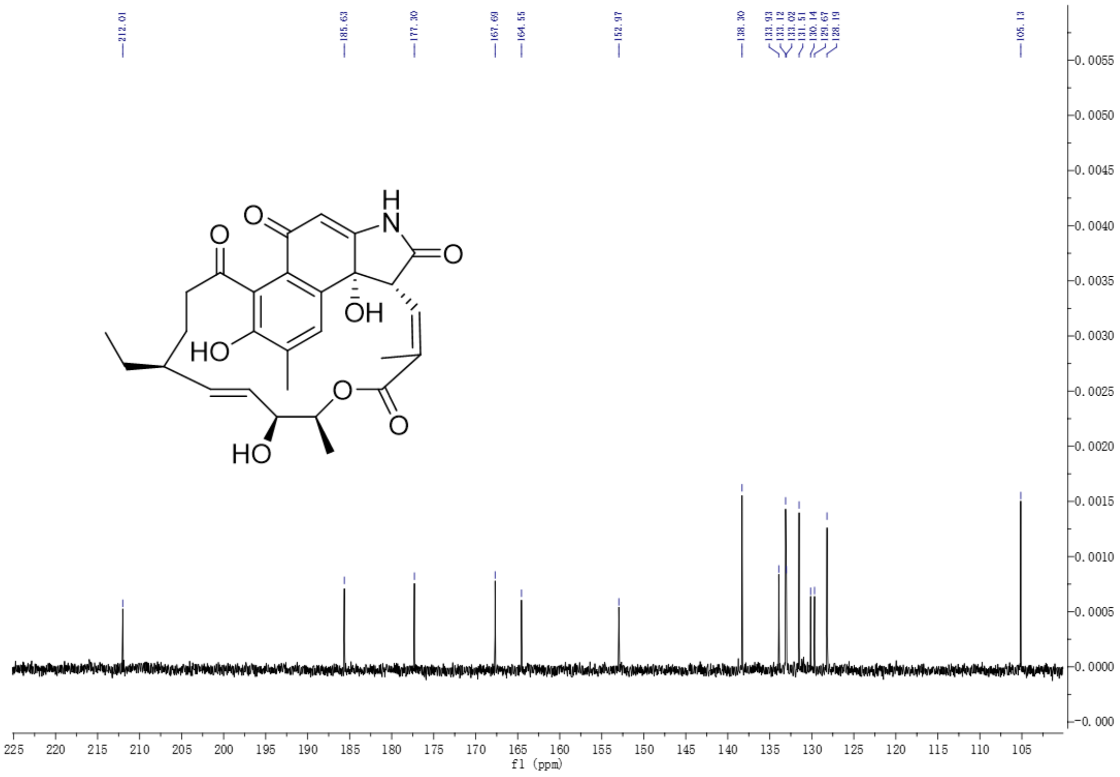

Figure S8. ^{13}C NMR spectrum of hygrocin K (**5**)

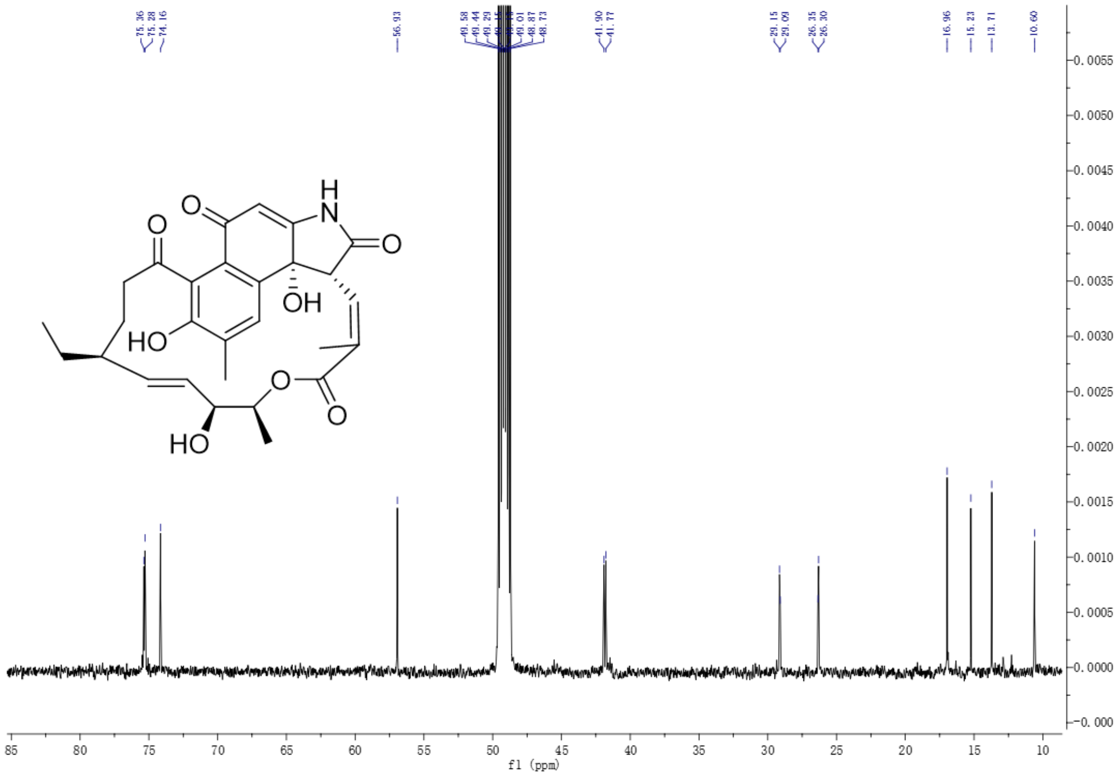

Figure S9. HMQC spectrum of hygrocin K (**5**)

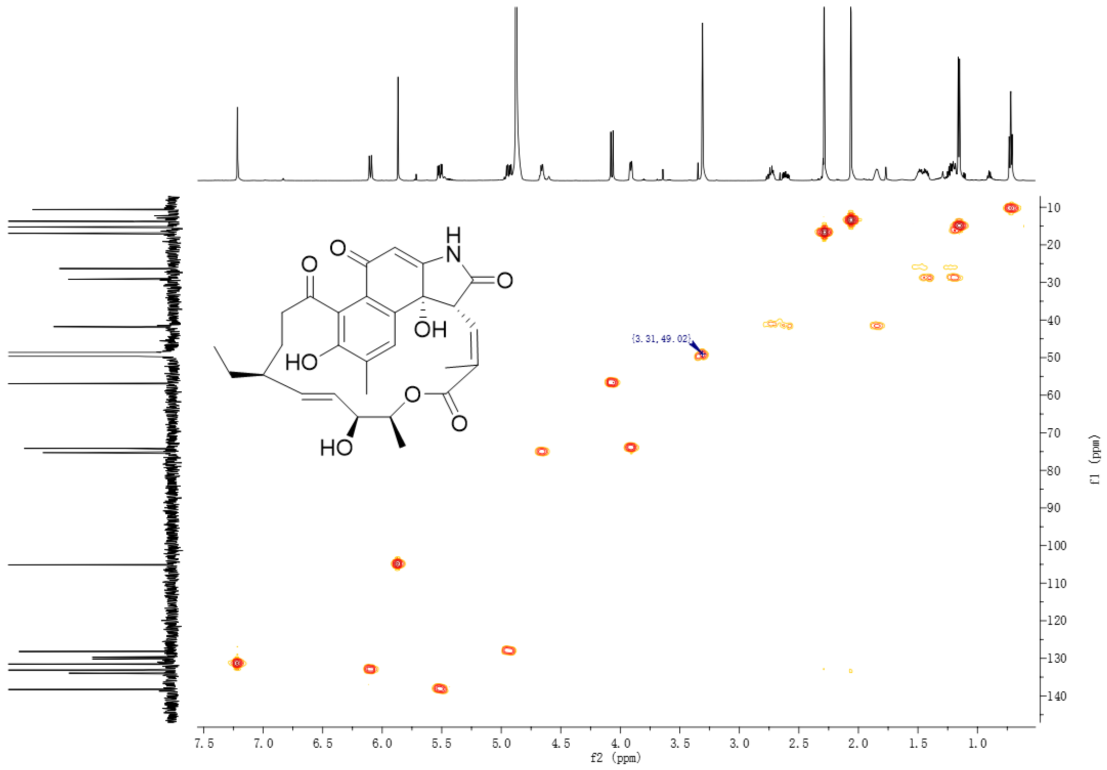

Figure S10. HMQC spectrum of hygrocin K (**5**)

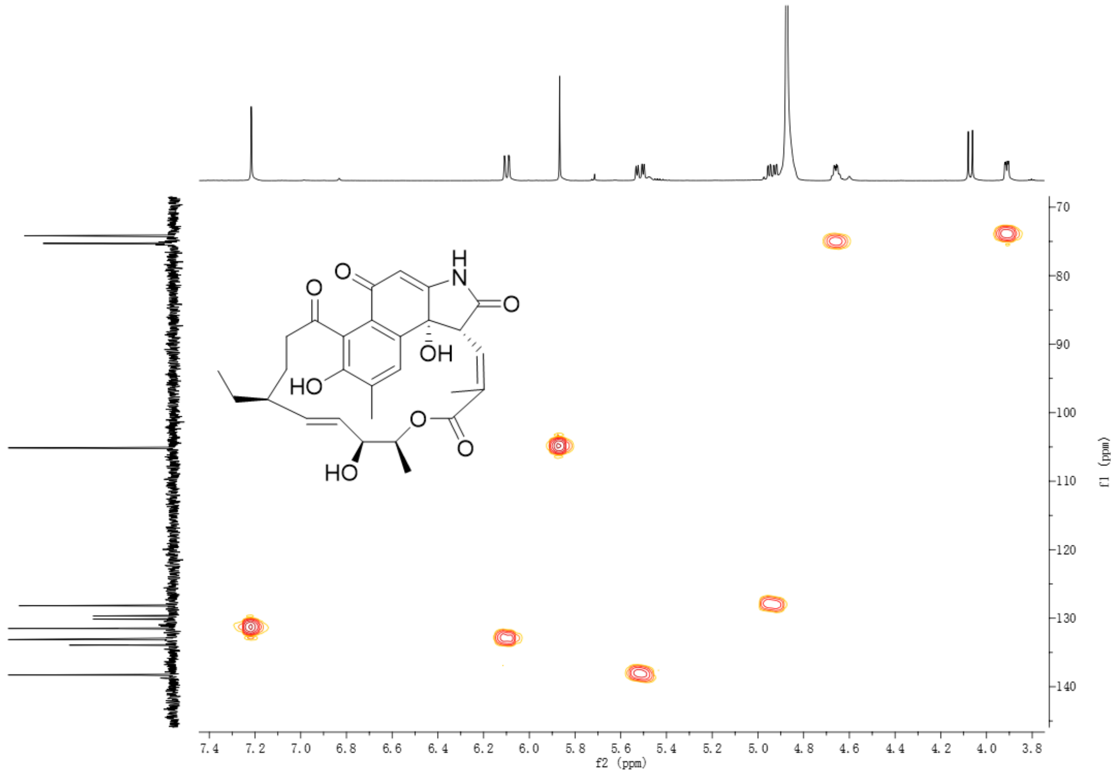

Figure S11. HMQC spectrum of hygrocin K (**5**)

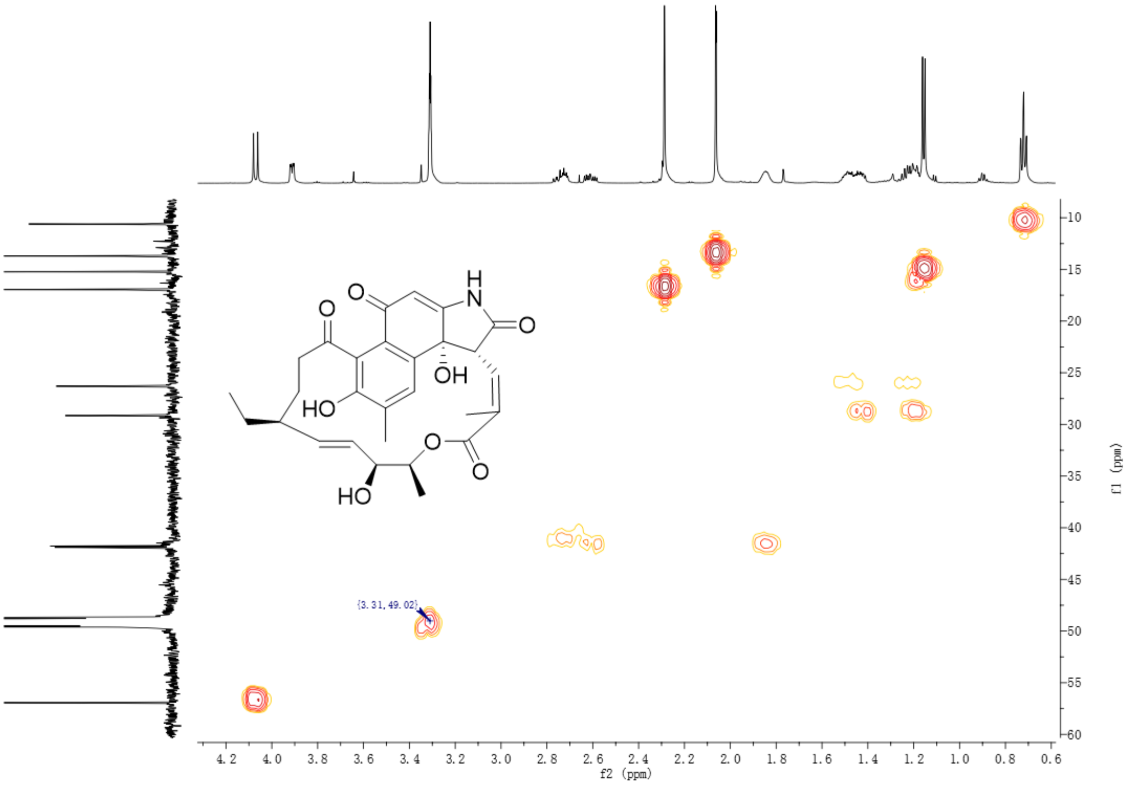

Figure S12. COSY spectrum of hygrocin K (**5**)

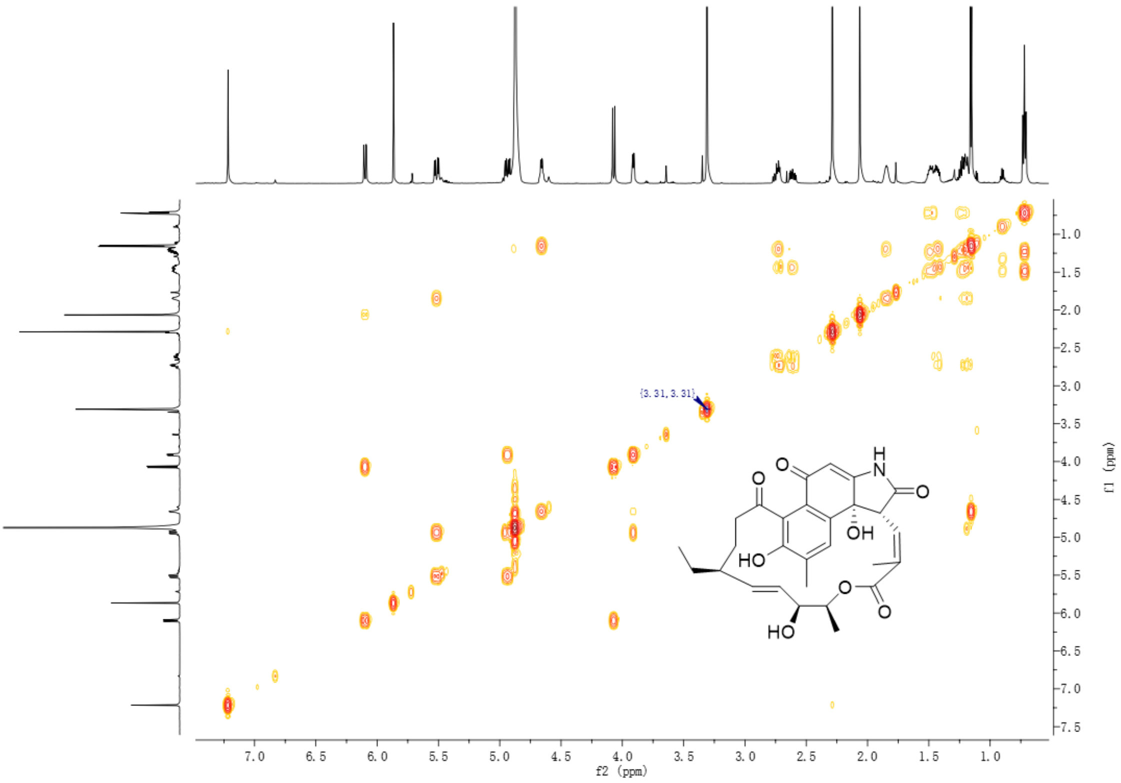

Figure S13. HMBC spectrum of hygrocin K (**5**)

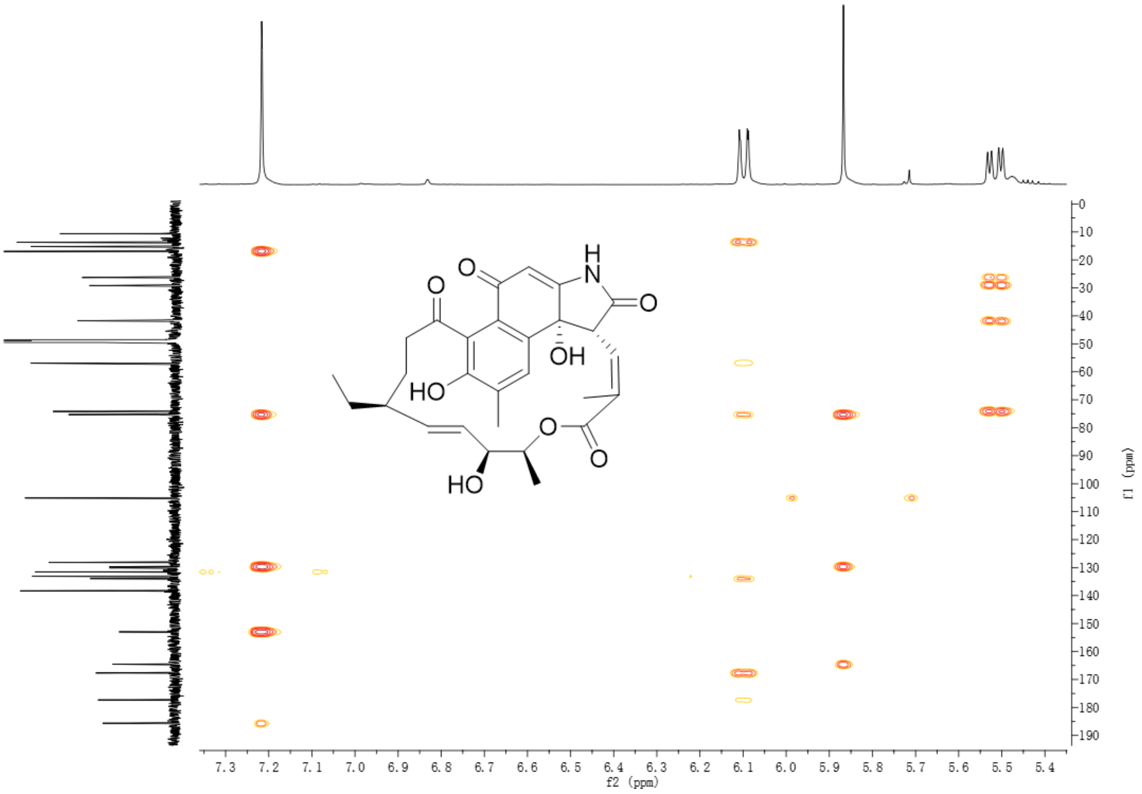

Figure S14. HMBC spectrum of hygrocin K (**5**)

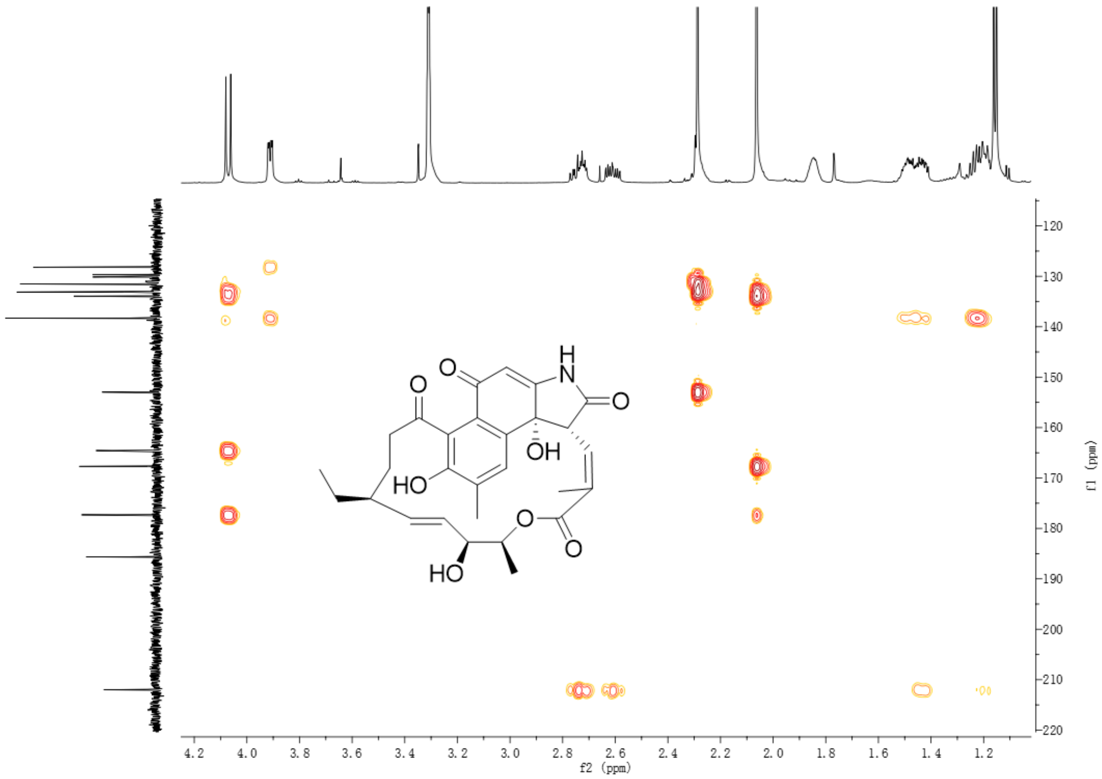

Figure S15. HMBC spectrum of hygrocin K (**5**)

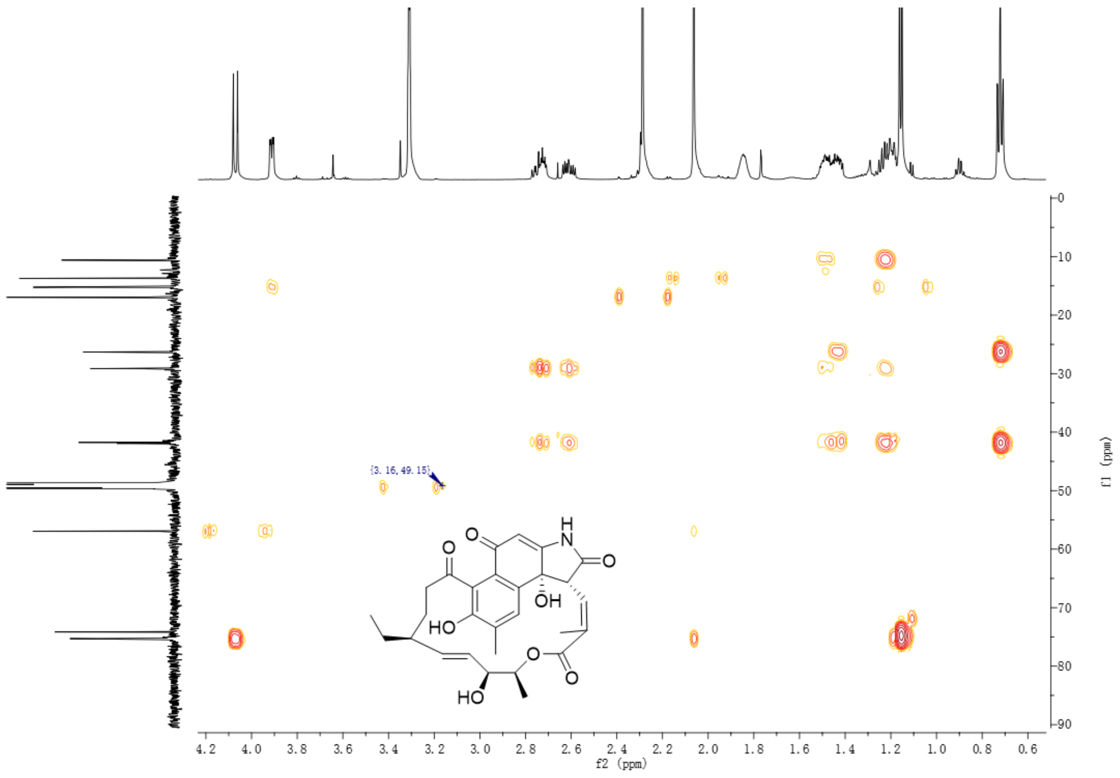

Figure S16. NOESY spectrum of hygrocin K (**5**)

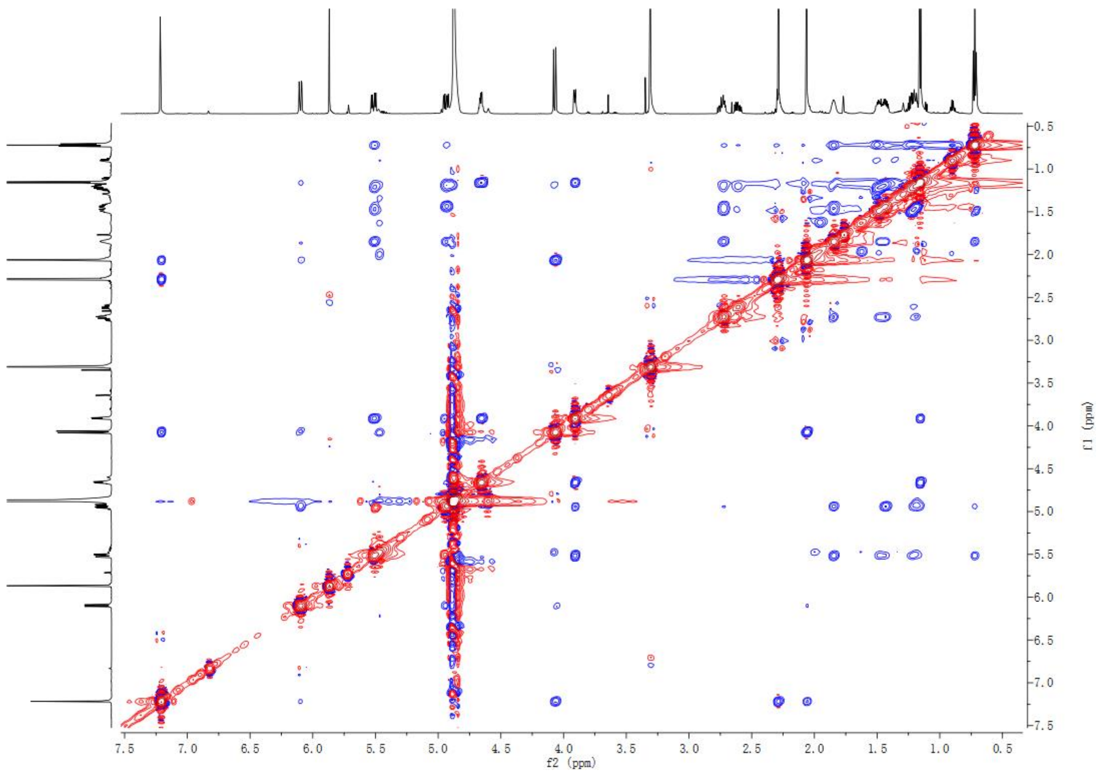

Figure S17. HRESIMS spectrum of hygrocin K (**5**)

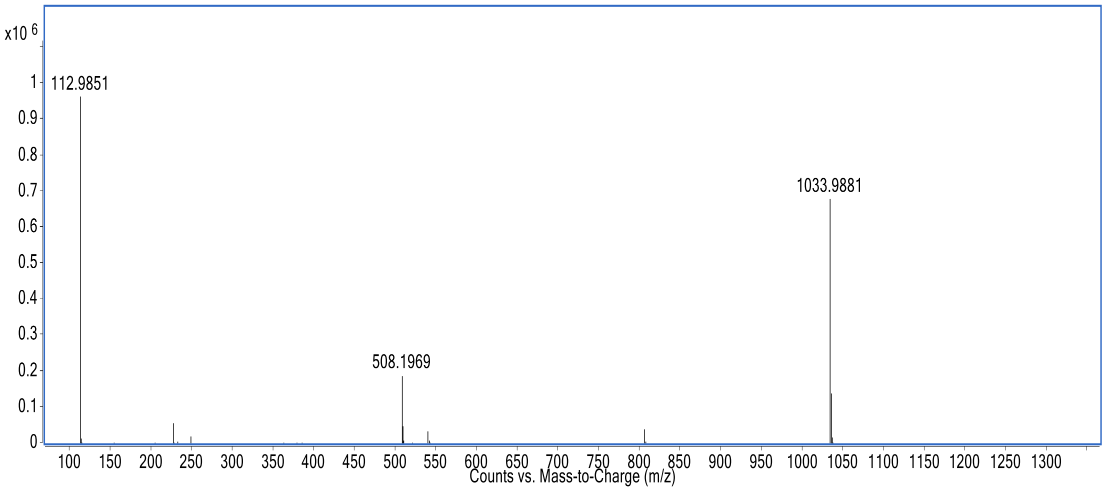

Figure S18. UV spectrum of hygrocin K (**5**)

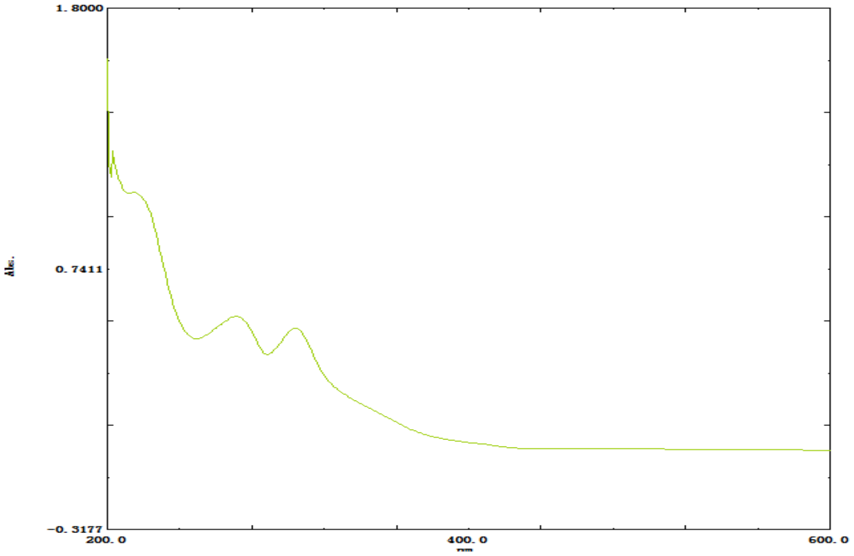

Figure S19. IR spectrum of hygrocin K (**5**)

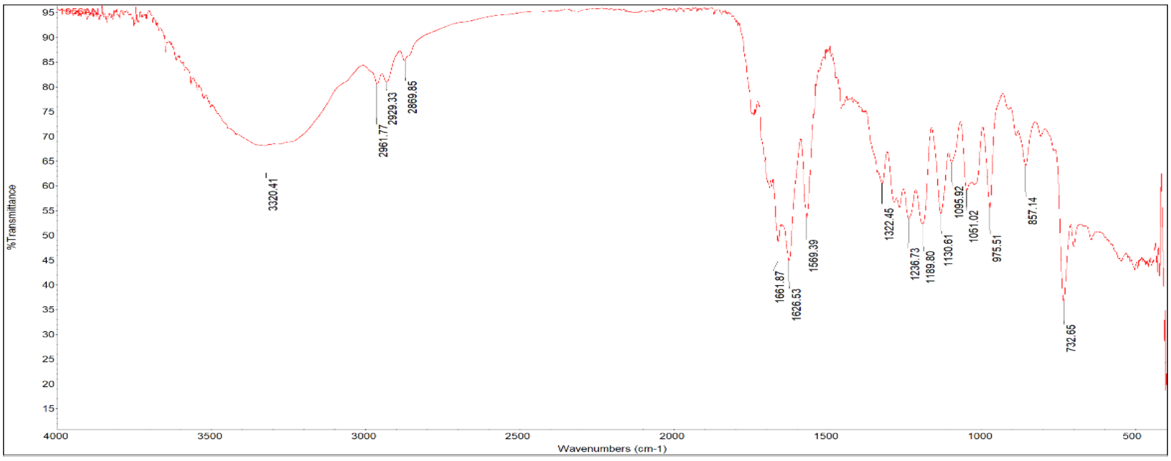

Figure S20. ^{1}H NMR spectrum of hygrocin L (**6**)

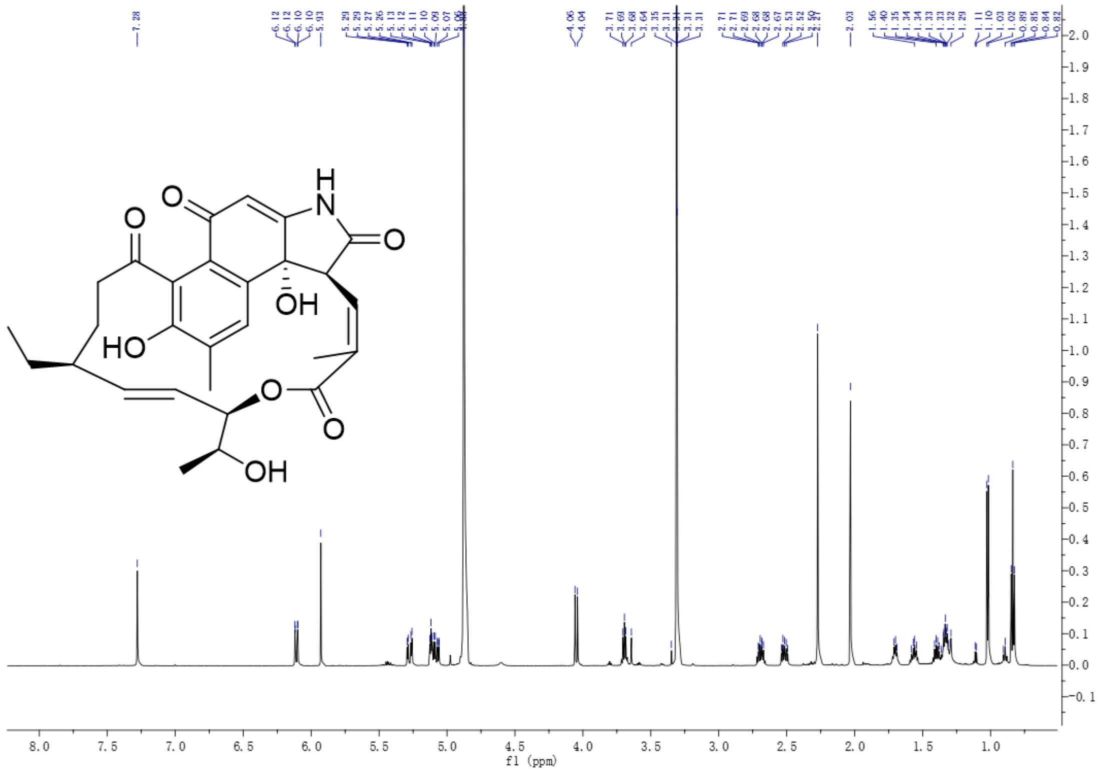

Figure S21. ^{1}H NMR spectrum of hygrocin L (**6**)

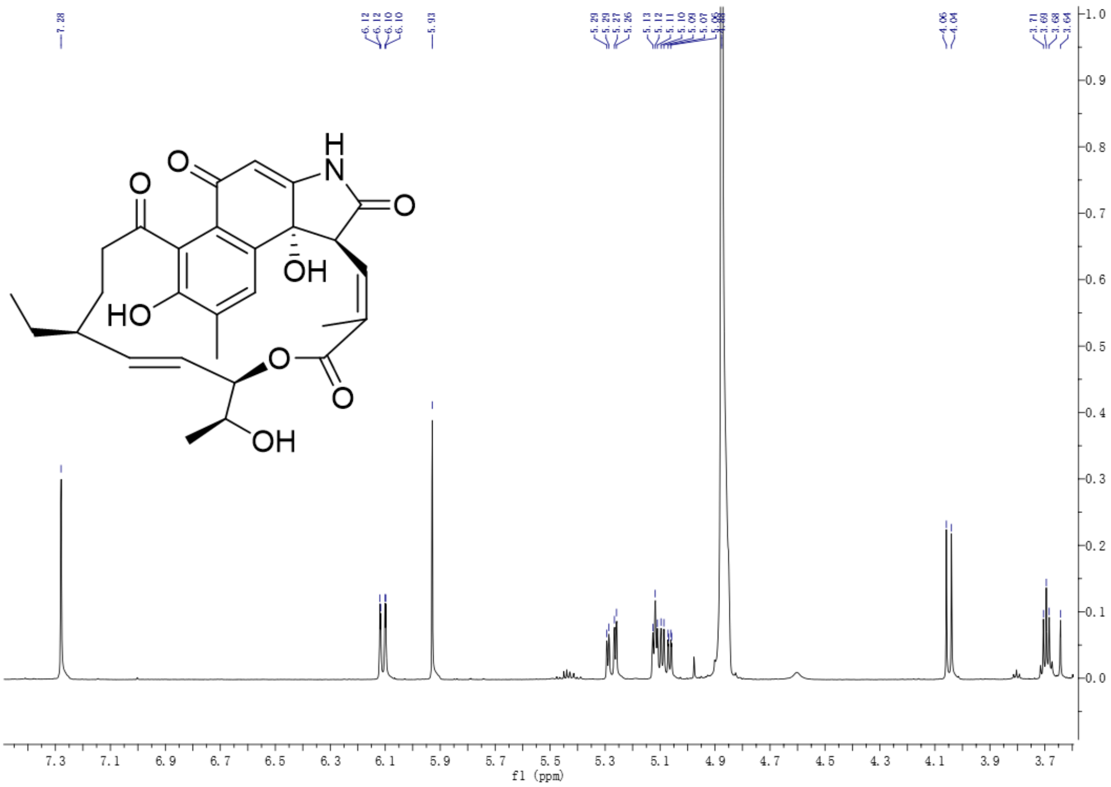

Figure S22. ^{1}H NMR spectrum of hygrocin L (**6**)

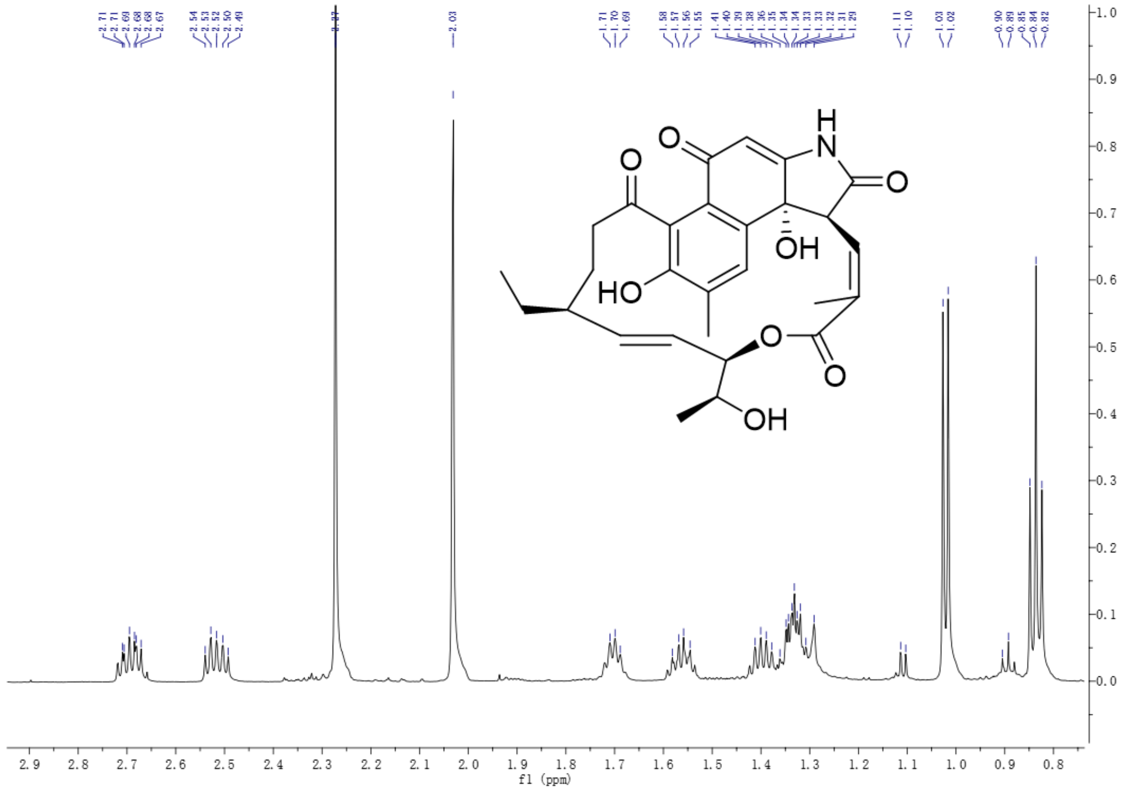

Figure S23. ^{13}C NMR spectrum of hygrocin L (**6**)

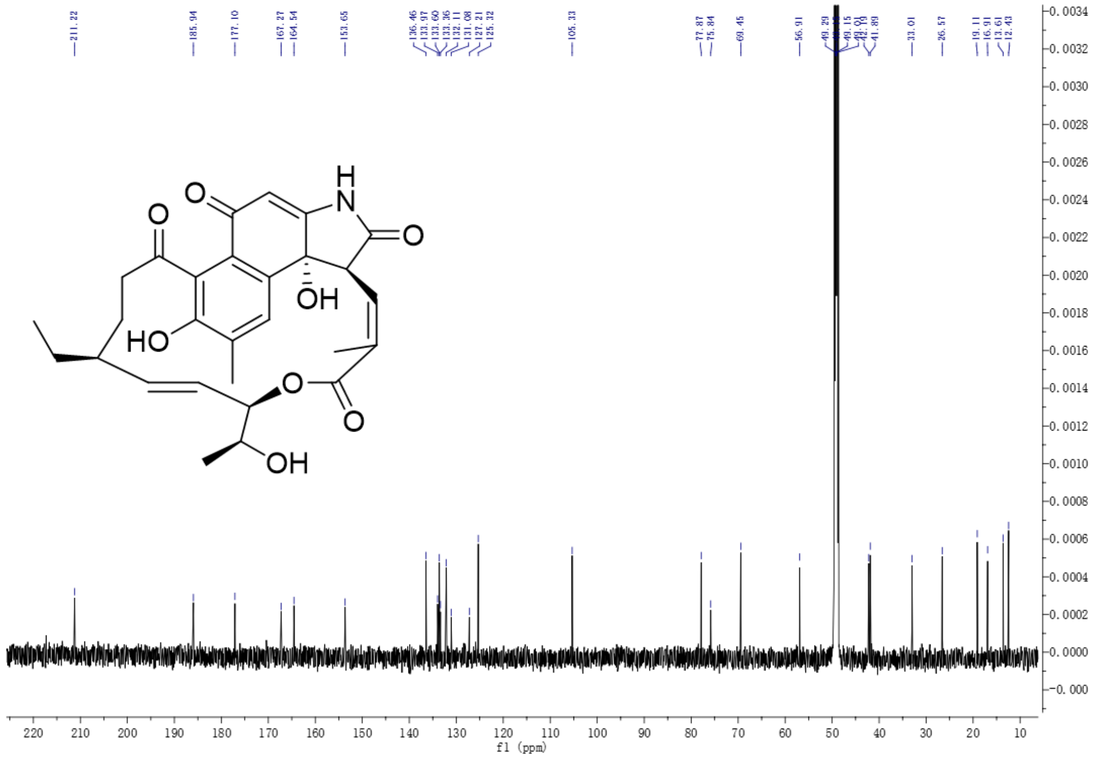

Figure S24. ^{13}C NMR spectrum of hygrocin L (**6**)

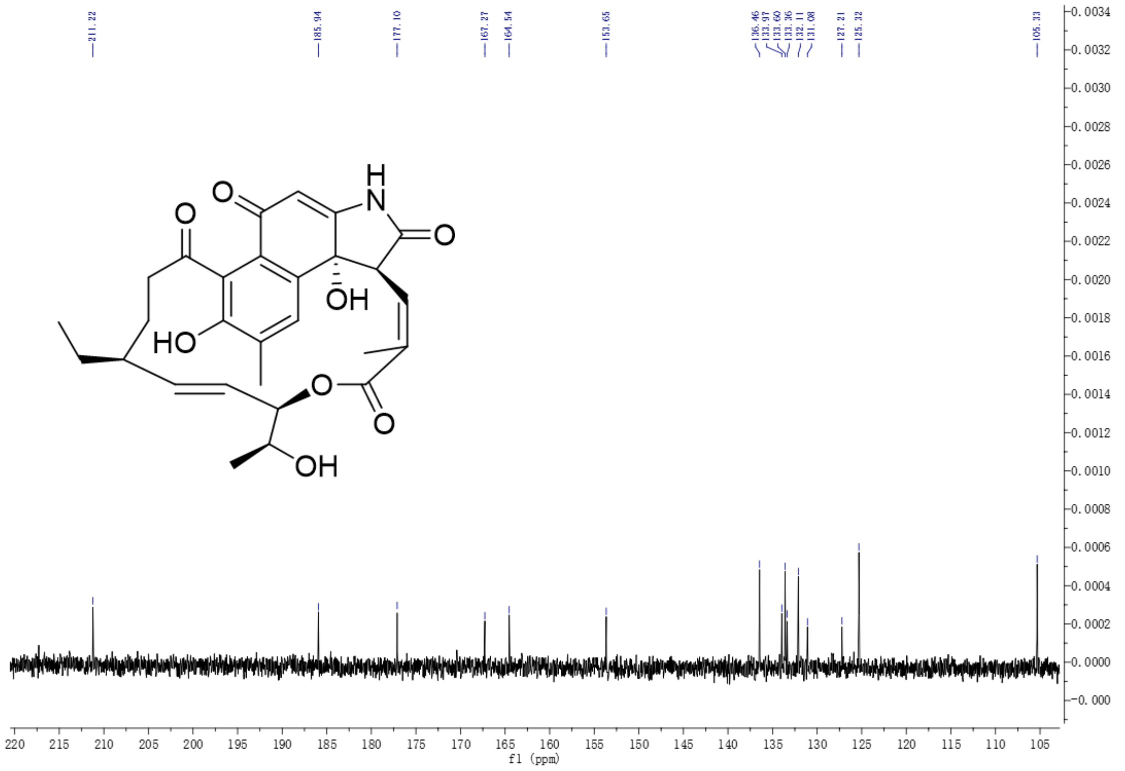

Figure S25. ^{13}C NMR spectrum of hygrocin L (**6**)

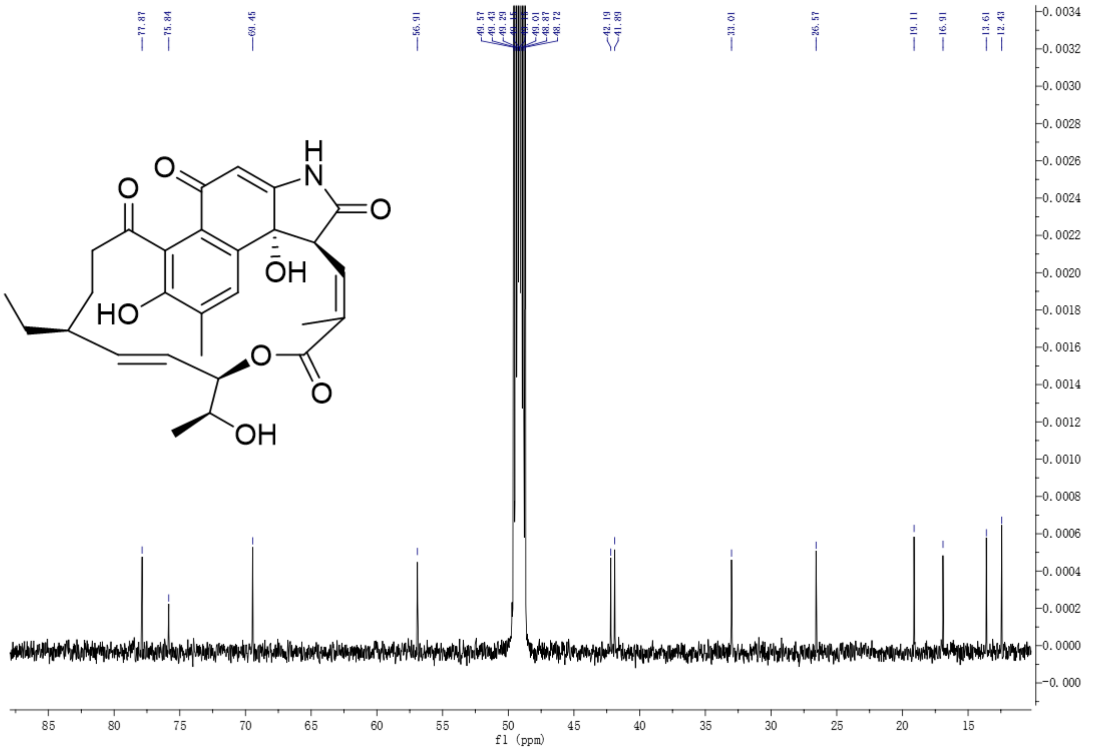

Figure S26. HMQC spectrum of hygrocin L (**6**)

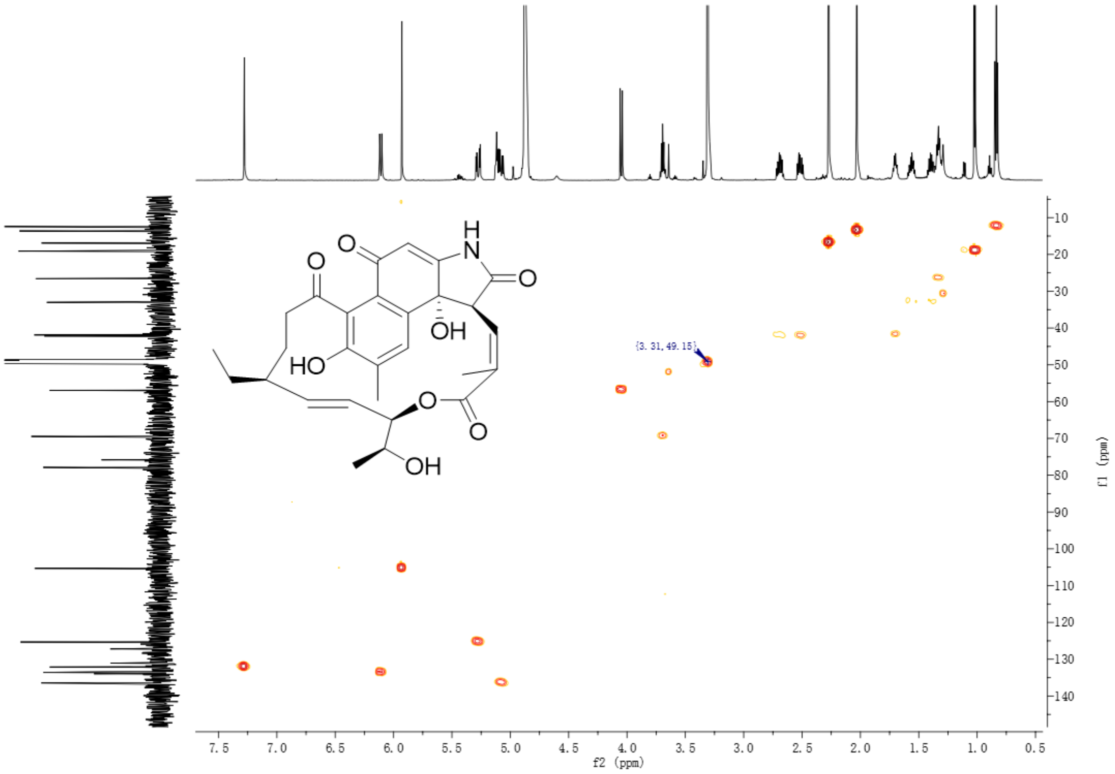

Figure S27. HMQC spectrum of hygrocin L (**6**)

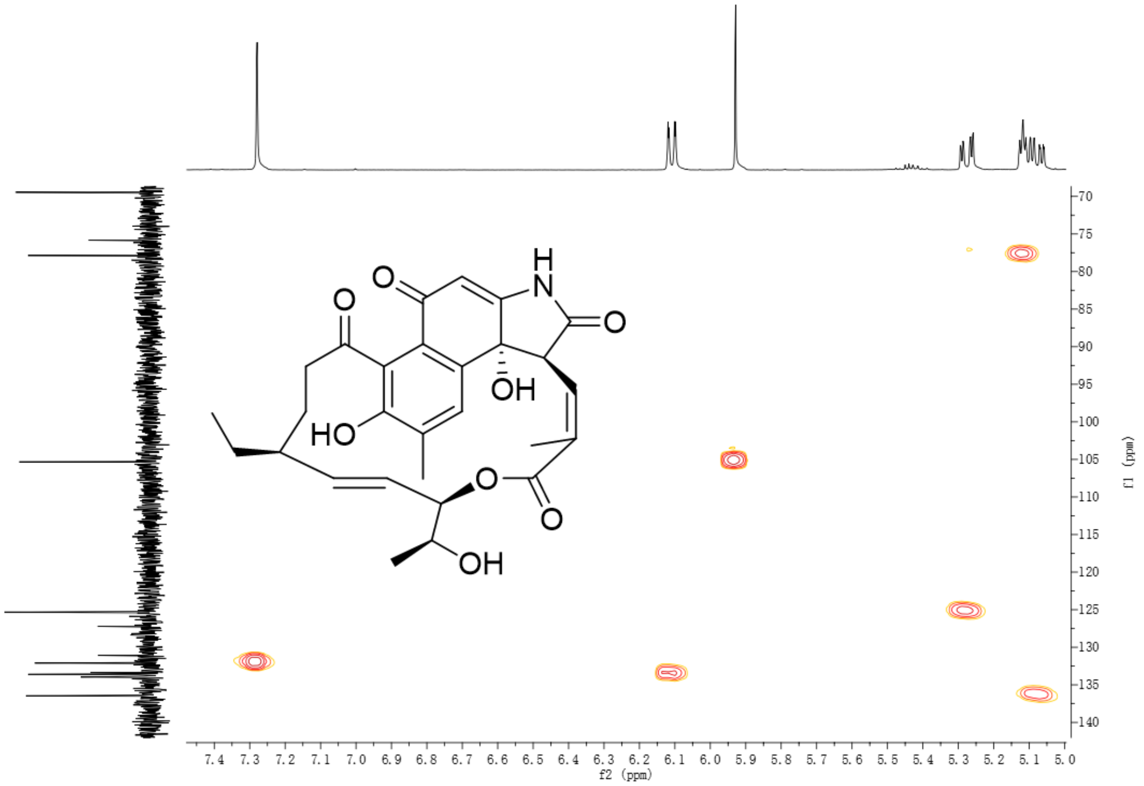

Figure S28. HMQC spectrum of hygrocin L (**6**)

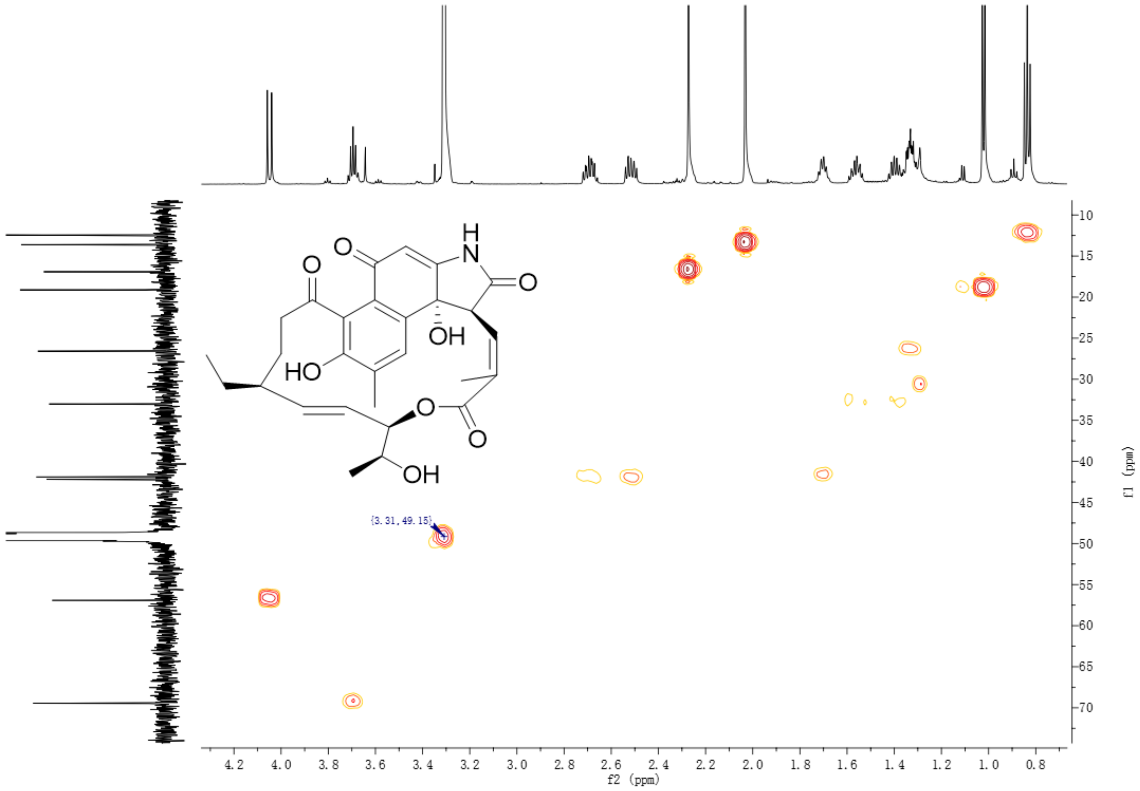

Figure S29. COSY spectrum of hygrocin L (**6**)

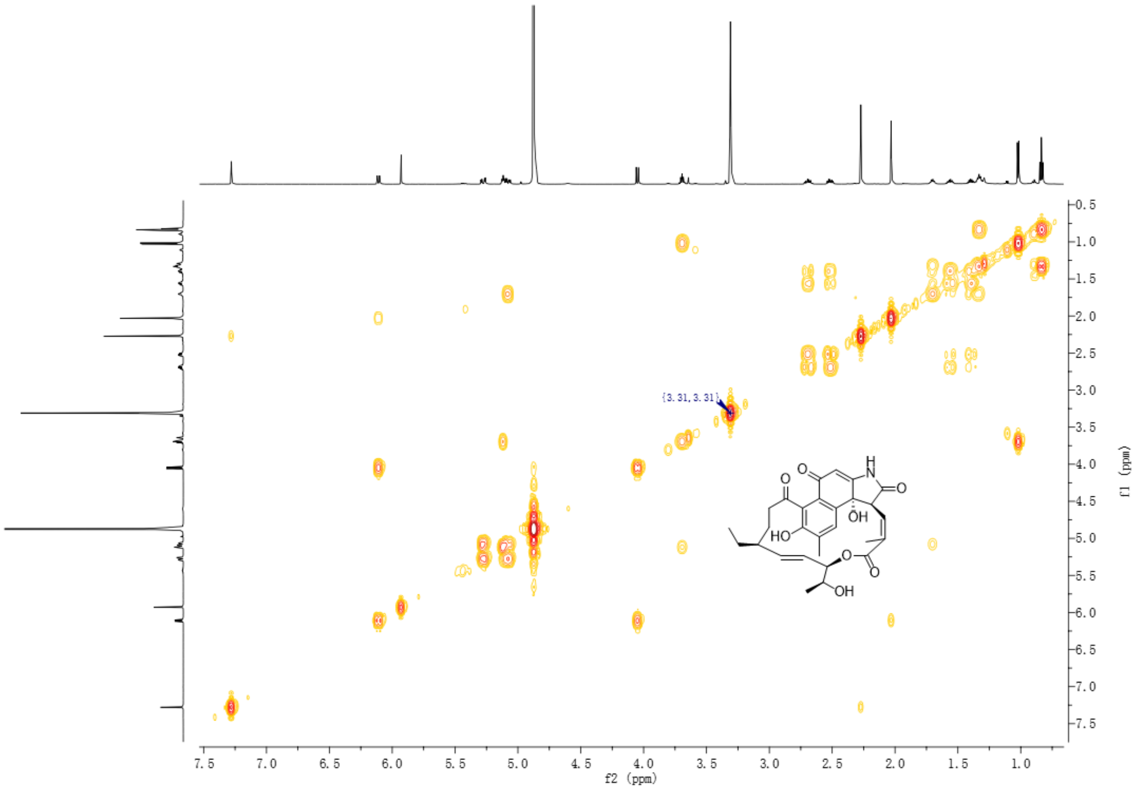

Figure S30. HMBC spectrum of hygrocin L (**6**)

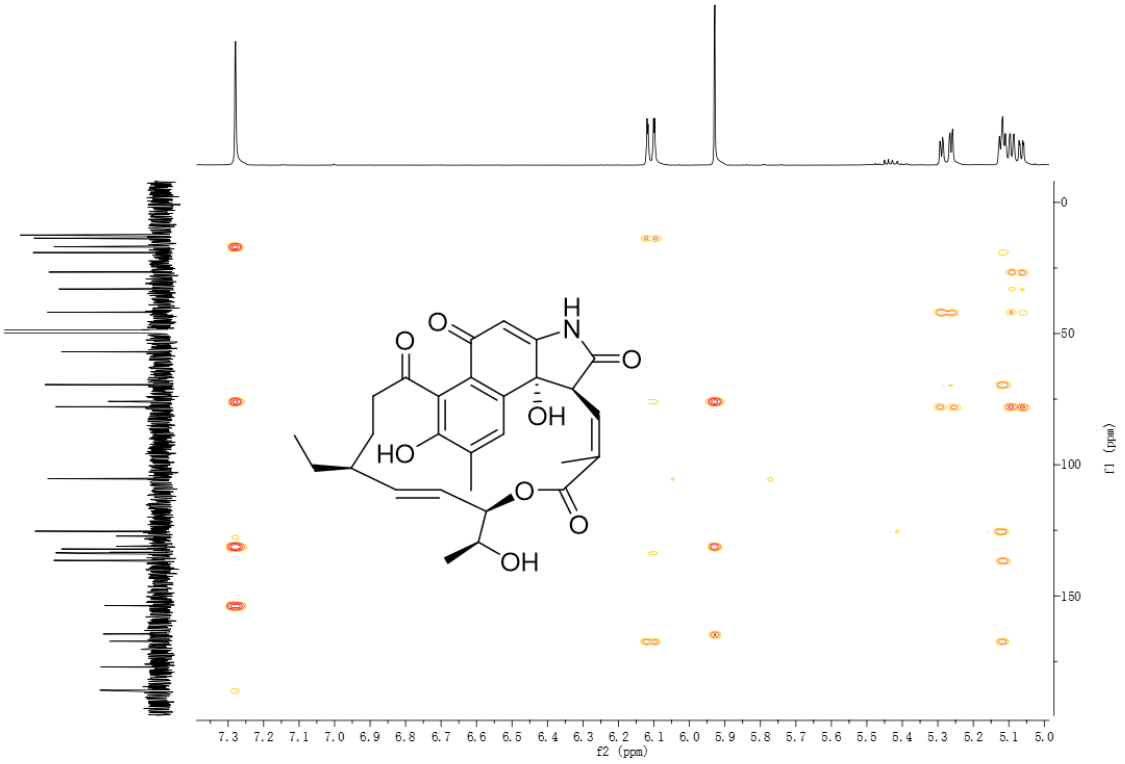

Figure S31. HMBC spectrum of hygrocin L (**6**)

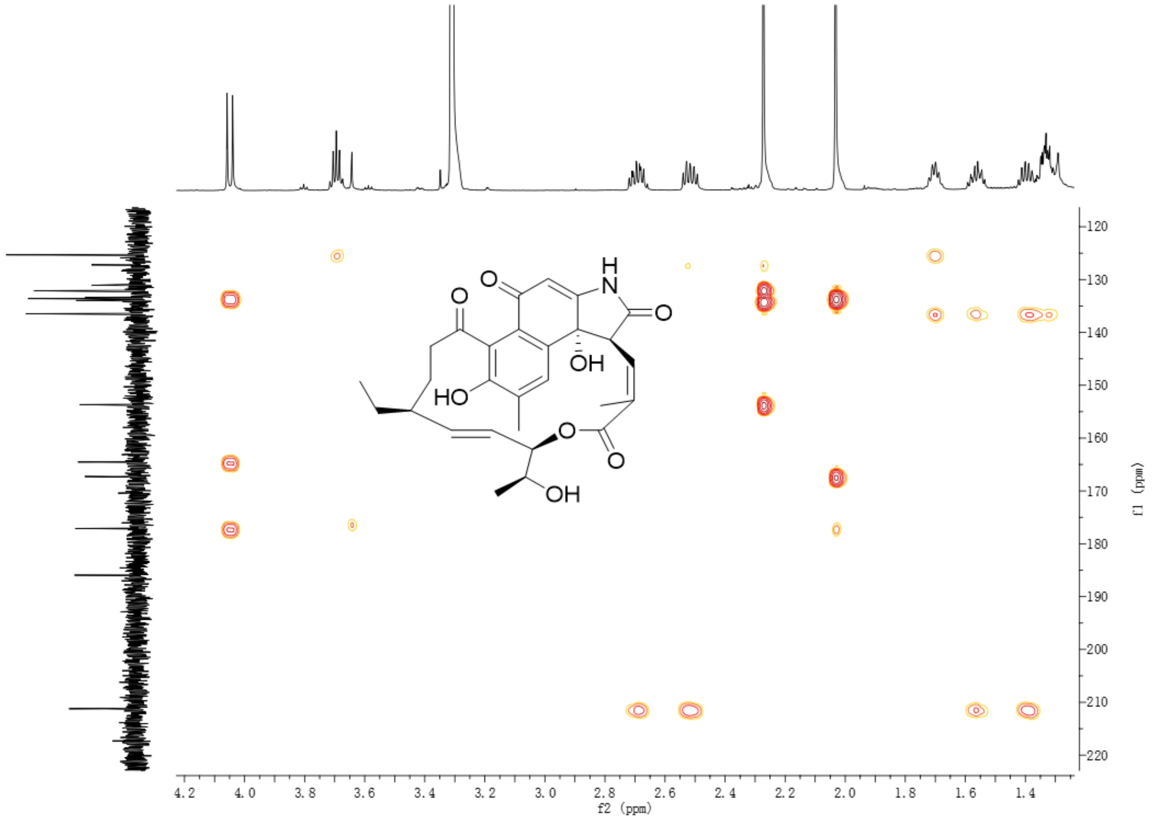

Figure S32. HMBC spectrum of hygrocin L (**6**)

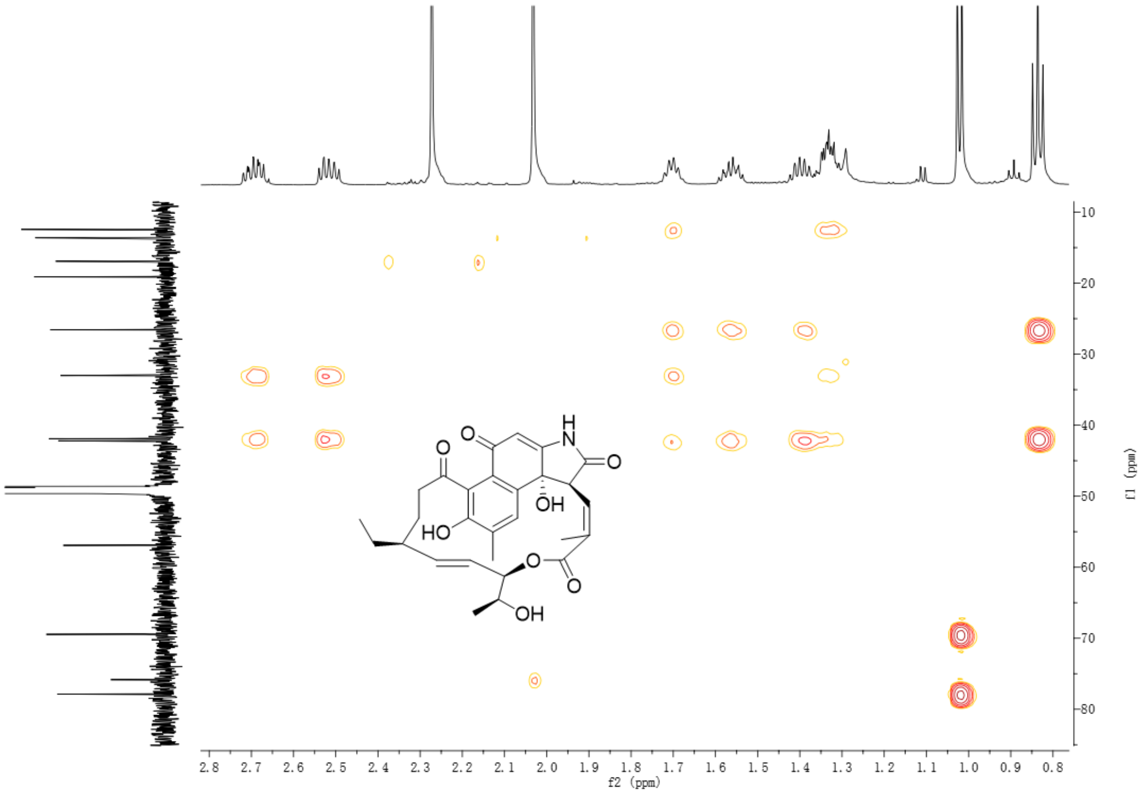

Figure S33. NOESY spectrum of hygrocin L (**6**)

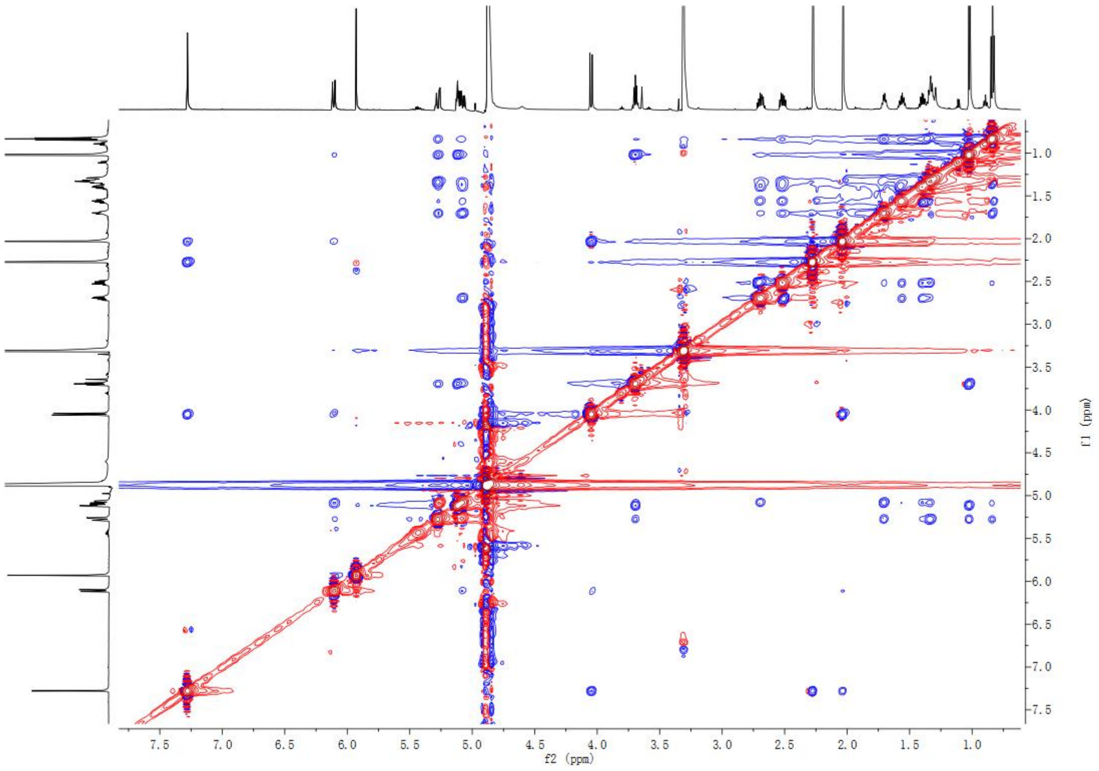

Figure S34. HRESIMS spectrum of hygrocin L (**6**)

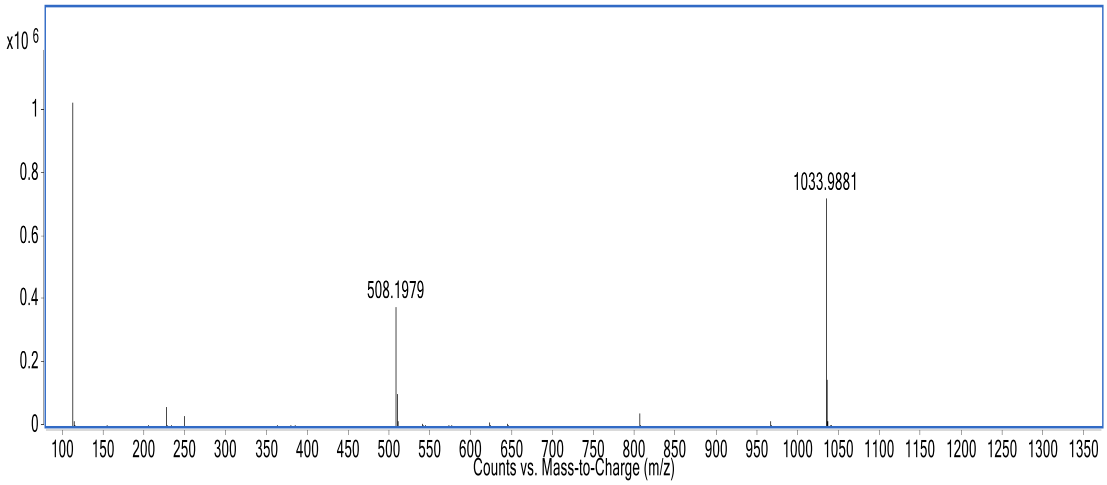

Figure S35. UV spectrum of hygrocin L (**6**)

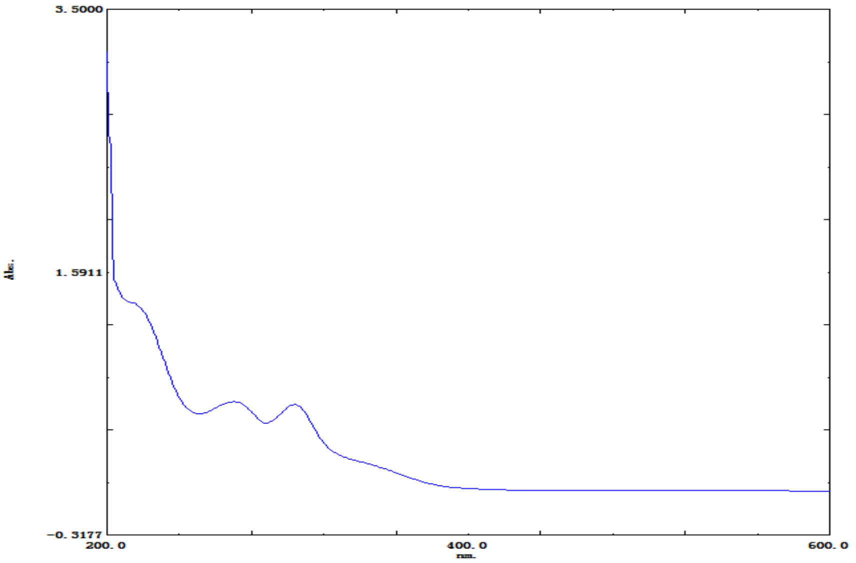

Figure S36. IR spectrum of hygrocin L (**6**)

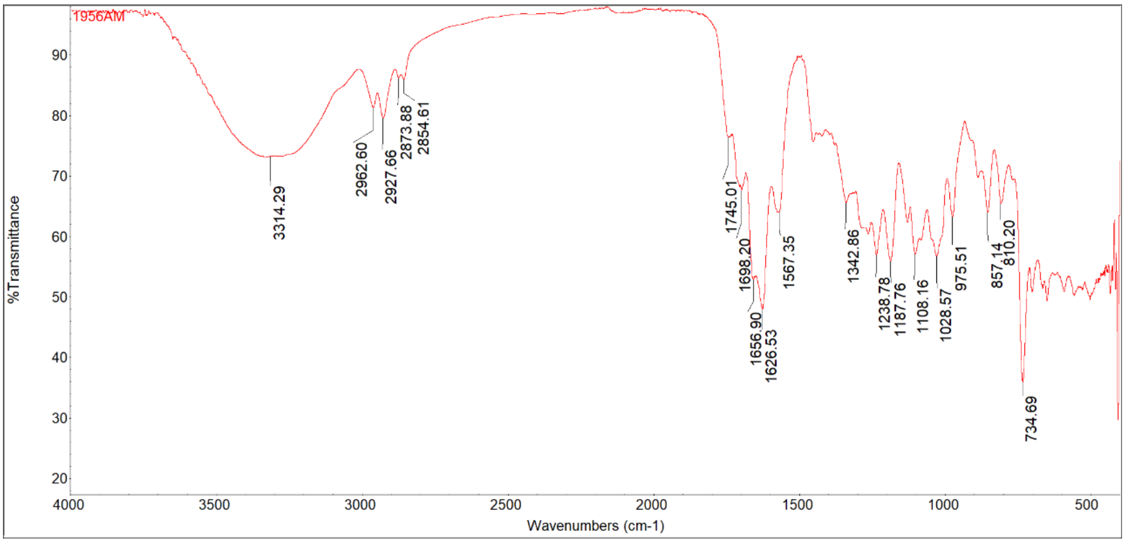

Figure S37. ^{1}H NMR spectrum of hygrocin M (**7**)

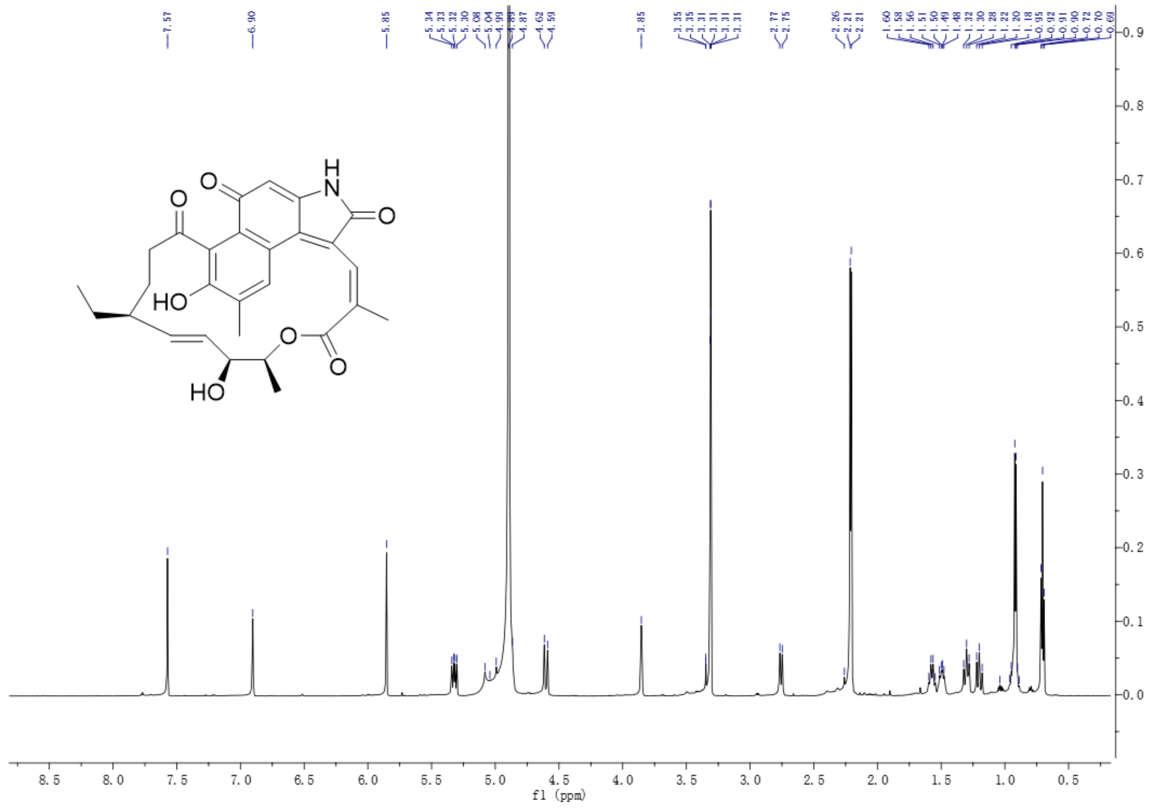

Figure S38. ^{1}H NMR spectrum of hygrocin M (**7**)

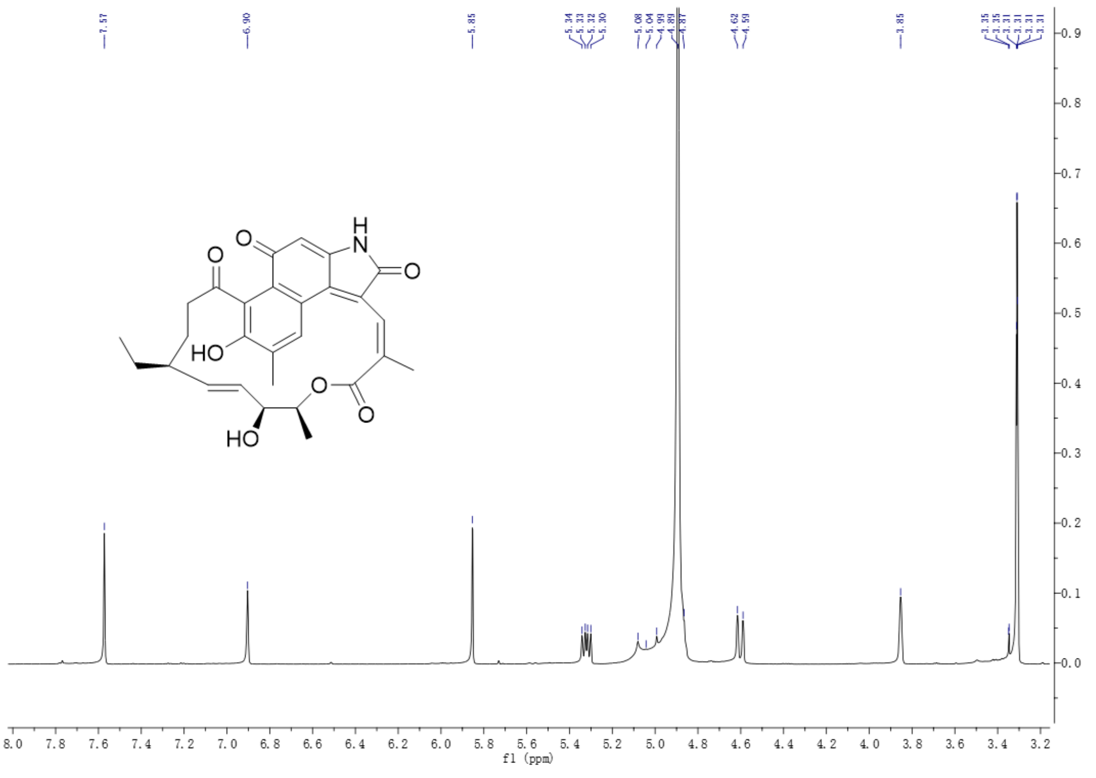

Figure S39. ^{1}H NMR spectrum of hygrocin M (**7**)

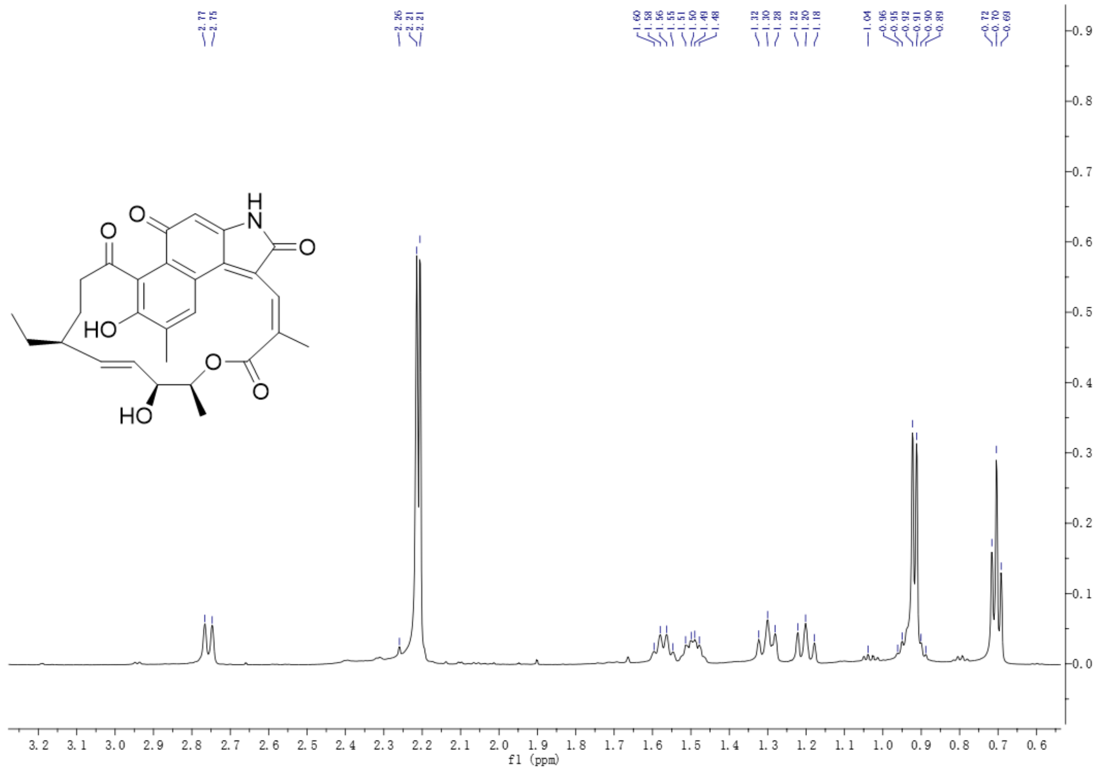

Figure S40. ^{13}C NMR spectrum of hygrocin M (**7**)

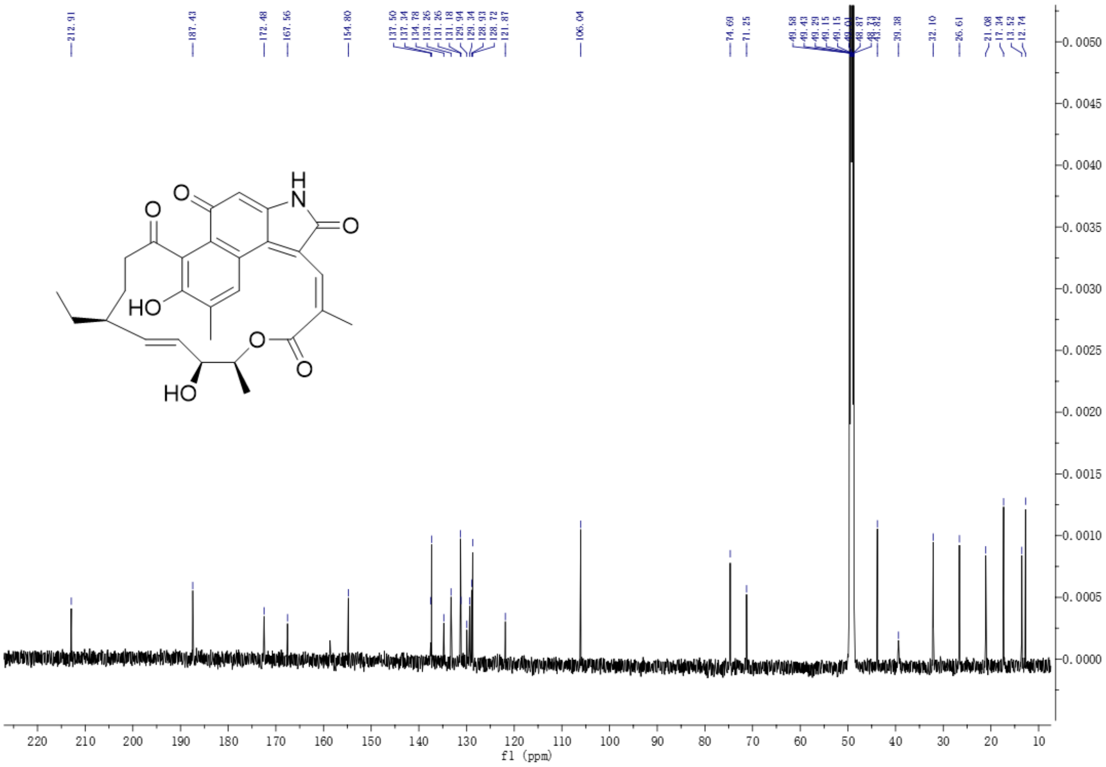

Figure S41. ^{13}C NMR spectrum of hygrocin M (**7**)

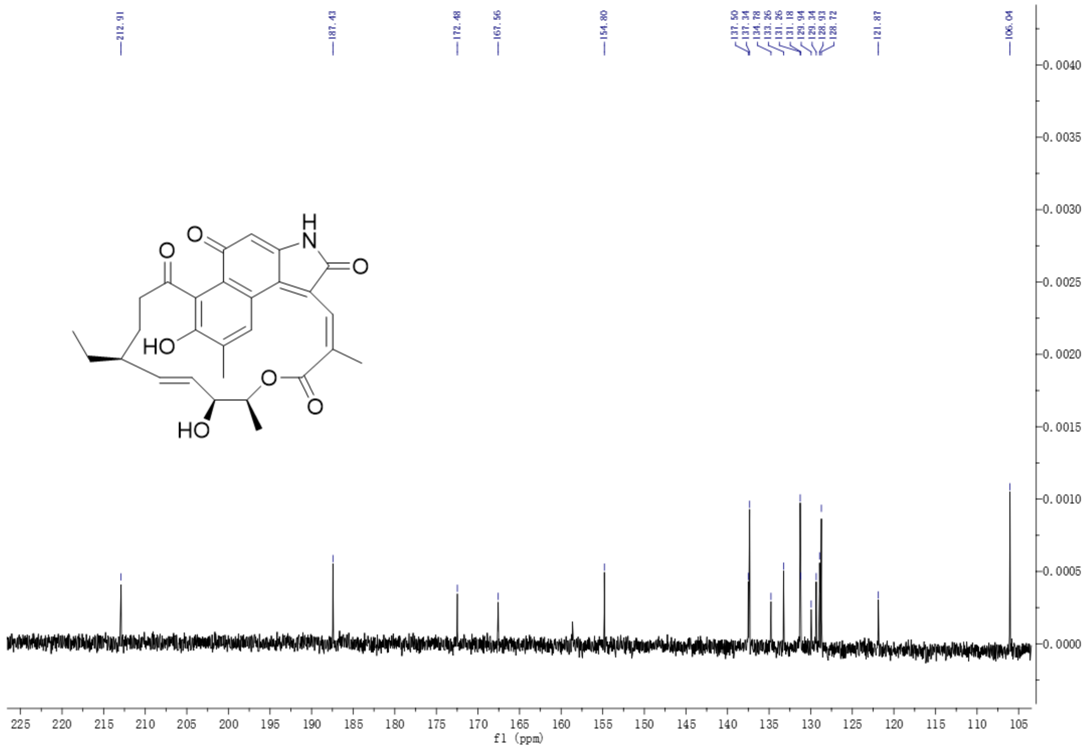

Figure S42. ^{13}C NMR spectrum of hygrocin M (**7**)

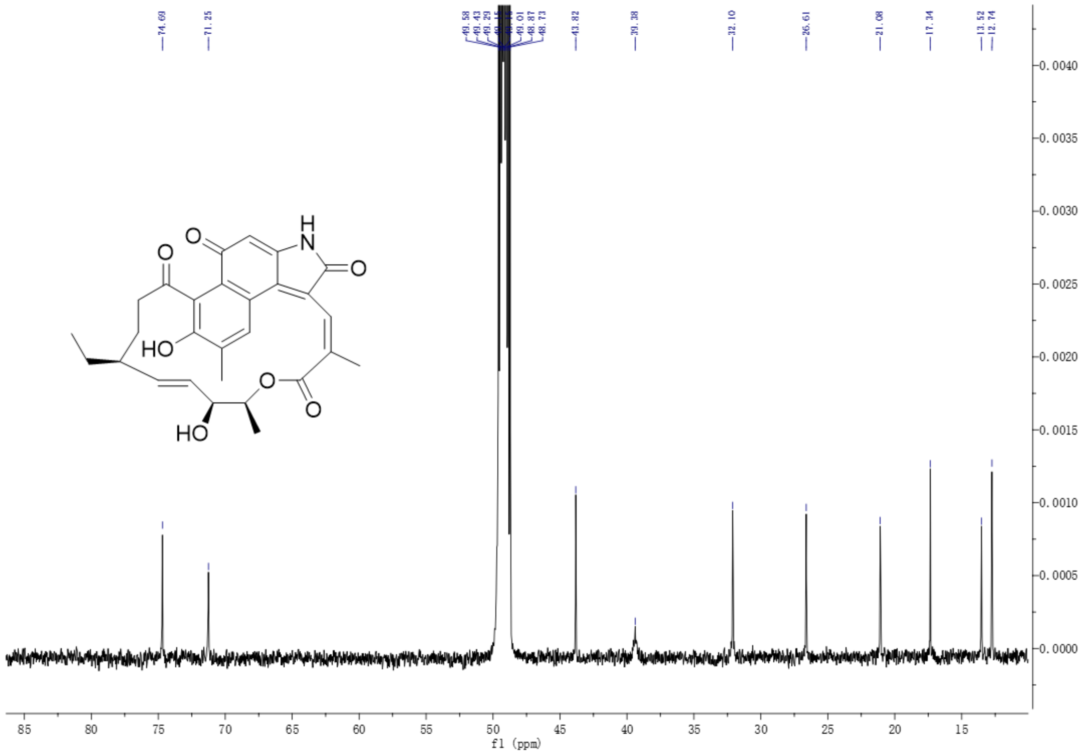

Figure S43. HMQC spectrum of hygrocin M (**7**)

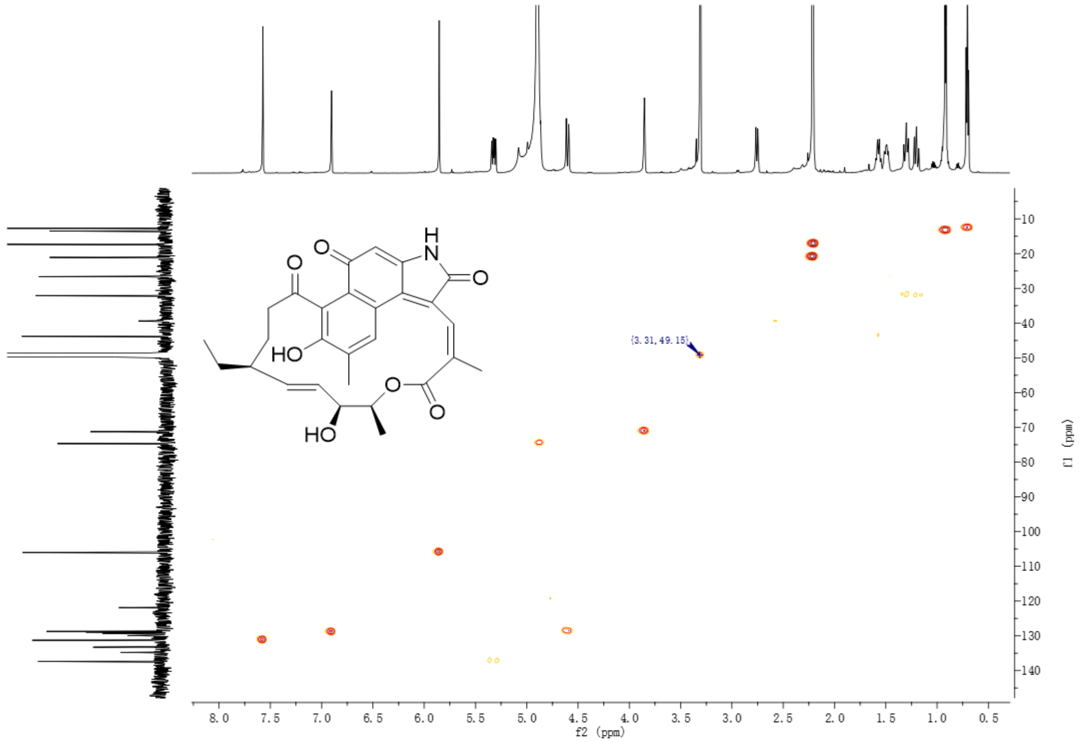

Figure S44. HMQC spectrum of hygrocin M (**7**)

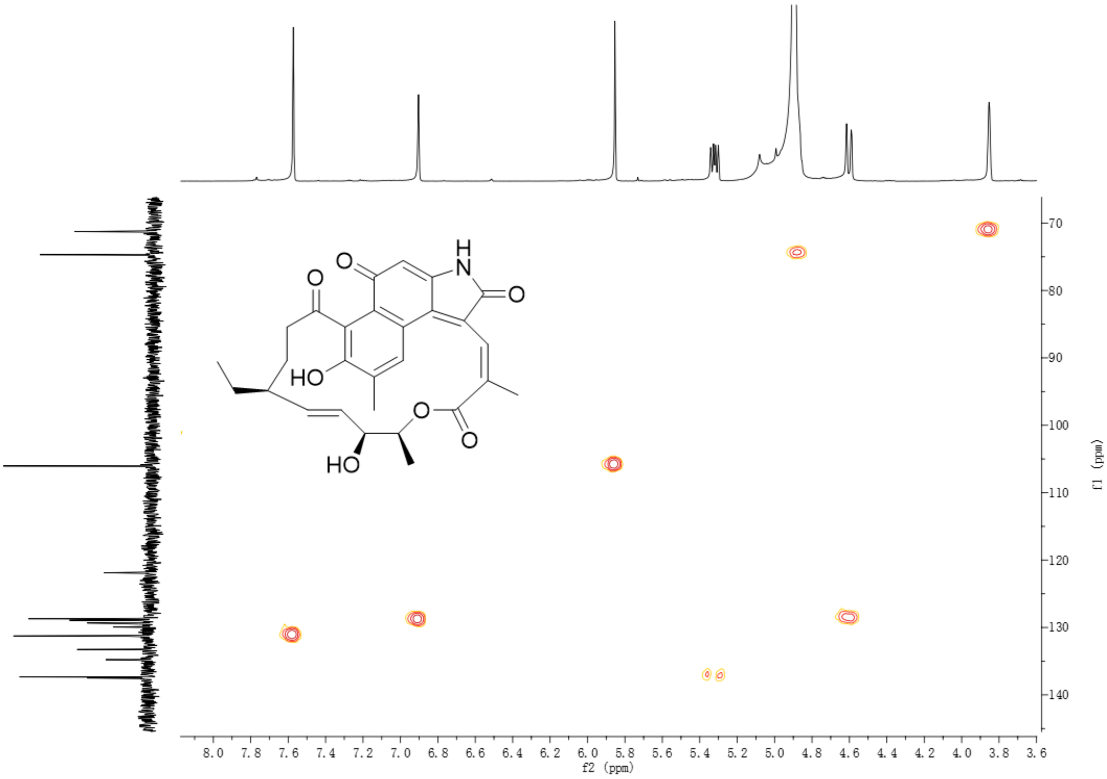

Figure S45. HMQC spectrum of hygrocin M (**7**)

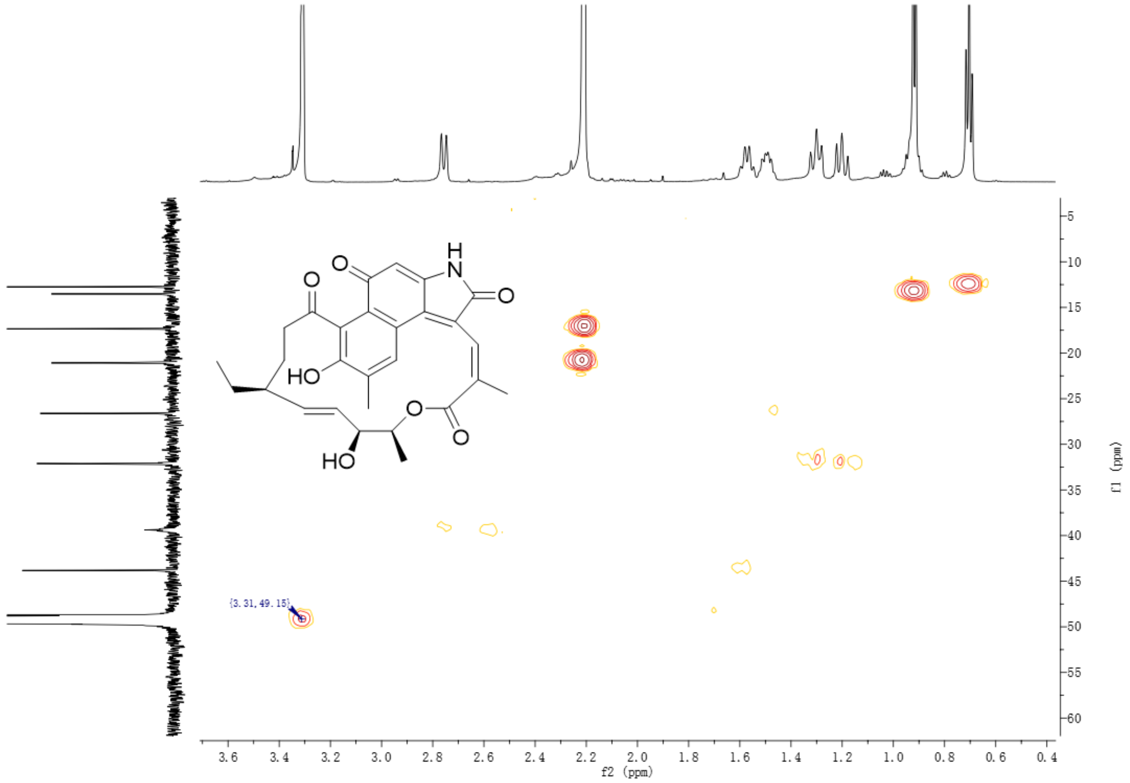

Figure S46. COSY spectrum of hygrocin M (**7**)

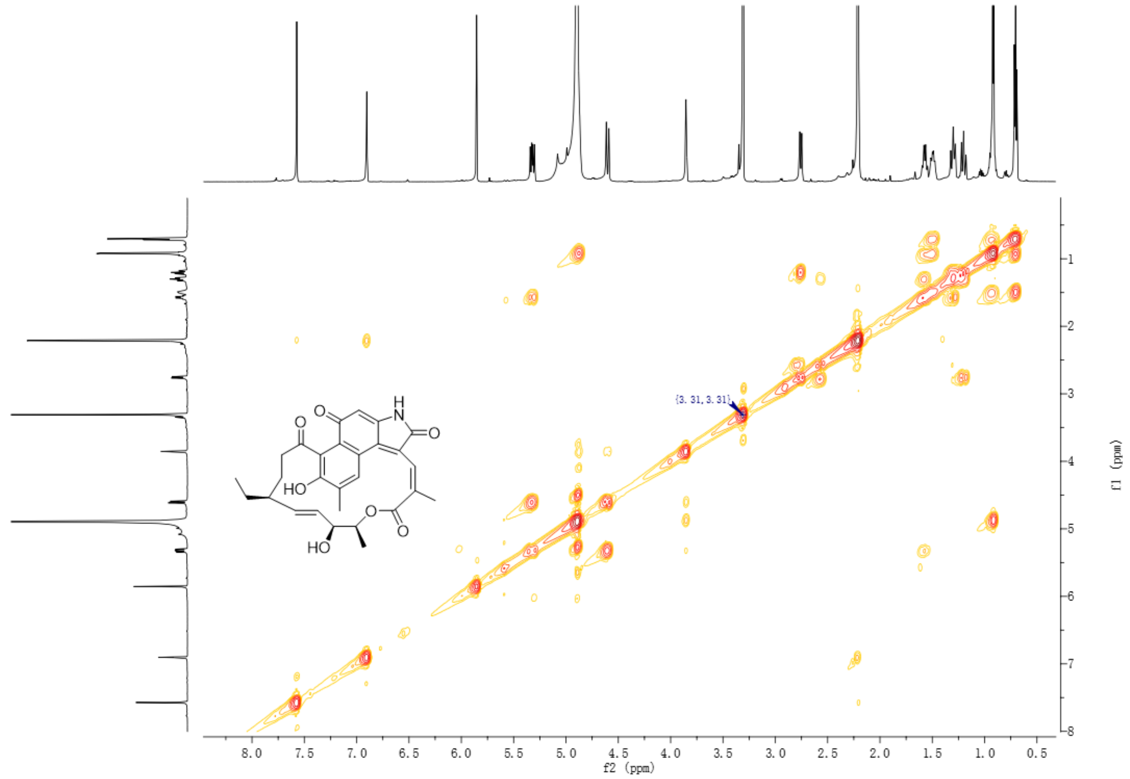

Figure S47. HMBC spectrum of hygrocin M (**7**)

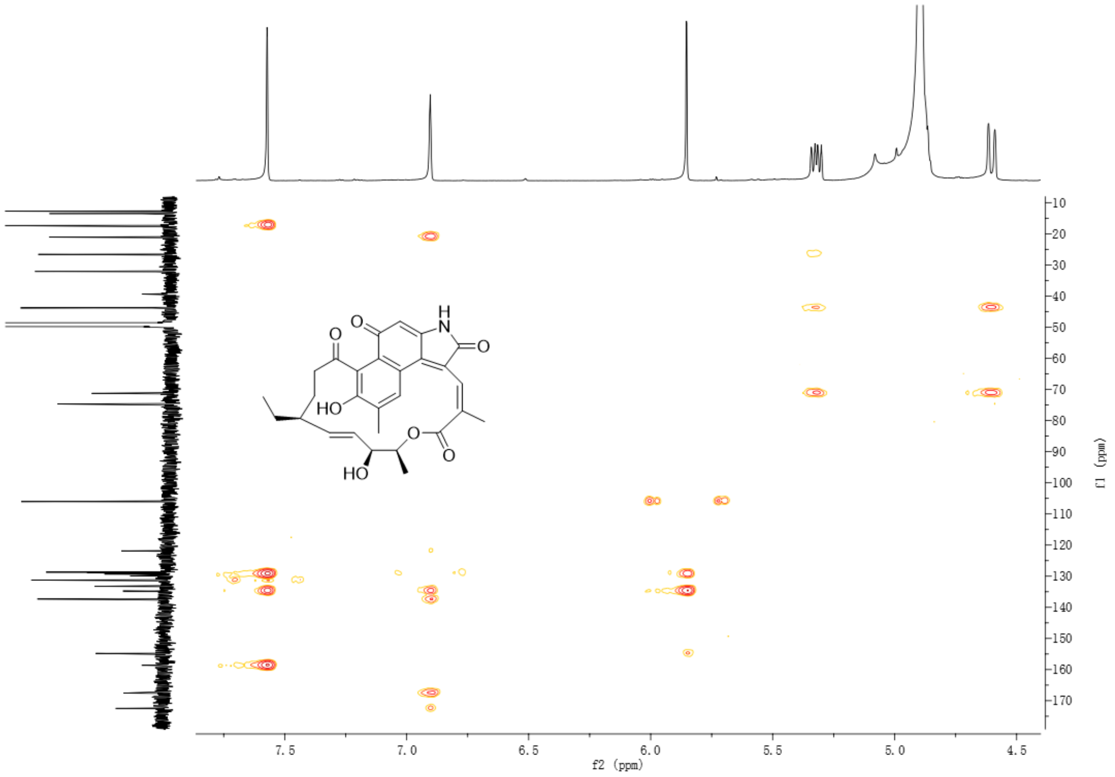

Figure S48. HMBC spectrum of hygrocin M (**7**)

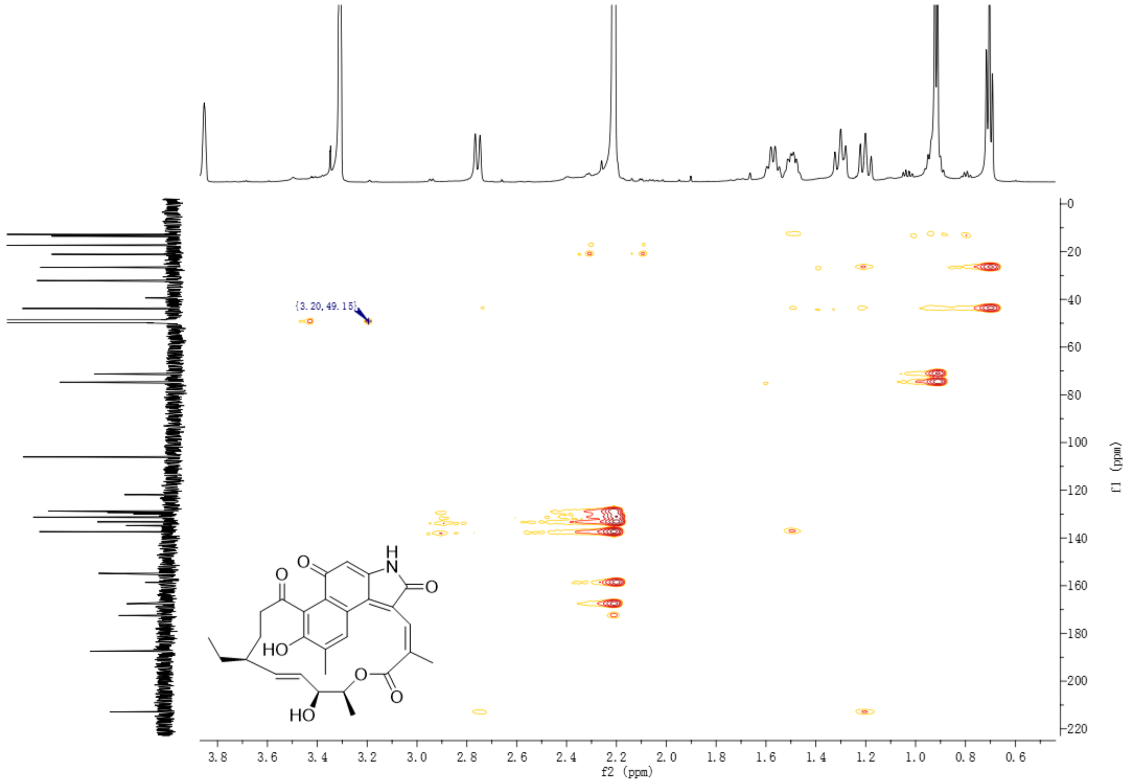

Figure S49. NOESY spectrum of hygrocin M (**7**)

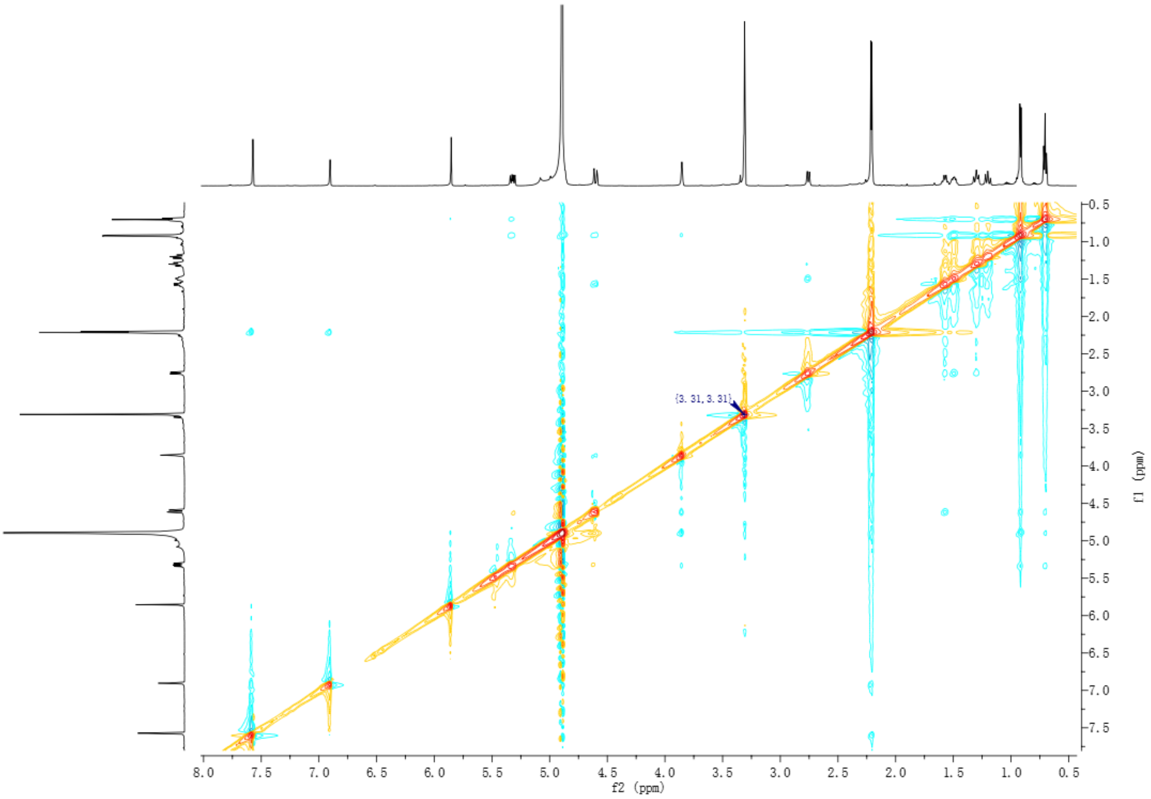

Figure S50. HRESIMS spectrum of hygrocin M (**7**)

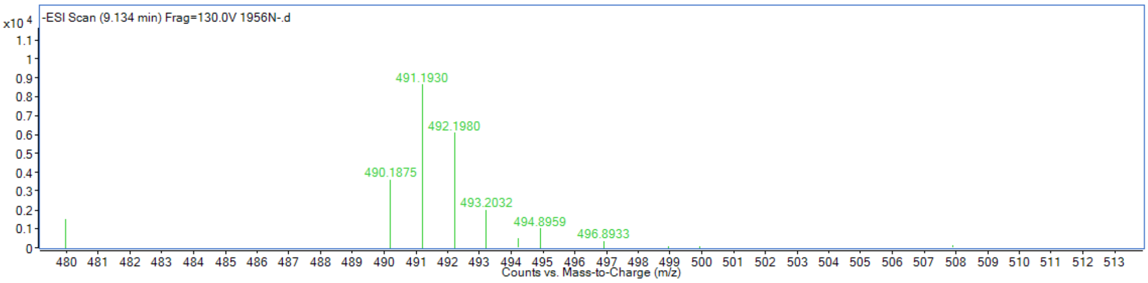

Figure S51. UV spectrum of hygrocin M (**7**)

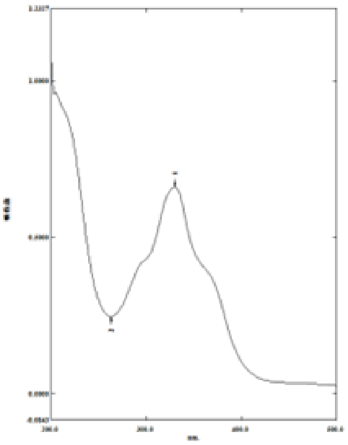

Figure S52. IR spectrum of hygrocin M (**7**)

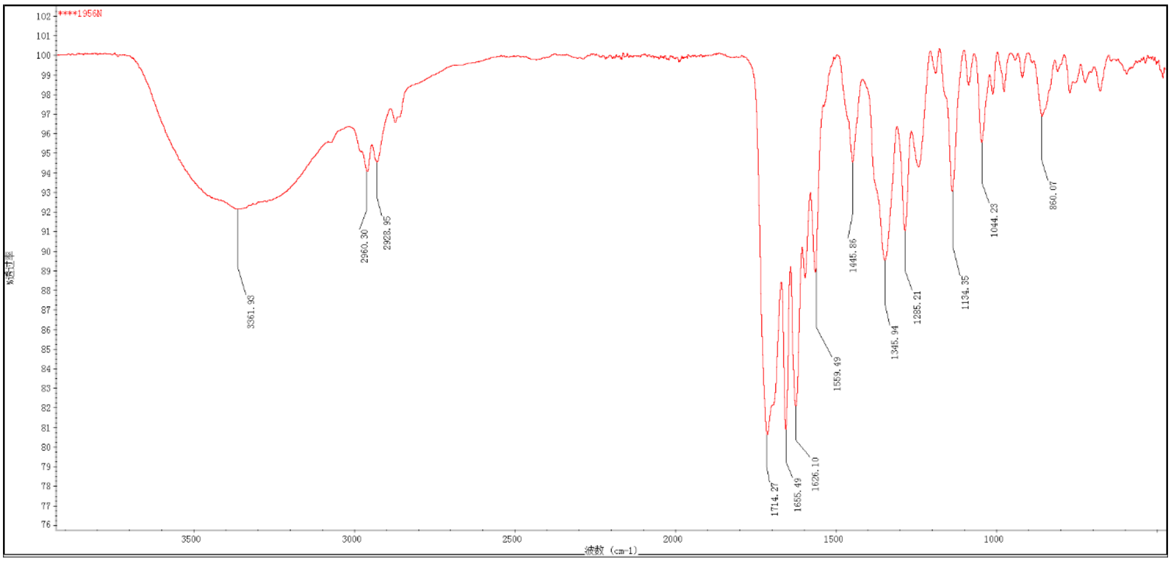

Figure S53. ^{1}H NMR spectrum of hygrocin N (**8**)

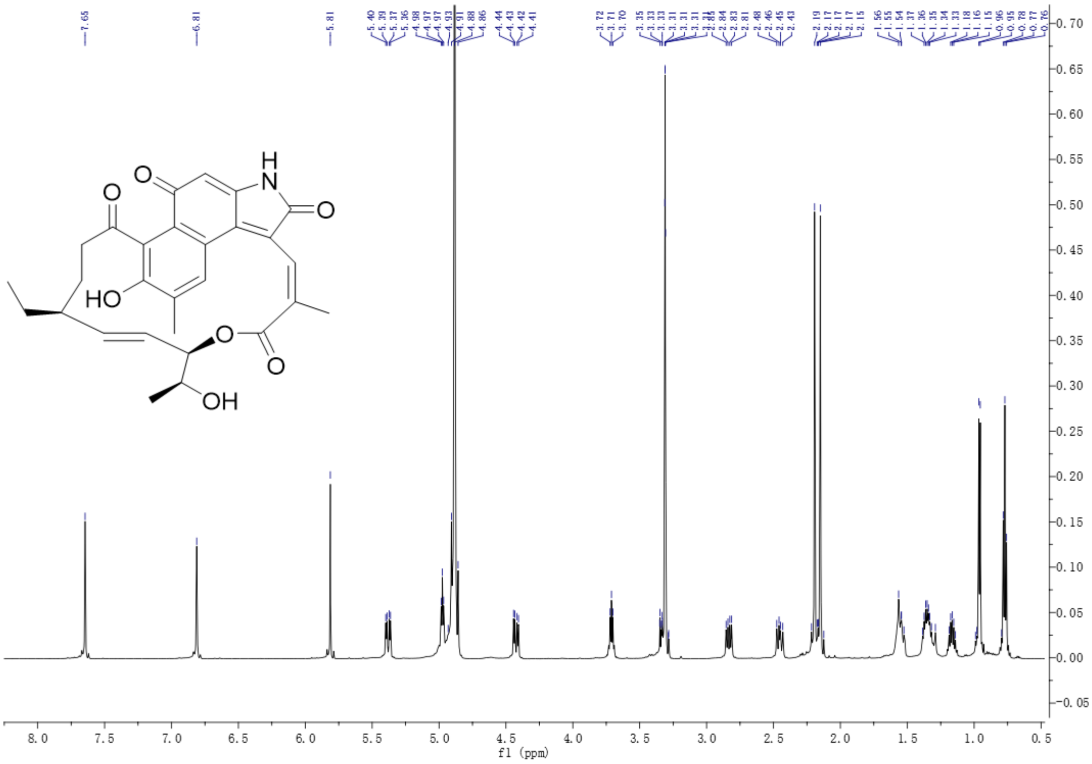

Figure S54. ^{1}H NMR spectrum of hygrocin N (**8**)

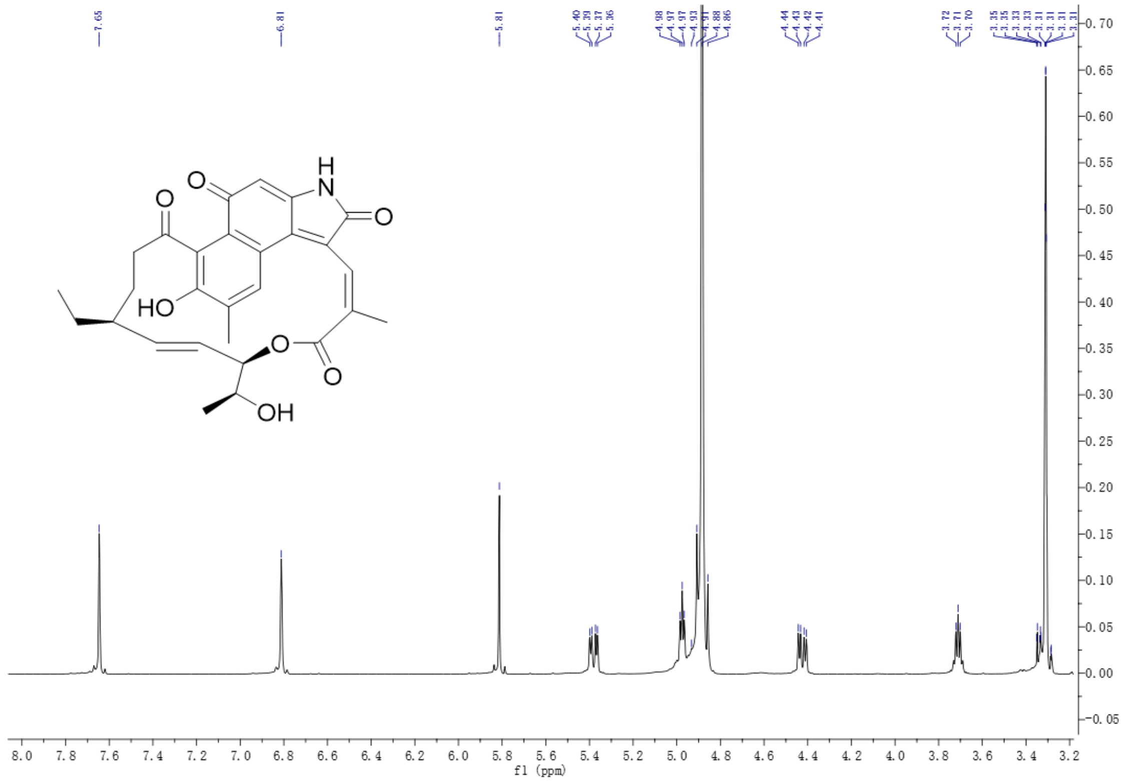

Figure S55. ^{1}H NMR spectrum of hygrocin N (**8**)

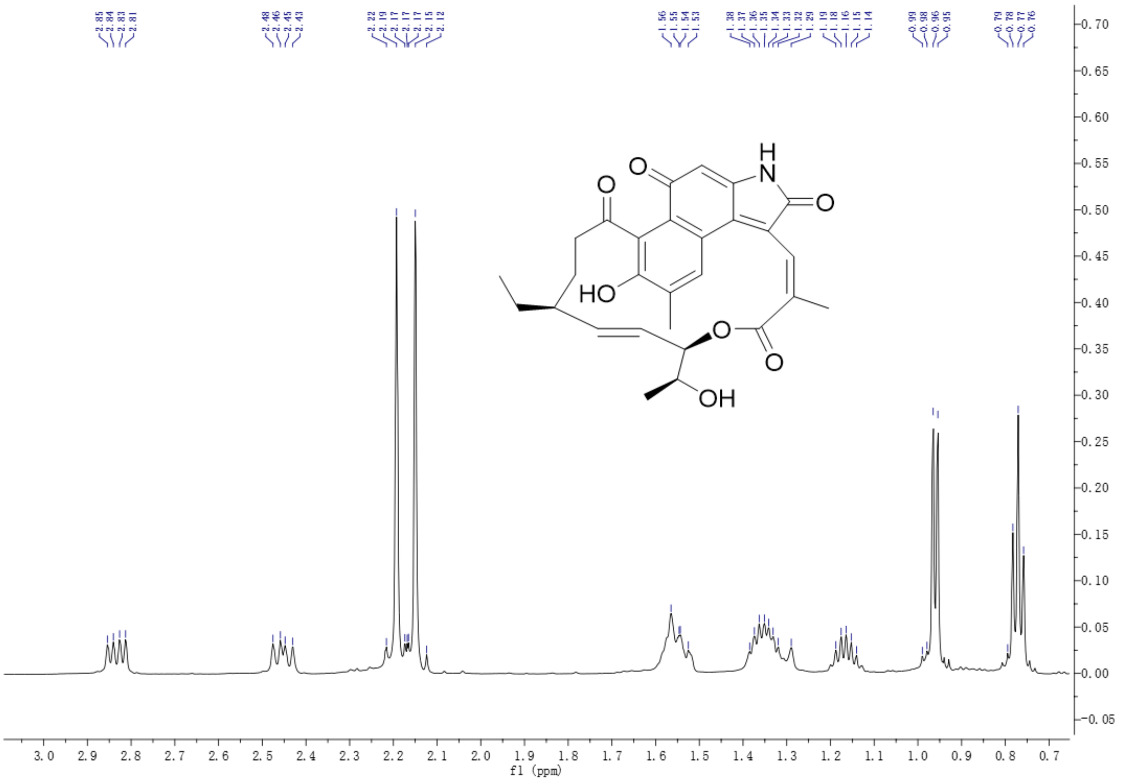

Figure S56. ^{13}C NMR spectrum of hygrocin N (**8**)

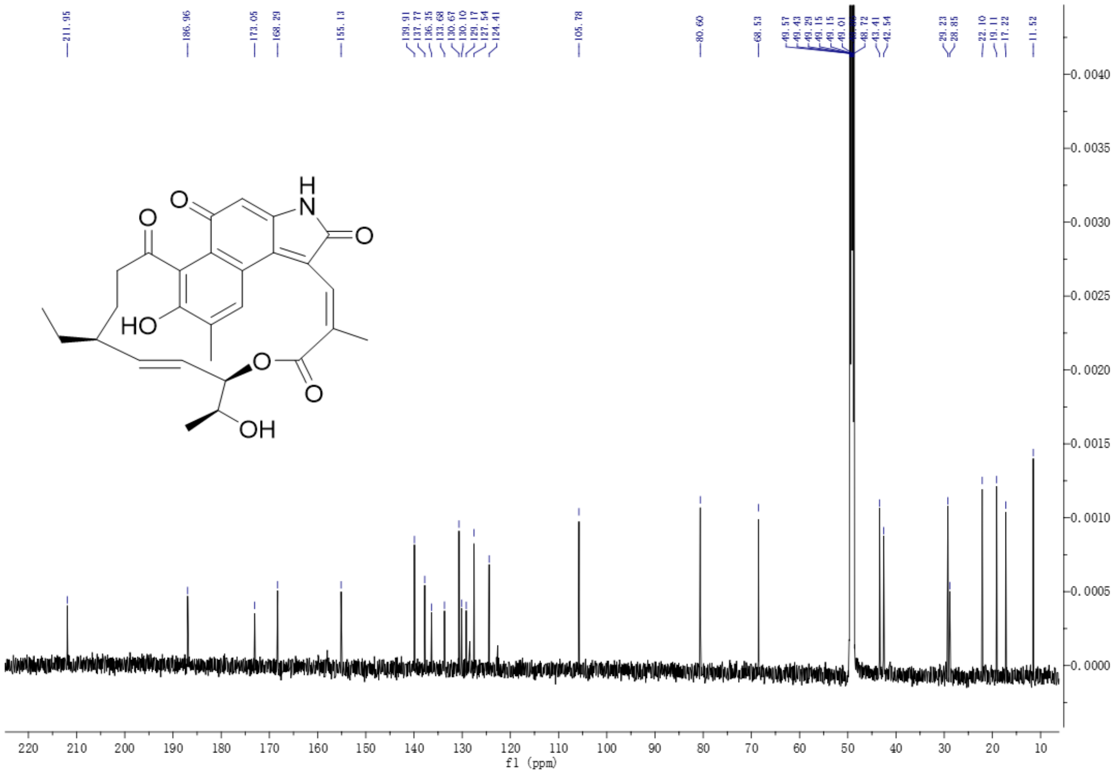

Figure S57. ^{13}C NMR spectrum of hygrocin N (**8**)

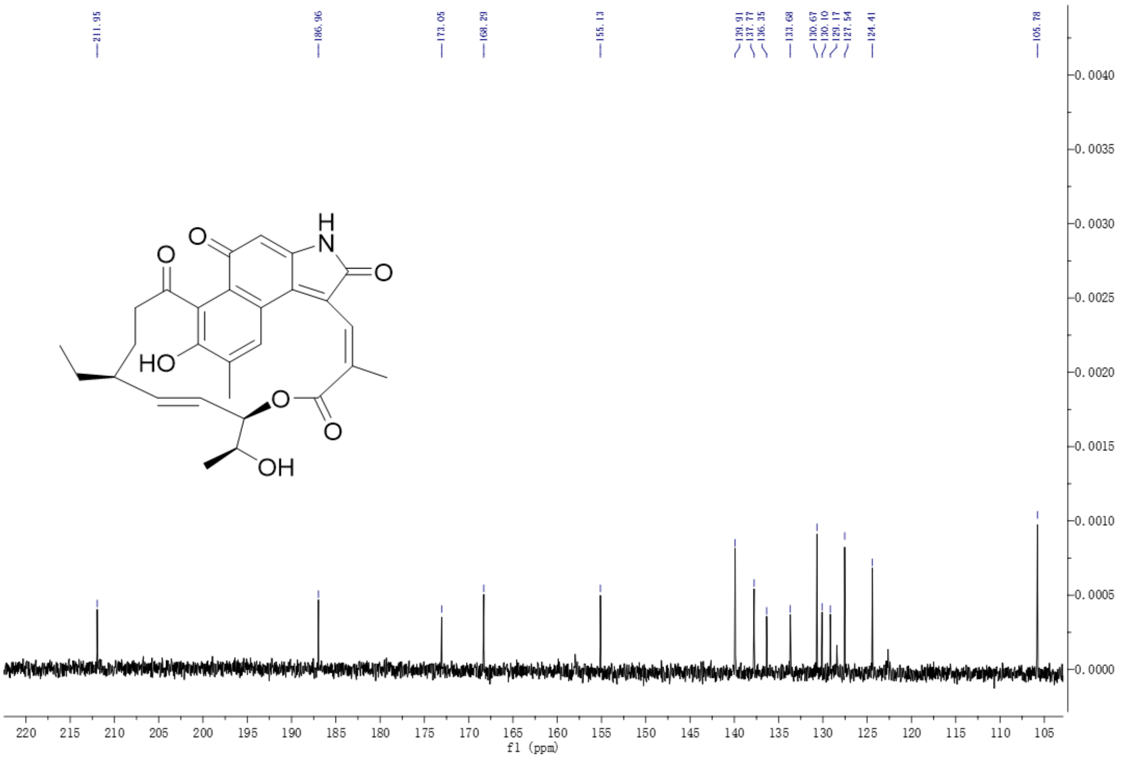

Figure S58. ^{13}C NMR spectrum of hygrocin N (**8**)

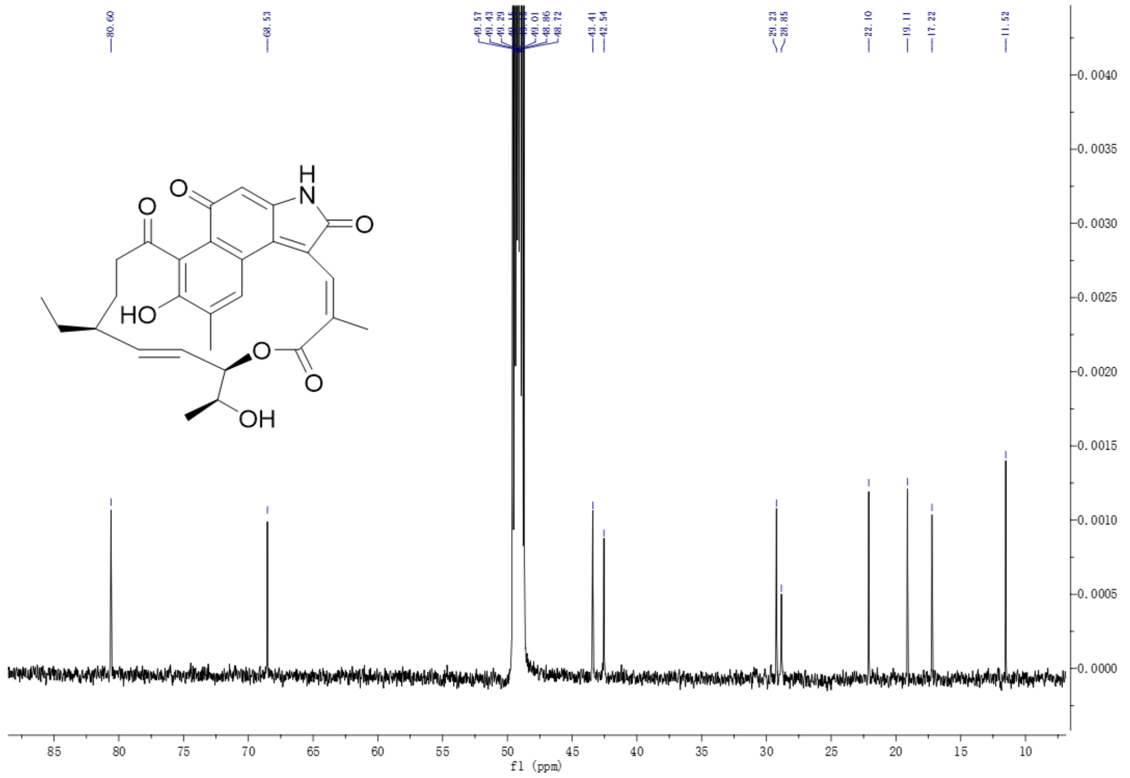

Figure S59. HMQC spectrum of hygrocin N (**8**)

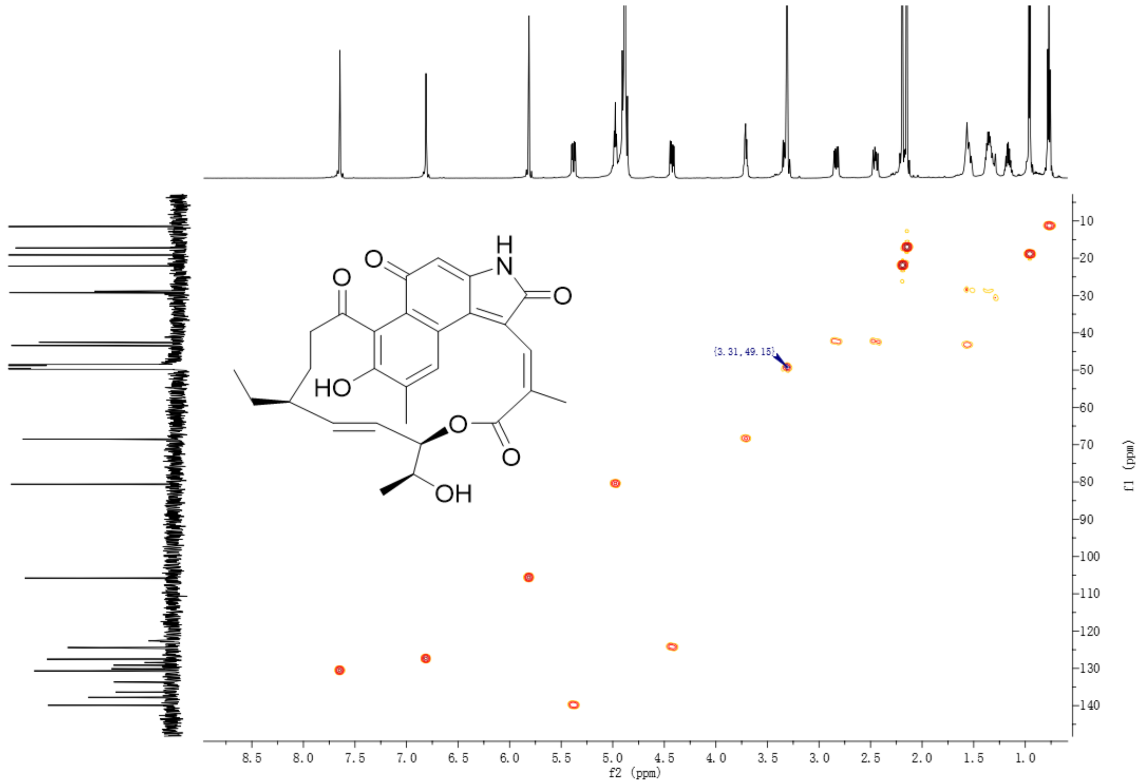

Figure S60. HMQC spectrum of hygrocin N (**8**)

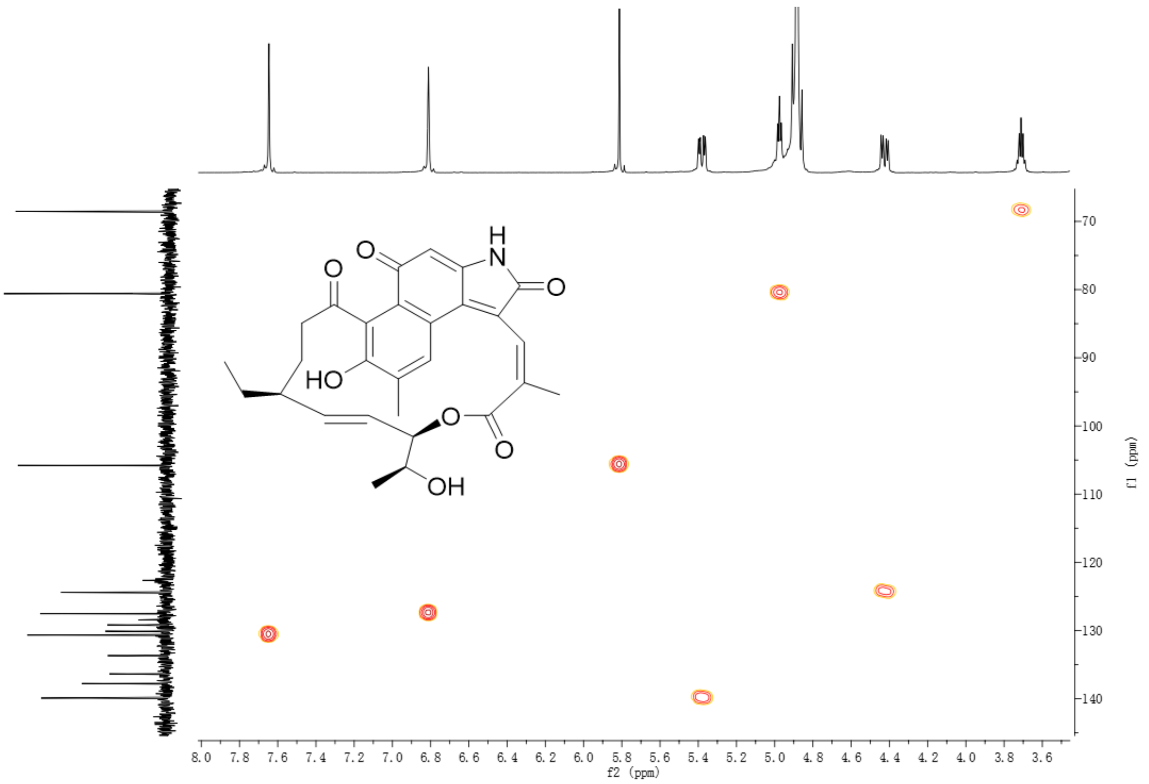

Figure S61. HMQC spectrum of hygrocin N (**8**)

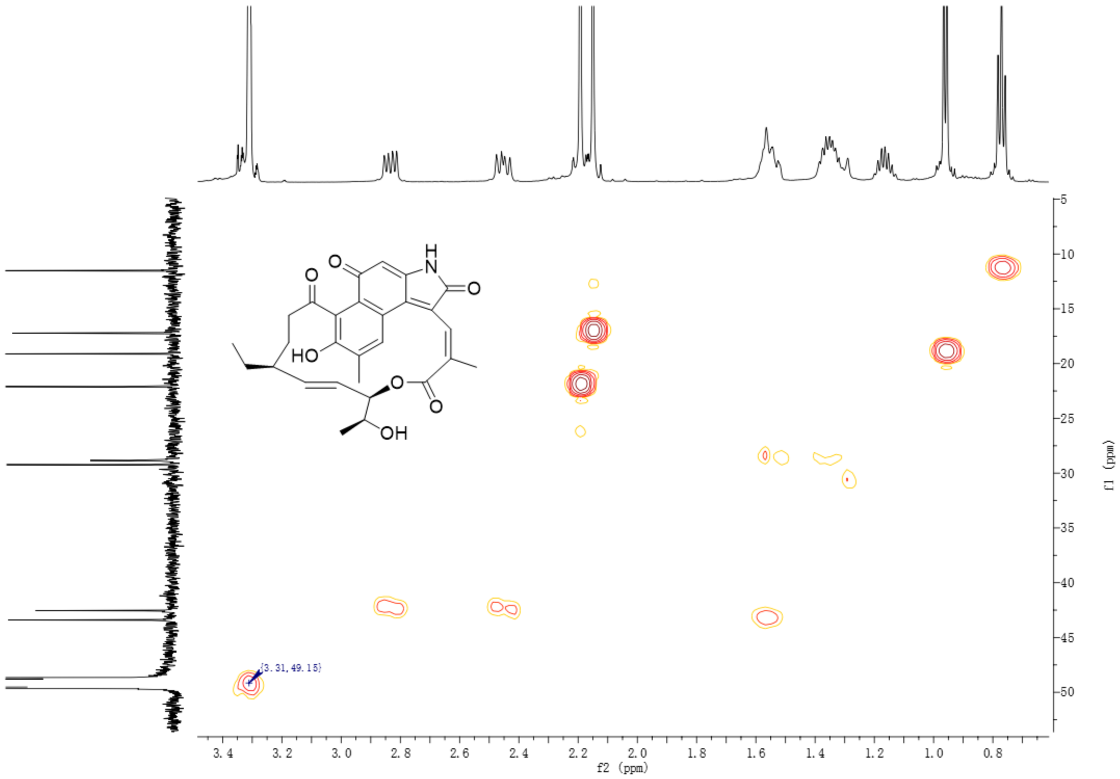

Figure S62. COSY spectrum of hygrocin N (**8**)

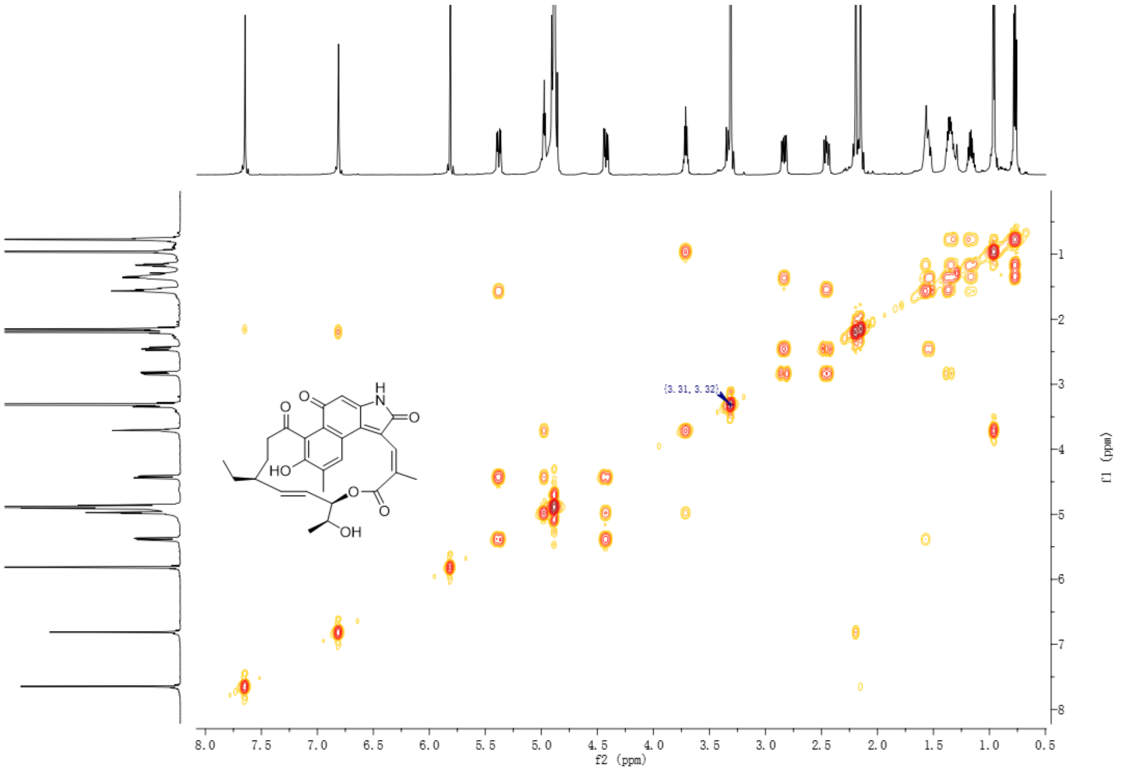

Figure S63. HMBC spectrum of hygrocin N (**8**)

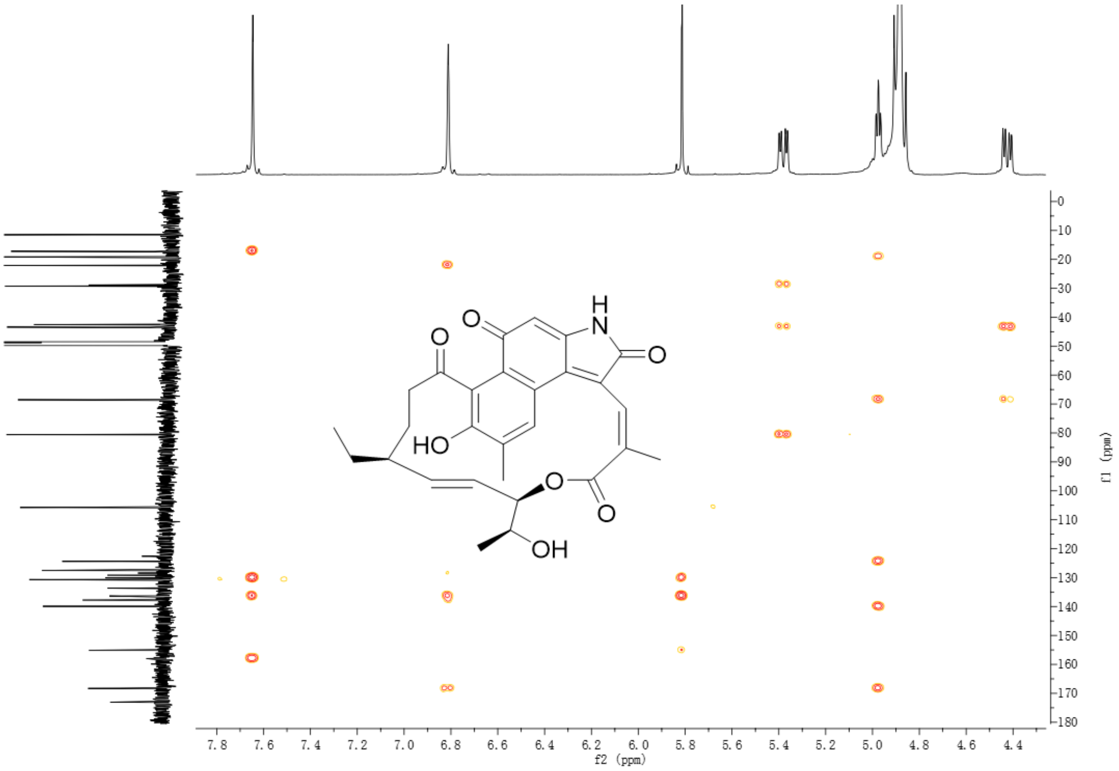

Figure S64. HMBC spectrum of hygrocin N (**8**)

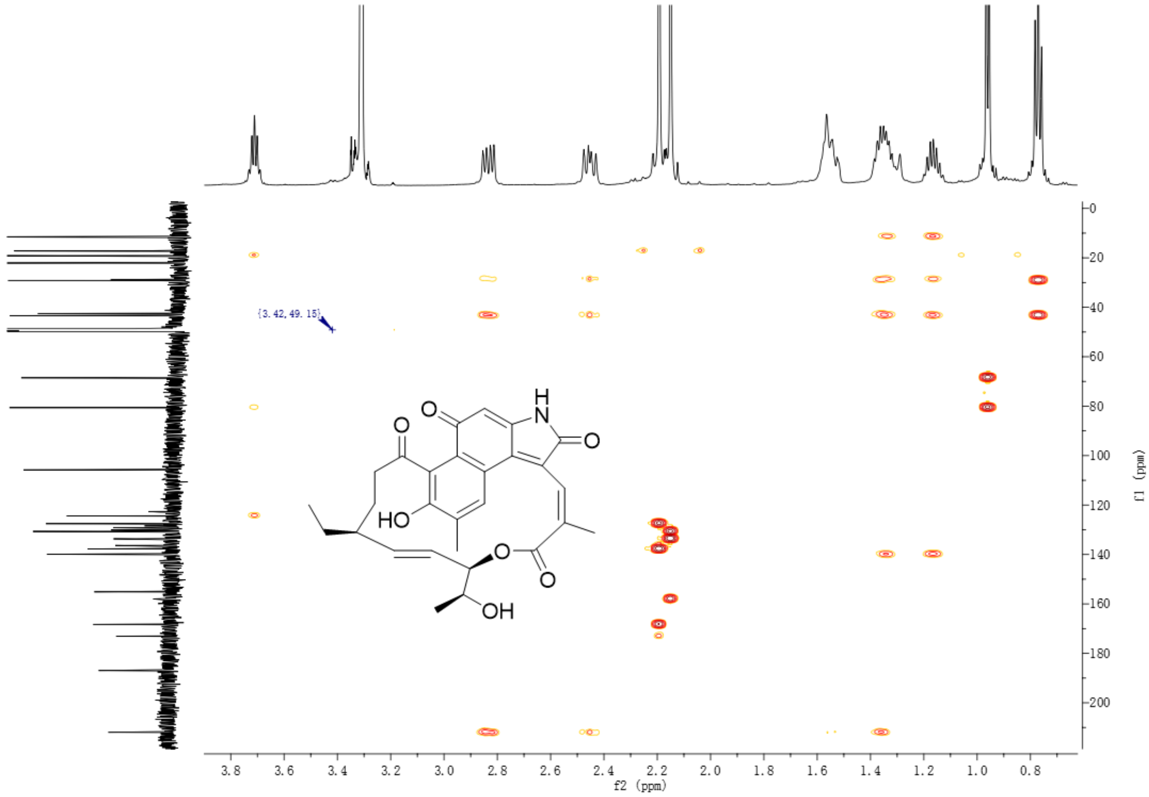

Figure S65. NOESY spectrum of hygrocin N (**8**)

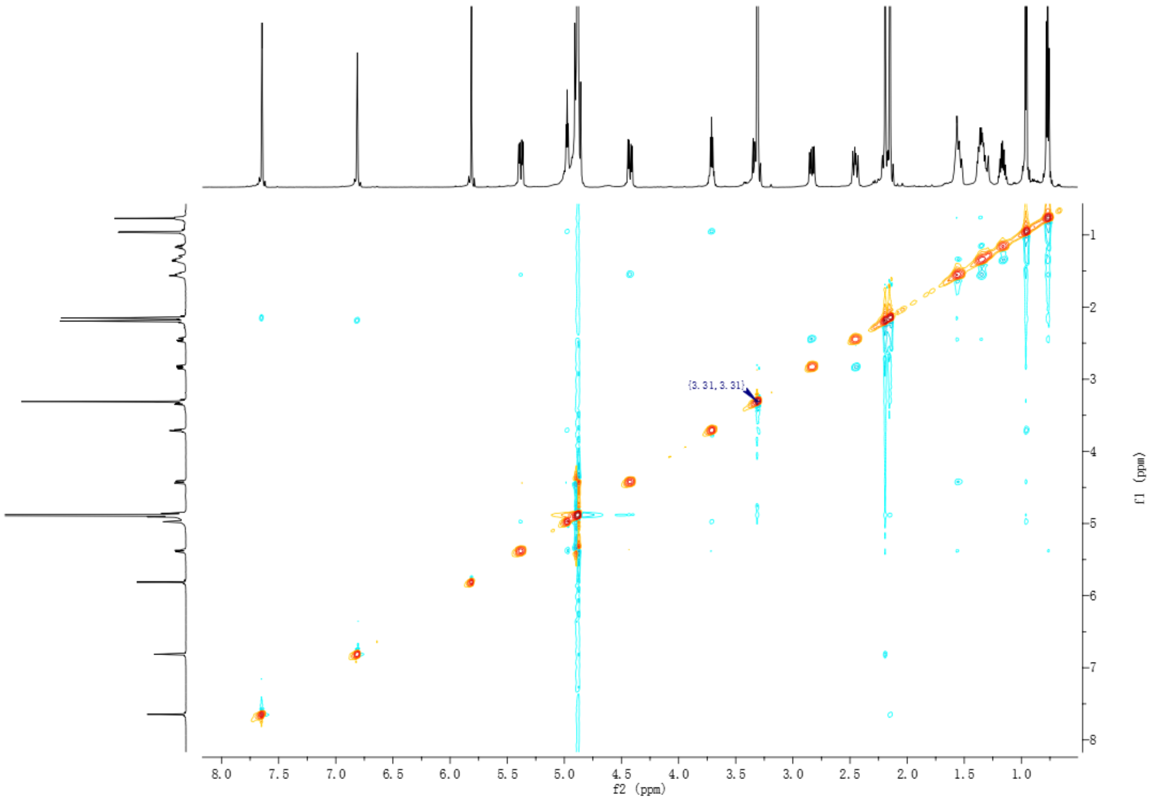

Figure S66. HRESIMS spectrum of hygrocin N (**8**)

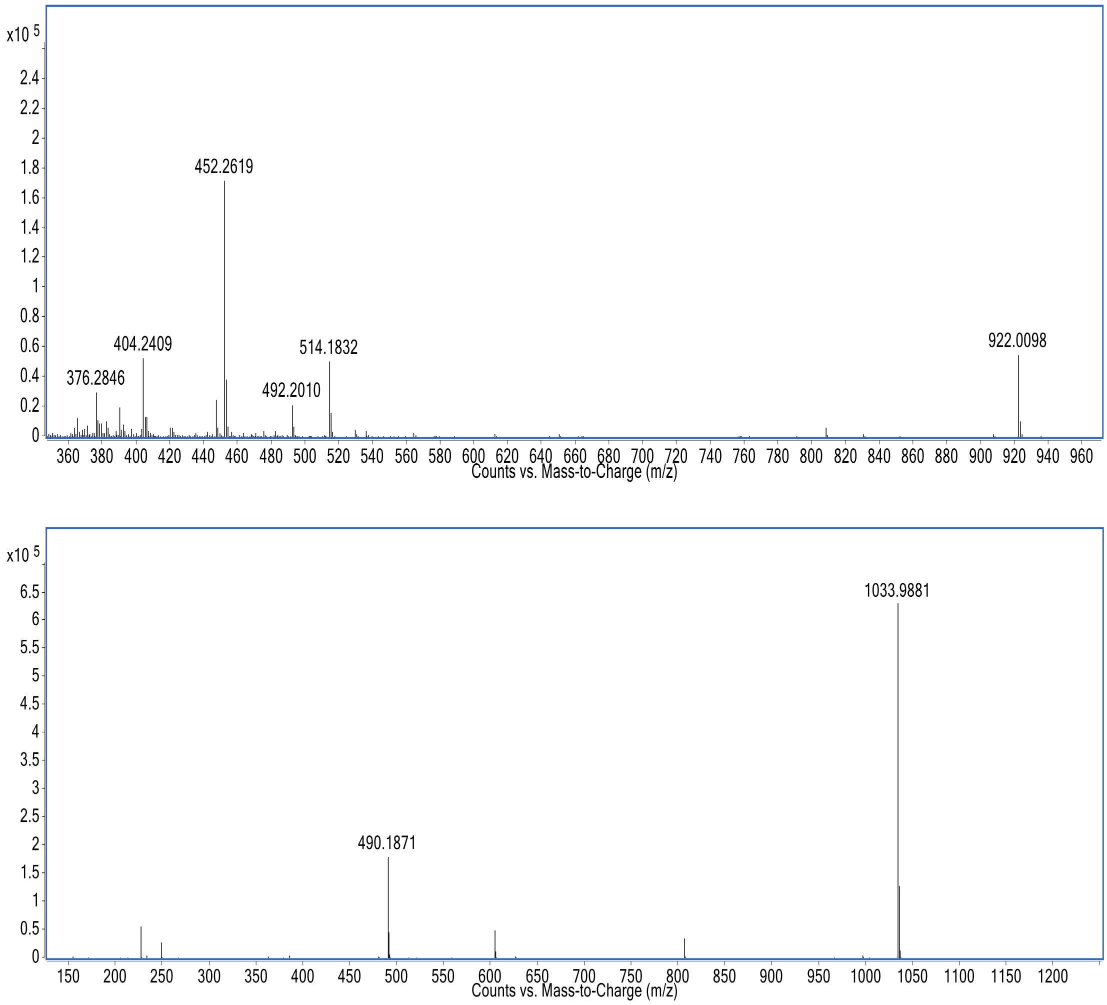

Figure S67. UV spectrum of hygrocin N (**8**)

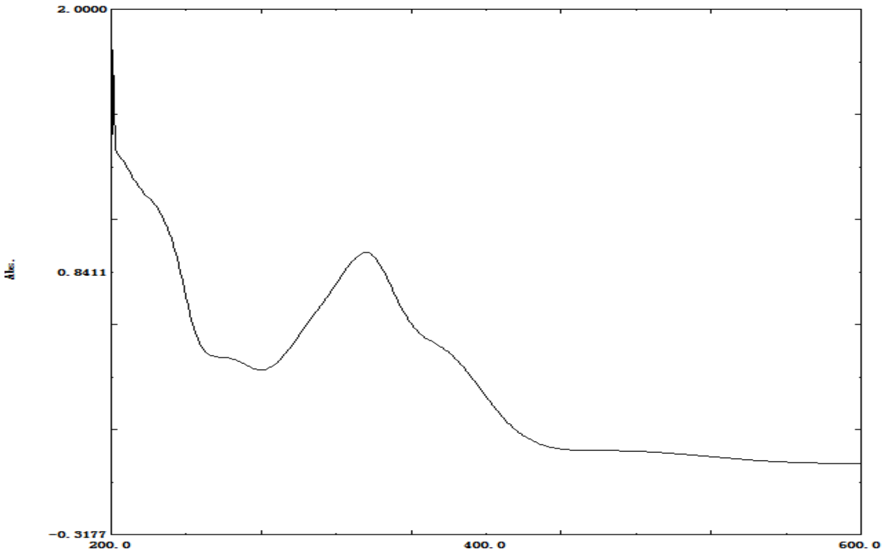

Figure S68. IR spectrum of hygrocin N (**8**)

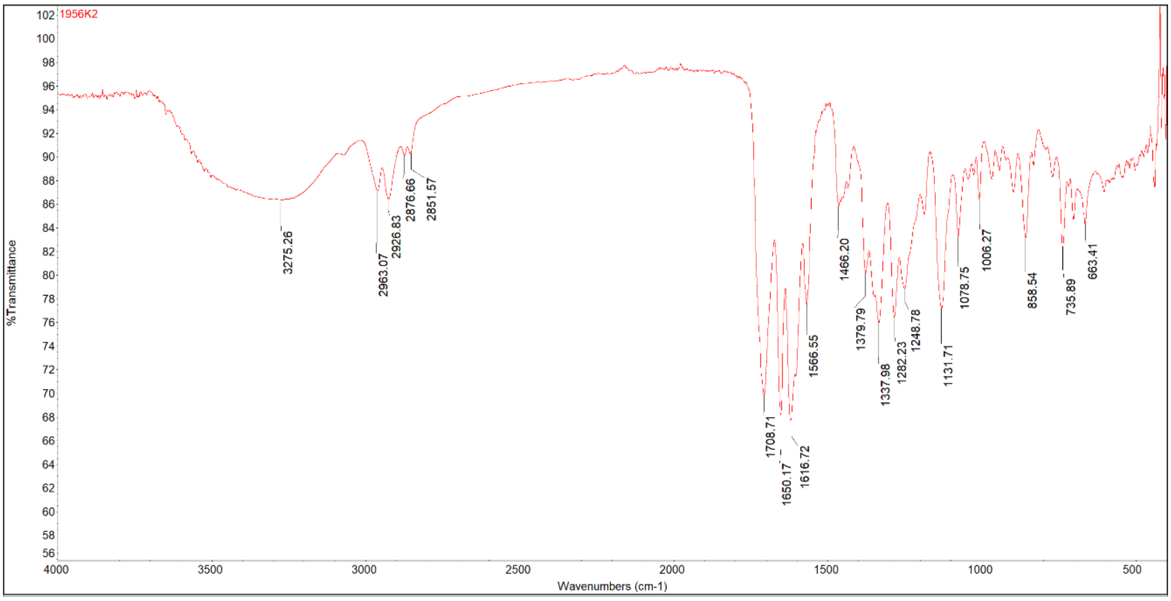

Figure S69. ^{1}H NMR spectrum of **8s** (600 MHz, in MeOH-d_4)

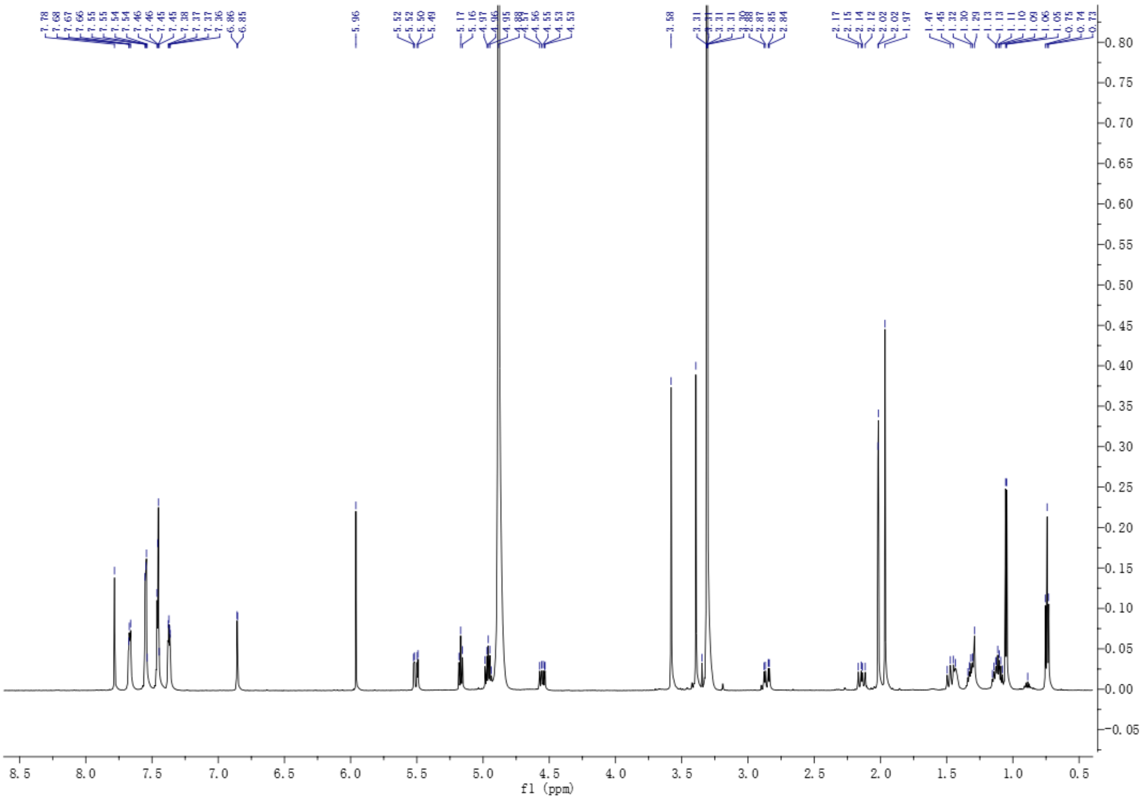

Figure S70. HRESIMS spectrum of **8s**

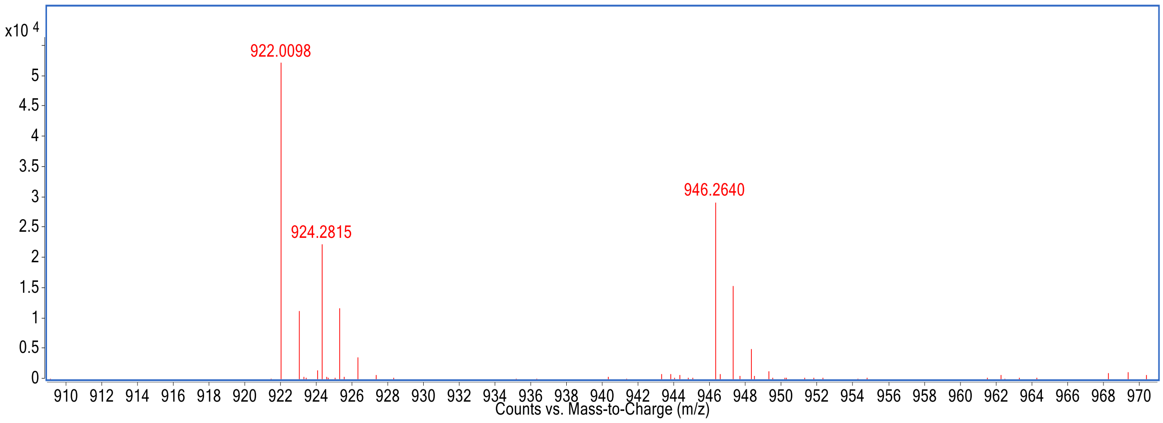

Figure S71. ^{1}H NMR spectrum of **8r** (600 MHz, in MeOH-d_4)

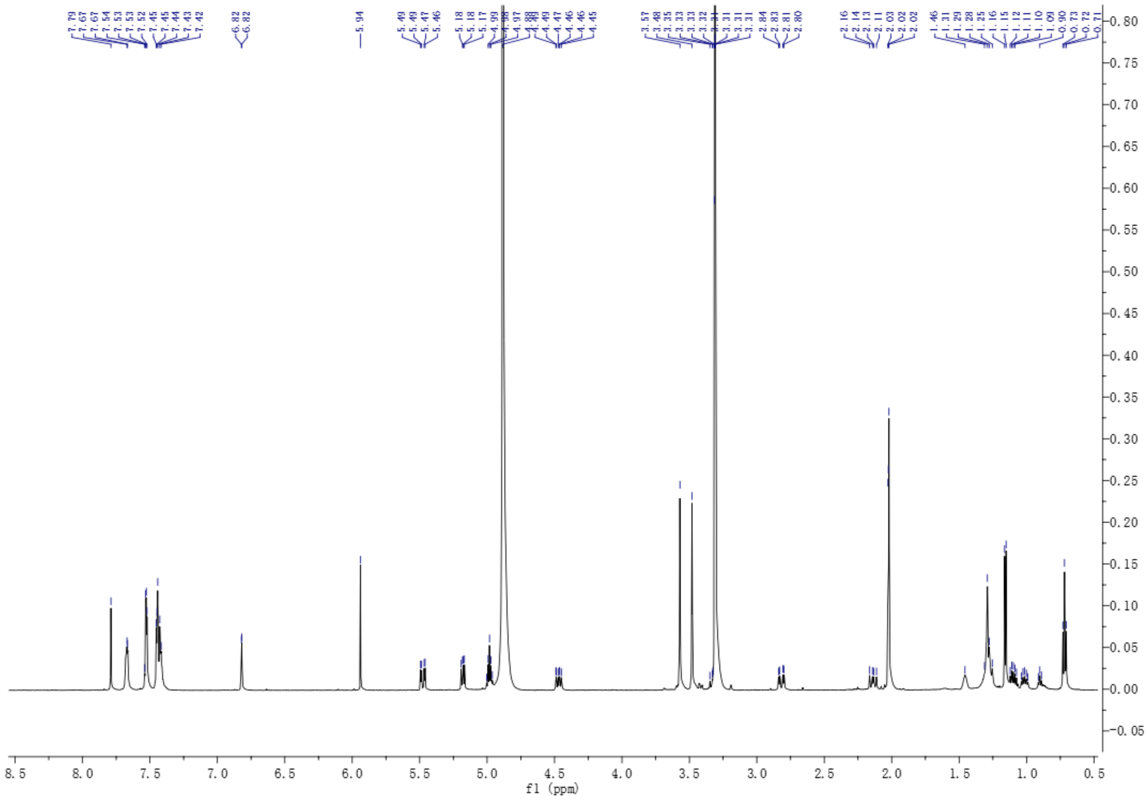

Figure S72. HRESIMS spectrum of **8r**

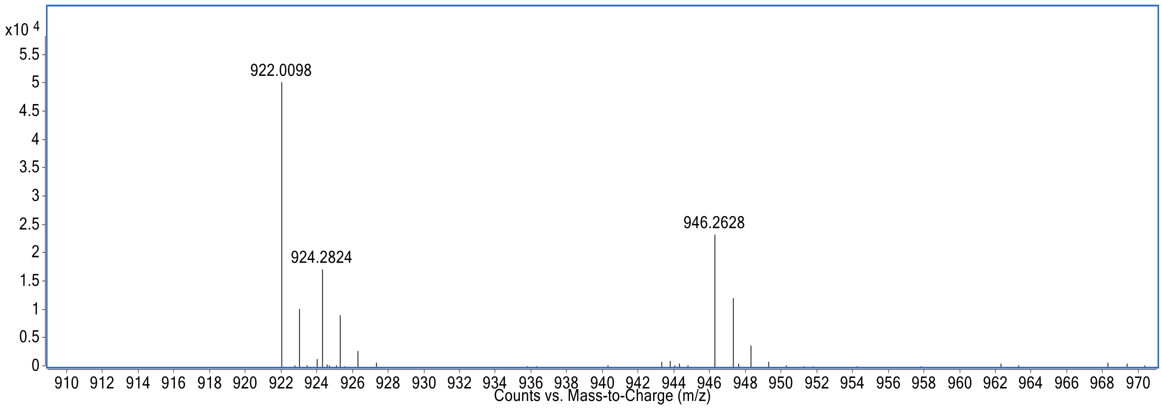

Figure S73. ^{1}H NMR spectrum of hygrocin O (**9**)

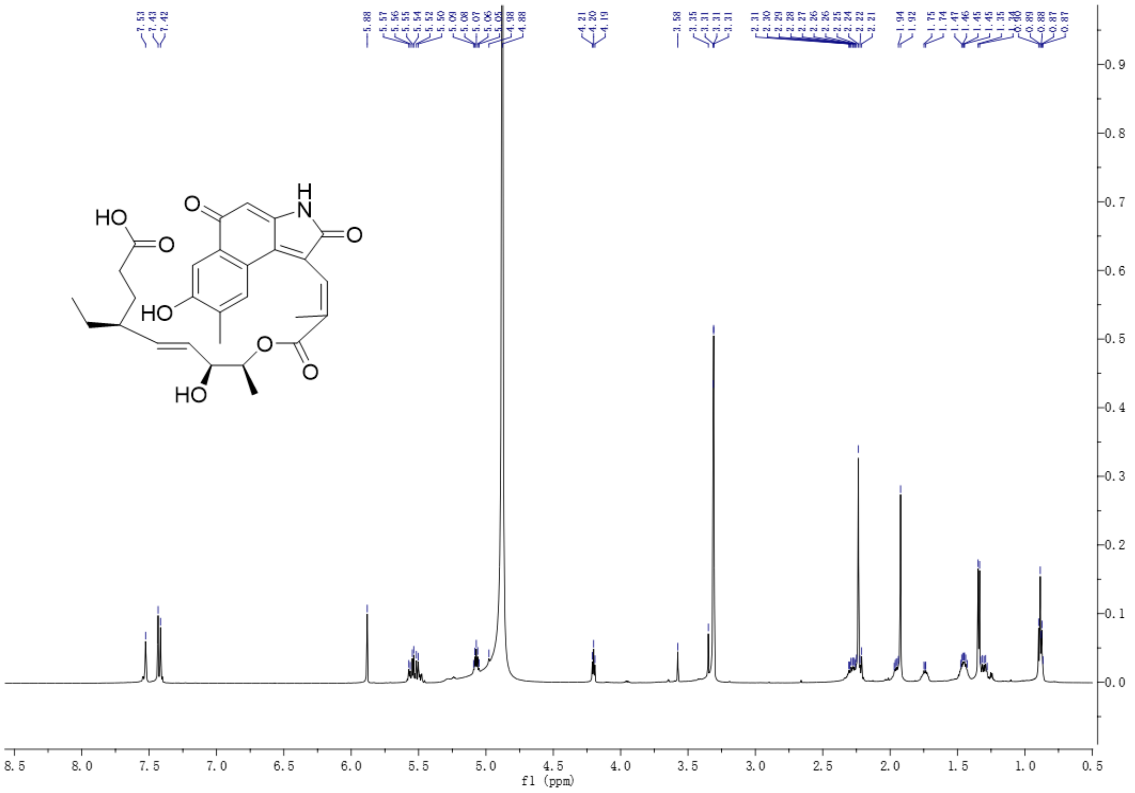

Figure S74. ^{1}H NMR spectrum of hygrocin O (**9**)

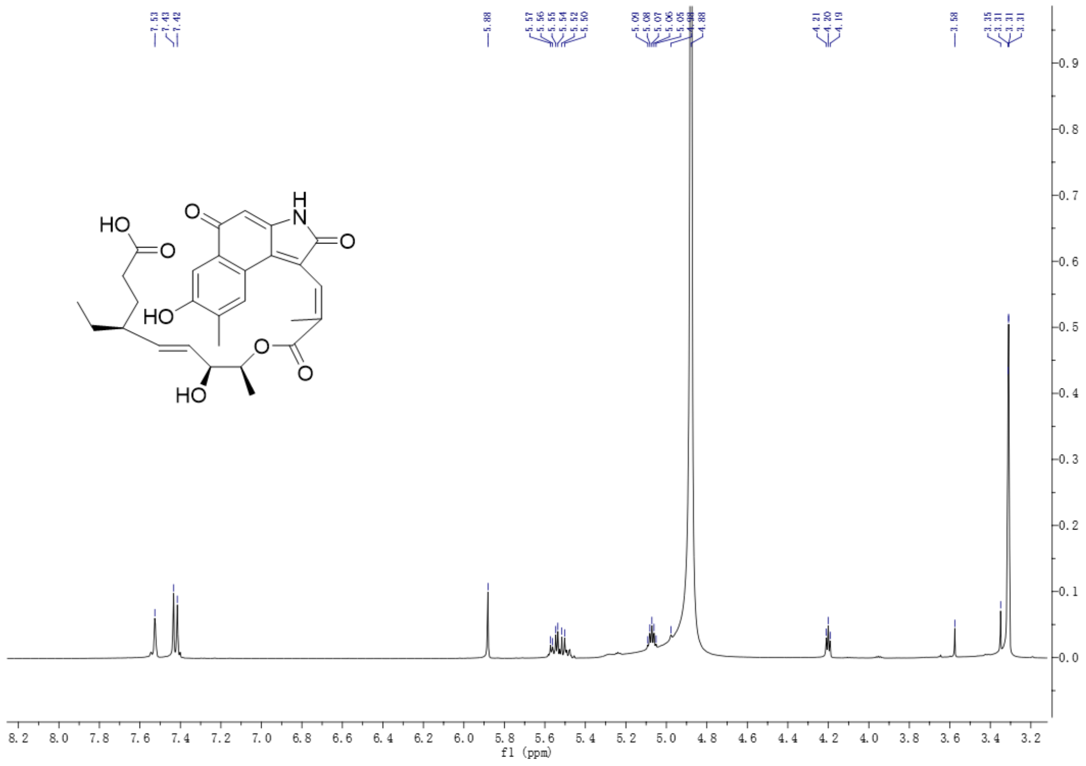

Figure S75. ^{1}H NMR spectrum of hygrocin O (**9**)

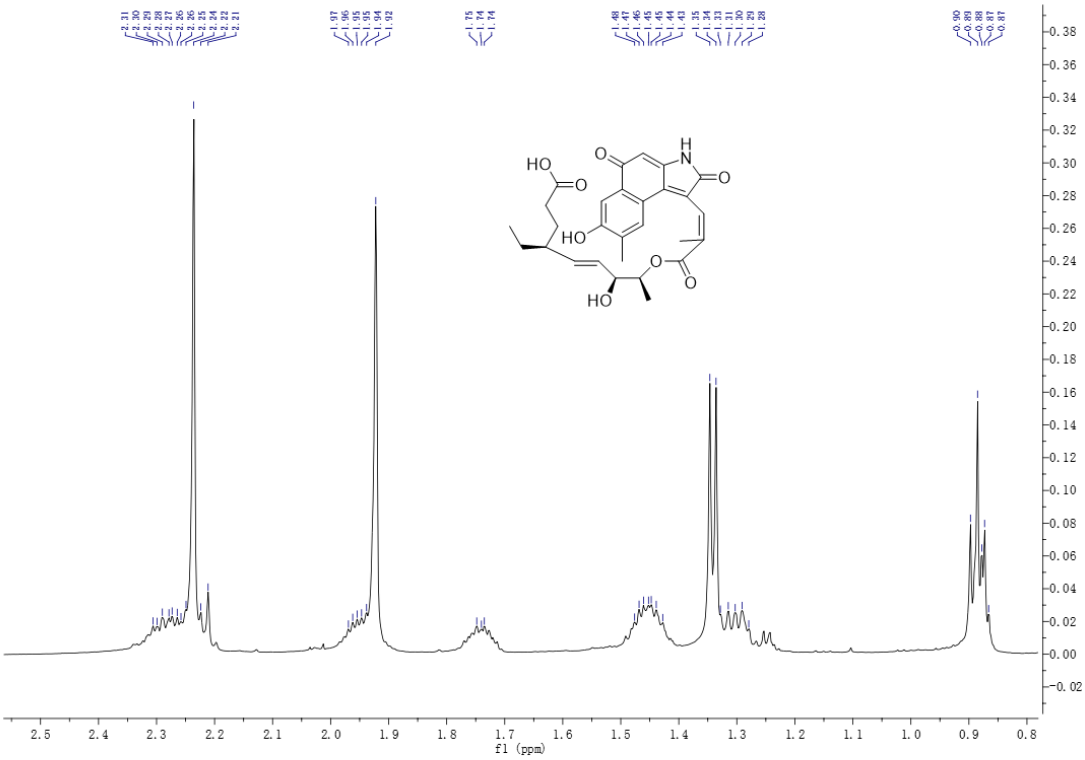

Figure S76. ^{13}C NMR spectrum of hygrocin O (**9**)

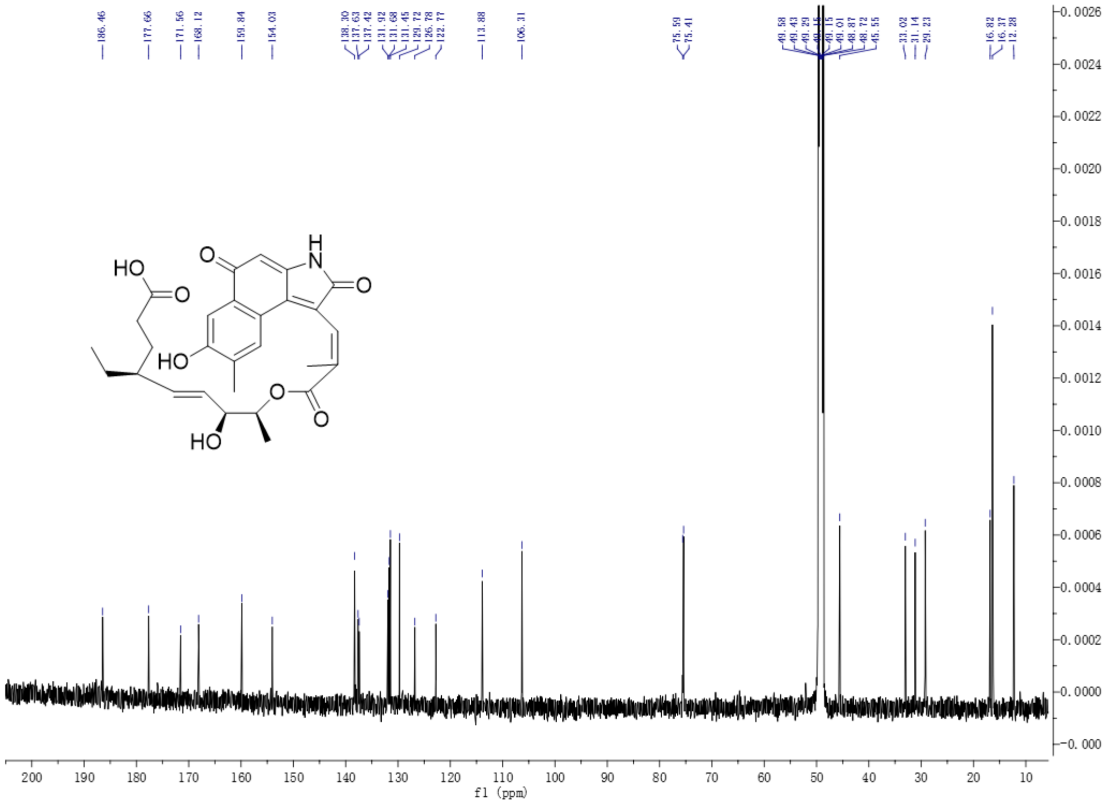

Figure S77. ^{13}C NMR spectrum of hygrocin O (**9**)

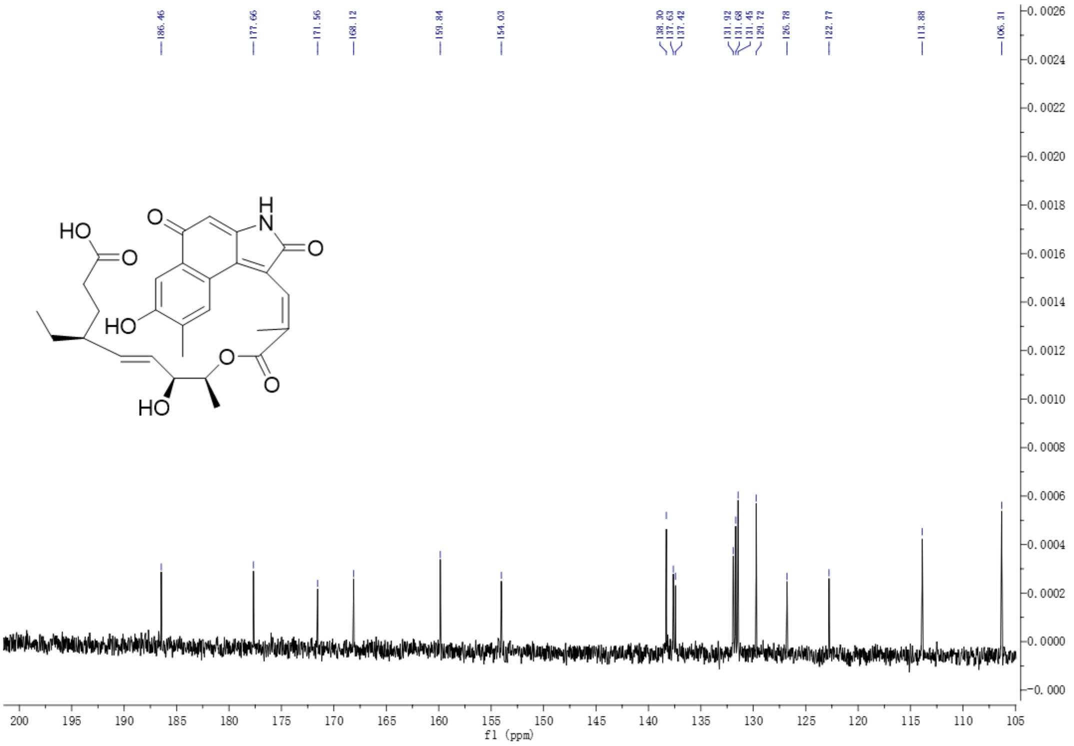

Figure S78. ^{13}C NMR spectrum of hygrocin O (**9**)

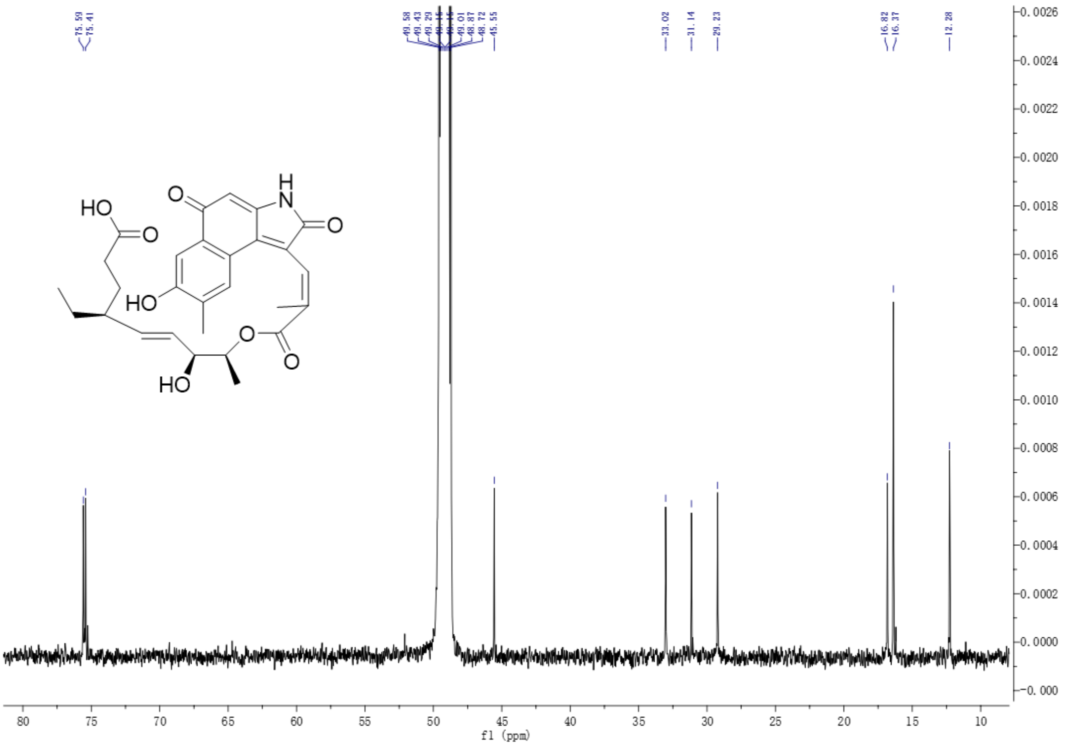

Figure S79. HMQC spectrum of hygrocin O (**9**)

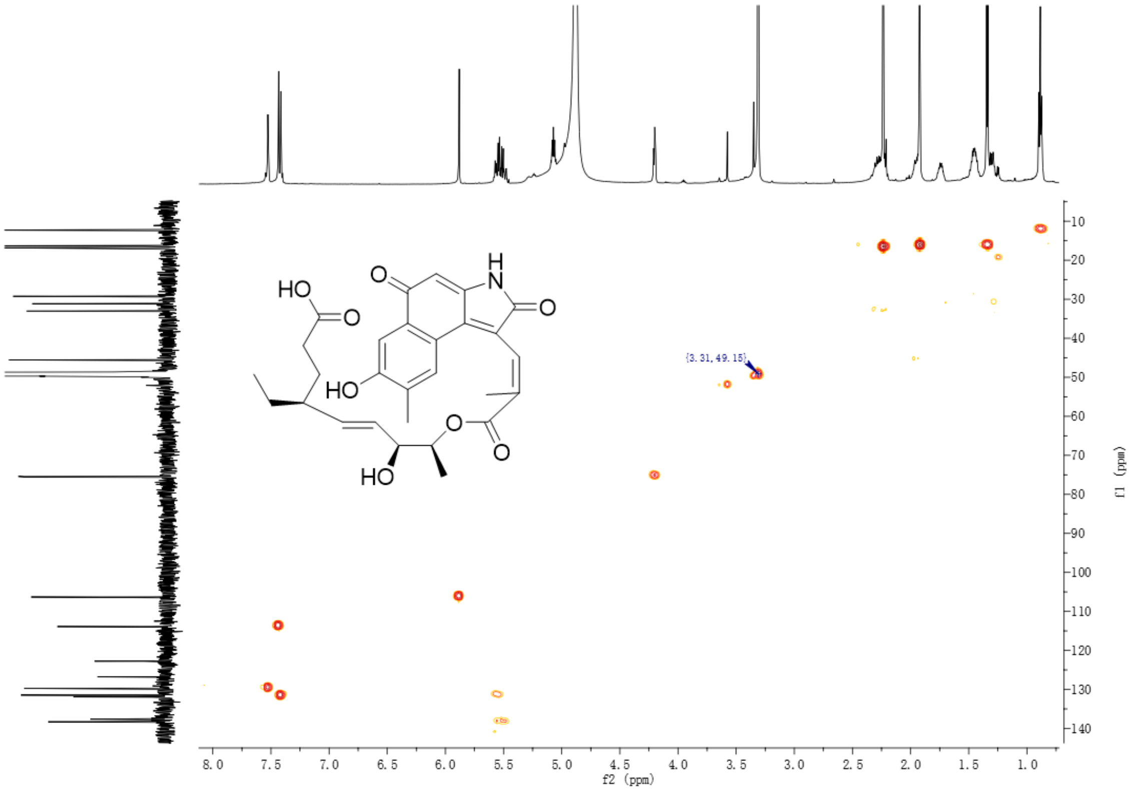

Figure S80. HMQC spectrum of hygrocin O (**9**)

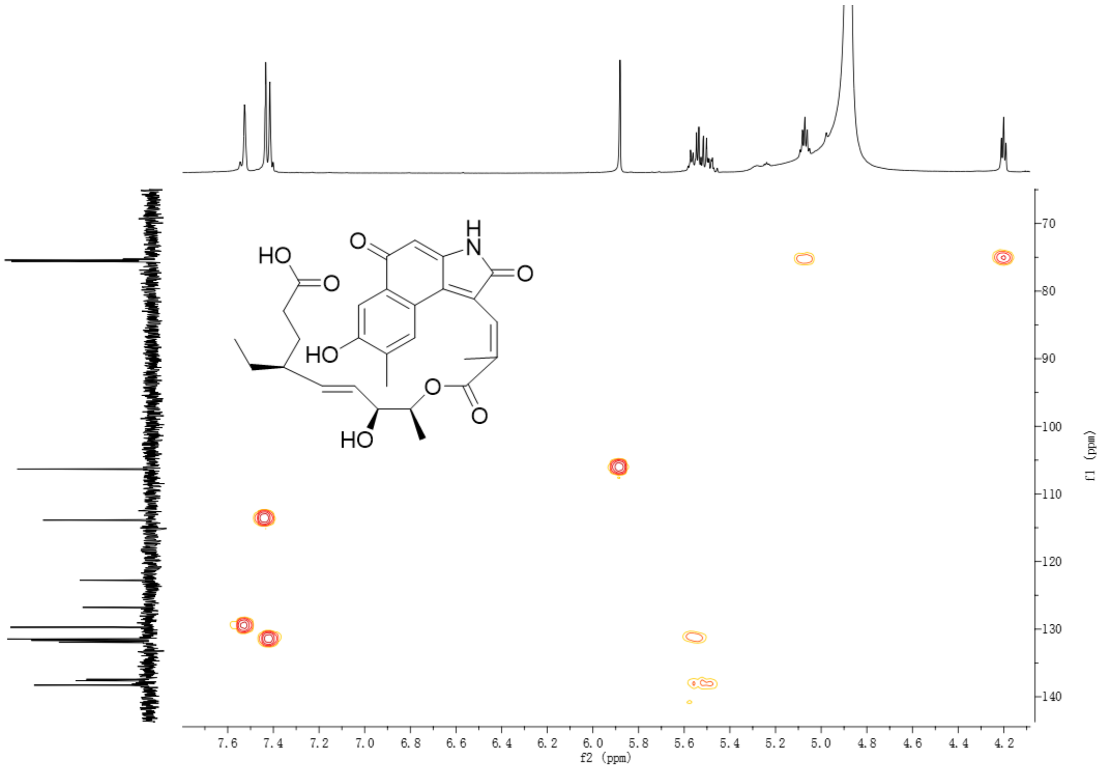

Figure S81. HMQC spectrum of hygrocin O (**9**)

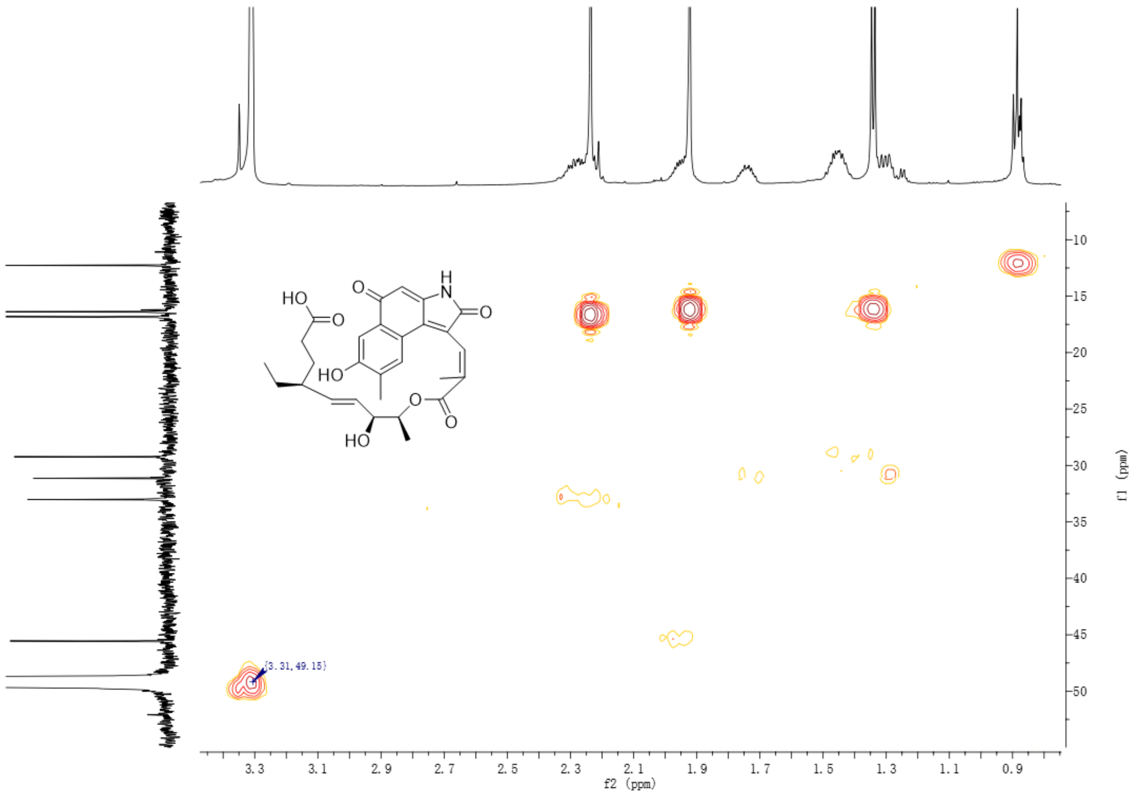

Figure S82. COSY spectrum of hygrocin O (**9**)

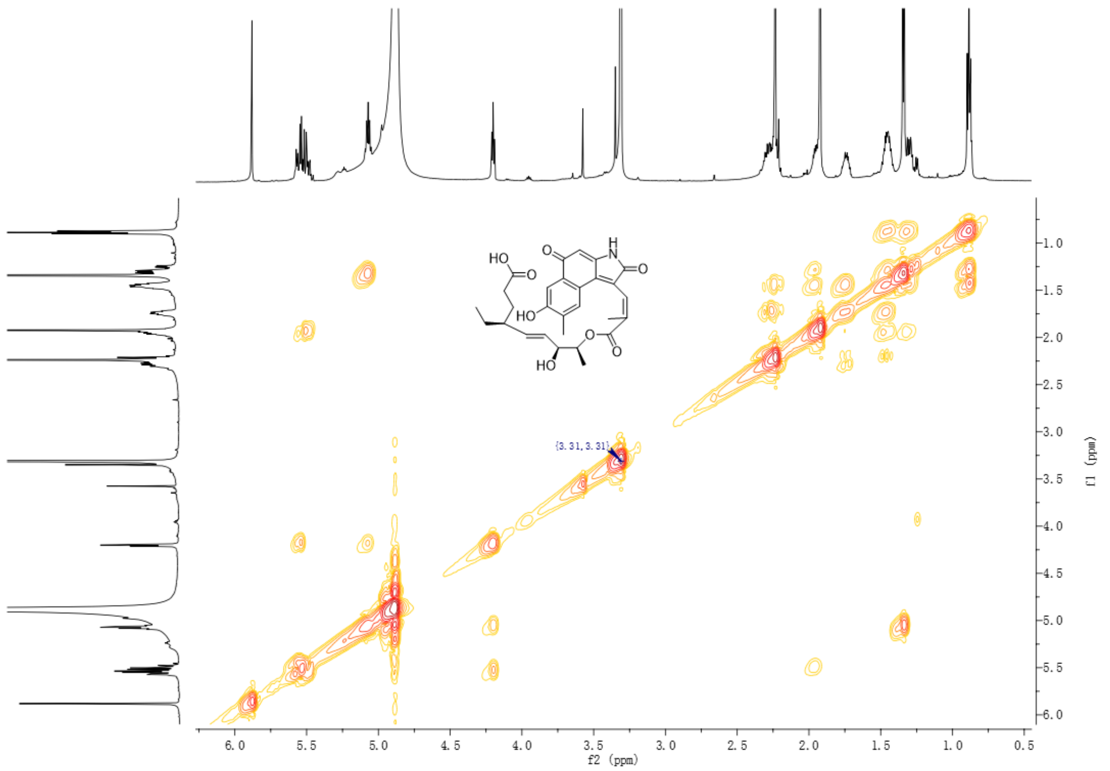

Figure S83. HMBC spectrum of hygrocin O (**9**)

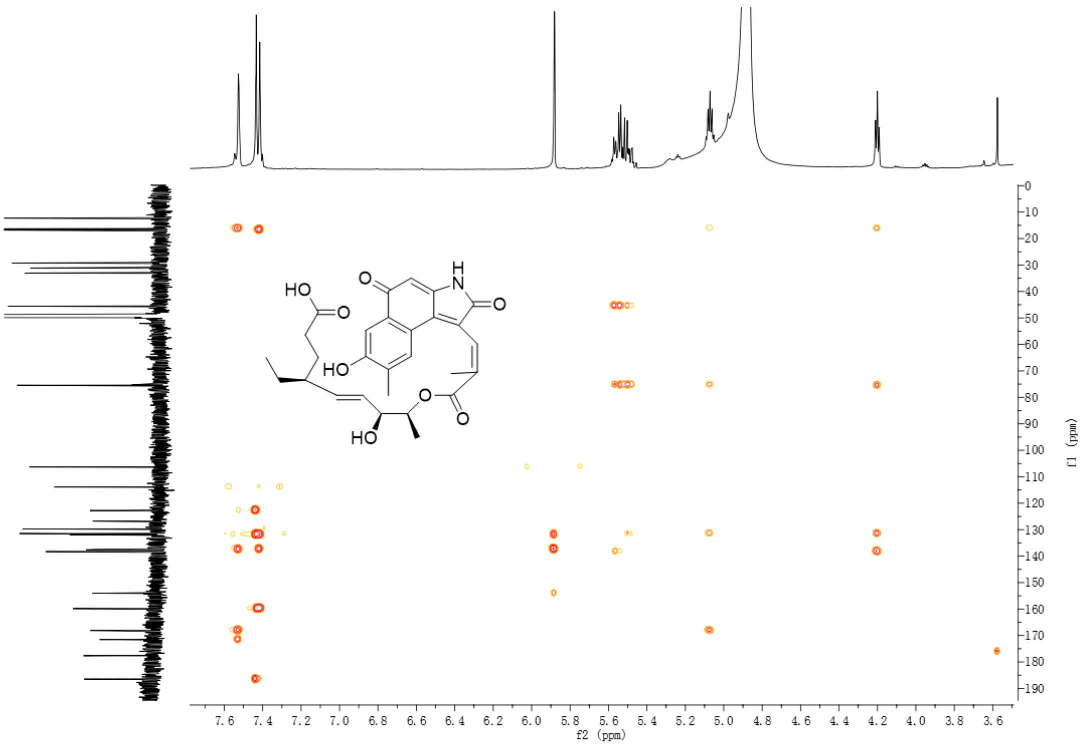

Figure S84. HMBC spectrum of hygrocin O (**9**)

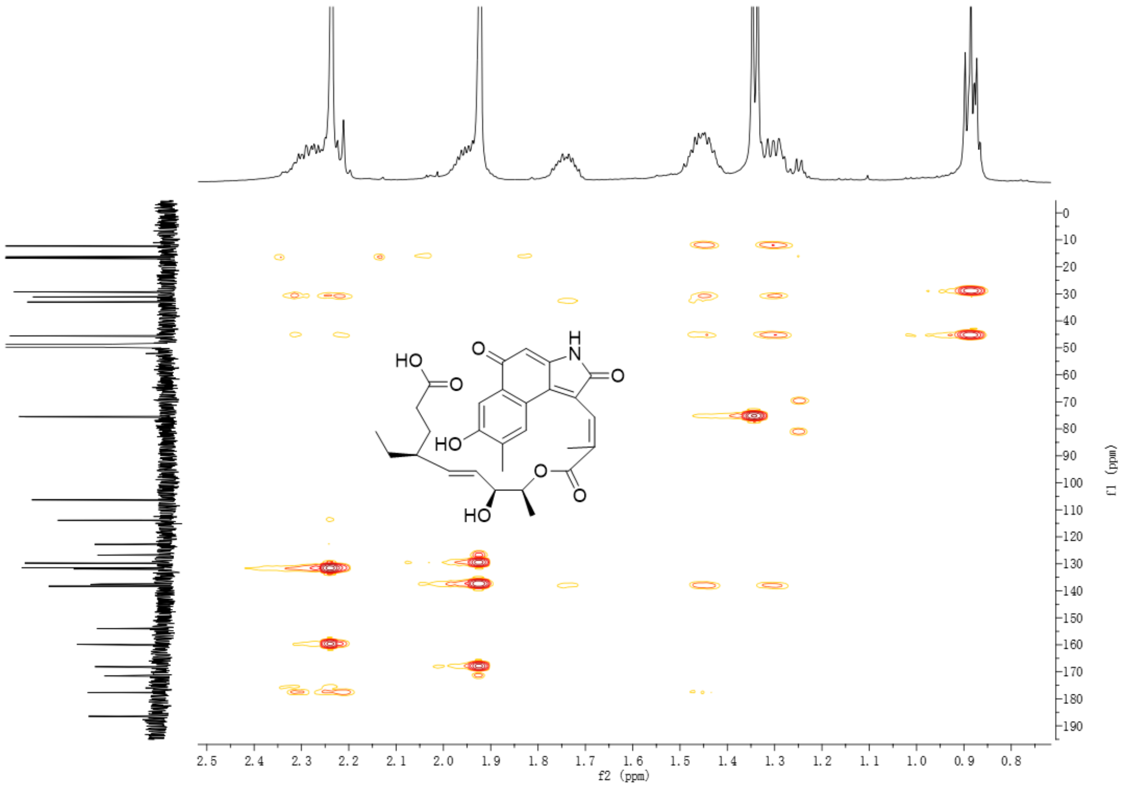

Figure S85. NOESY spectrum of hygrocin O (**9**)

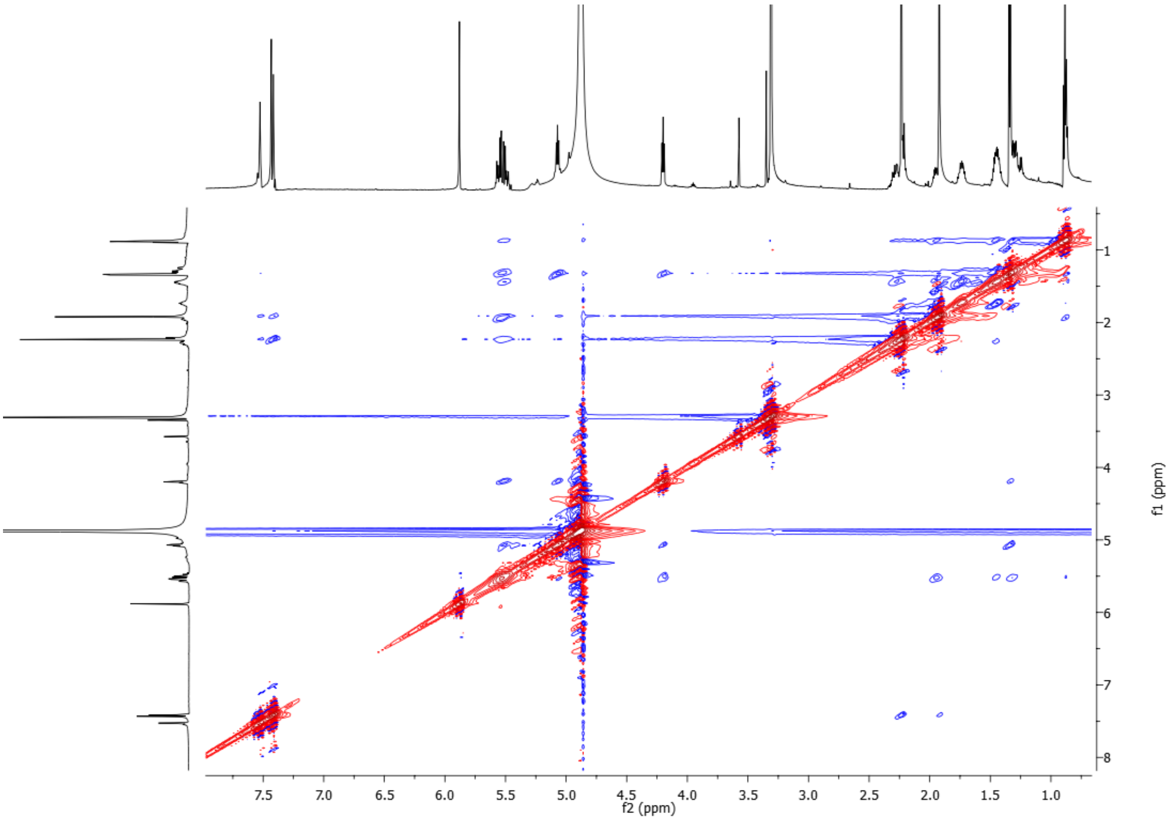

Figure S86. HRESIMS spectrum of hygrocin O (**9**)

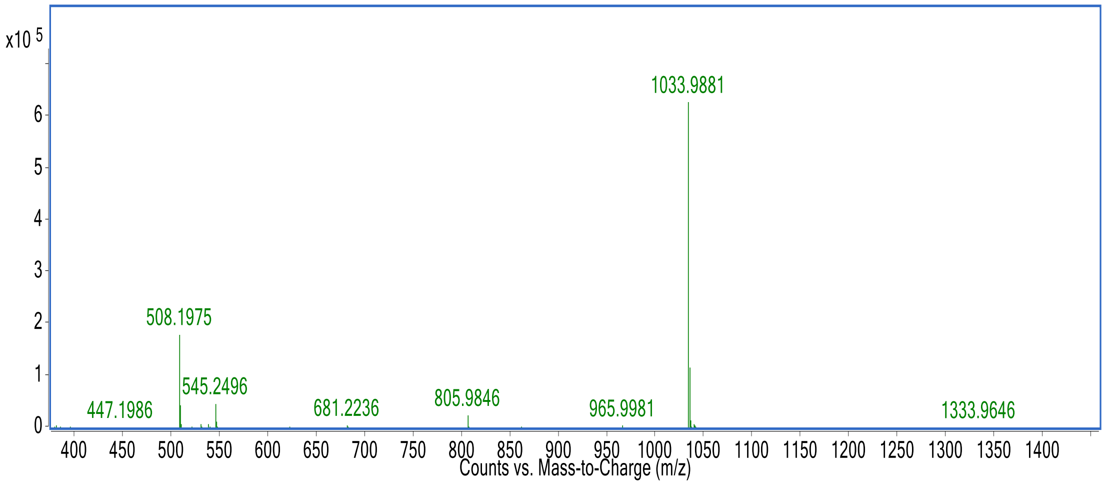

Figure S87. UV spectrum of hygrocin O (**9**)

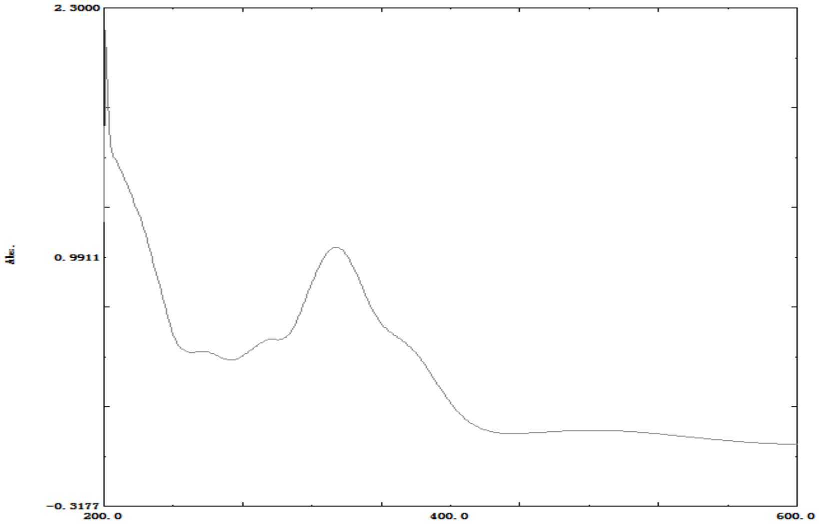

Figure S88. IR spectrum of hygrocin O (**9**)

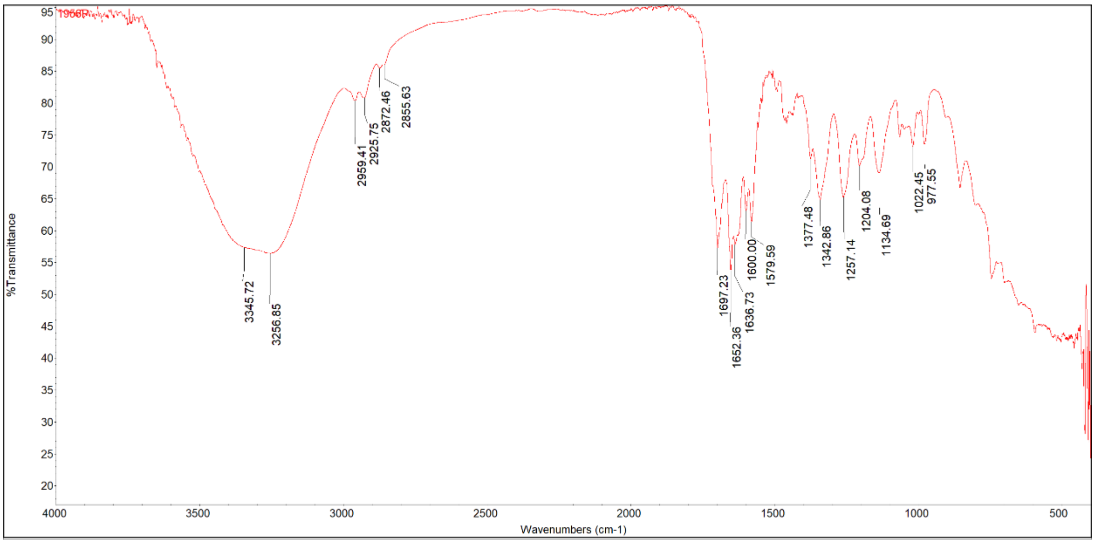

Figure S89. ^{1}H NMR spectrum of hygrocin P (**10**)

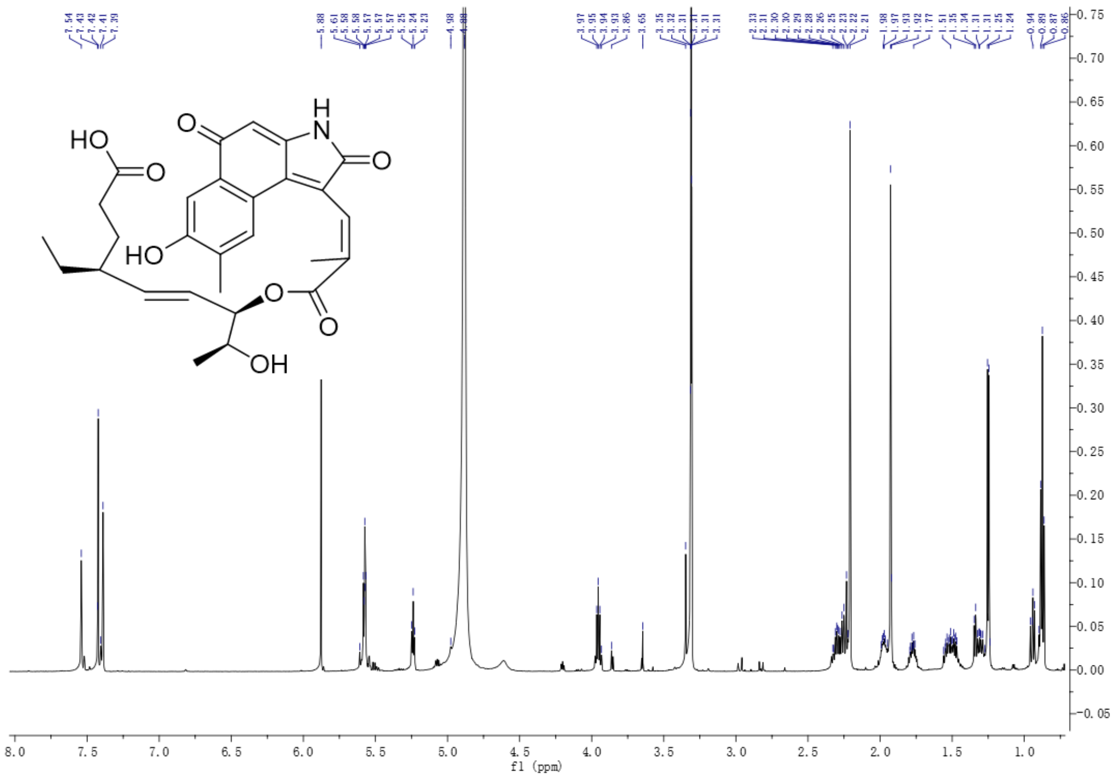

Figure S90. ^{1}H NMR spectrum of hygrocin P (**10**)

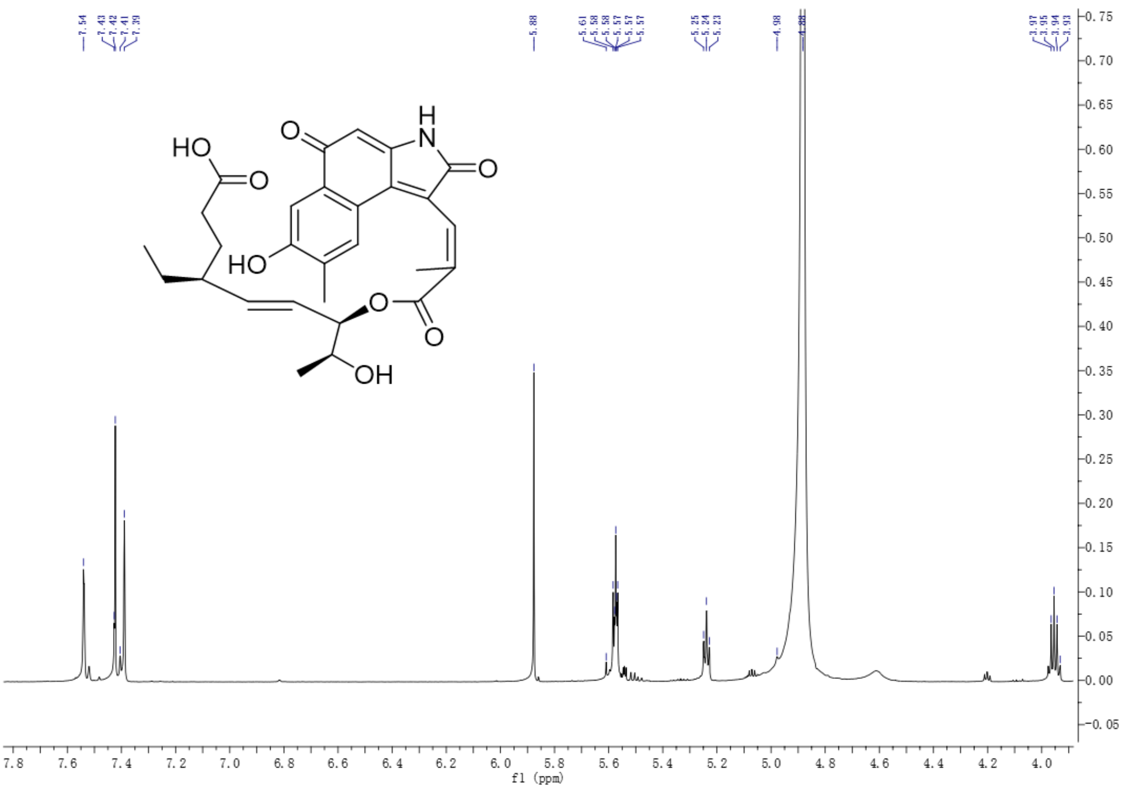

Figure S91. ^{1}H NMR spectrum of hygrocin P (**10**)

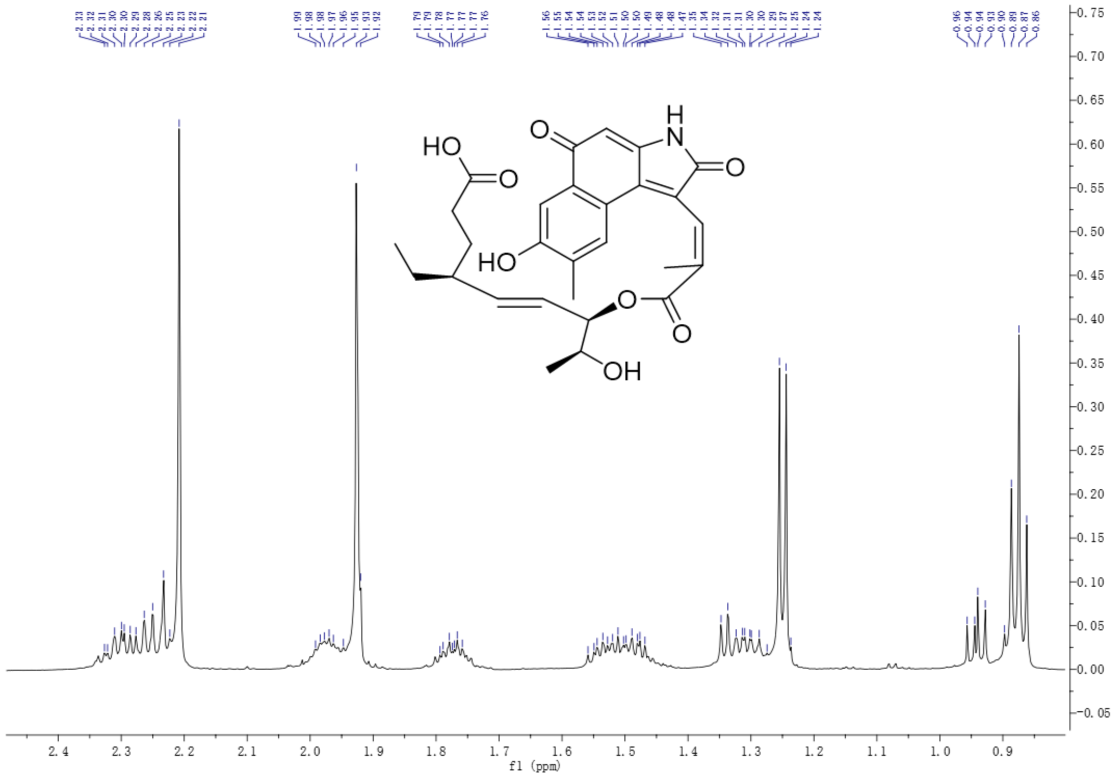

Figure S92. ^{13}C NMR spectrum of hygrocin P (**10**)

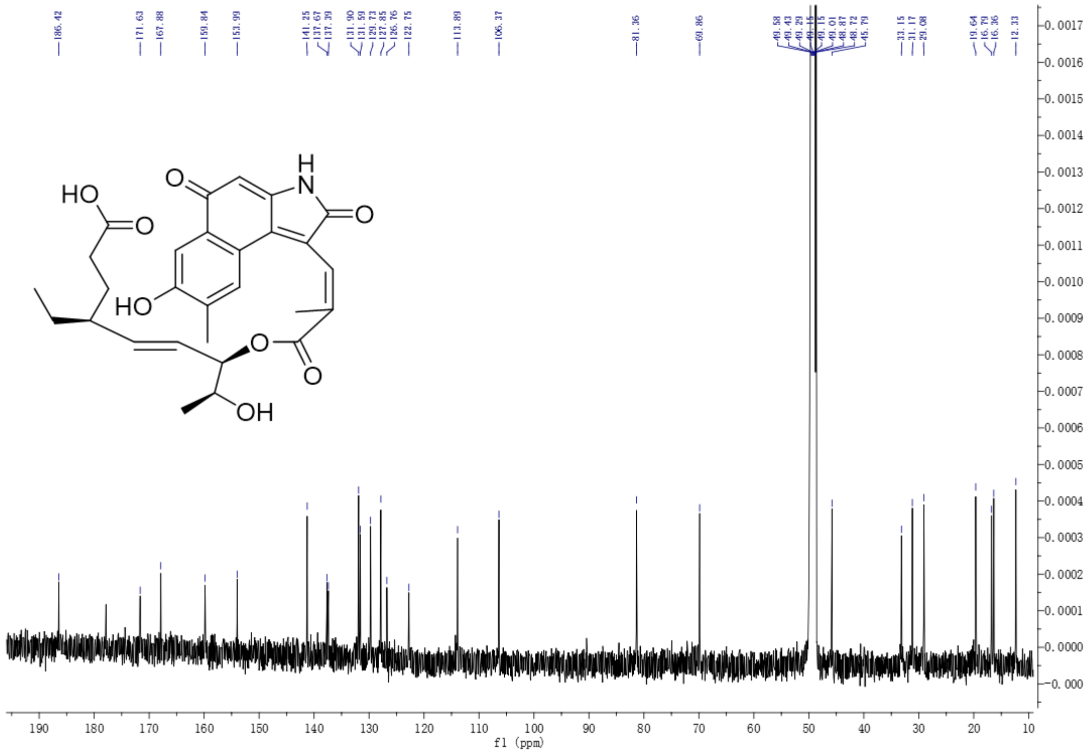

Figure S93. ^{13}C NMR spectrum of hygrocin P (**10**)

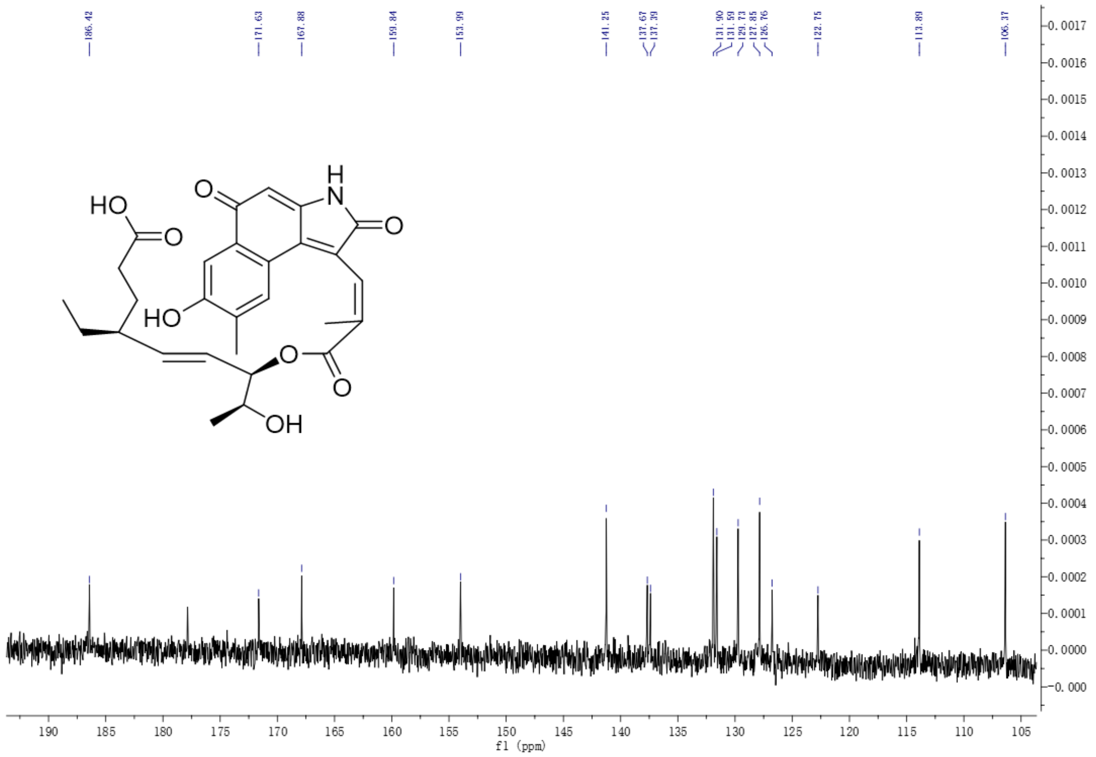

Figure S94. ^{13}C NMR spectrum of hygrocin P (**10**)

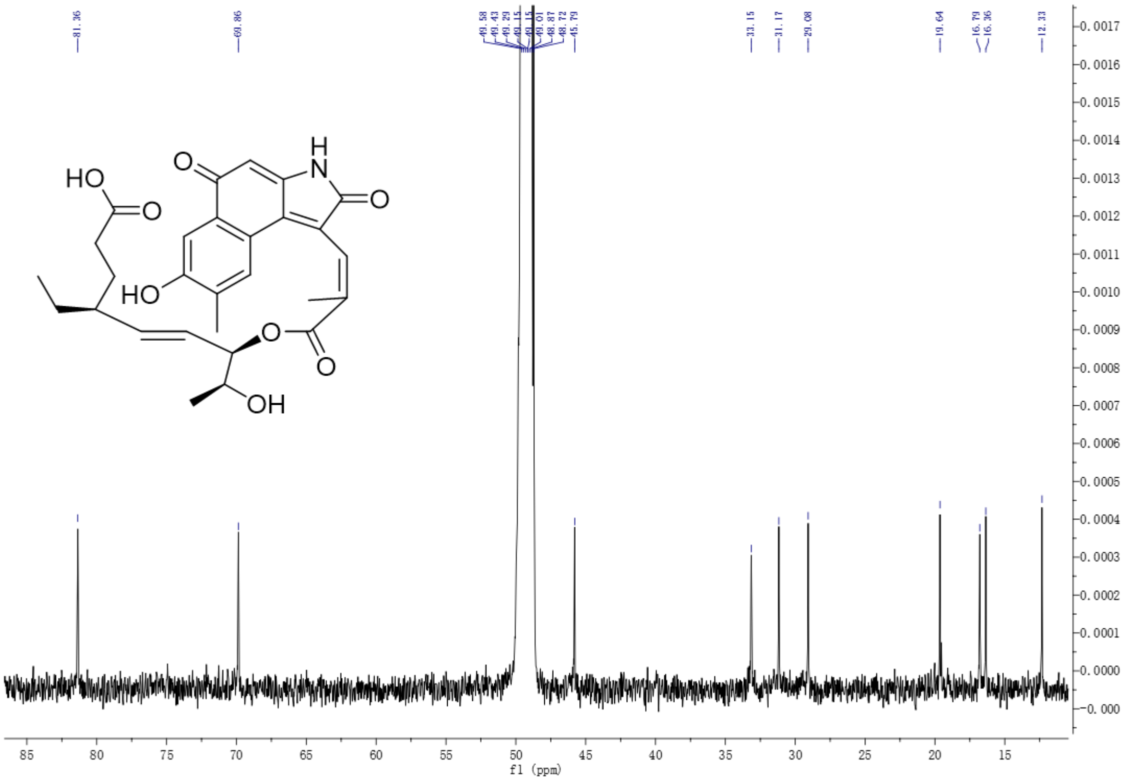

Figure S95. HMQC spectrum of hygrocin P (**10**)

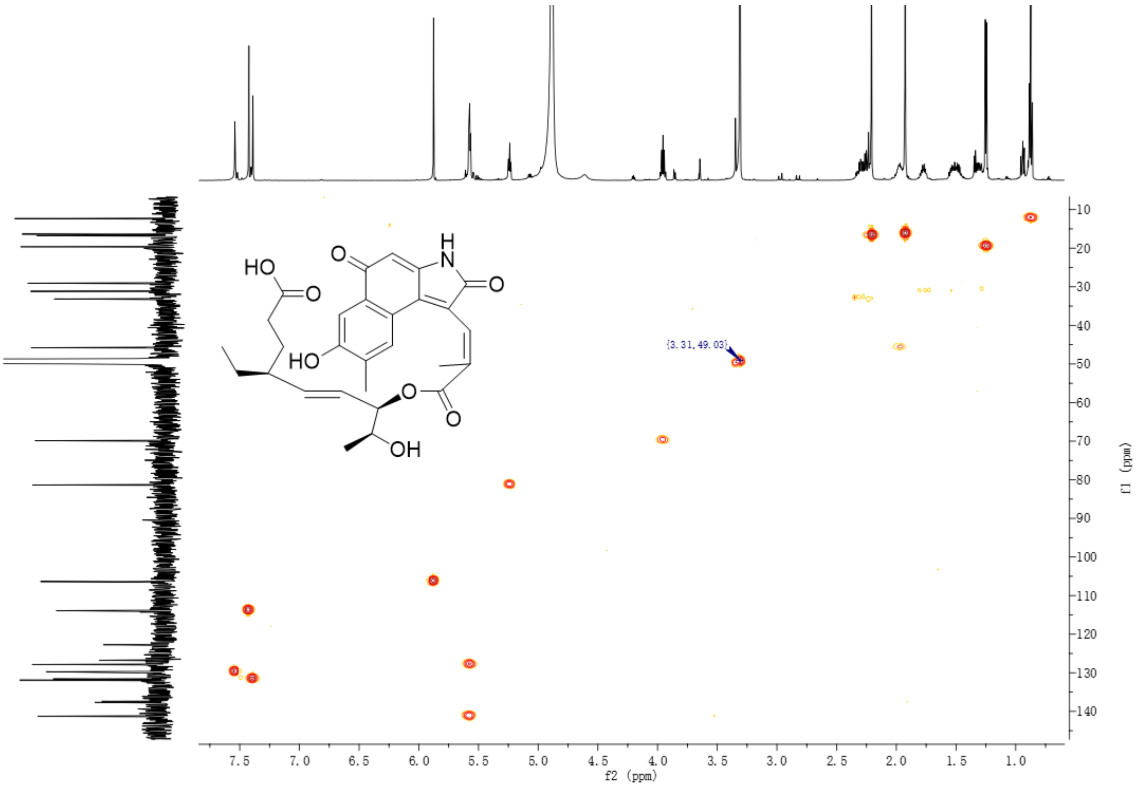

Figure S96. HMQC spectrum of hygrocin P (**10**)

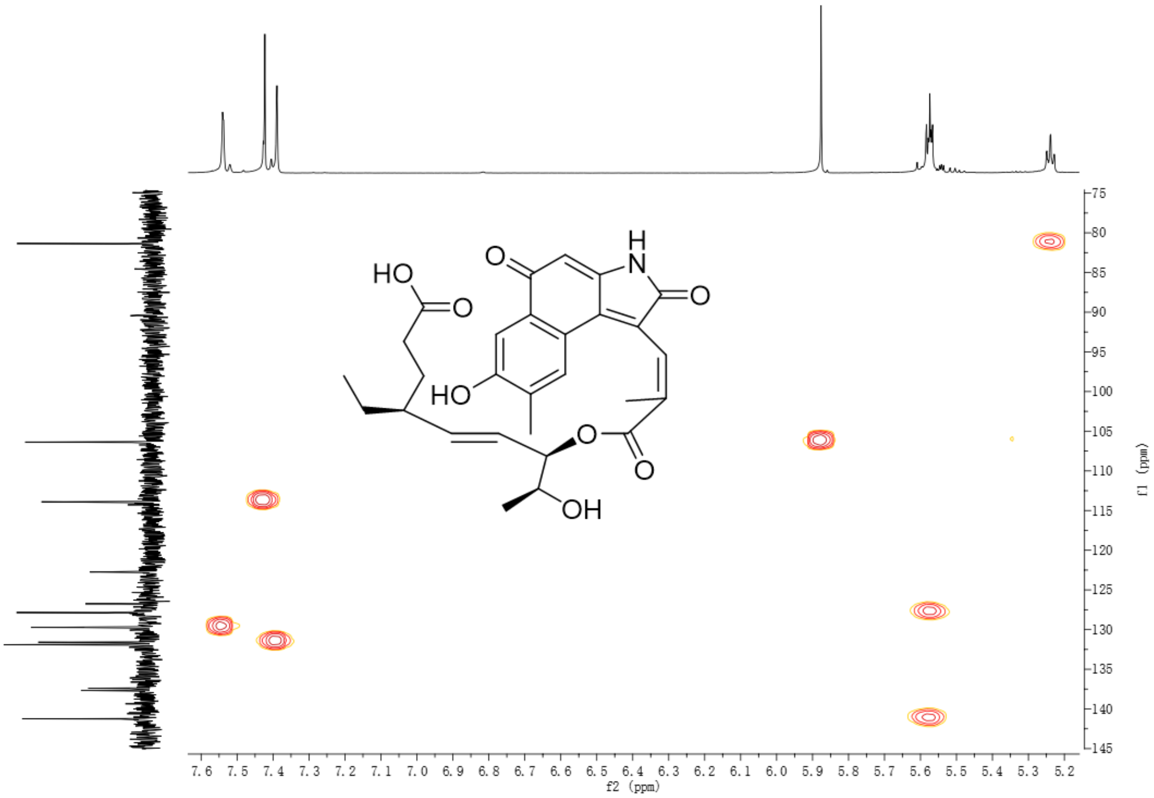

Figure S97. HMQC spectrum of hygrocin P (**10**)

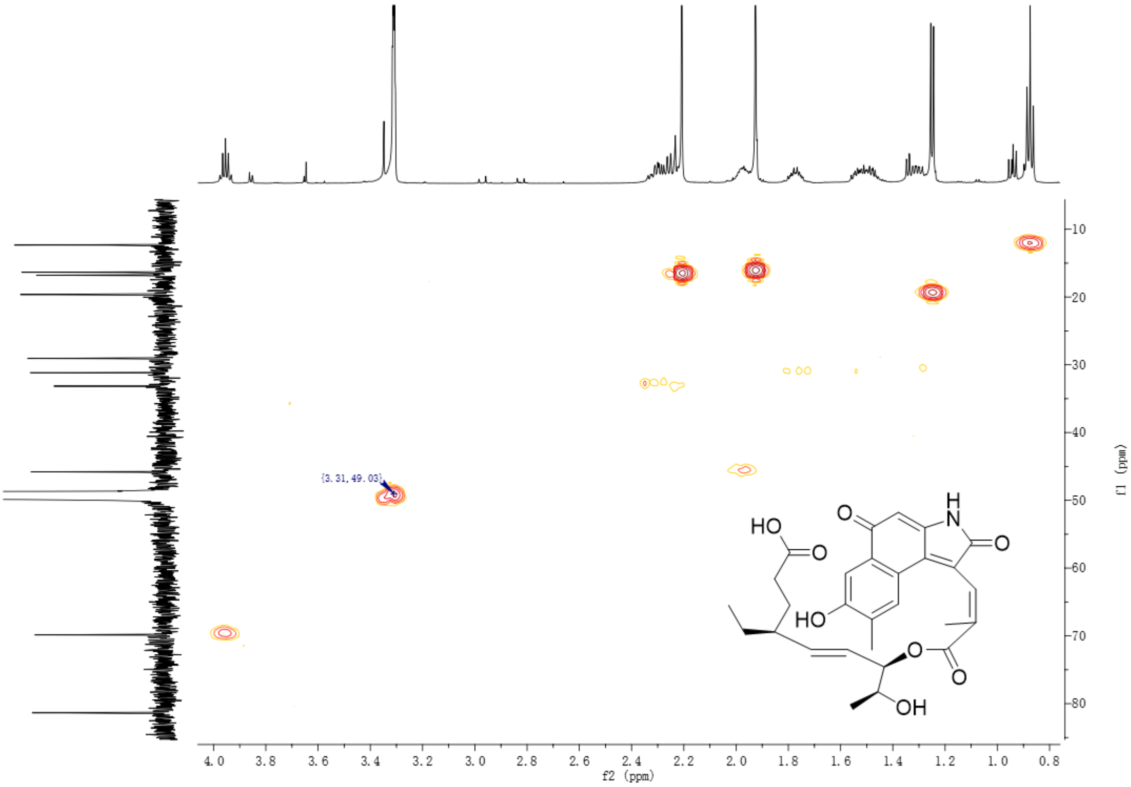

Figure S98. COSY spectrum of hygrocin P (**10**)

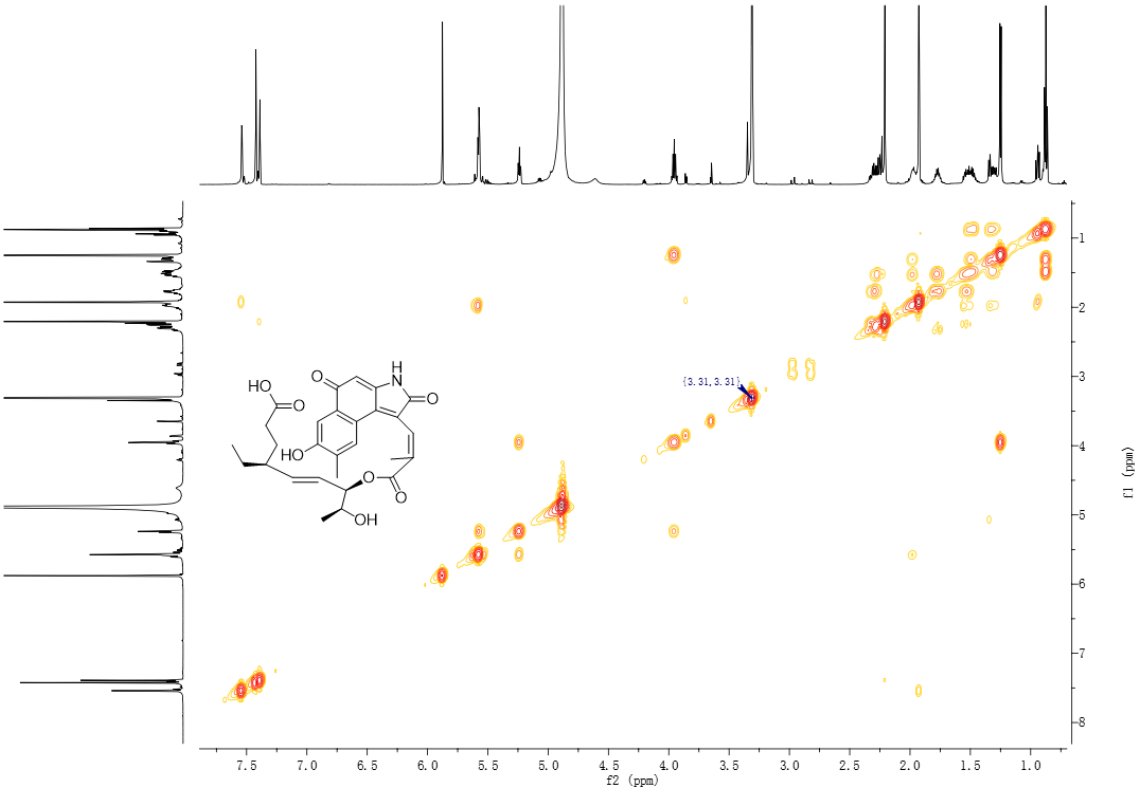

Figure S99. HMBC spectrum of hygrocin P (**10**)

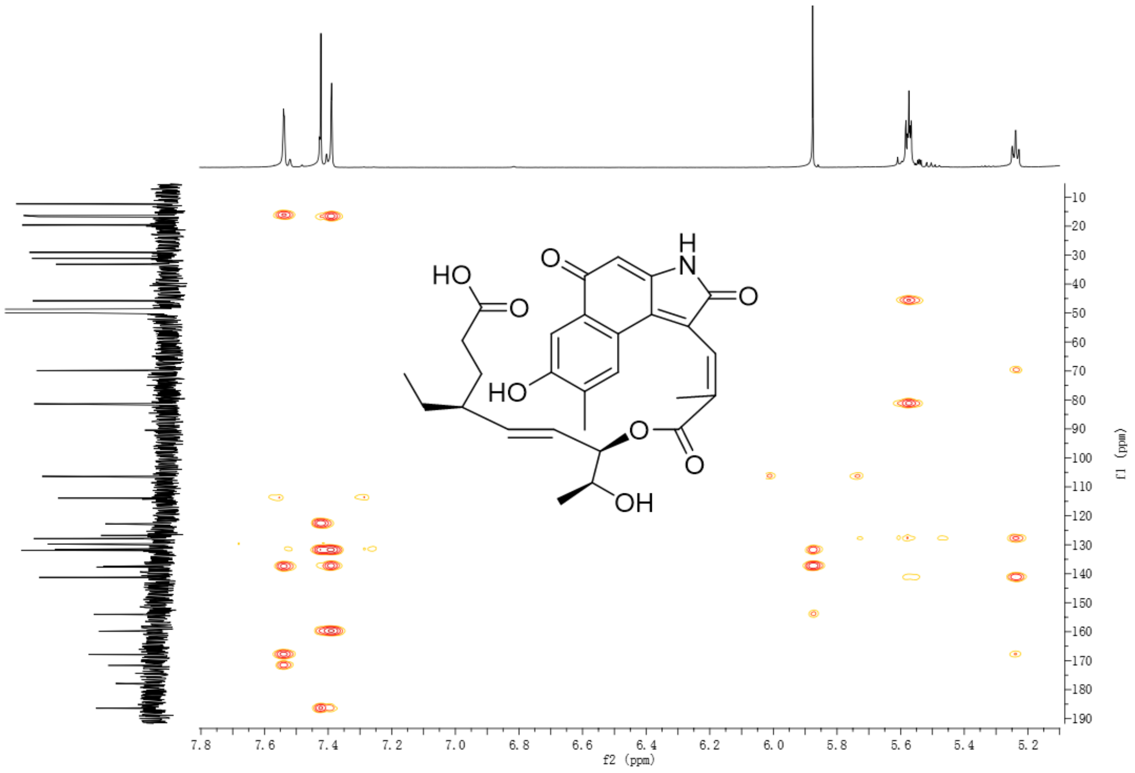

Figure S100. HMBC spectrum of hygrocin P (**10**)

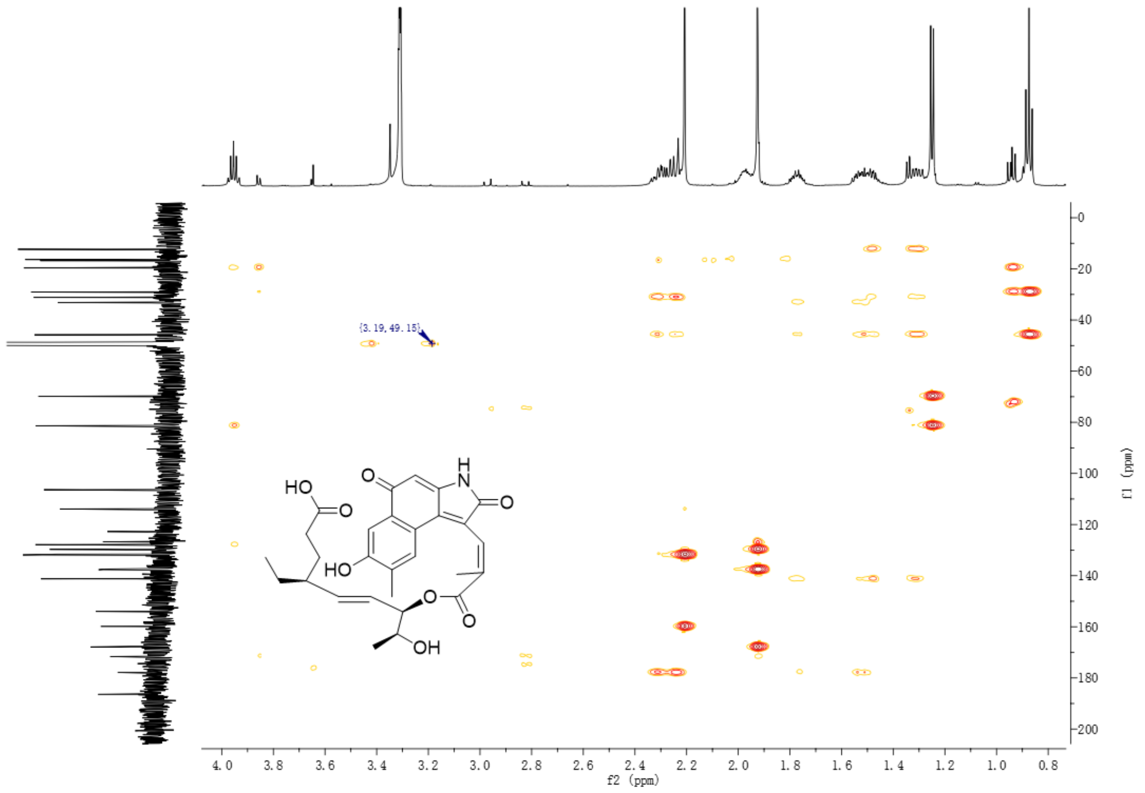

Figure S101. NOESY spectrum of hygrocin P (**10**)

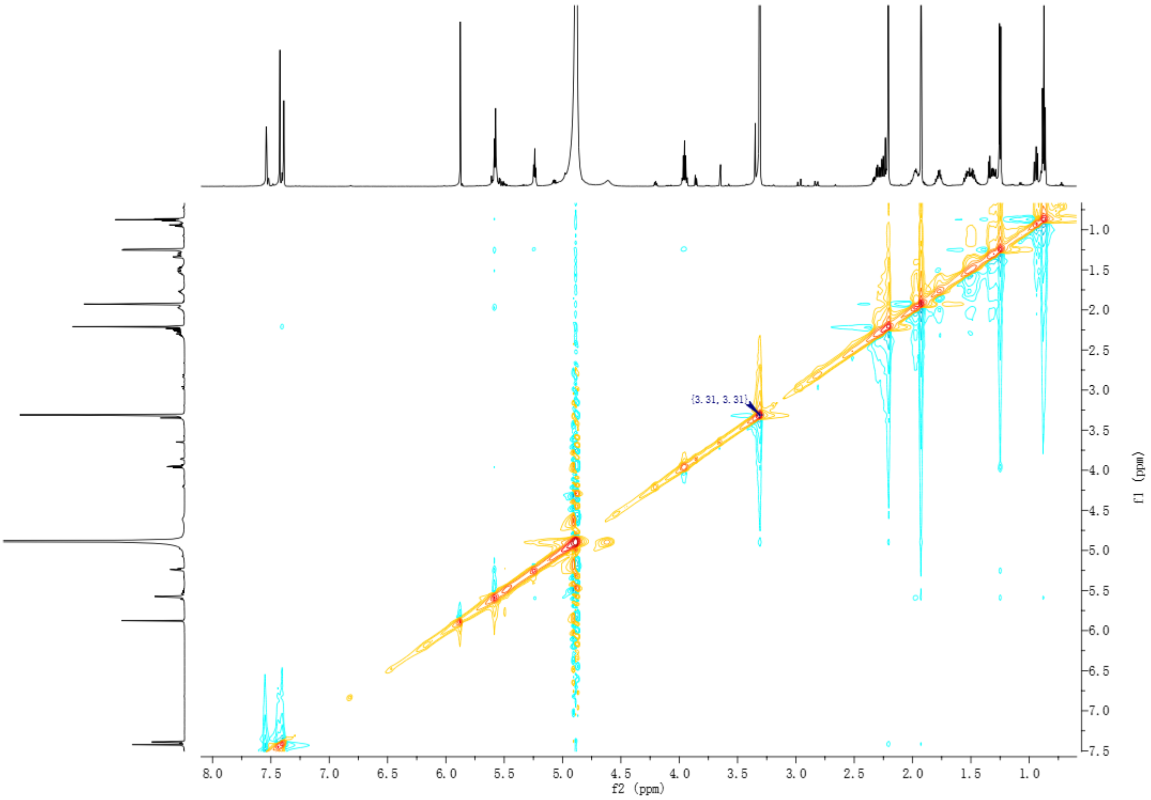

Figure S102. HRESIMS spectrum of hygrocin P (**10**)

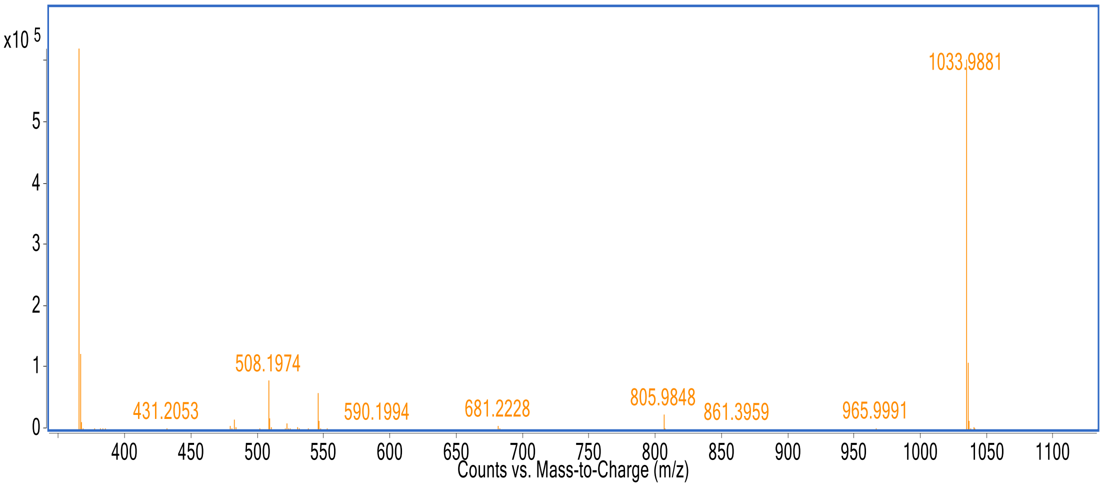

Figure S103. UV spectrum of hygrocin P (**10**)

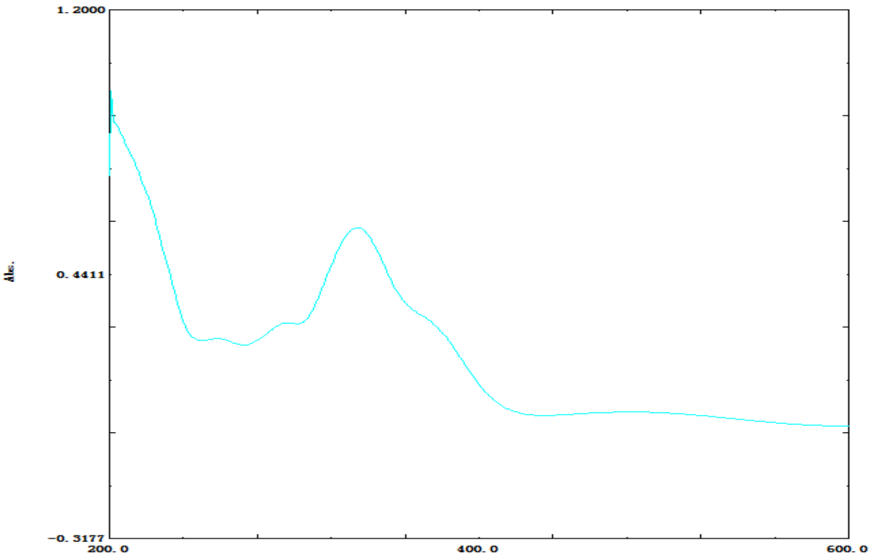

Figure S104. IR spectrum of hygrocin P (**10**)

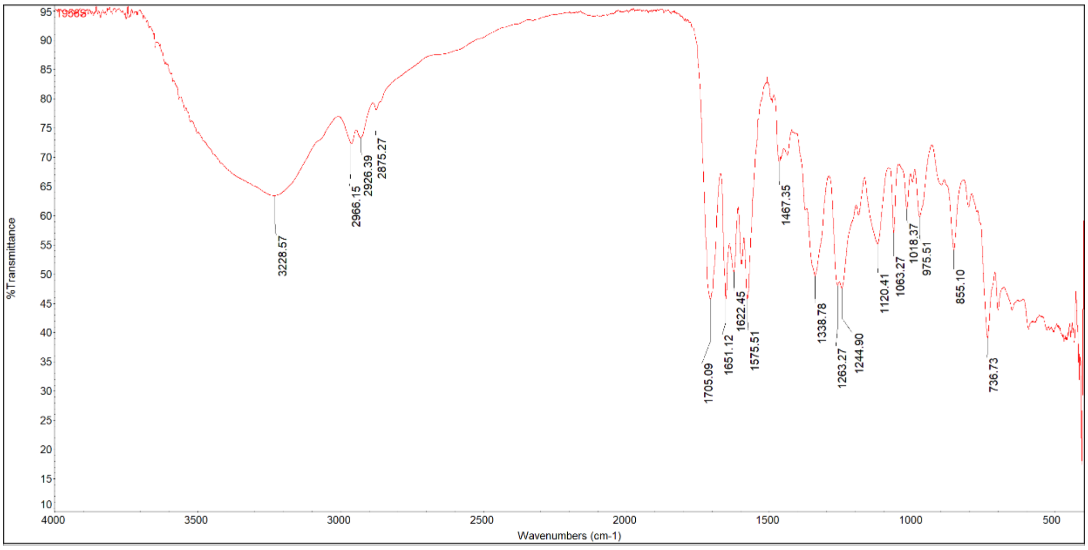

Figure S105. ^{1}H NMR spectrum of hygrocin Q (**11**)

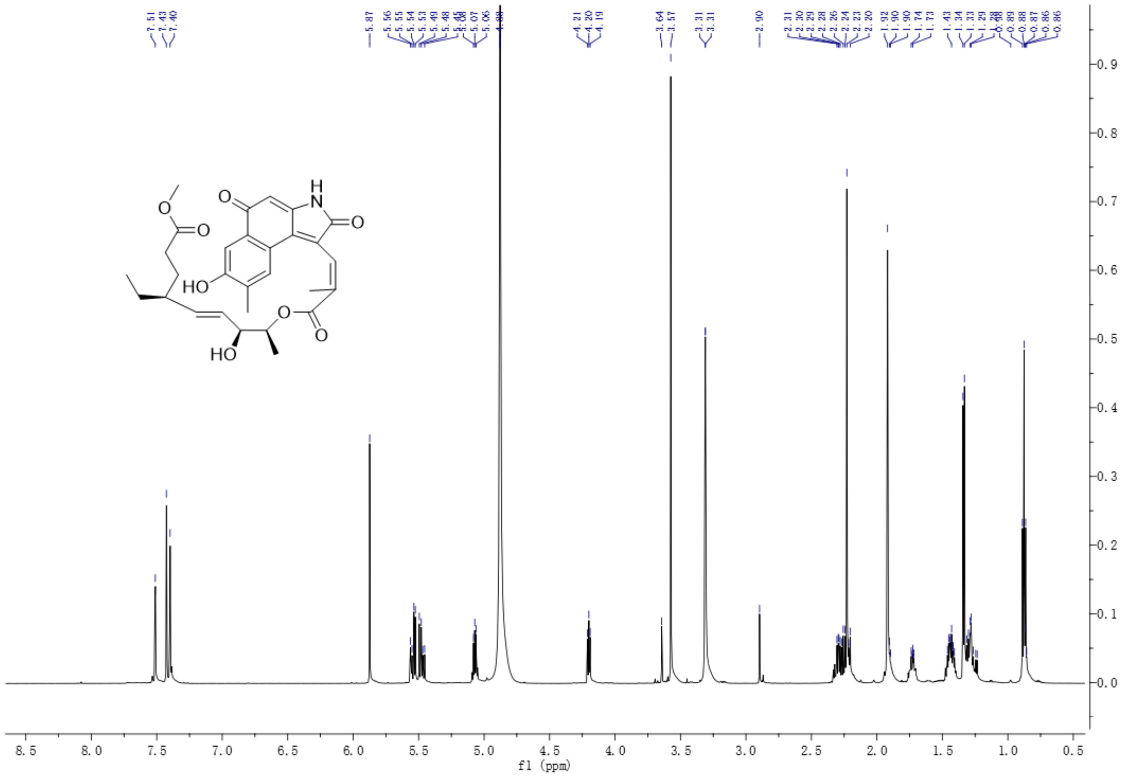

Figure S106. ^{1}H NMR spectrum of hygrocin Q (**11**)

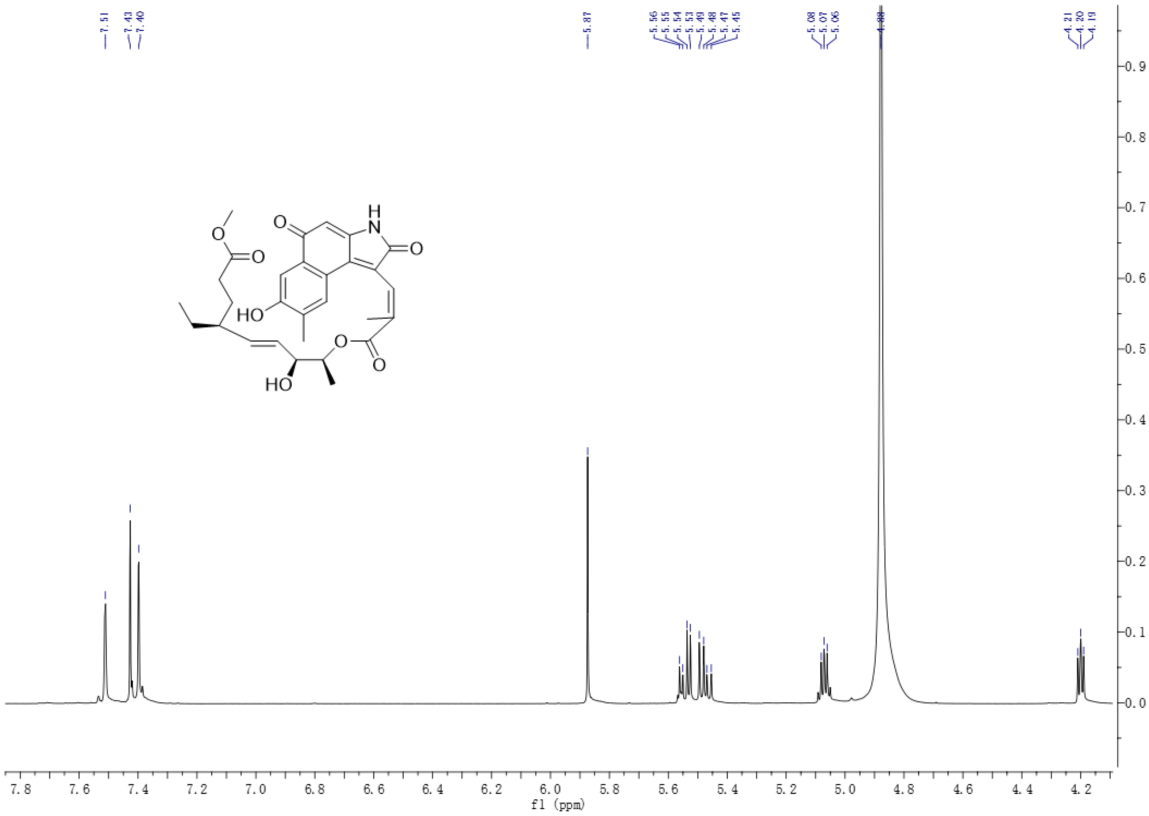

Figure S107. ^{1}H NMR spectrum of hygrocin Q (**11**)

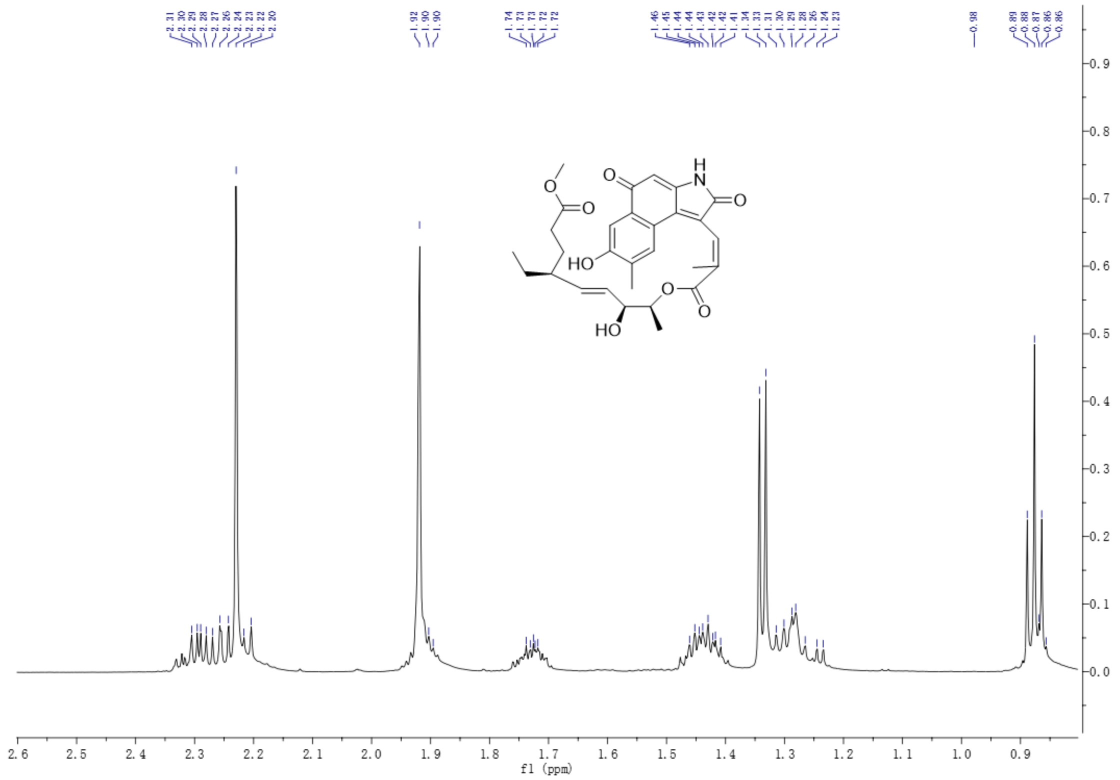

Figure S108. ^{13}C NMR spectrum of hygrocin Q (**11**)

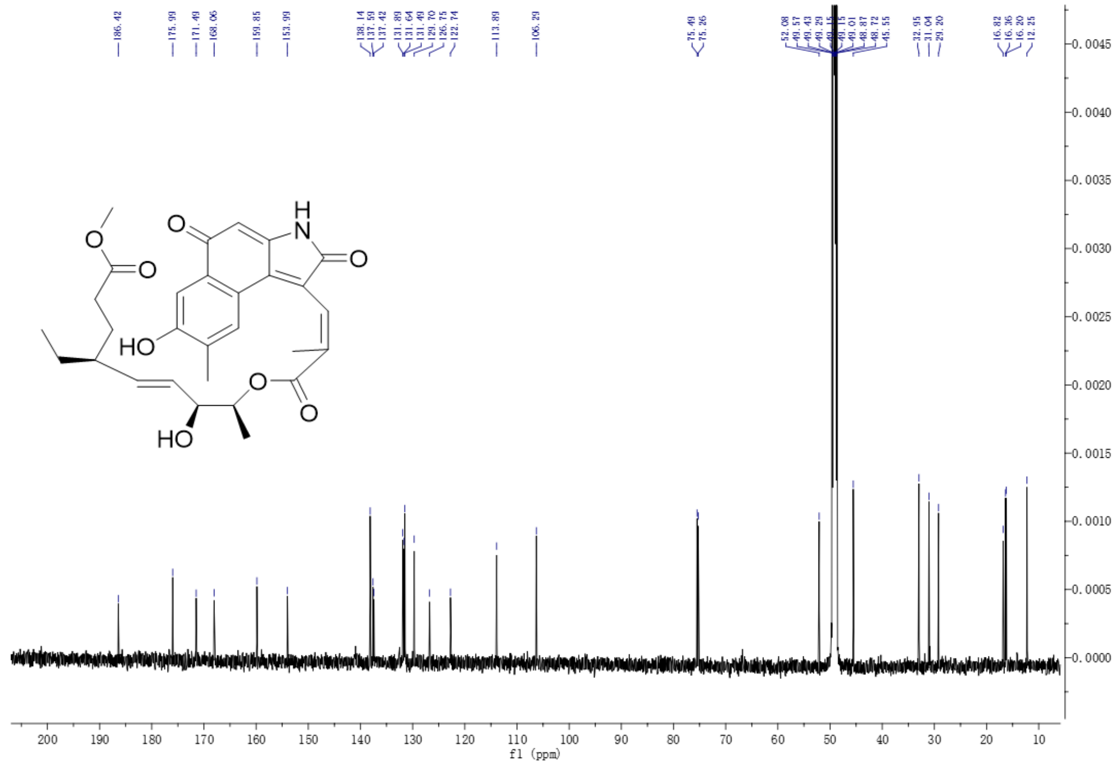

Figure S109. ^{13}C NMR spectrum of hygrocin Q (**11**)

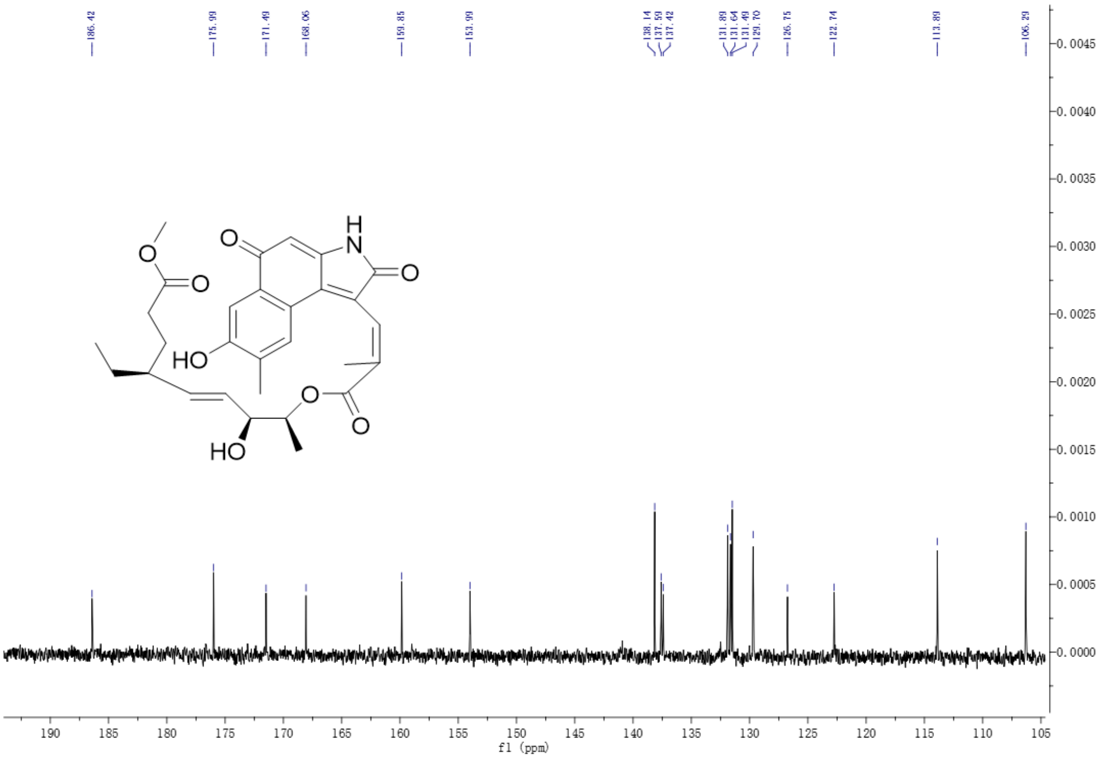

Figure S110. ^{13}C NMR spectrum of hygrocin Q (**11**)

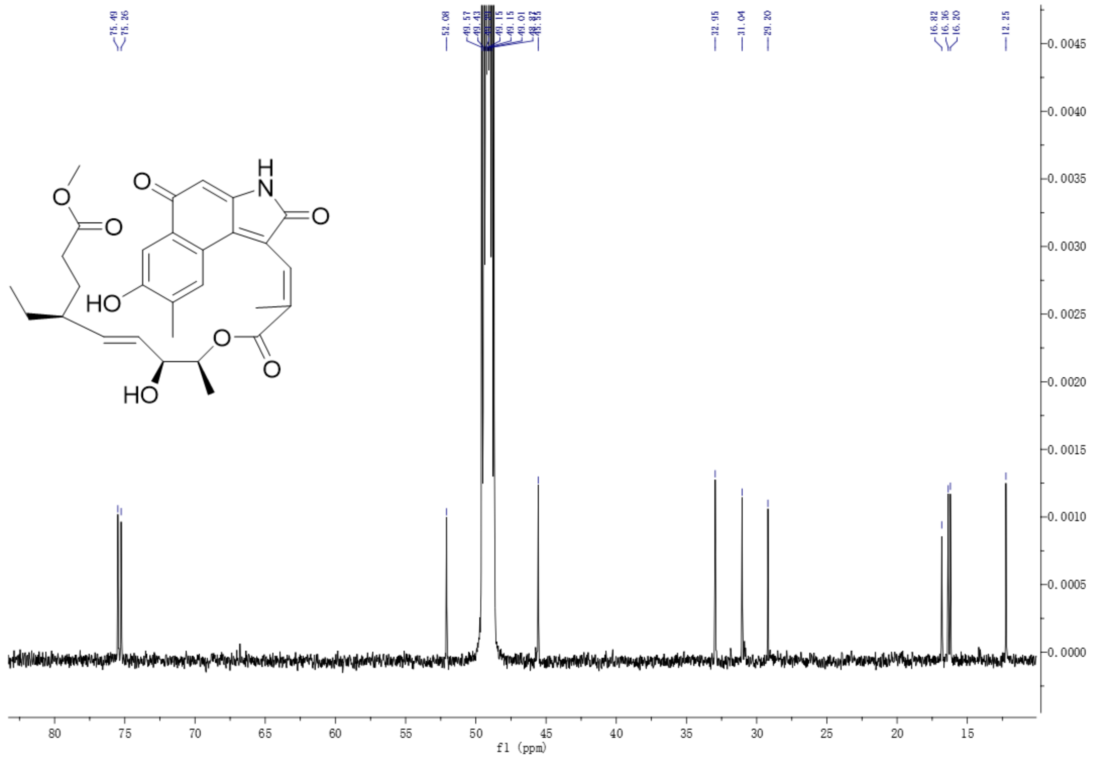

Figure S111. HMQC spectrum of hygrocin Q (**11**)

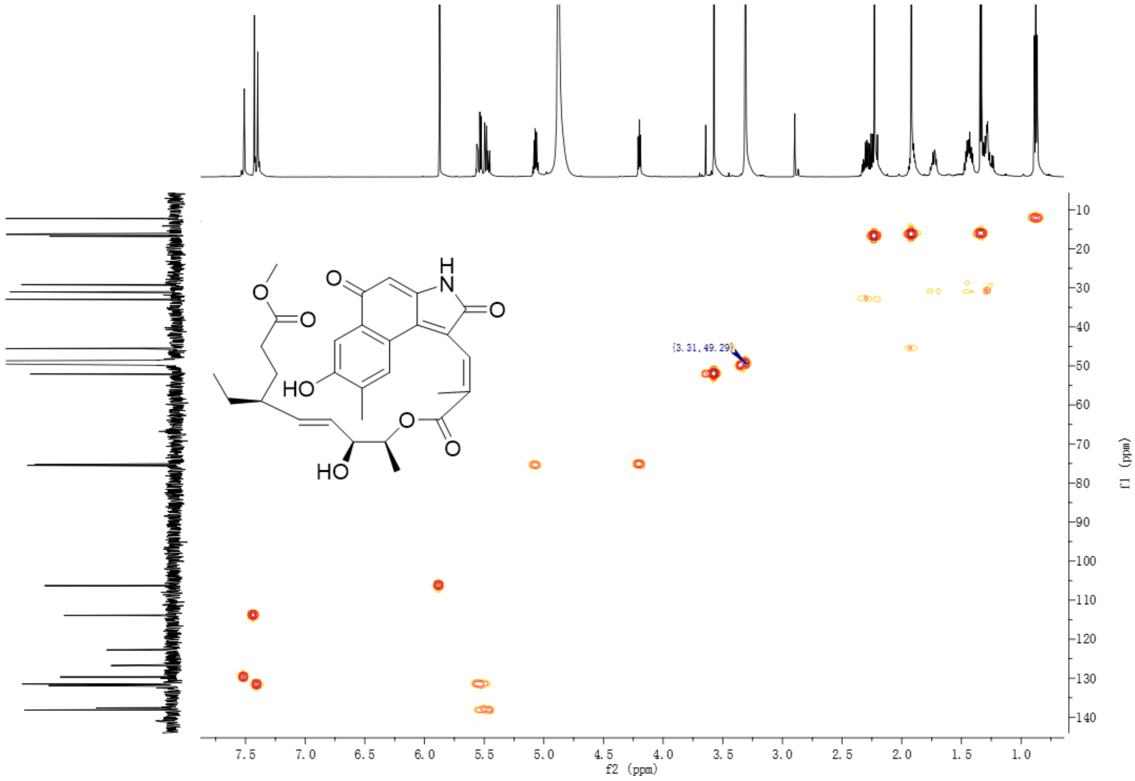

Figure S112. COSY spectrum of hygrocin Q (**11**)

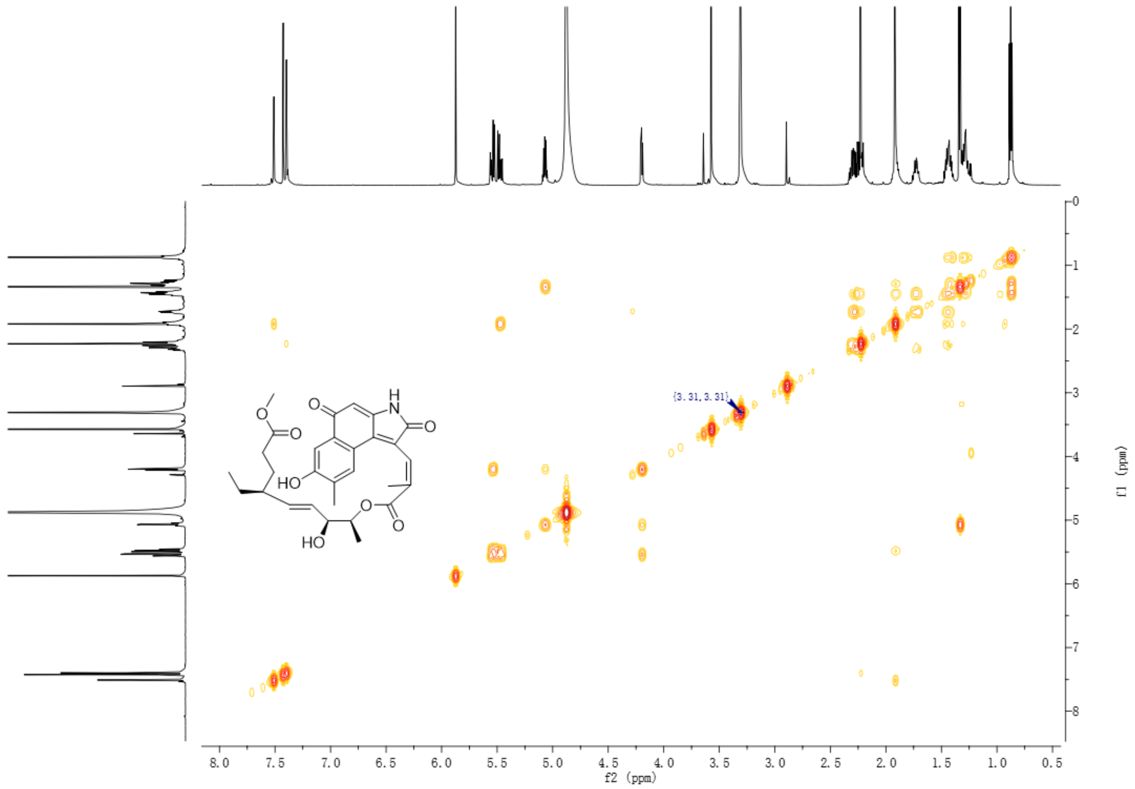

Figure S113. HMBC spectrum of hygrocin Q (**11**)

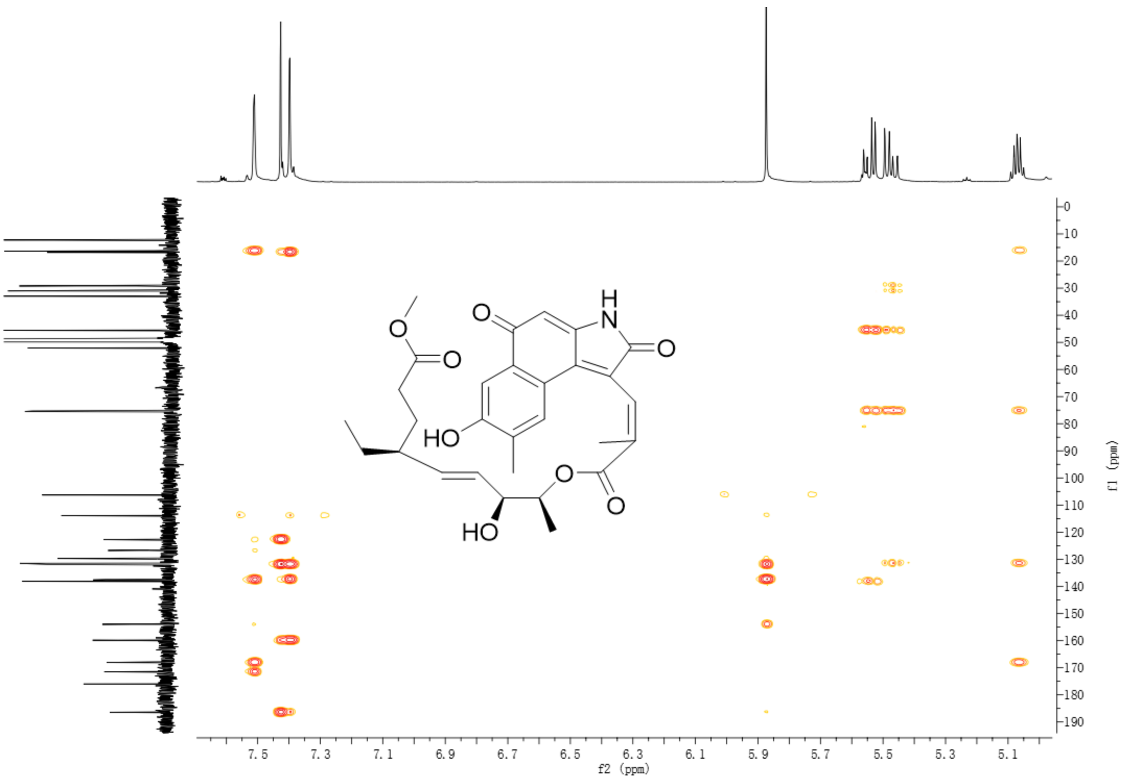

Figure S114. HMBC spectrum of hygrocin Q (**11**)

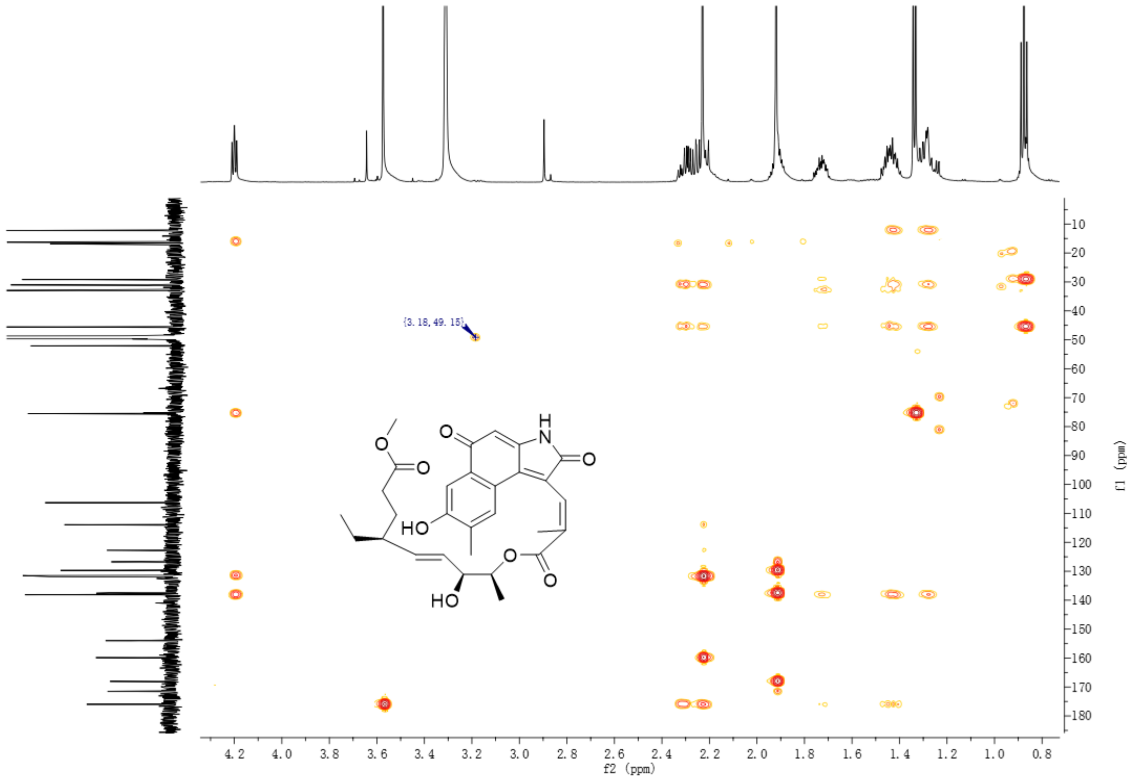

Figure S115. NOESY spectrum of hygrocin Q (**11**)

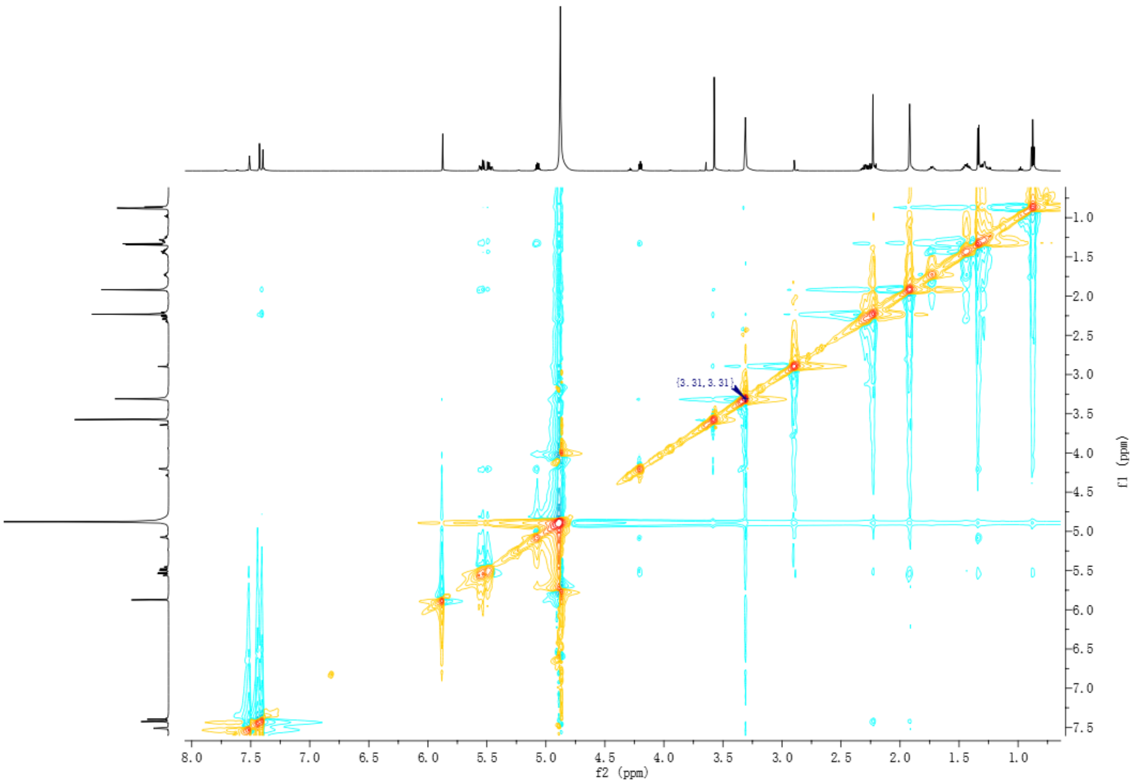

Figure S116. HRESIMS spectrum of hygrocin Q (**11**)

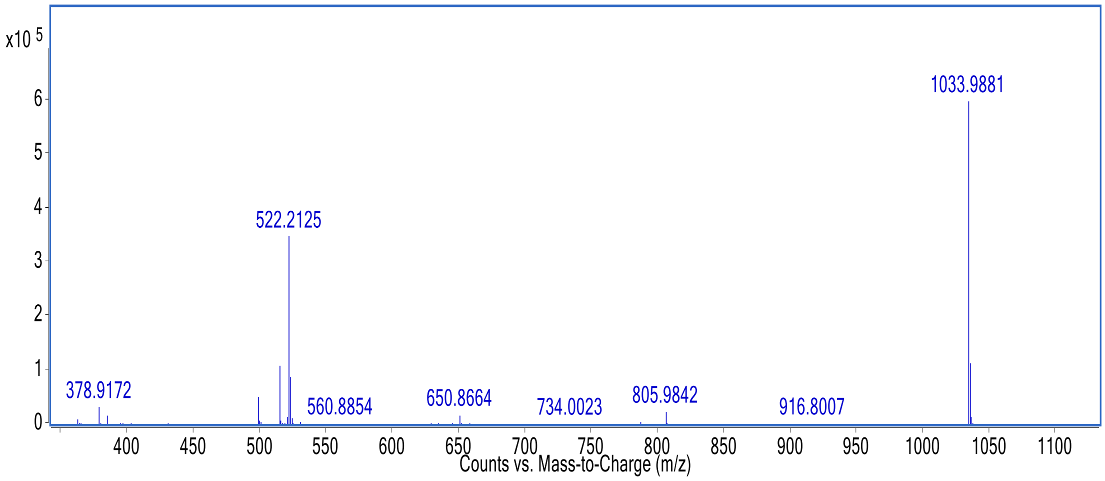

Figure S117. UV spectrum of hygrocin Q (**11**)

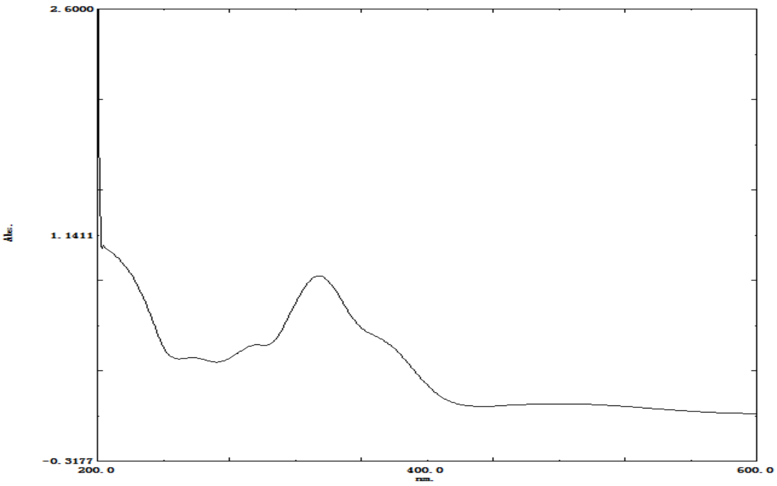

Figure S118. IR spectrum of hygrocin Q (**11**)

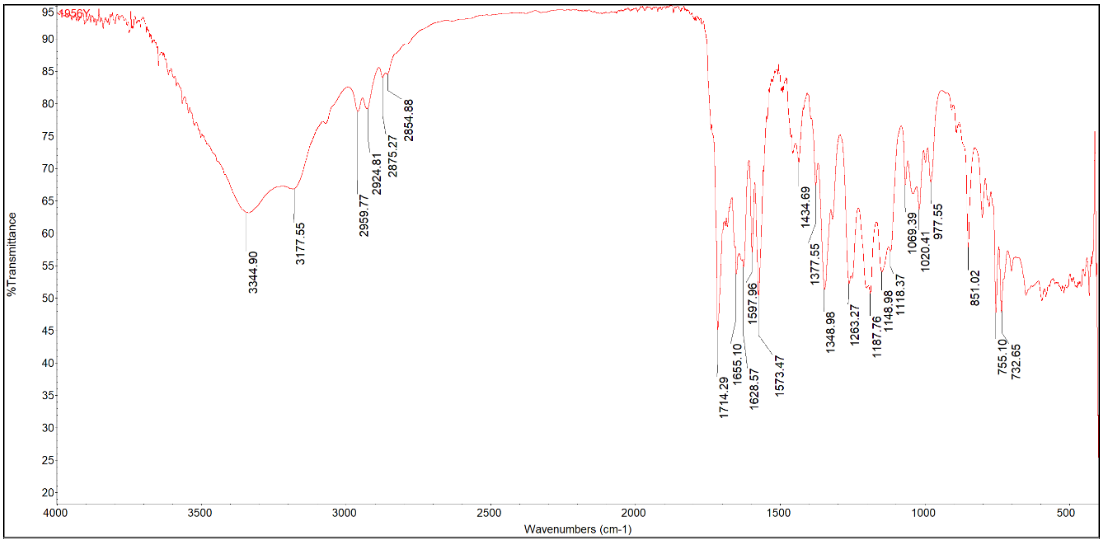

Figure S119. ¹H NMR spectrum of hygrocin R (**12**)

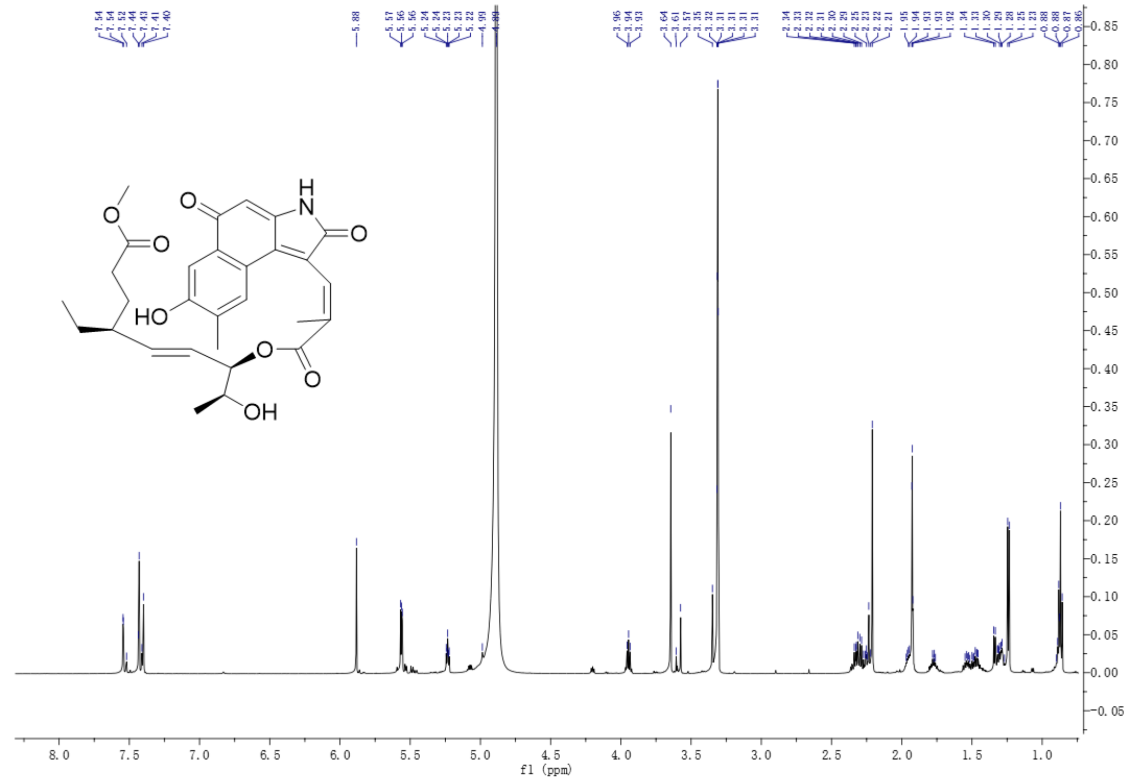

Figure S120. ¹H NMR spectrum of hygrocin R (**12**)

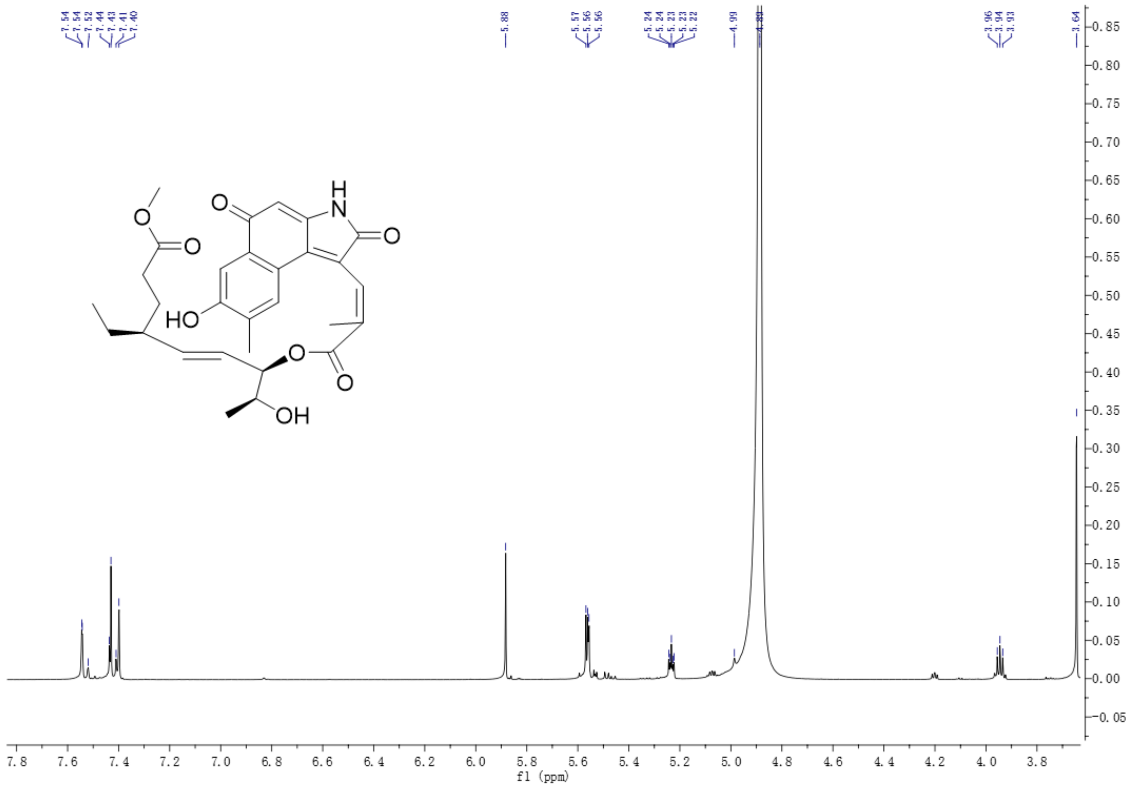

Figure S121. ^{1}H NMR spectrum of hygrocin R (**12**)

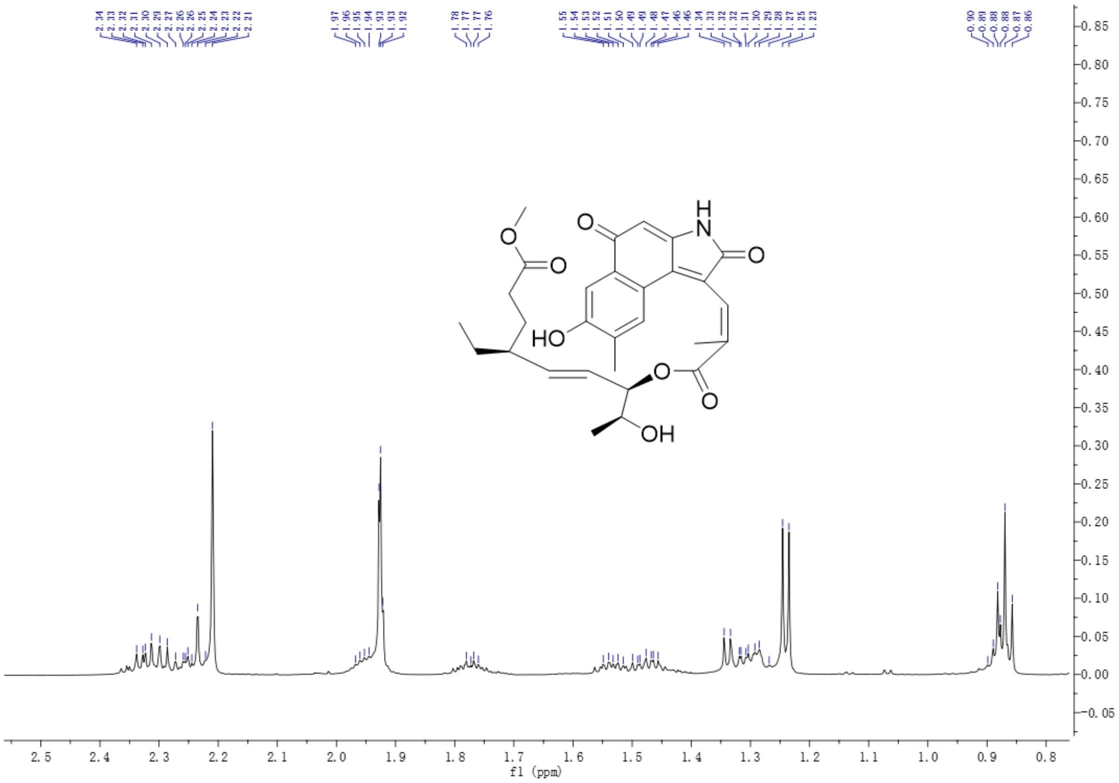

Figure S122. ^{13}C NMR spectrum of hygrocin R (**12**)

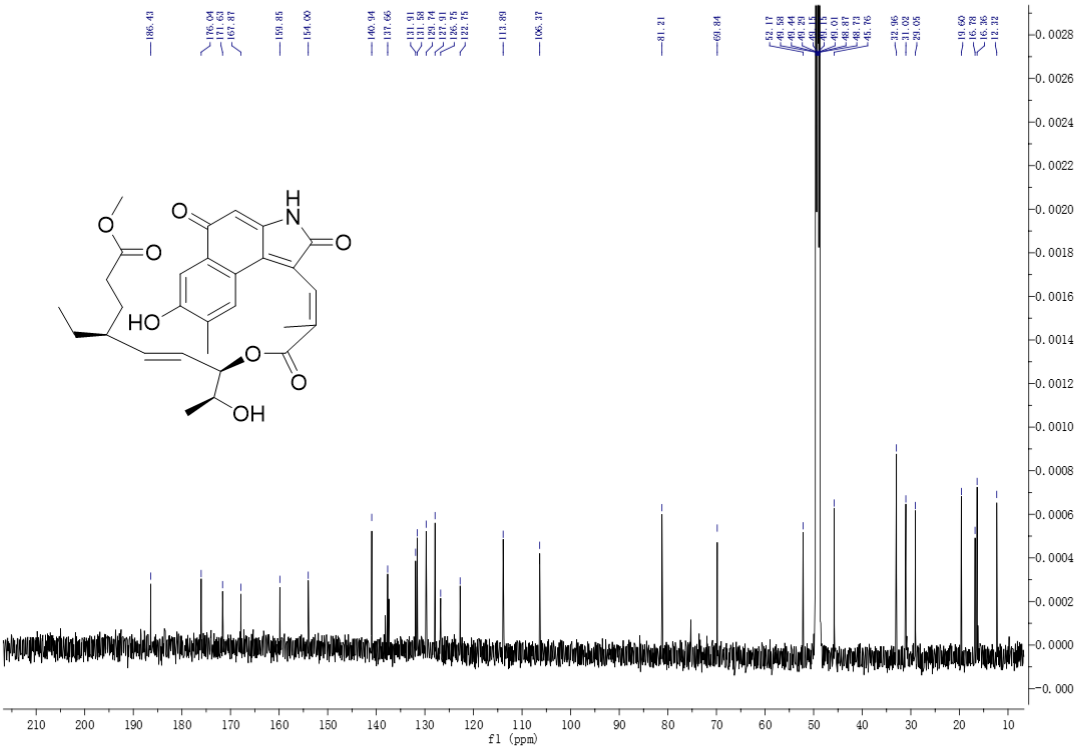

Figure S123. ^{13}C NMR spectrum of hygrocin R (**12**)

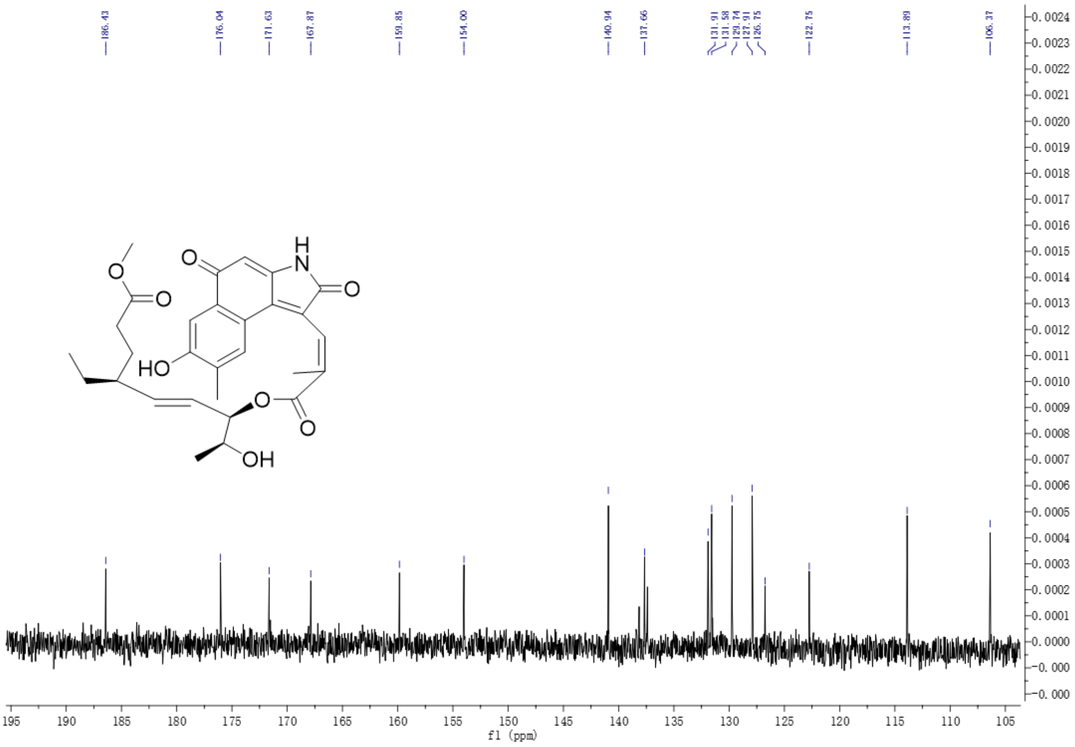

Figure S124. ^{13}C NMR spectrum of hygrocin R (**12**)

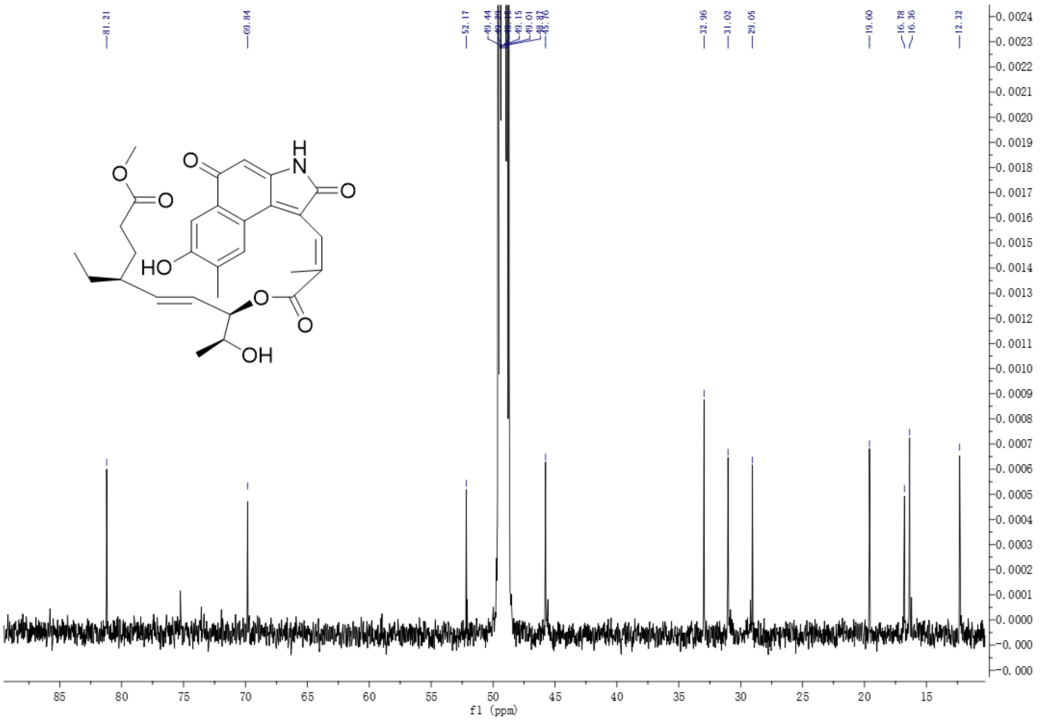

Figure S125. HMQC spectrum of hygrocin R (**12**)

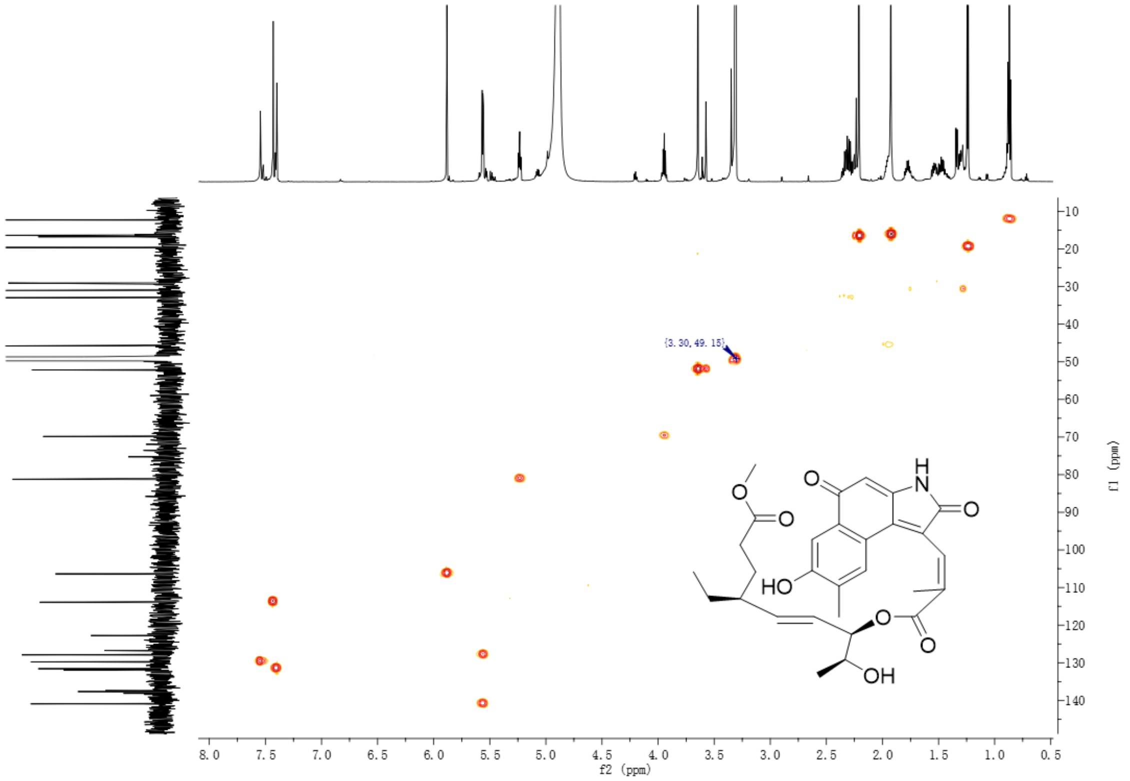

Figure S126. COSY spectrum of hygrocin R (**12**)

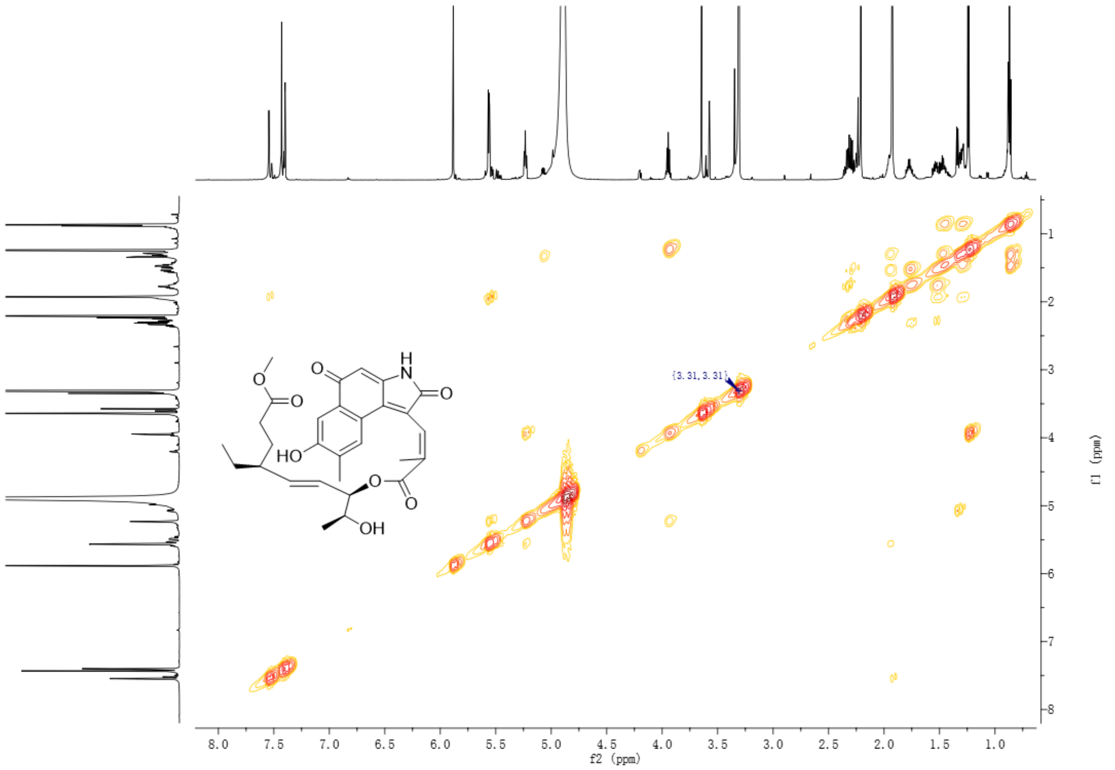

Figure S127. HMBC spectrum of hygrocin R (**12**)

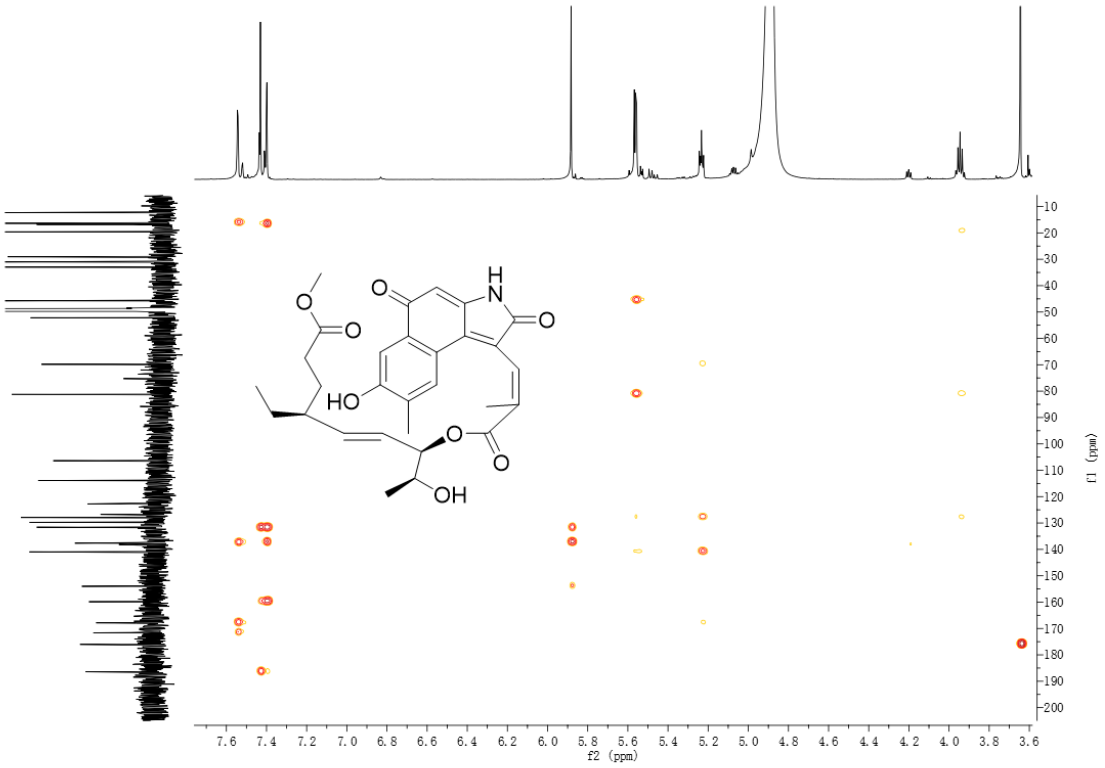

Figure S128. HMBC spectrum of hygrocin R (**12**)

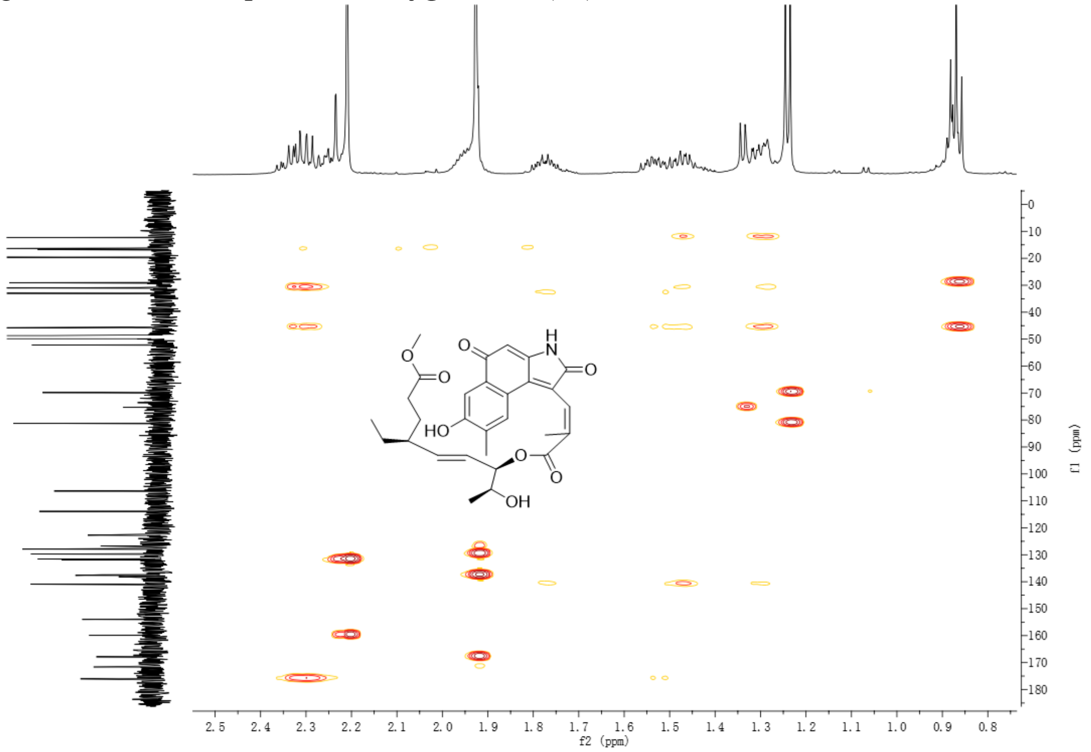

Figure S129. NOESY spectrum of hygrocin R (**12**)

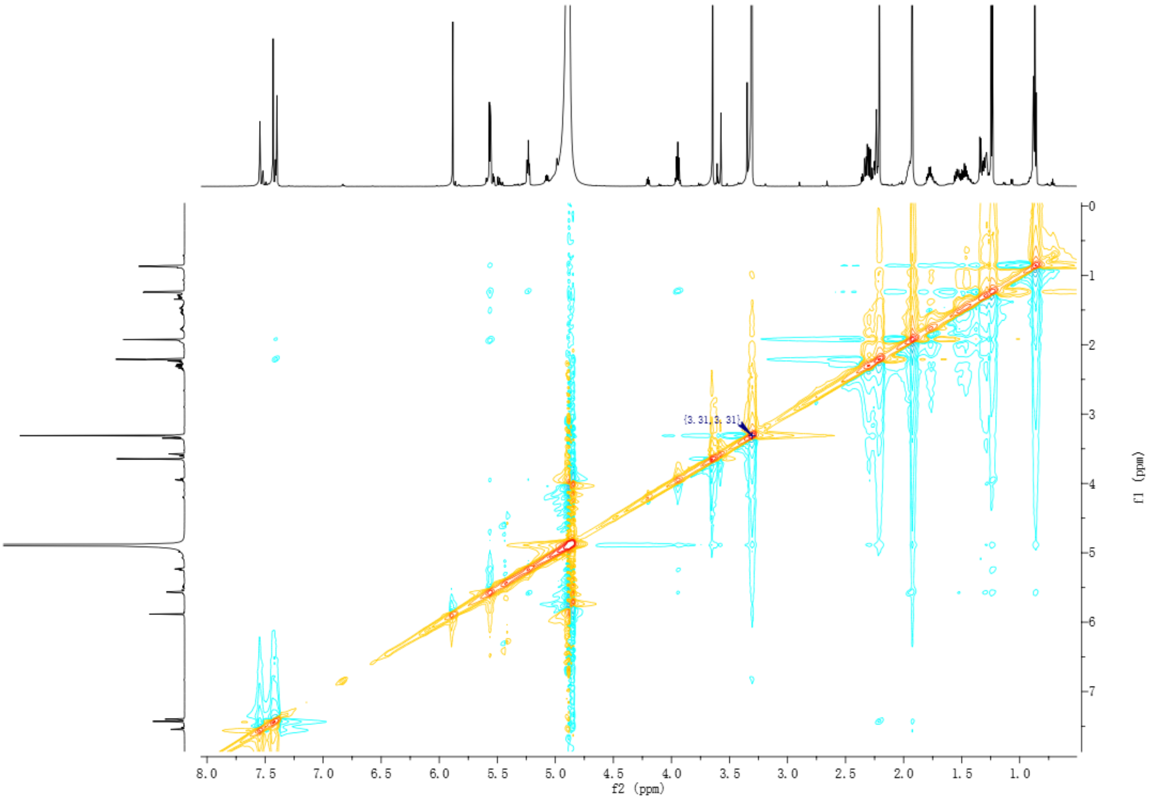

Figure S130. HRESIMS spectrum of hygrocin R (**12**)

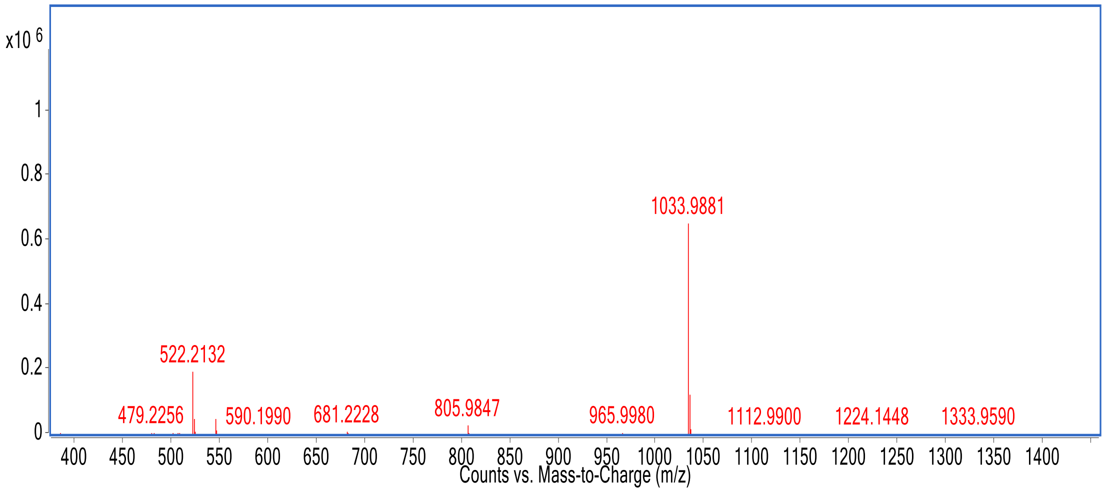

Figure S131. UV spectrum of hygrocin R (**12**)

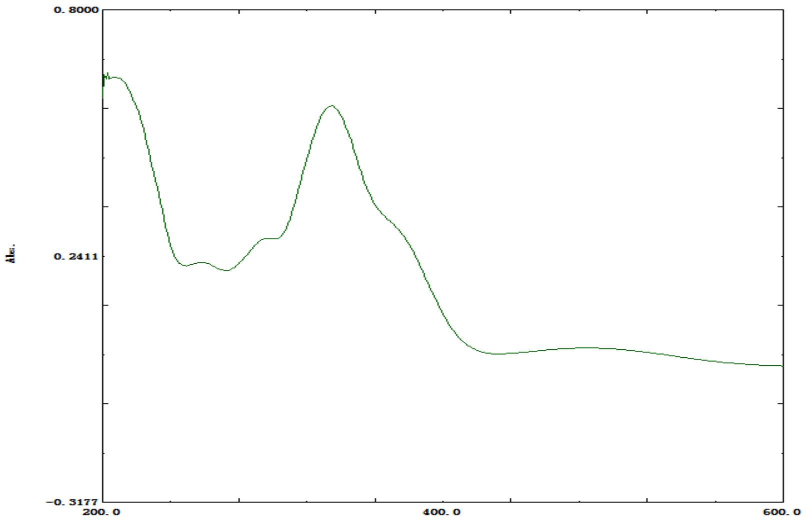

Figure S132. IR spectrum of hygrocin R (**12**)

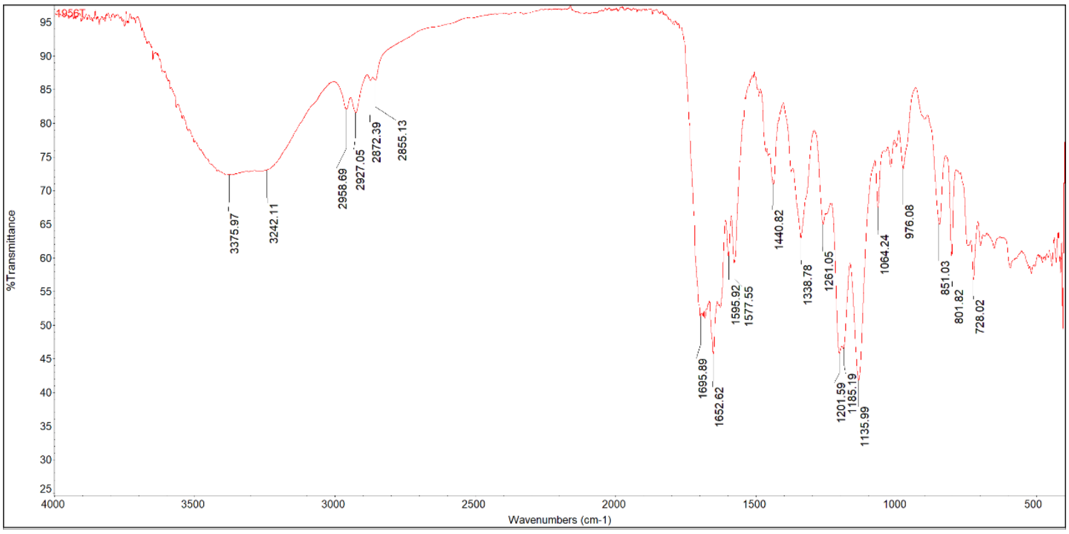

Figure S133. ^{1}H NMR spectrum of hygrocin S (**13**)

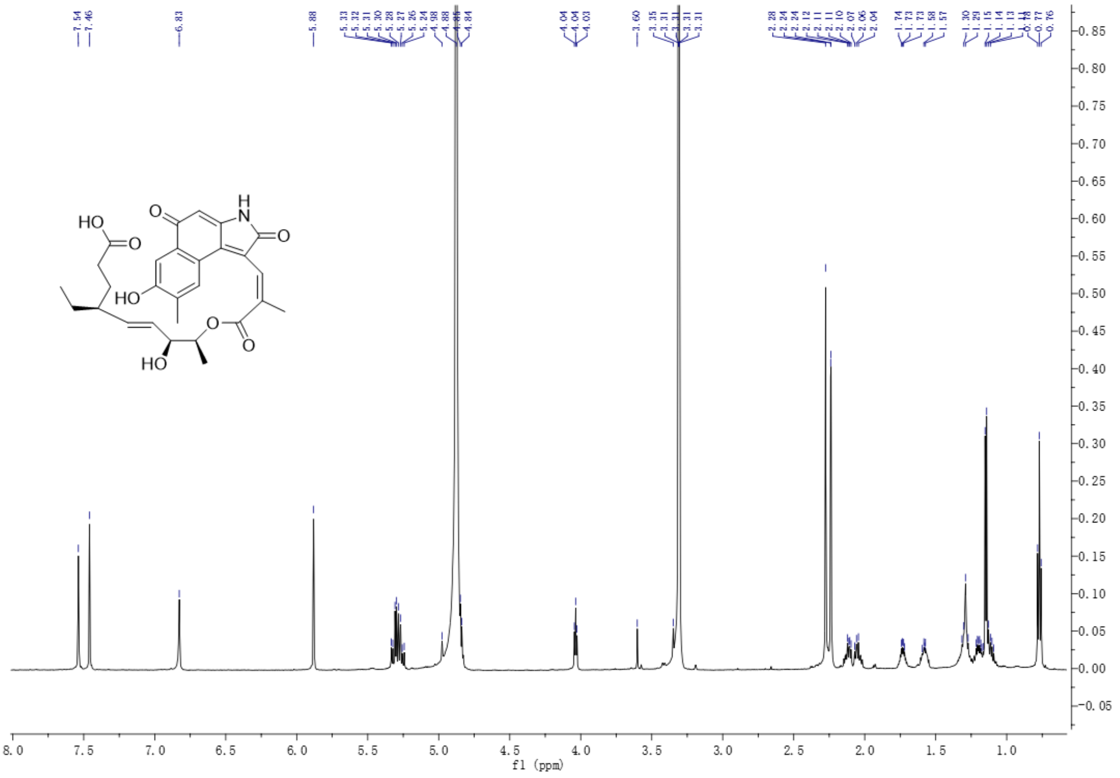

Figure S134. ^{1}H NMR spectrum of hygrocin S (**13**)

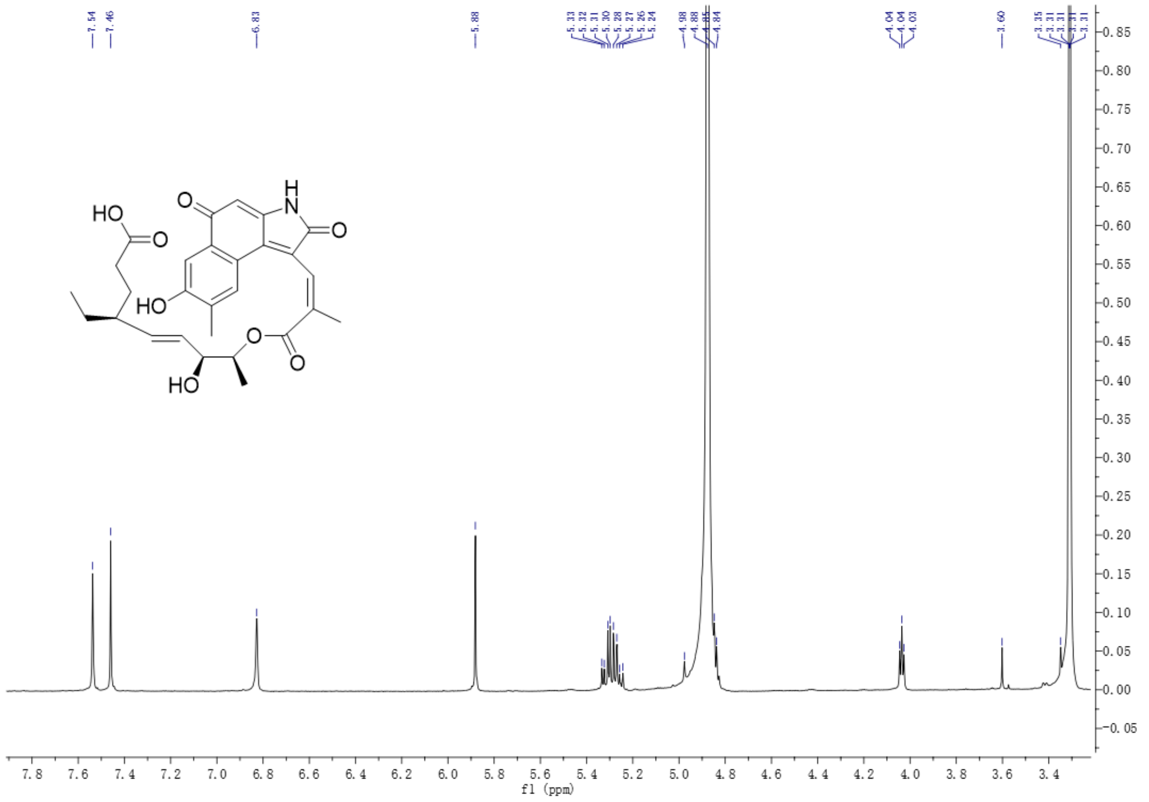

Figure S135. ^{1}H NMR spectrum of hygrocin S (**13**)

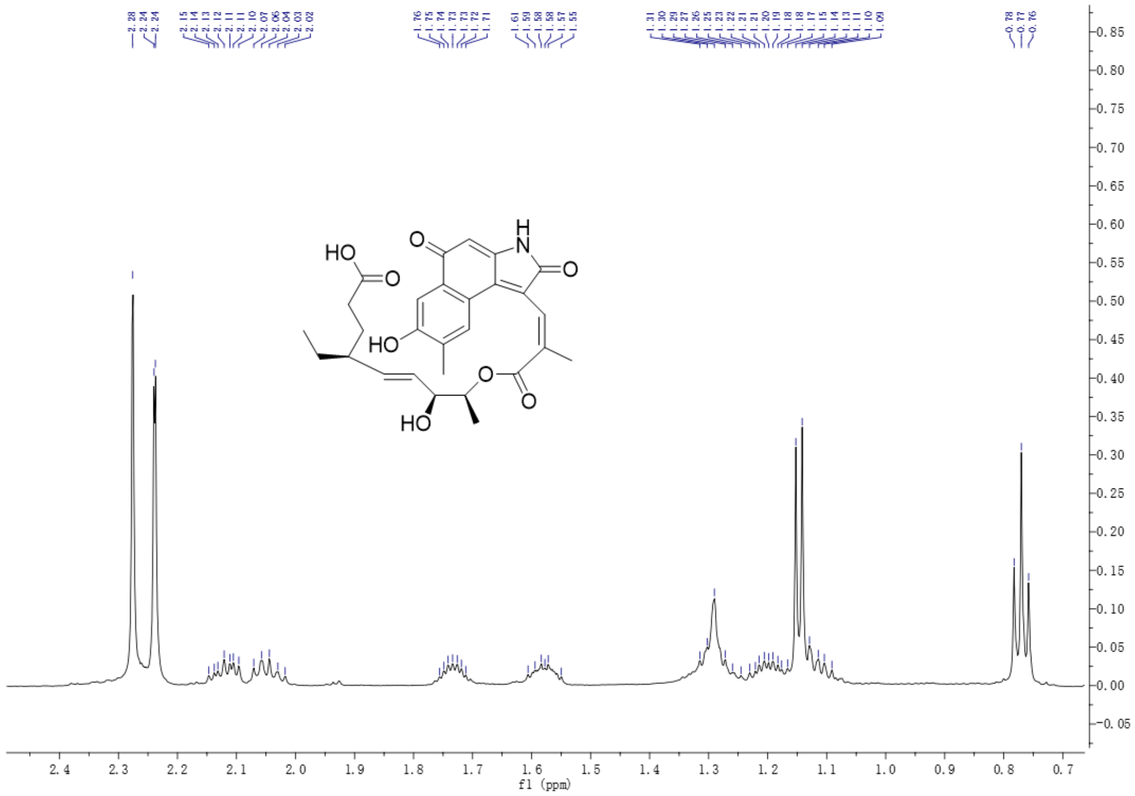

Figure S136. ^{13}C NMR spectrum of hygrocin S (**13**)

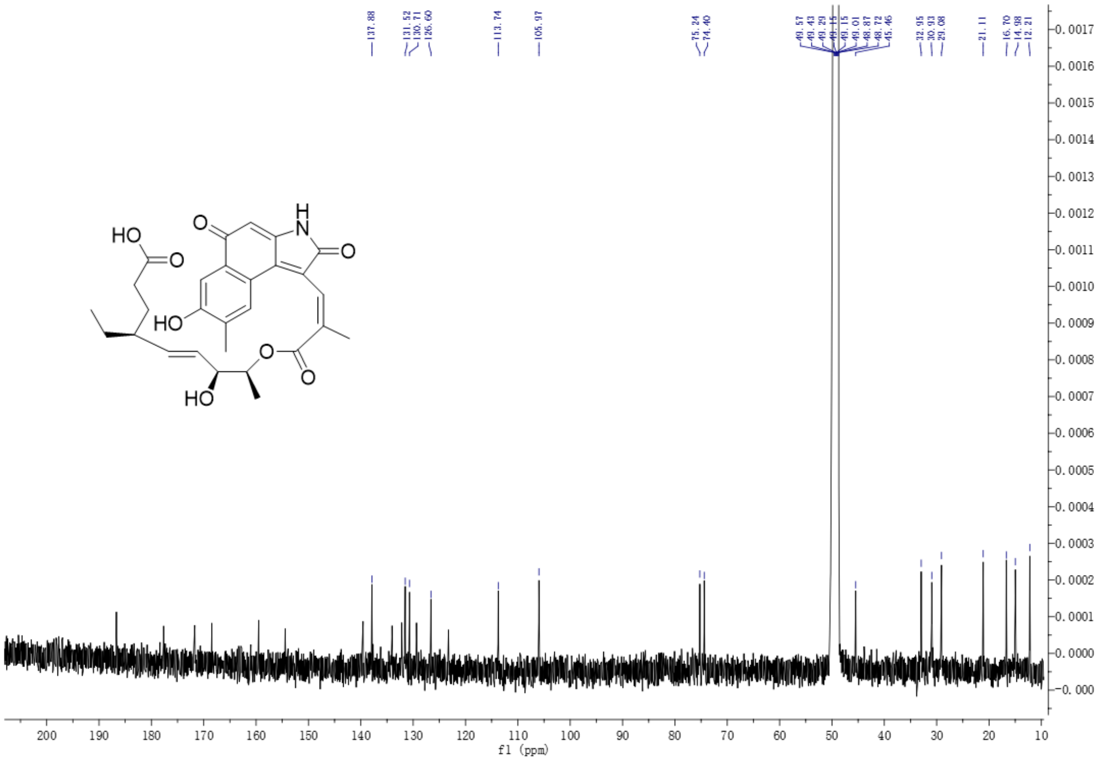

Figure S137. ^{13}C NMR spectrum of hygrocin S (**13**)

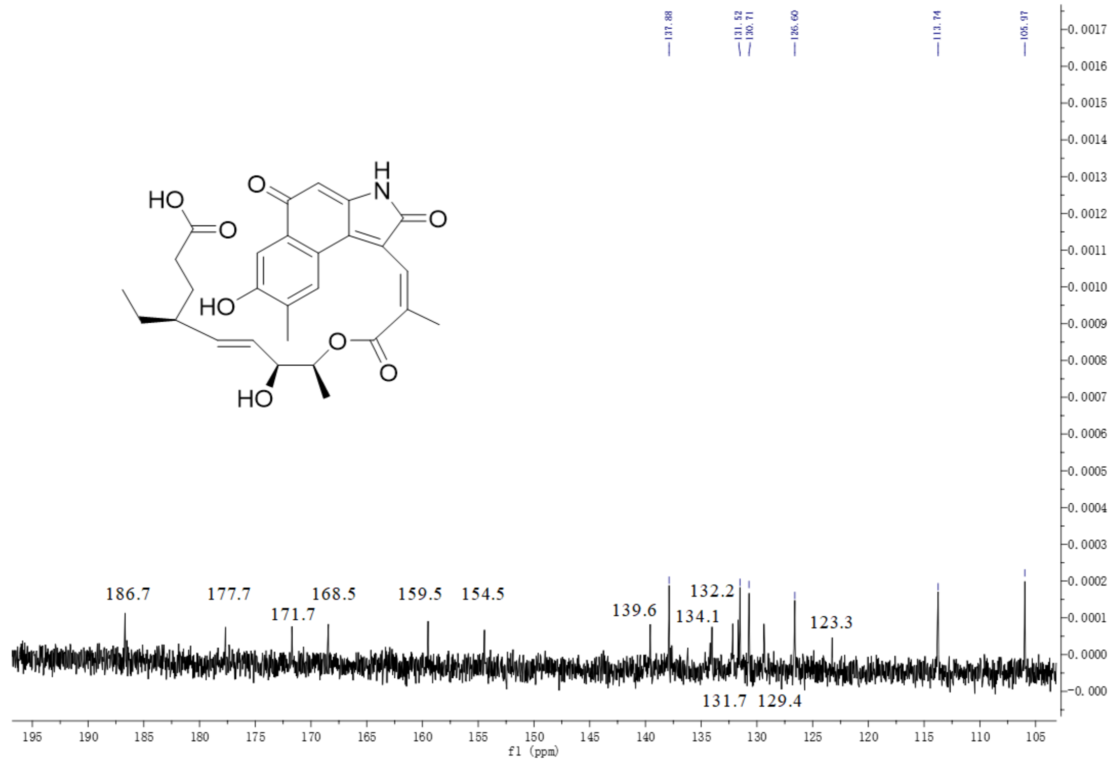

Figure S138. ^{13}C NMR spectrum of hygrocin S (**13**)

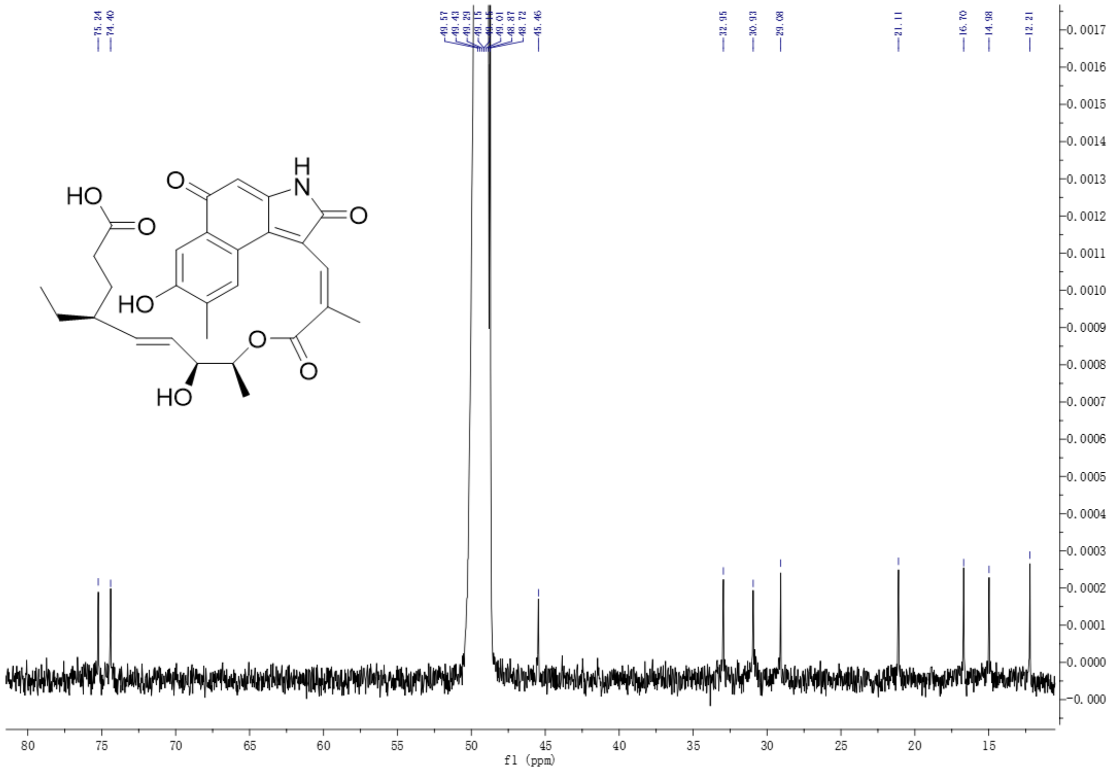

Figure S139. HMQC spectrum of hygrocin S (**13**)

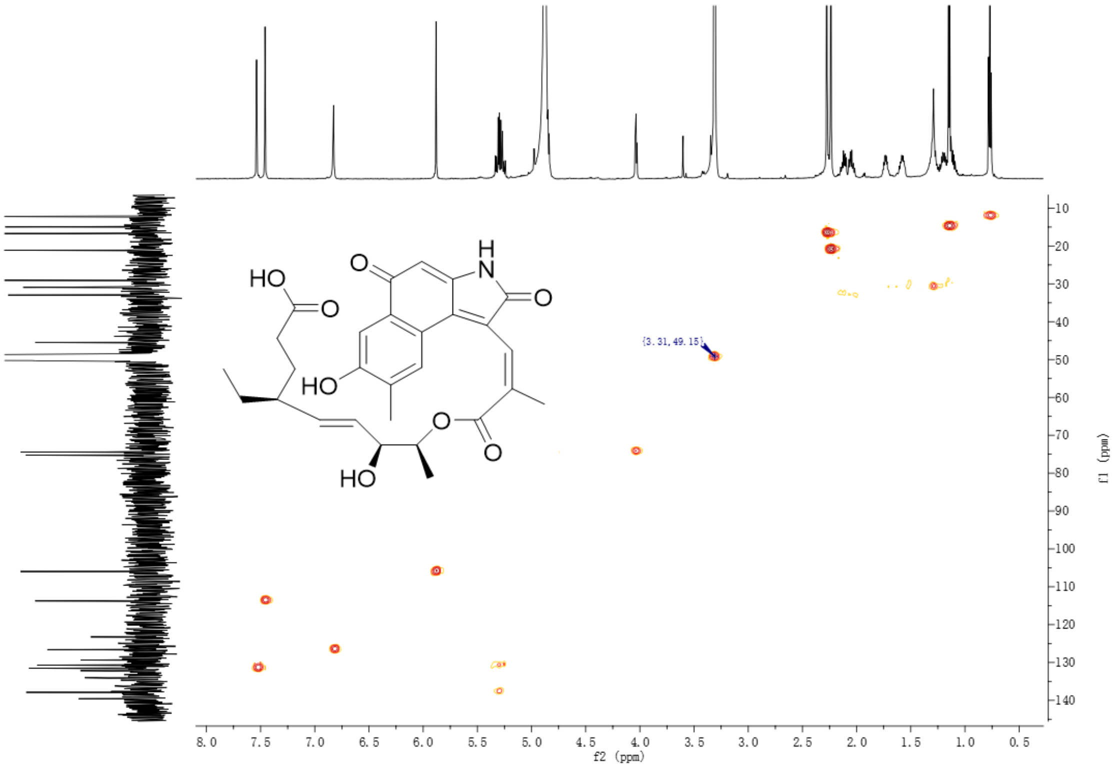

Figure S140. COSY spectrum of hygrocin S (**13**)

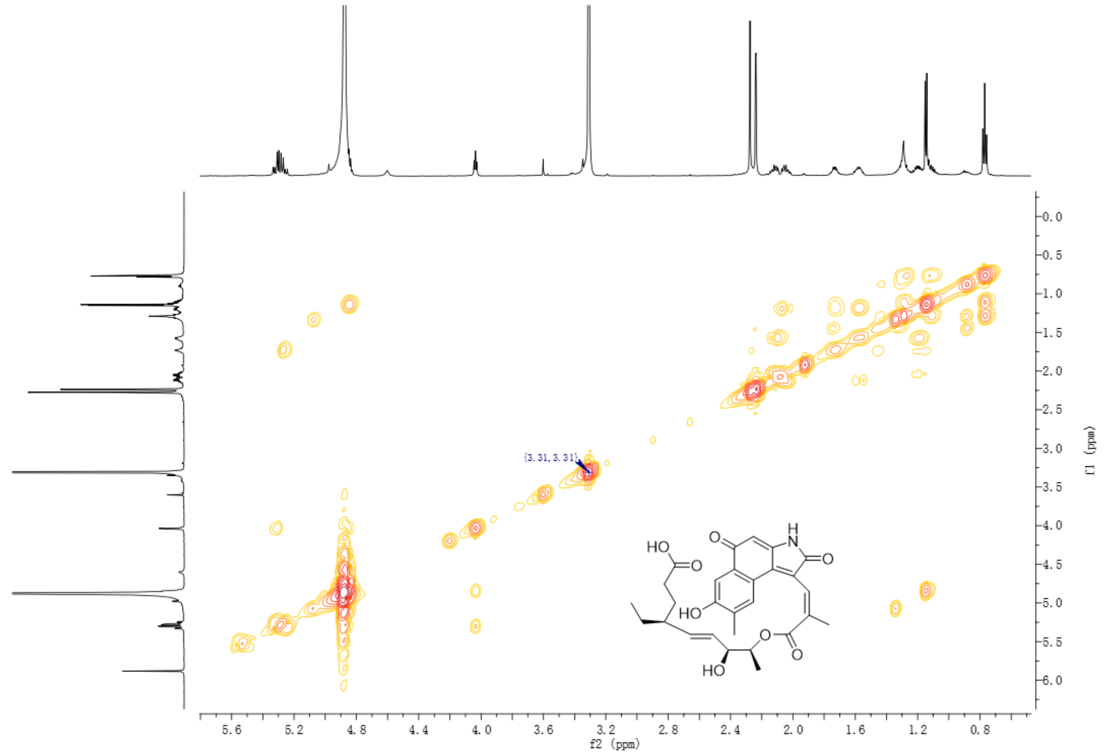

Figure S141. HMBC spectrum of hygrocin S (**13**)

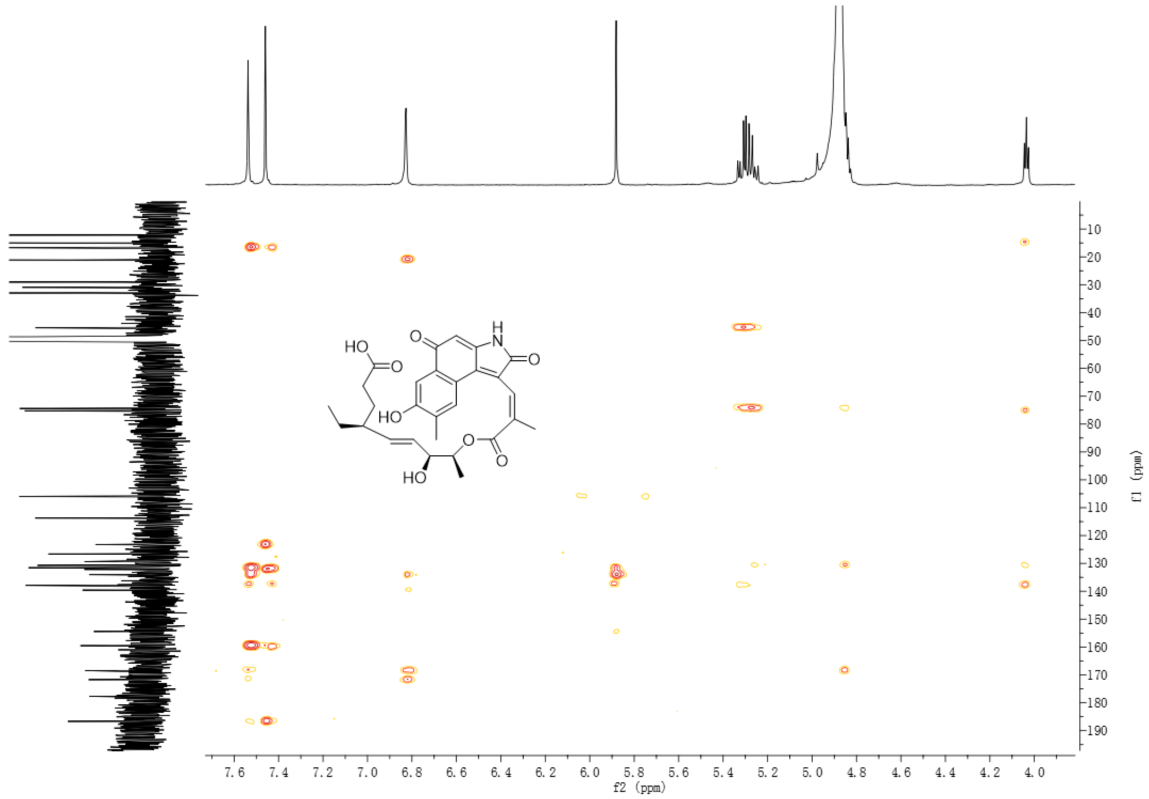

Figure S142. HMBC spectrum of hygrocin S (**13**)

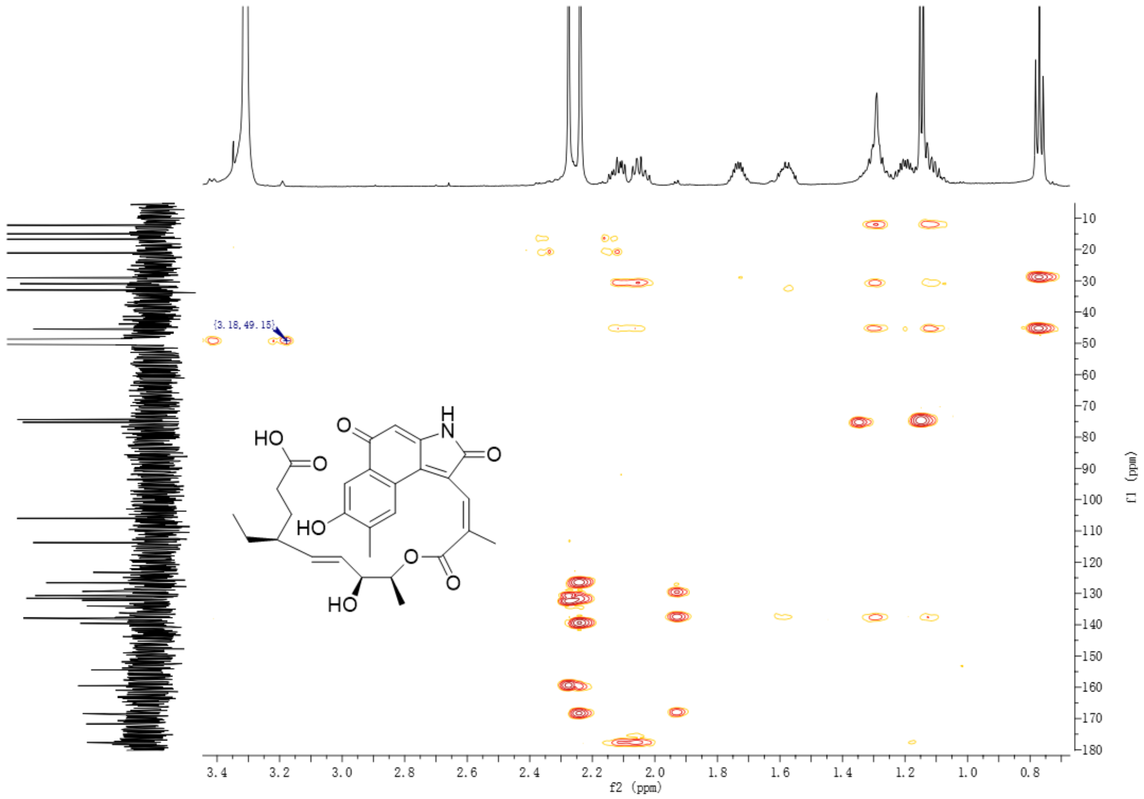

Figure S143. NOESY spectrum of hygrocin S (**13**)

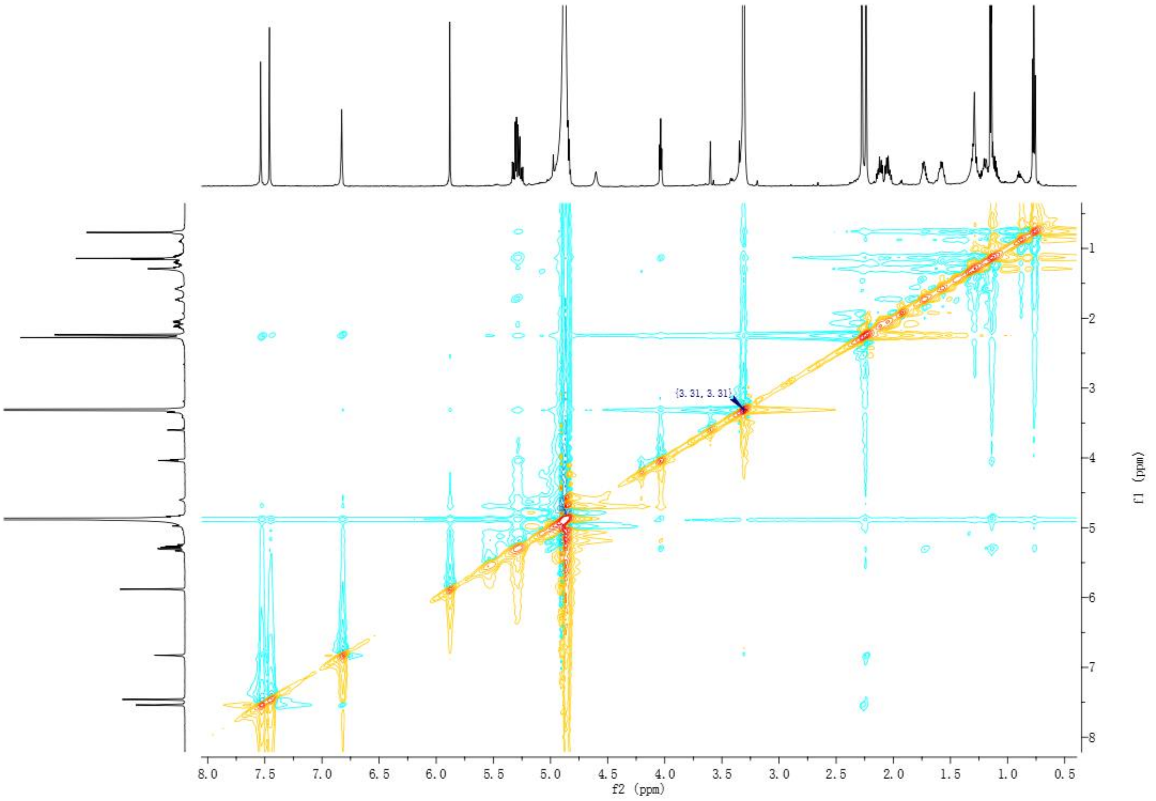

Figure S144. HRESIMS spectrum of hygrocin S (**13**)

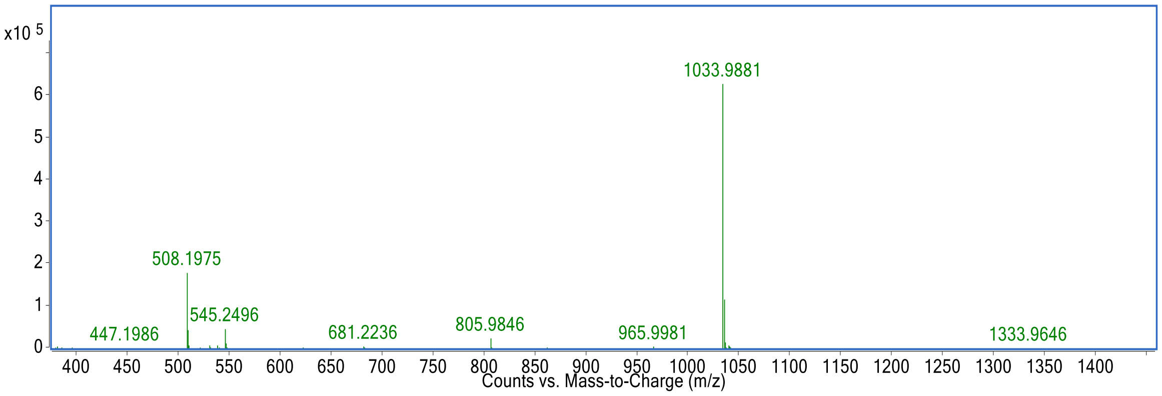

Figure S145. UV spectrum of hygrocin S (**13**)

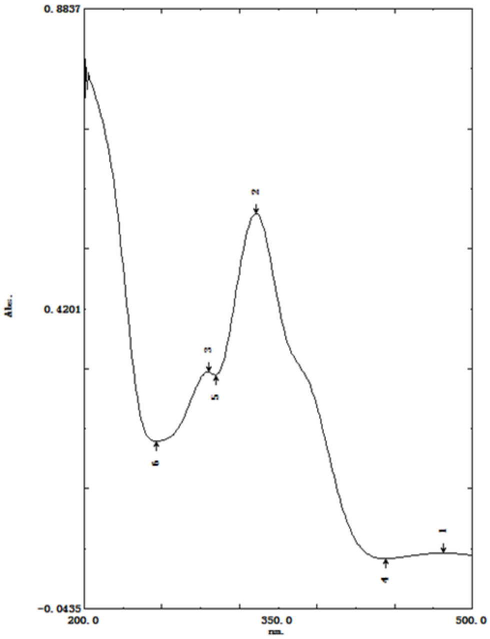

Figure S146. IR spectrum of hygrocin S (**13**)

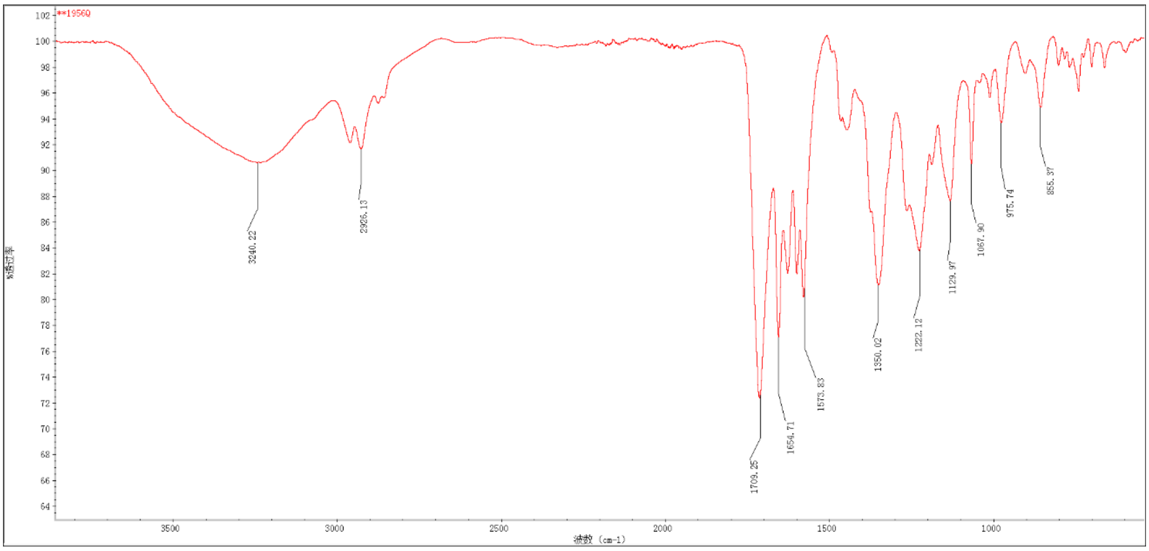

Figure S147. ^{1}H NMR spectrum of hygrocin T (**17**)

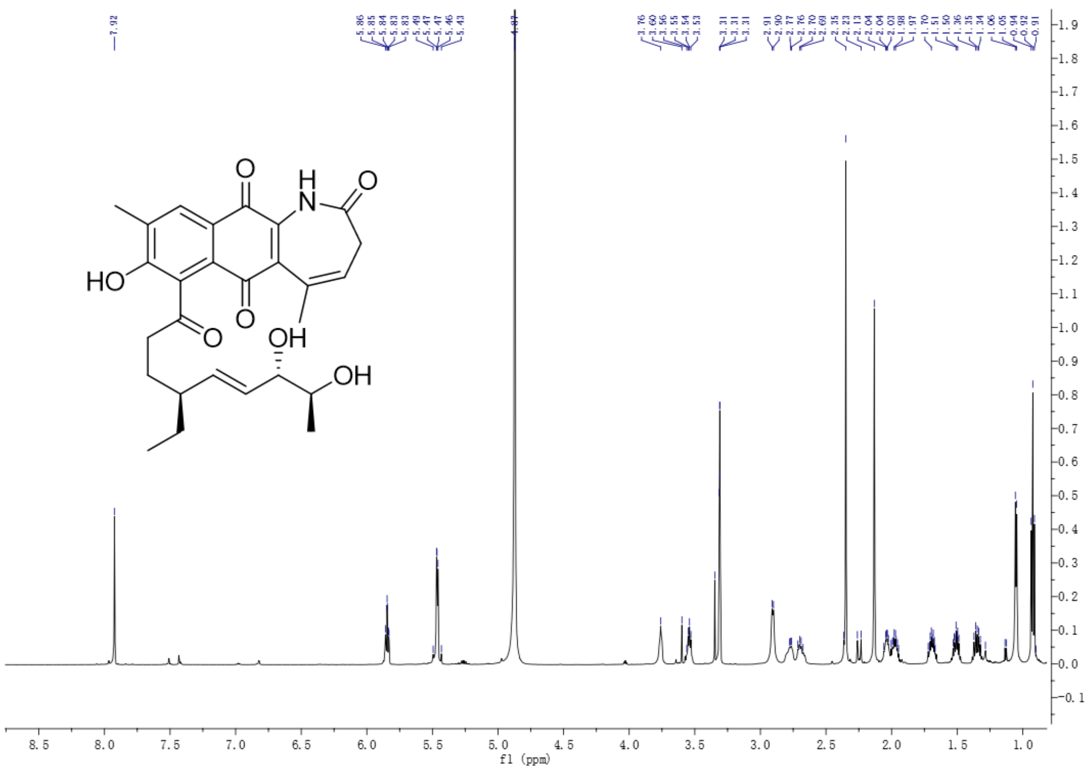

Figure S148. ^{1}H NMR spectrum of hygrocin T (**17**)

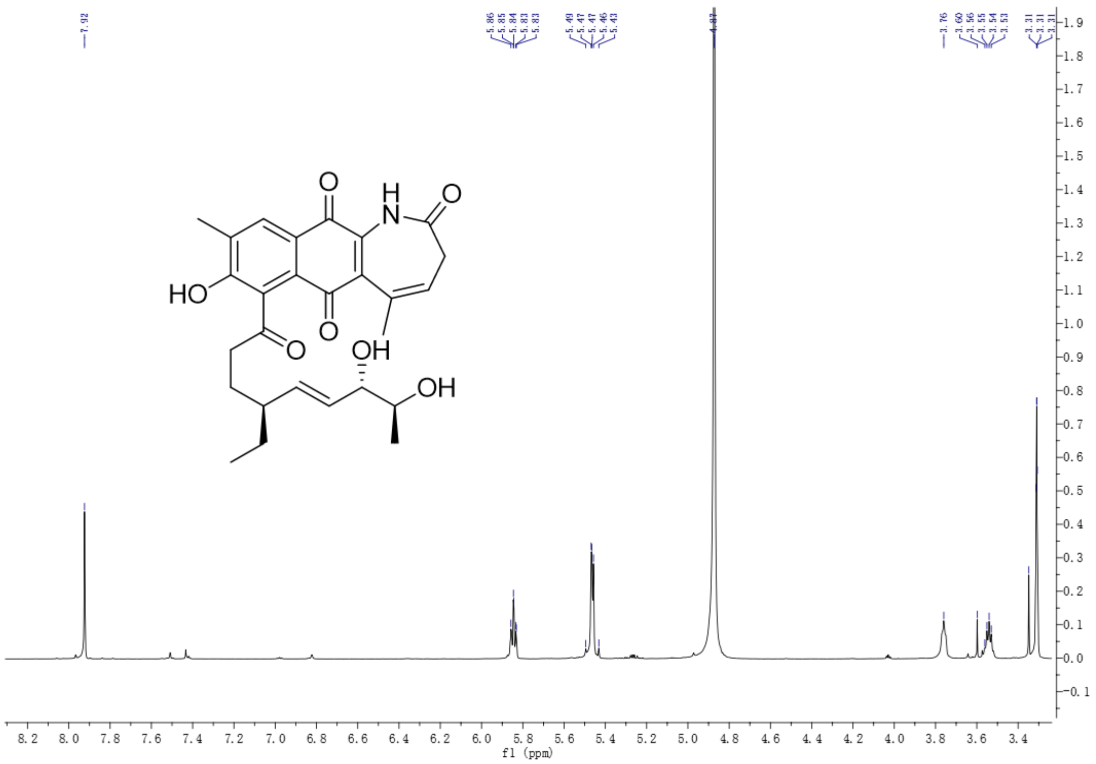

Figure S149. ^{1}H NMR spectrum of hygrocin T (**17**)

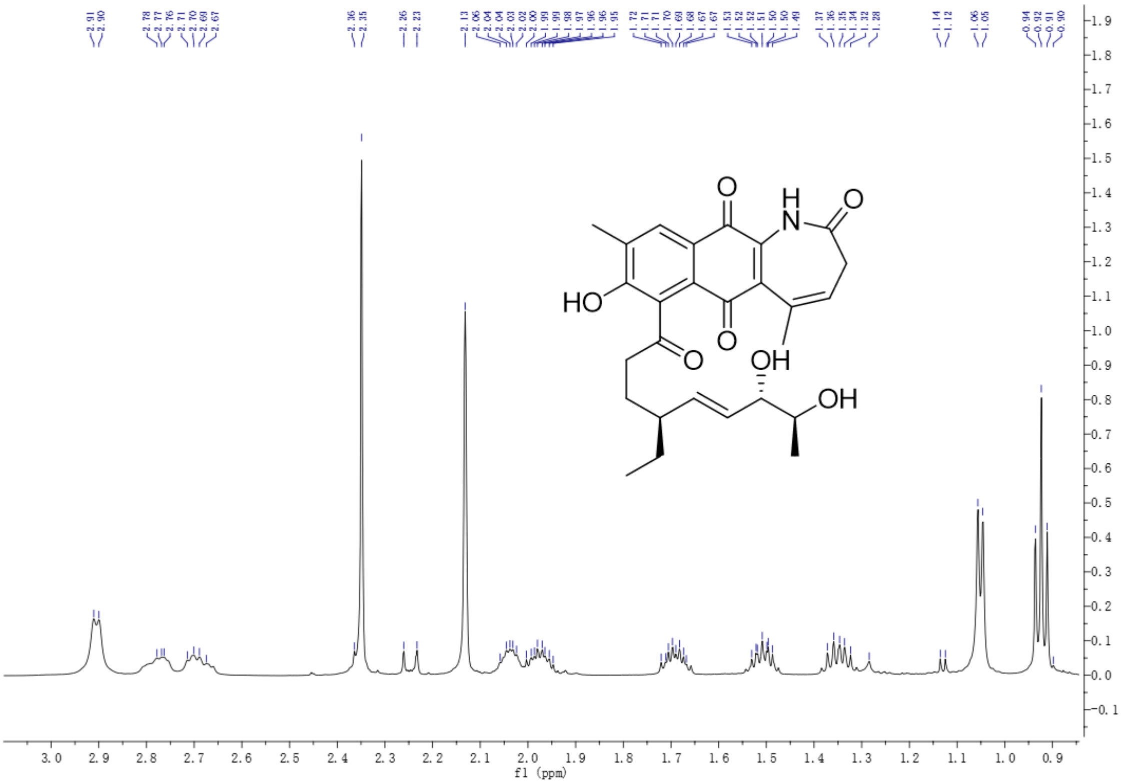

Figure S150. ^{13}C NMR spectrum of hygrocin T (**17**)

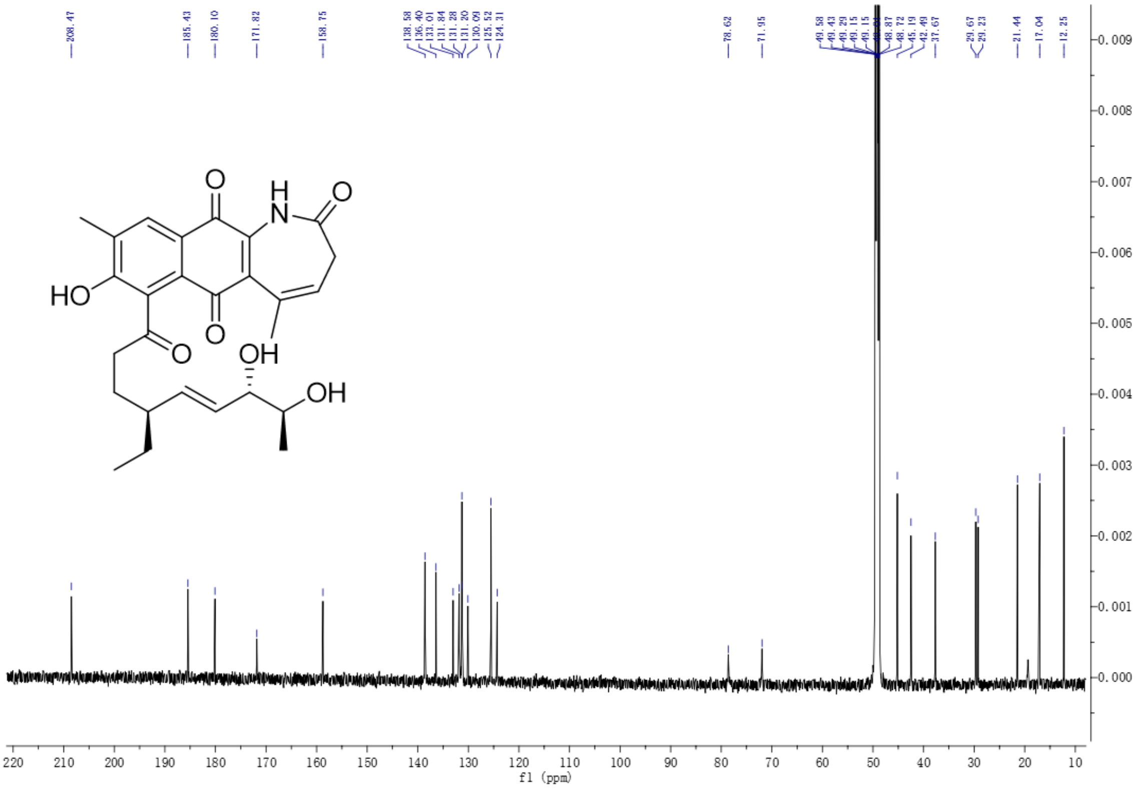

Figure S151. ^{13}C NMR spectrum of hygrocin T (**17**)

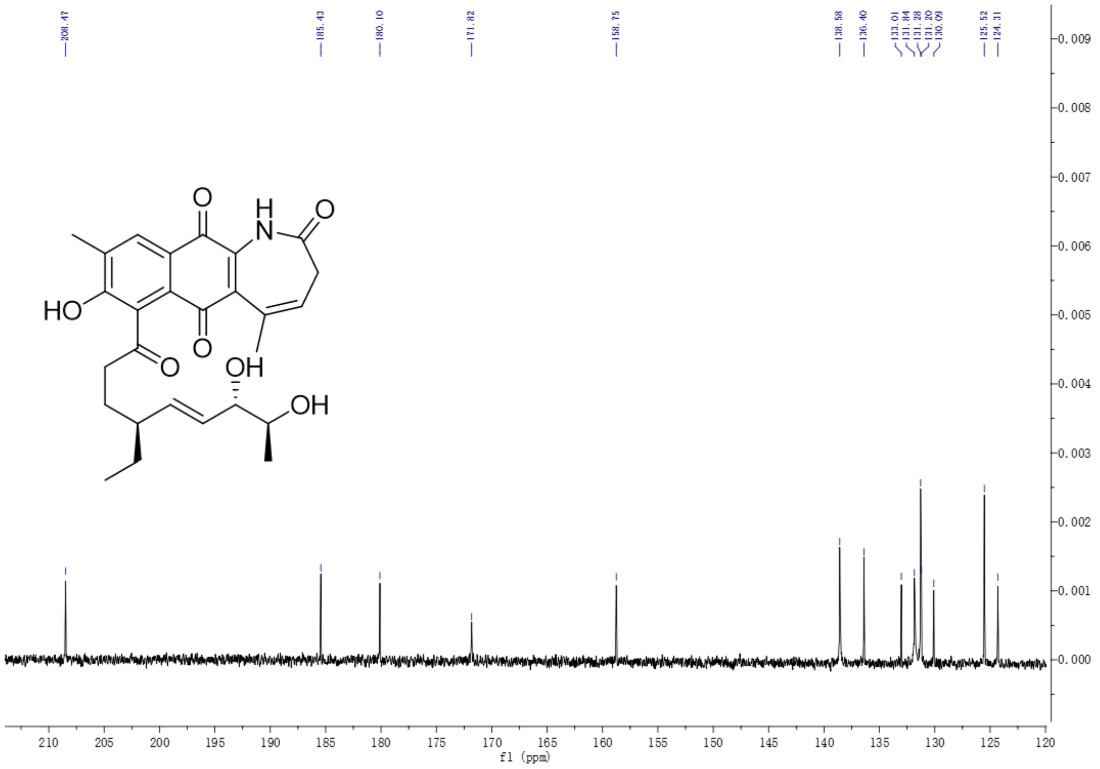

Figure S152. ^{13}C NMR spectrum of hygrocin T (**17**)

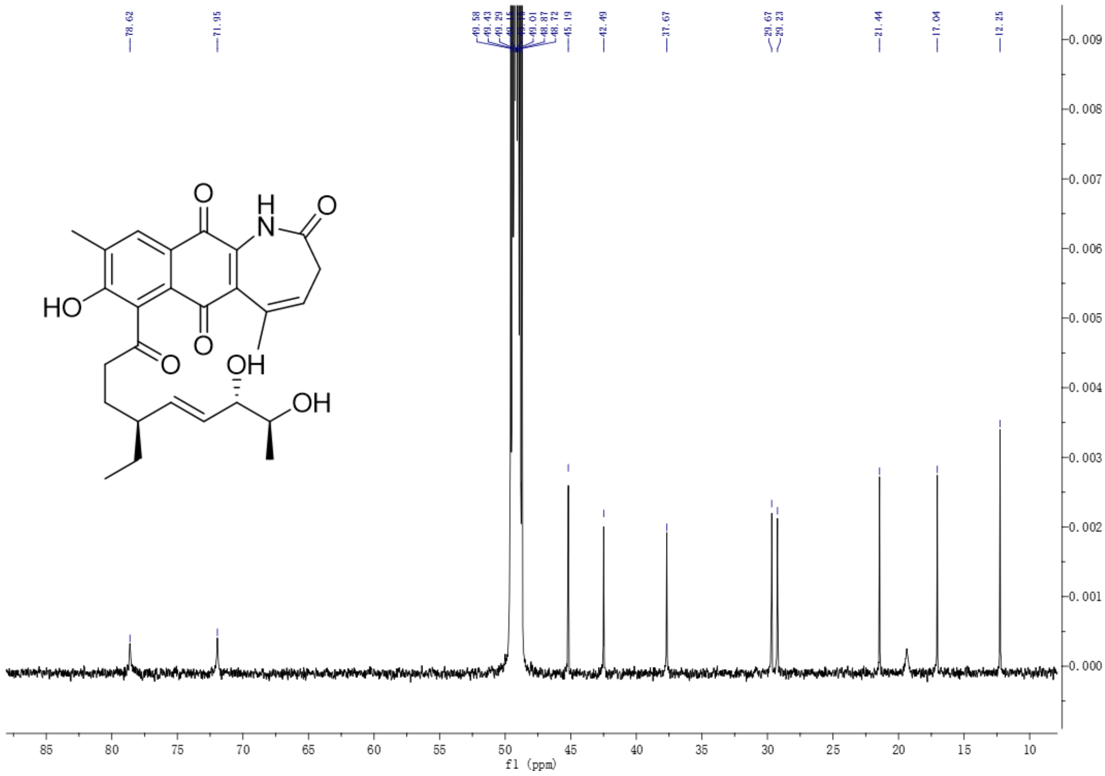

Figure S153. HMQC spectrum of hygrocin T (**17**)

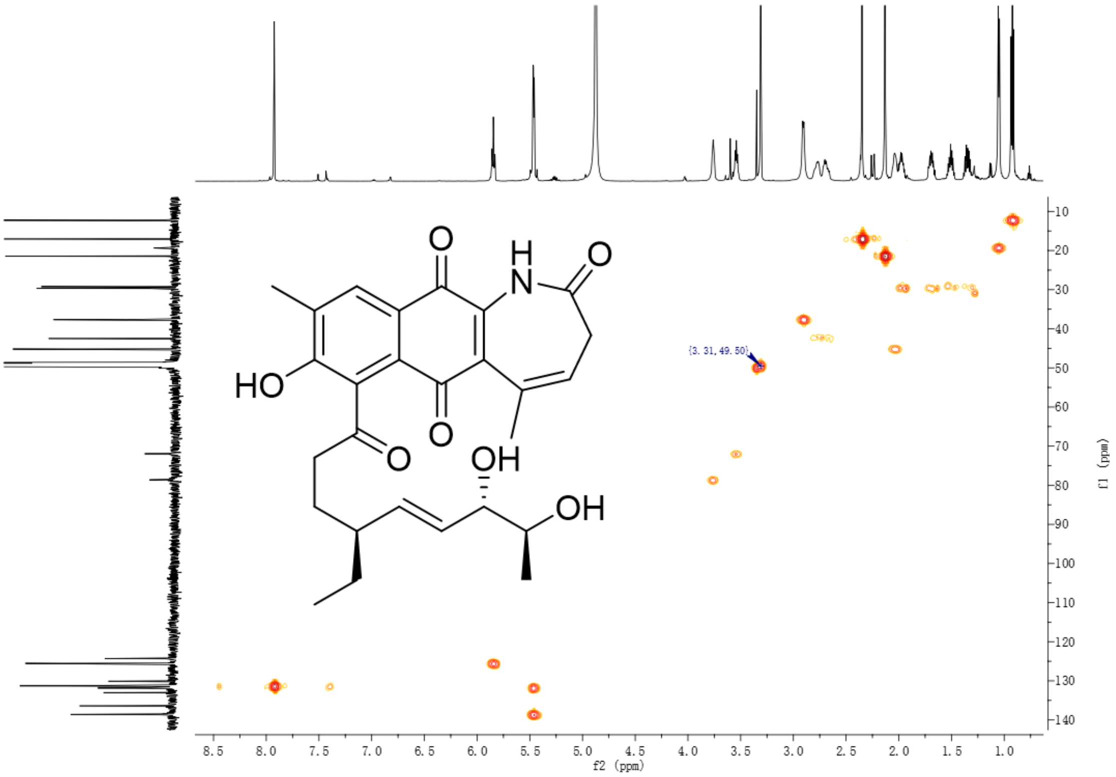

Figure S154. COSY spectrum of hygrocin T (**17**)

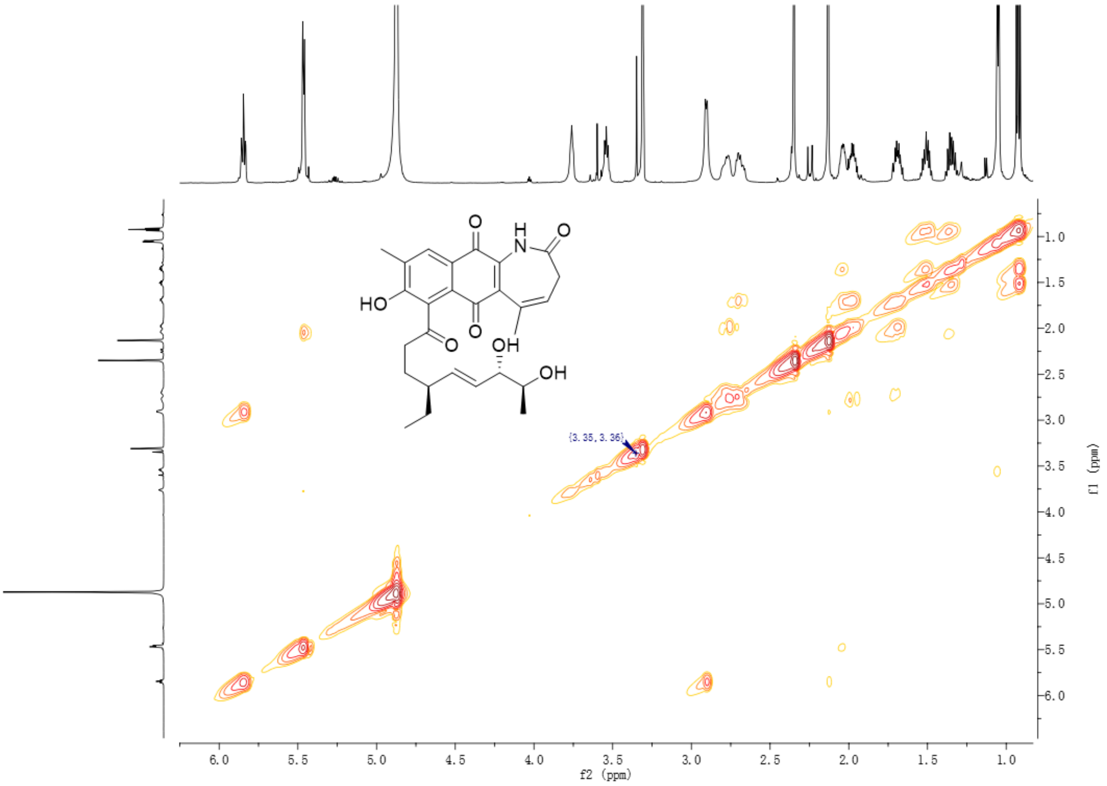

Figure S155. HMBC spectrum of hygrocin T (**17**)

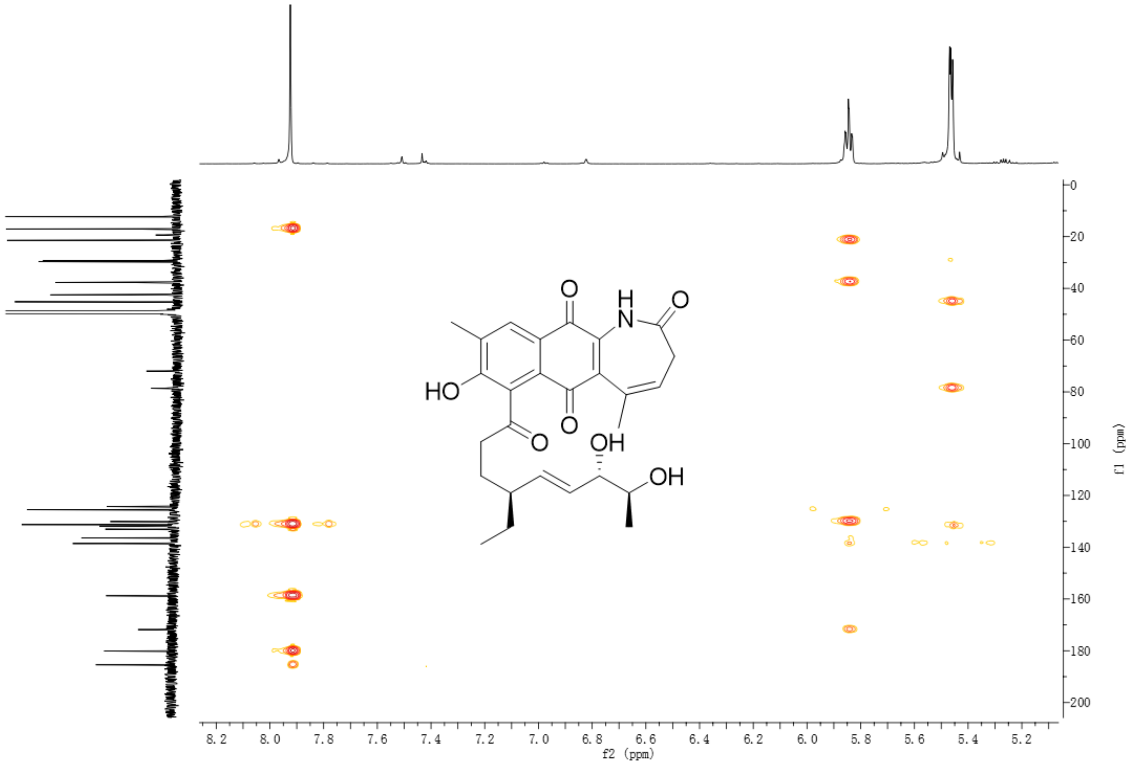

Figure S156. HMBC spectrum of hygrocin T (**17**)

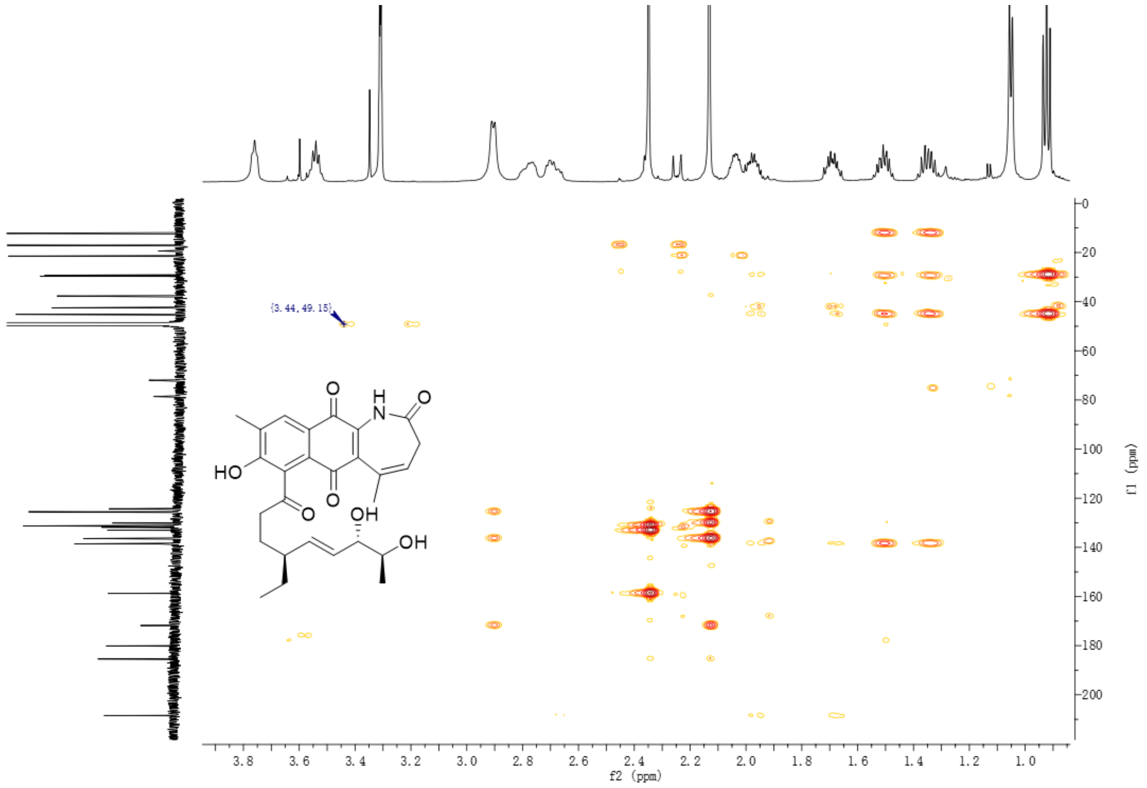

Figure S157. NOESY spectrum of hygrocin T (**17**)

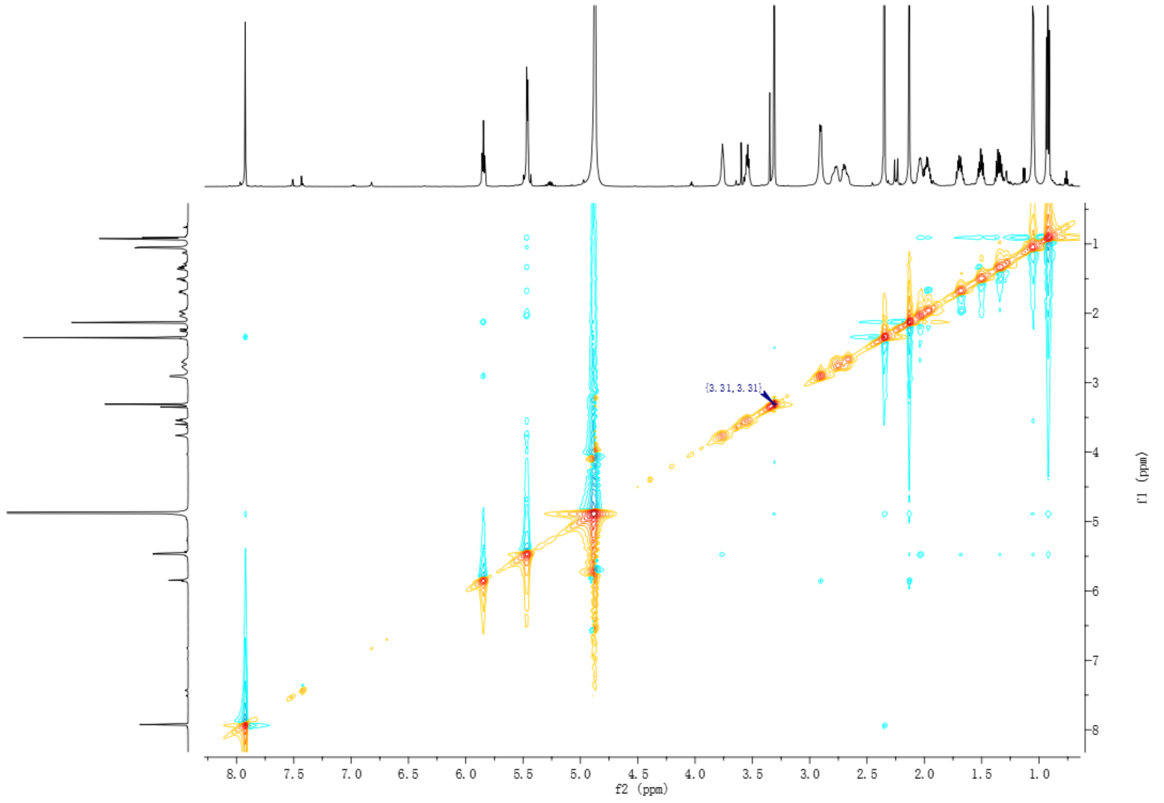

Figure S158. HRESIMS spectrum of hygrocin T (**17**)

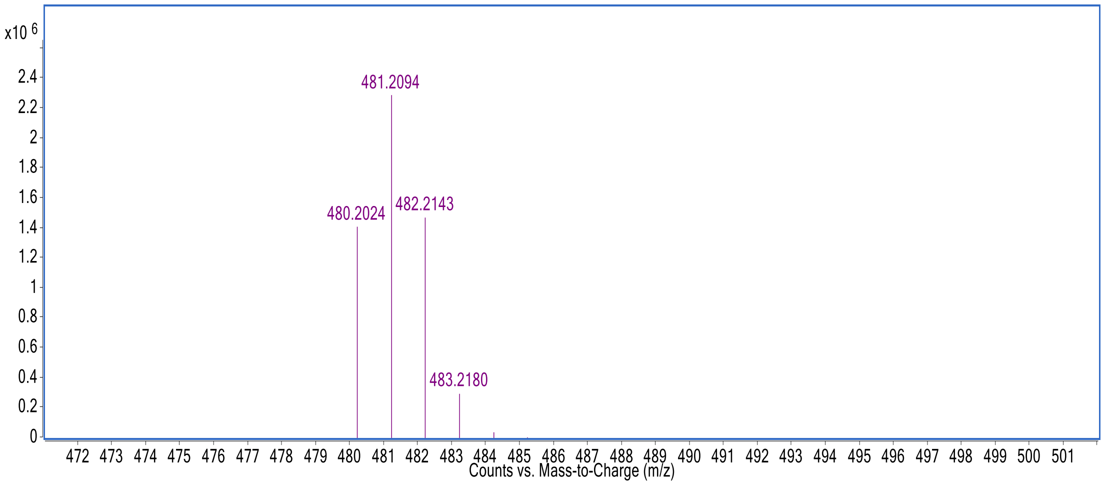

Figure S159. UV spectrum of hygrocin T (**17**)

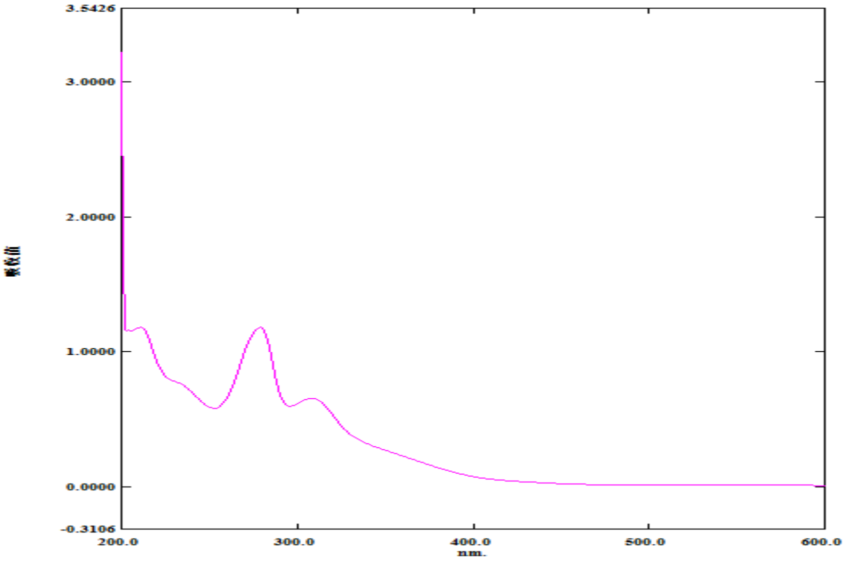

Figure S160. IR spectrum of hygrocin T (**17**)

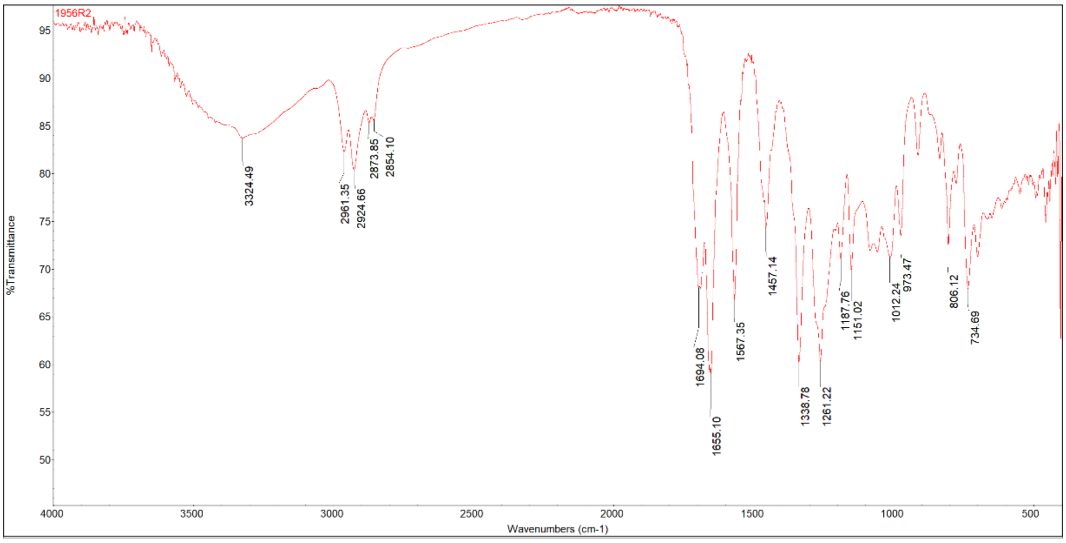

Figure S161. ^{1}H NMR spectrum of hygrocin U (**18**)

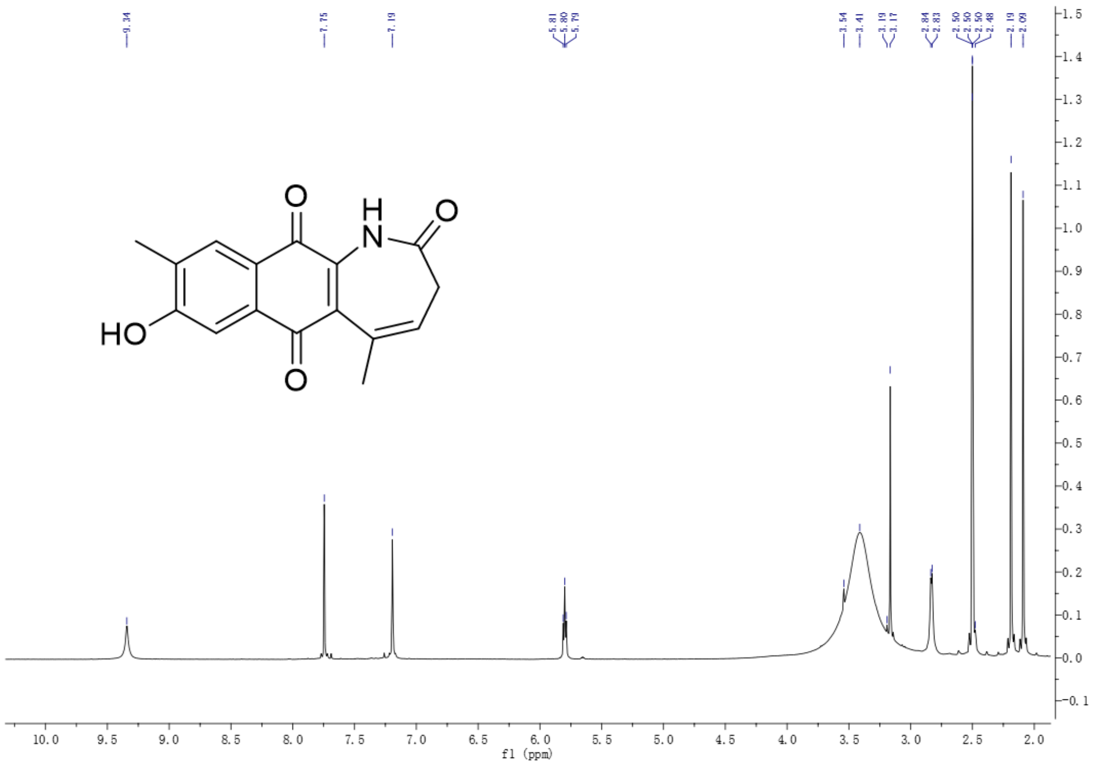

Figure S162. ^{13}C NMR spectrum of hygrocin U (**18**)

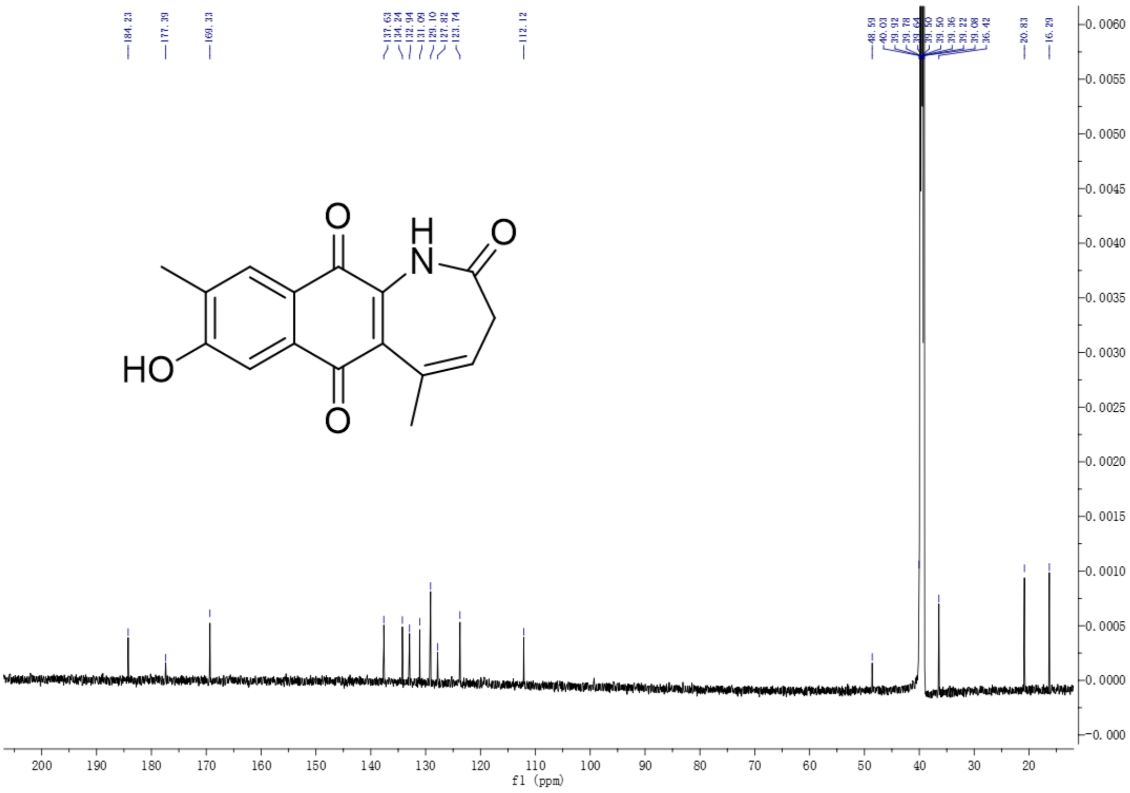

Figure S163. HMQC spectrum of hygrocin U (**18**)

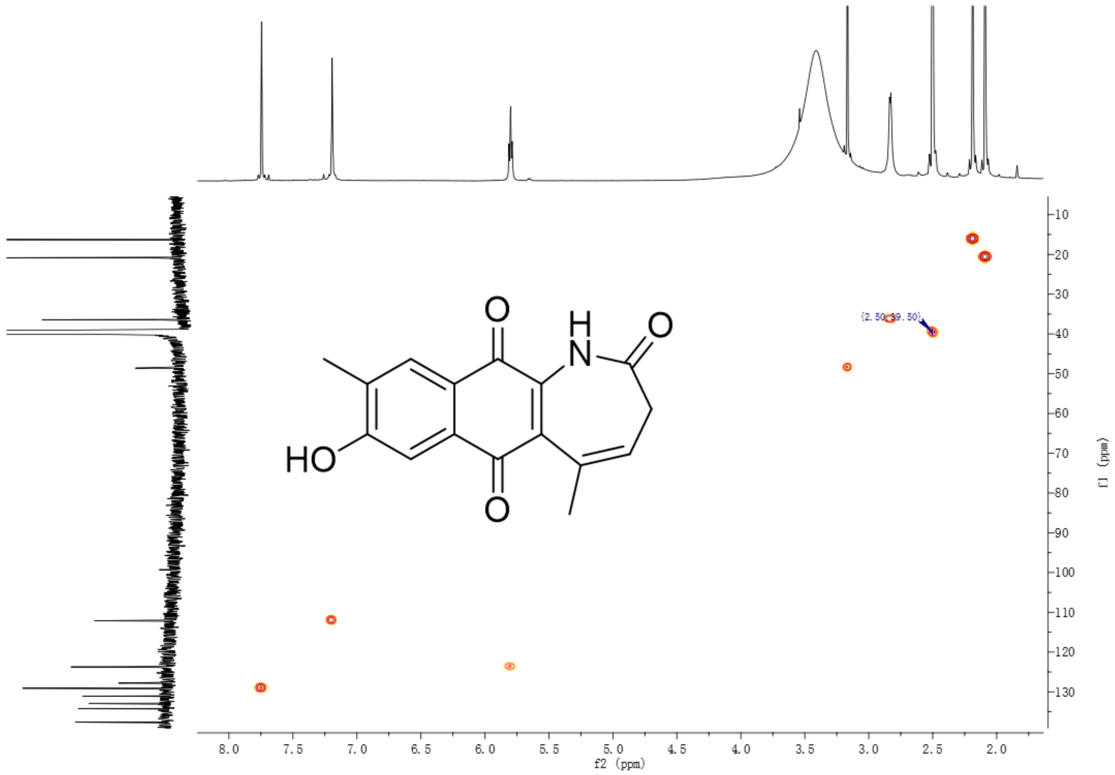

Figure S164. COSY spectrum of hygrocin U (**18**)

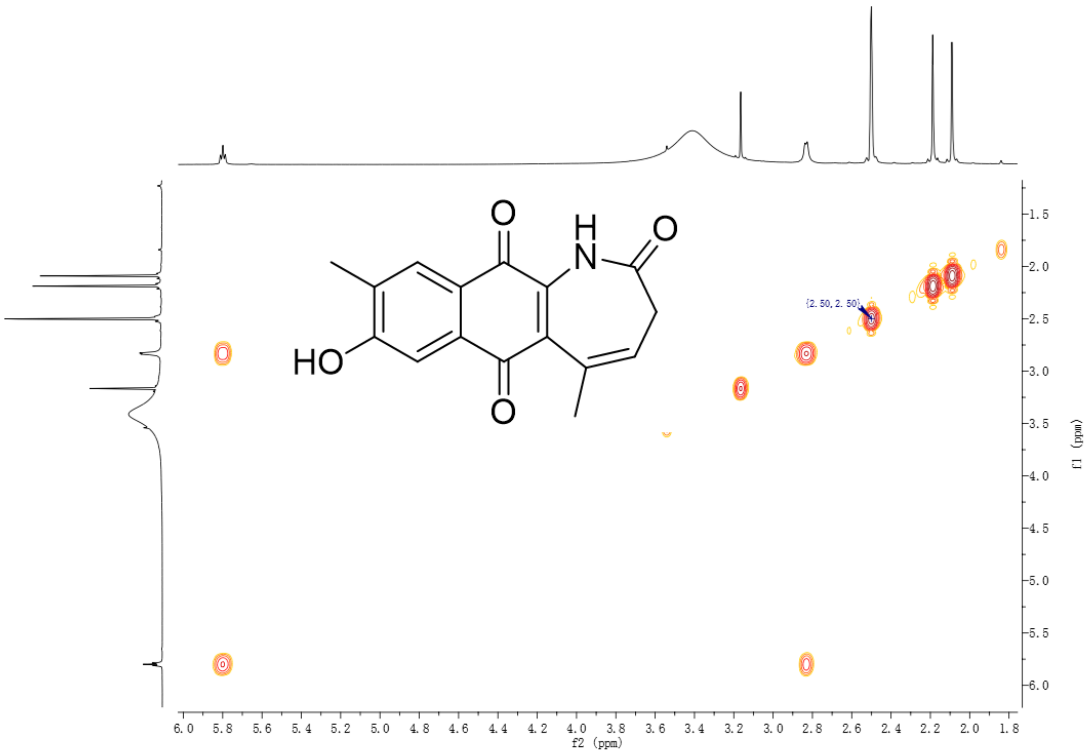

Figure S165. HMBC spectrum of hygrocin U (**18**)

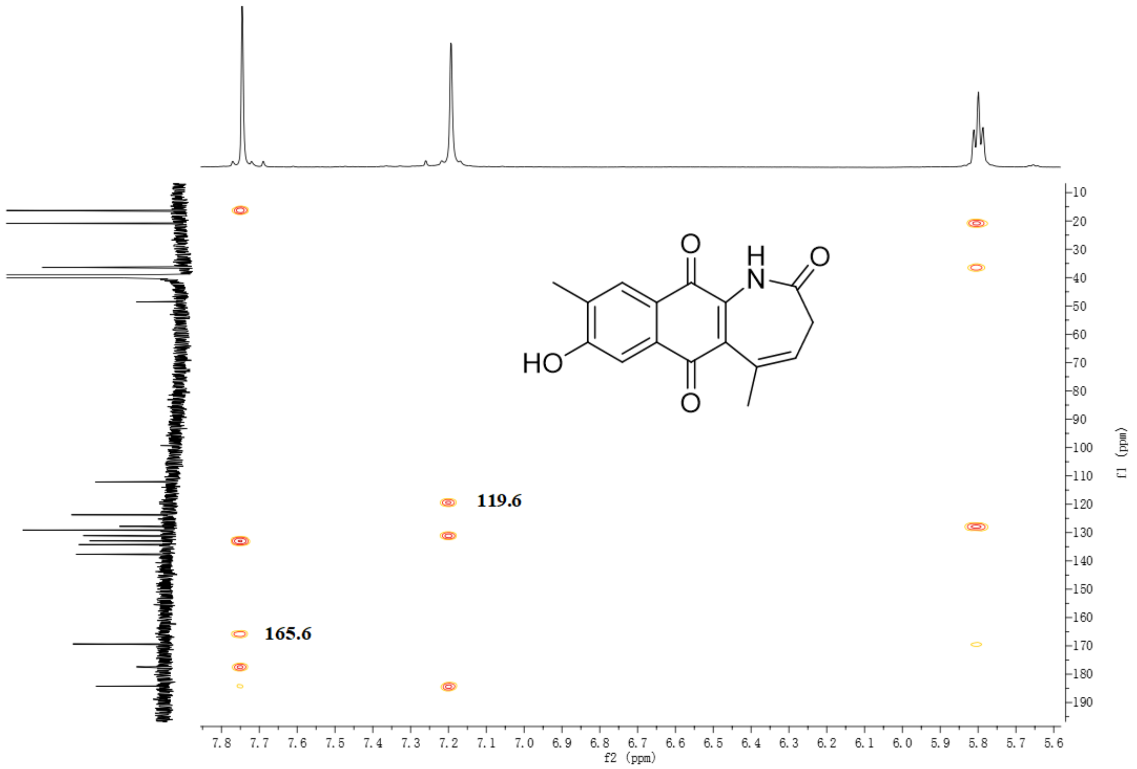

Figure S166. HMBC spectrum of hygrocin U (**18**)

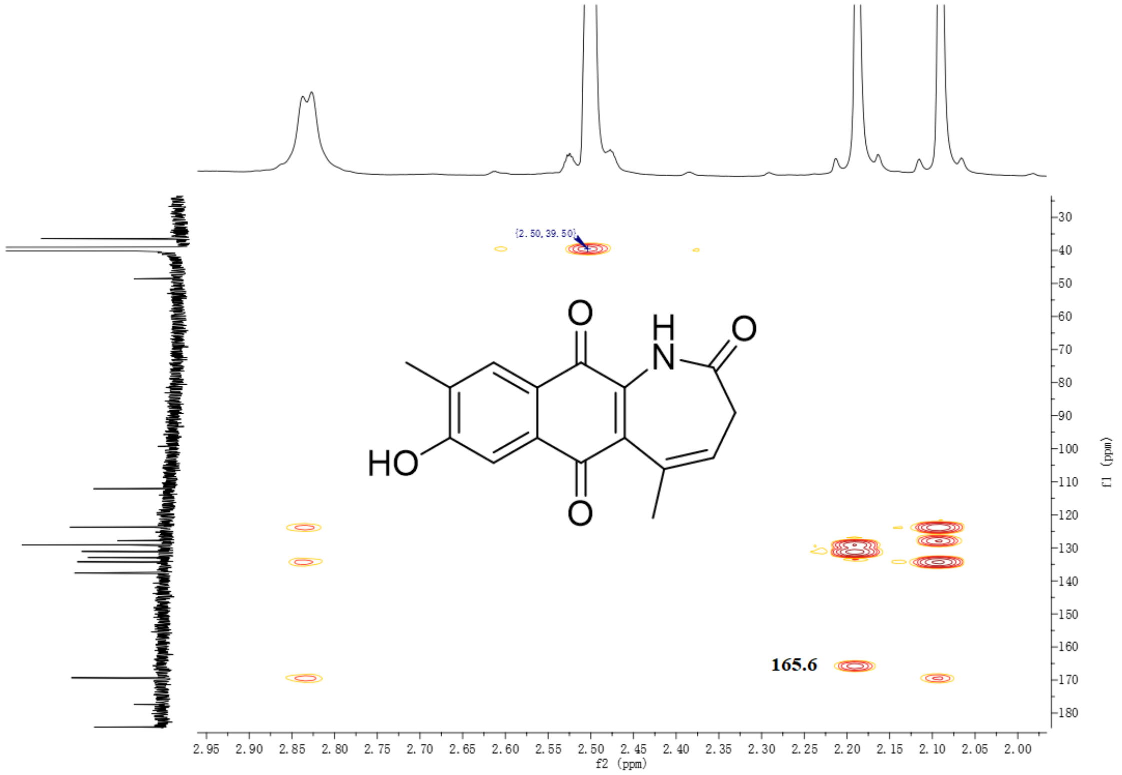

Figure S167. HRESIMS spectrum of hygrocin U (**18**)

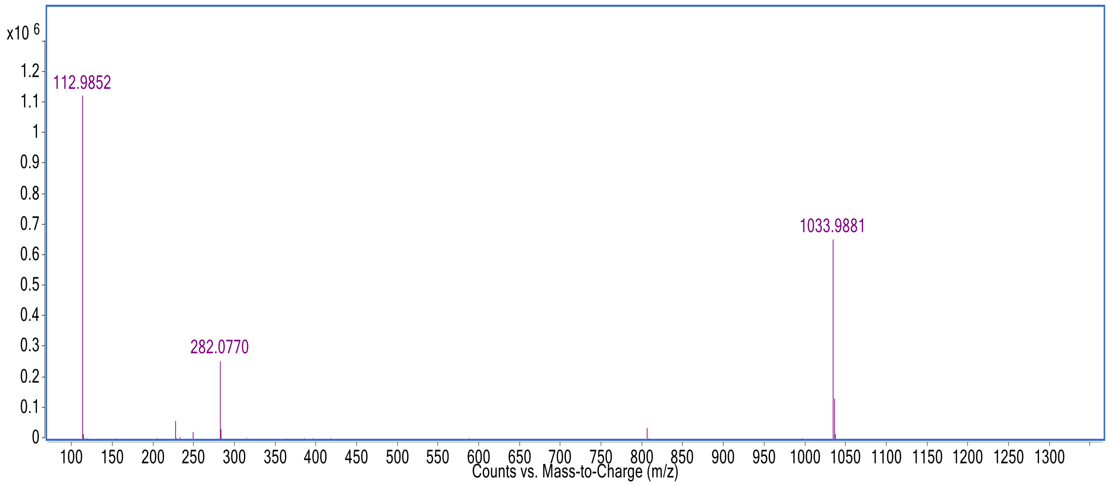

Figure S168. UV spectrum of hygrocin U (**18**)

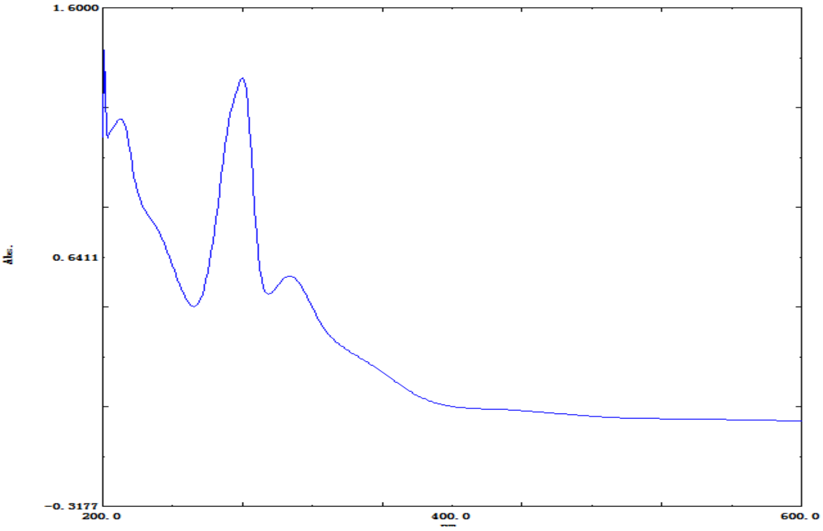

Figure S169. IR spectrum of hygrocin U (**18**)

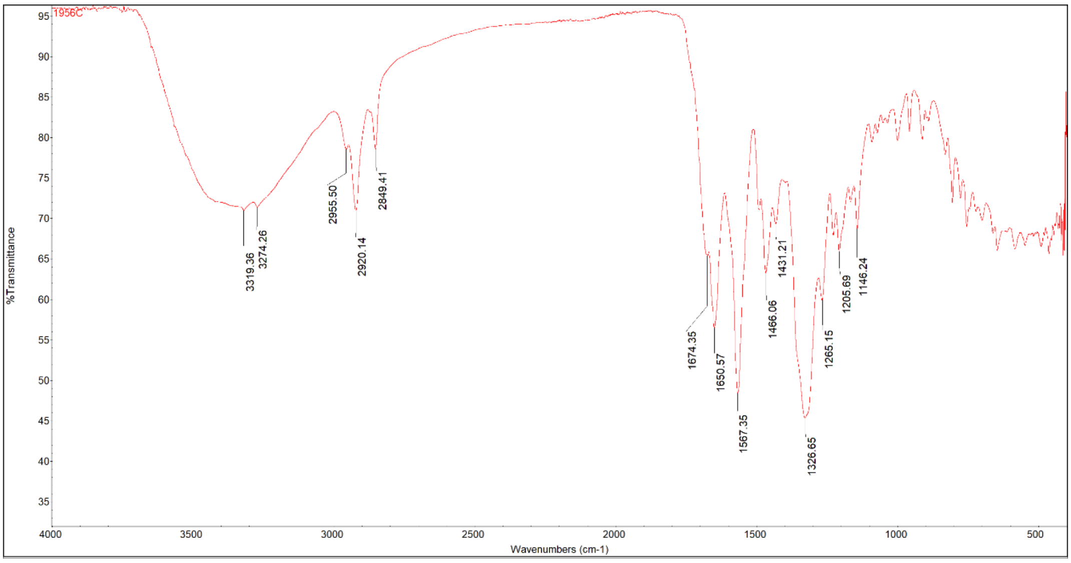

Figure S170. ^{1}H NMR spectrum of streptobenzenepropanamide A (**23**)

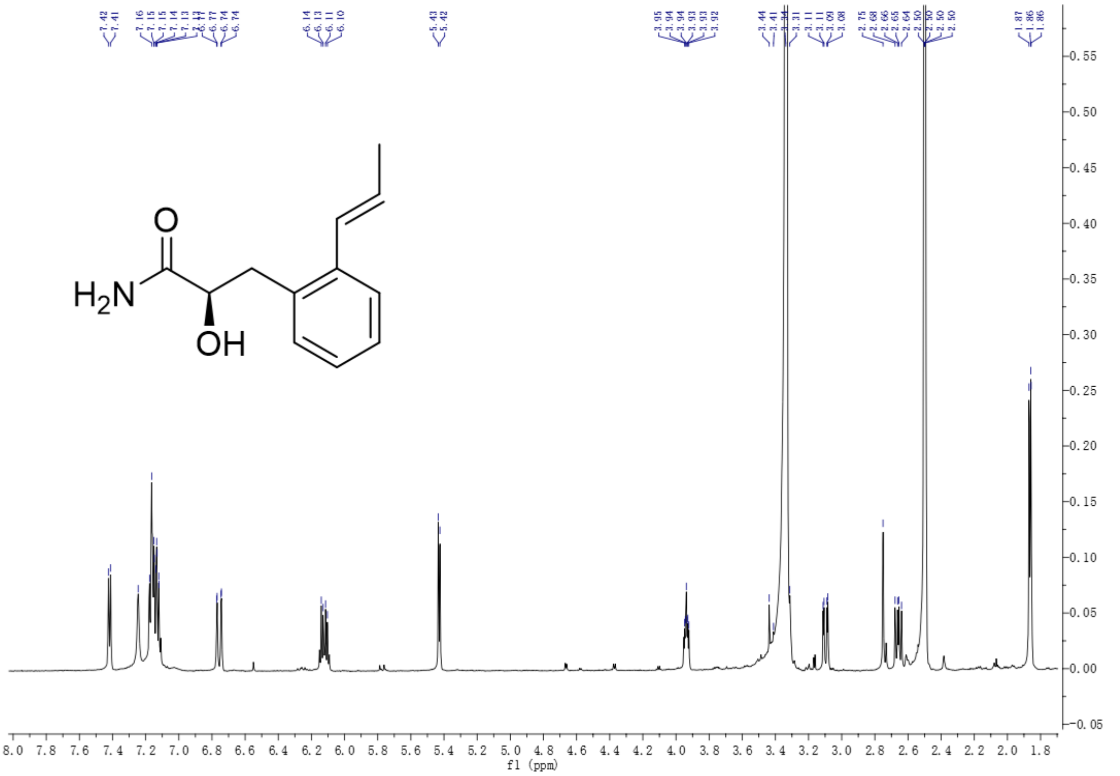

Figure S171. ^{1}H NMR spectrum of streptobenzenepropanamide A (**23**)

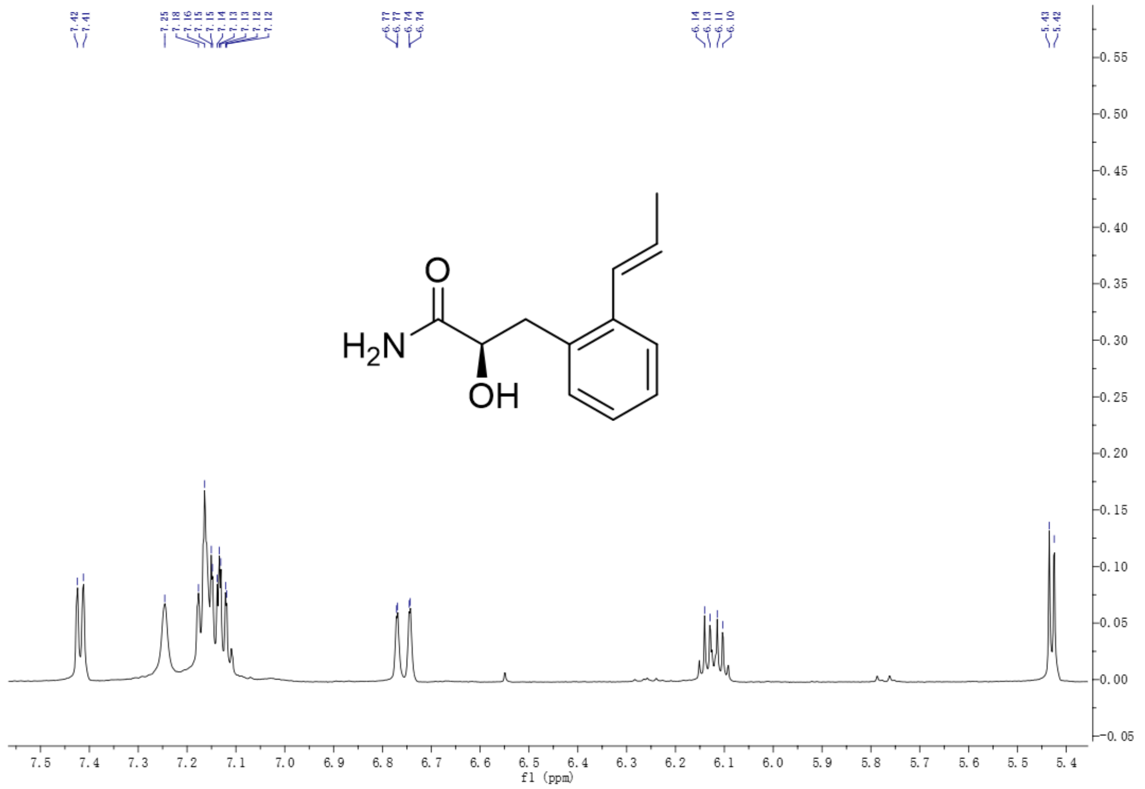

Figure S172. ^{1}H NMR spectrum of streptobenzenepropanamide A (**23**)

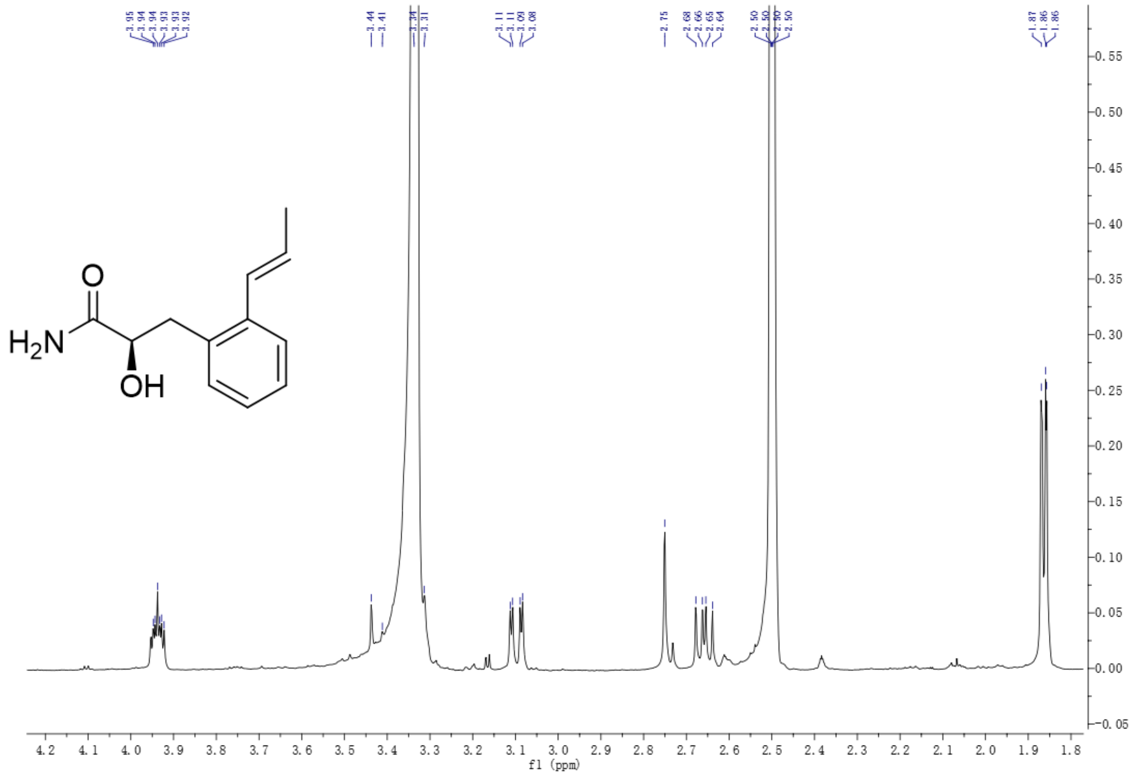

Figure S173. ^{13}C NMR spectrum of streptobenzenepropanamide A (**23**)

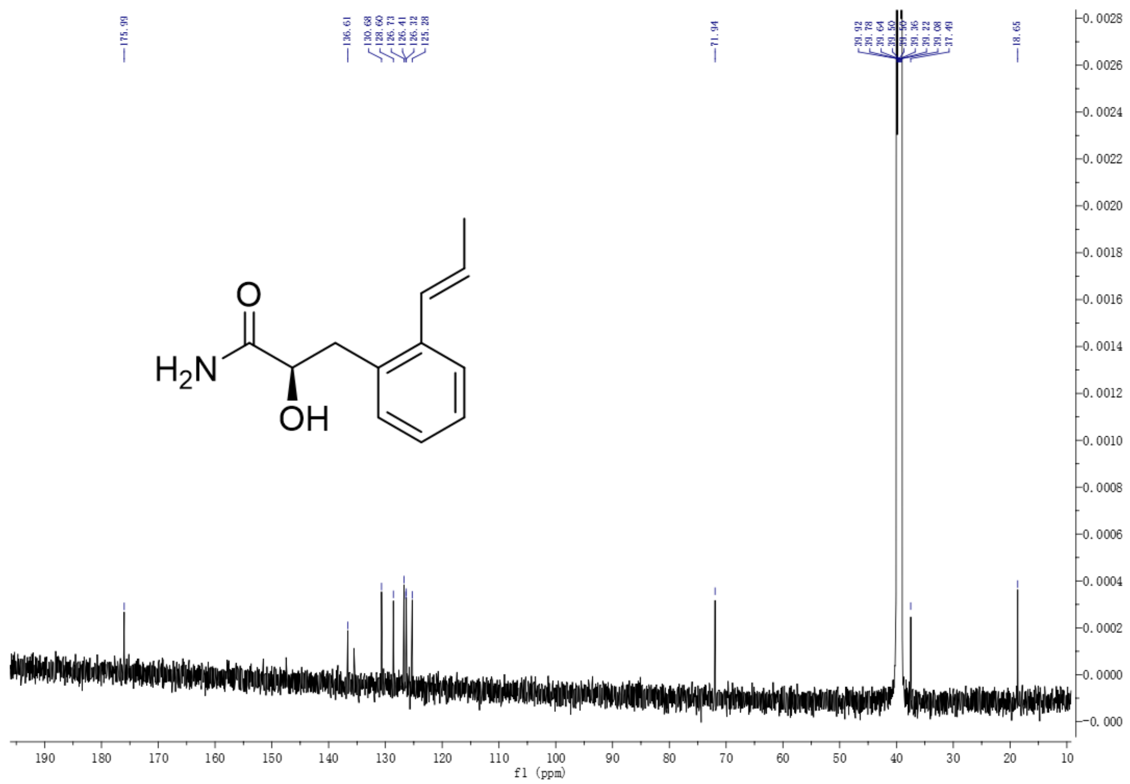

Figure S174. ^{13}C NMR spectrum of streptobenzenepropanamide A (**23**)

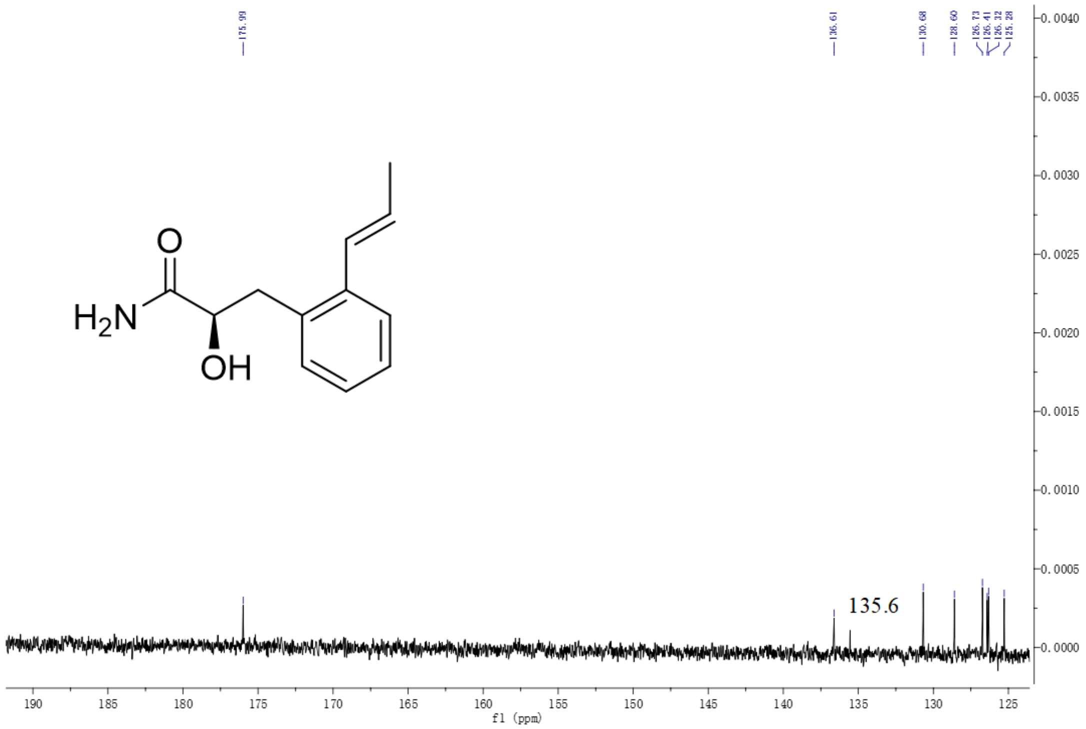

Figure S175. HMQC spectrum of streptobenzenepropanamide A (**23**)

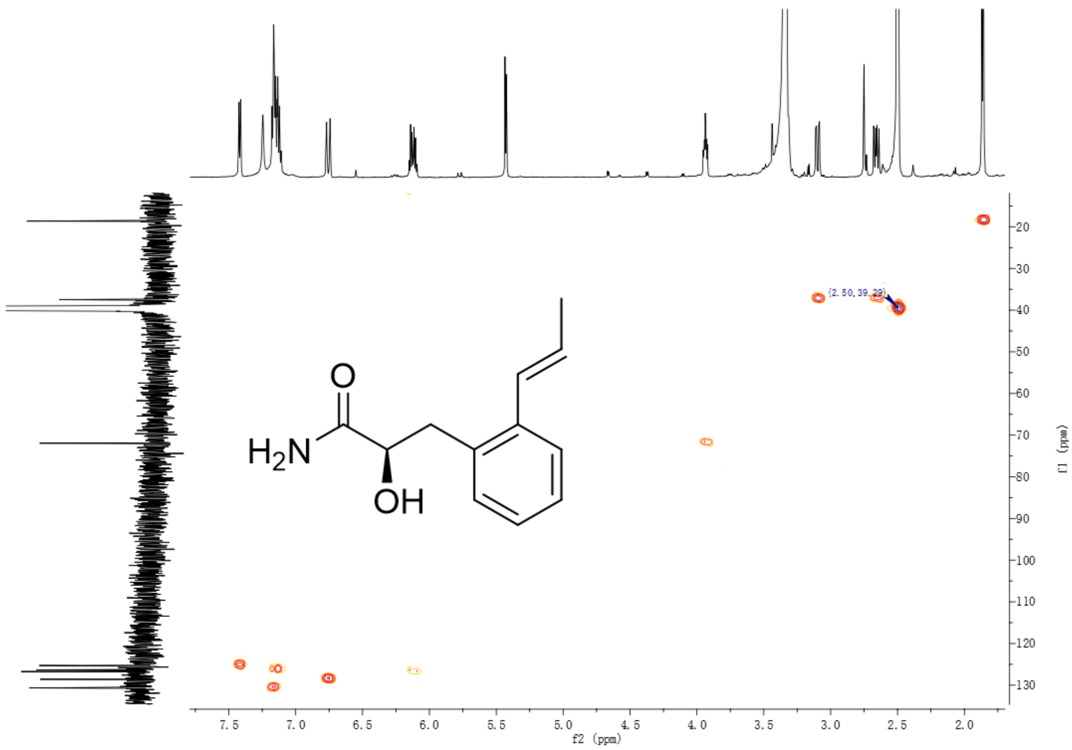

Figure S176. HMQC spectrum of streptobenzenepropanamide A (**23**)

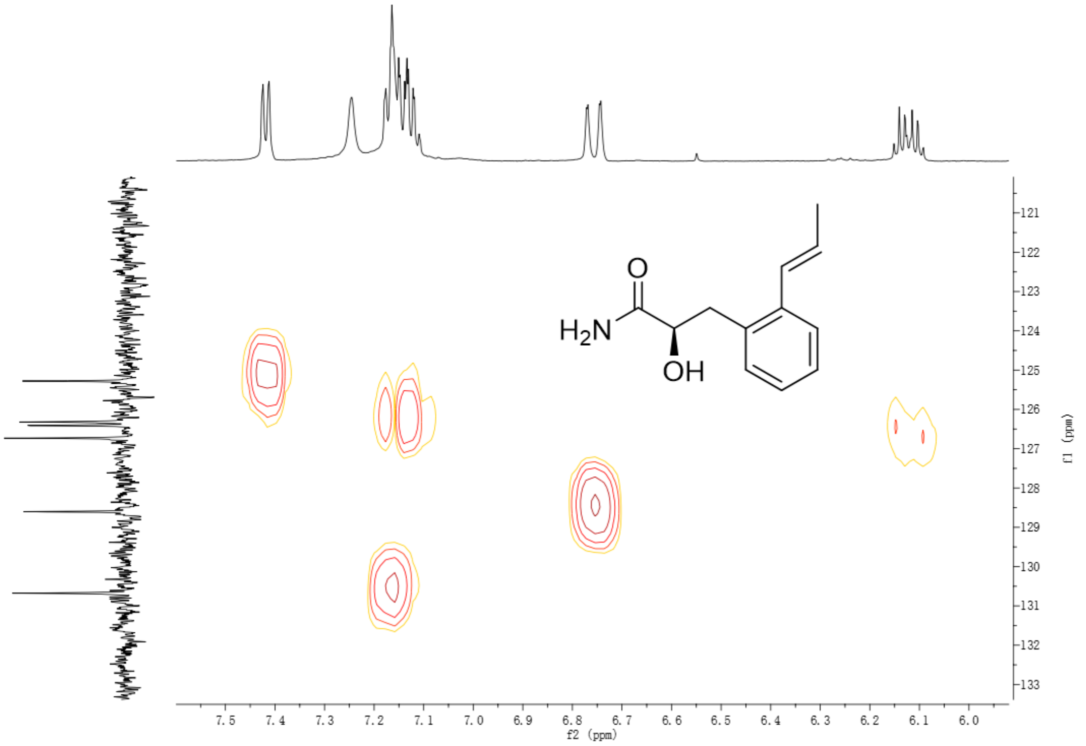

Figure S177. COSY spectrum of streptobenzenepropanamide A (**23**)

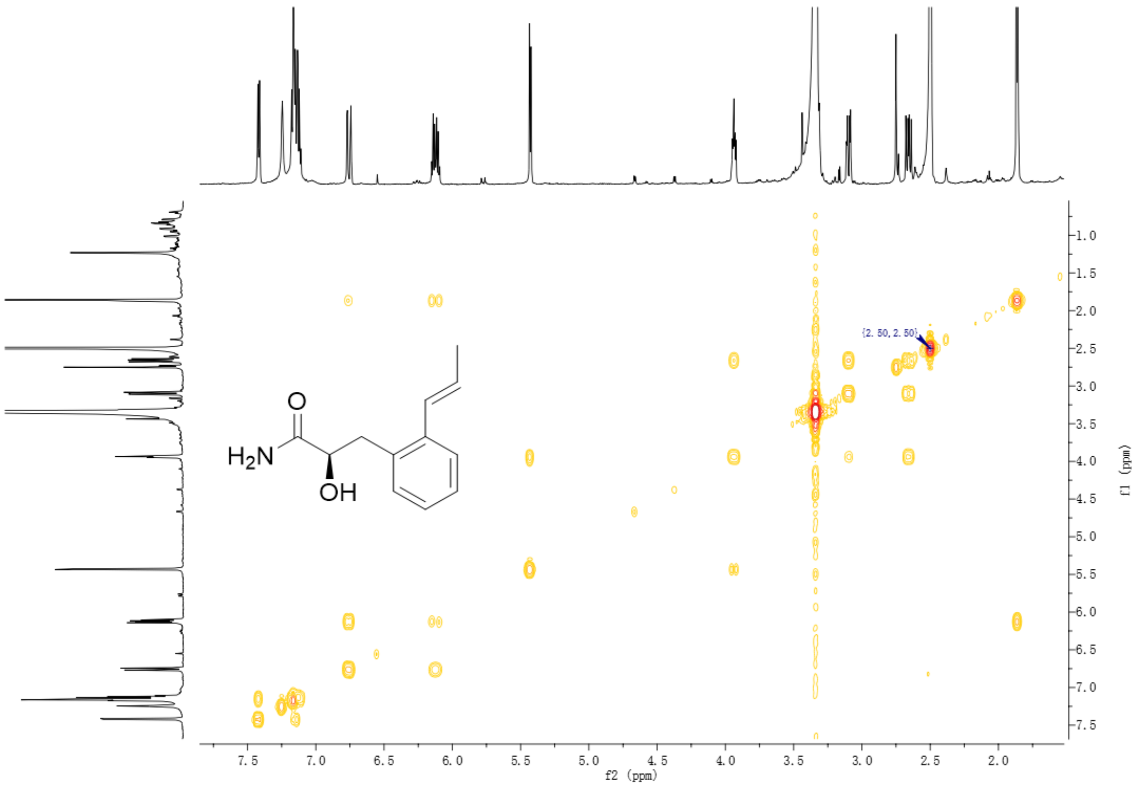

Figure S178. HMBC spectrum of streptobenzenepropanamide A (**23**)

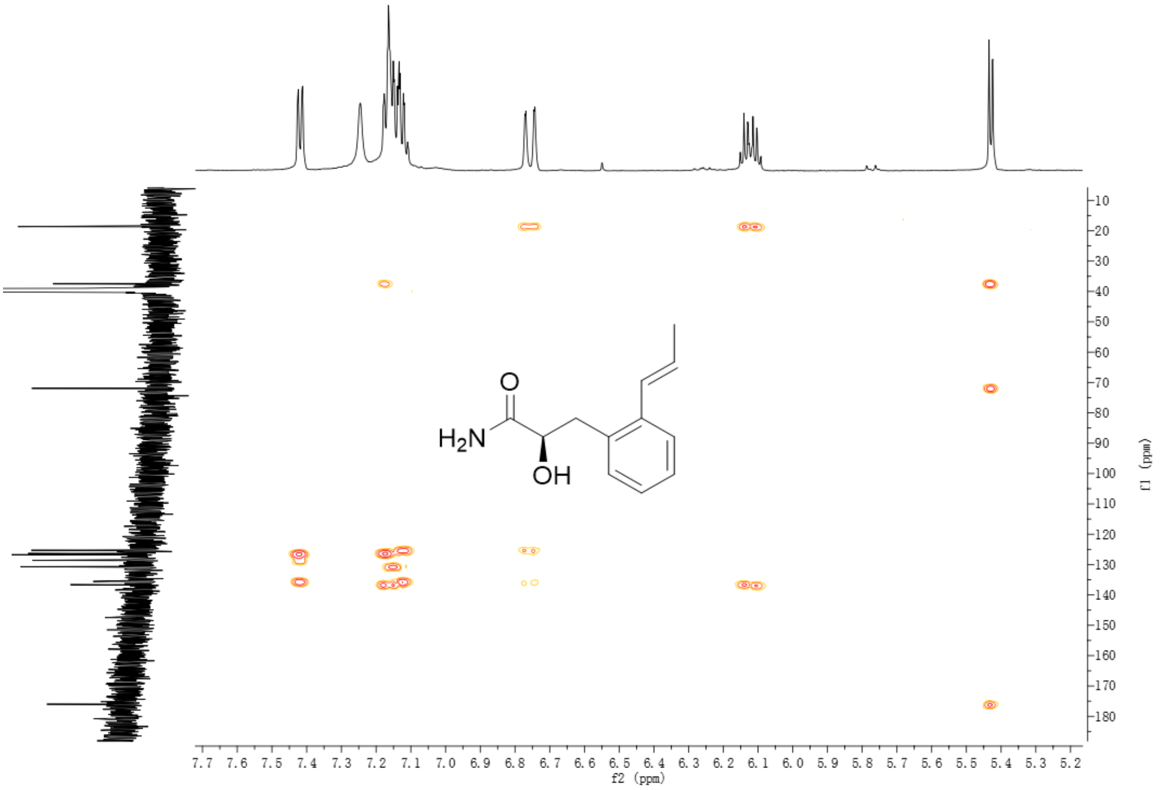

Figure S179. HMBC spectrum of streptobenzenepropanamide A (**23**)

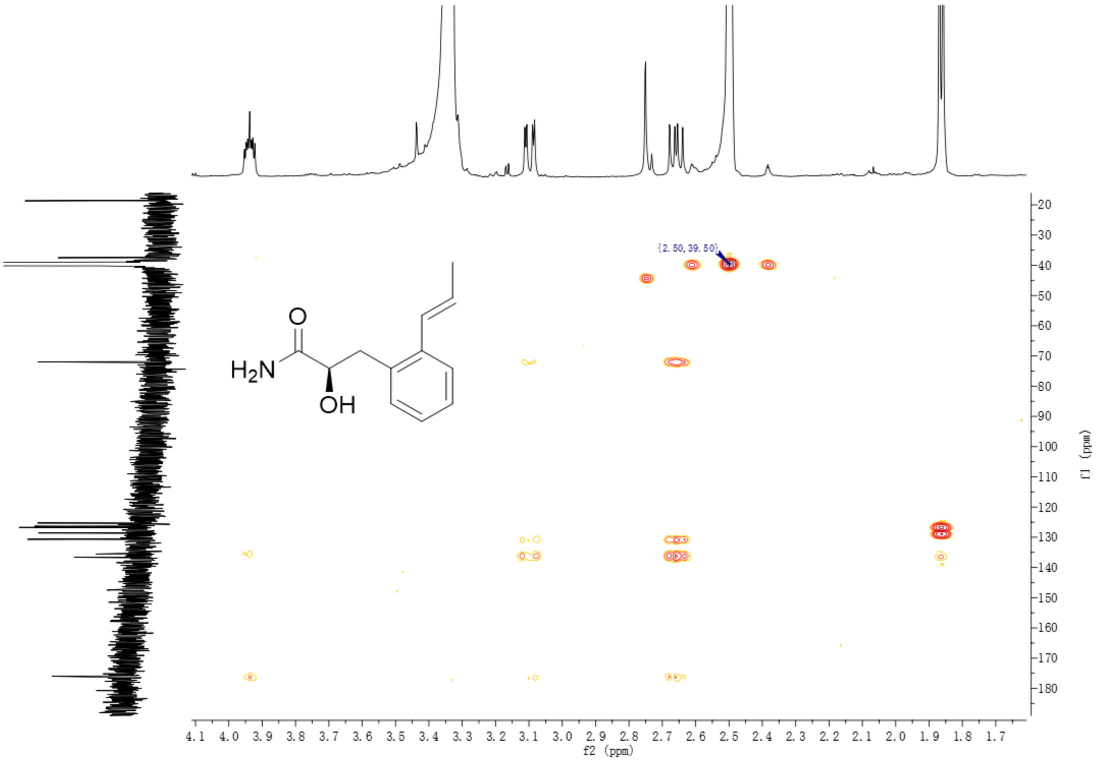

Figure S180. HRESIMS spectrum of streptobenzenepropanamide A (**23**)

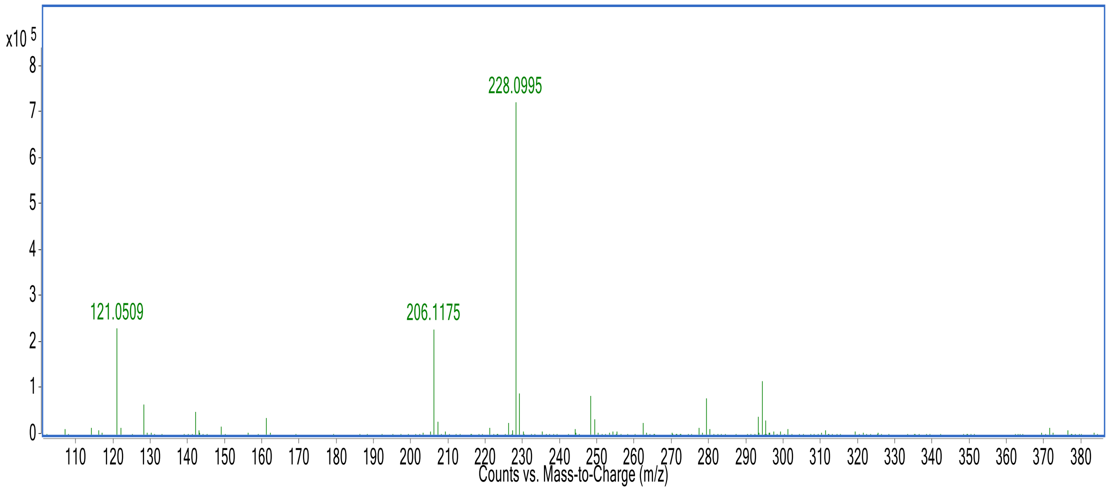

Figure S181. UV spectrum of streptobenzenepropanamide A (**23**)

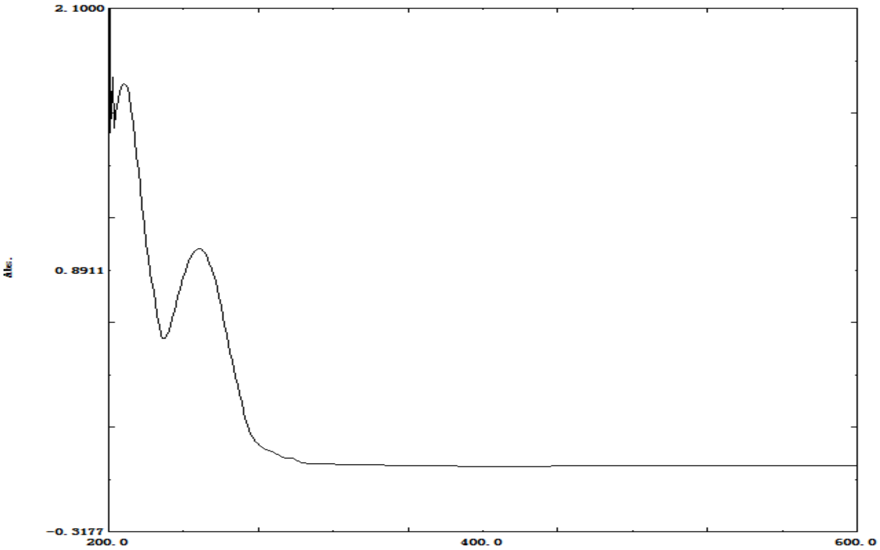

Figure S182. IR spectrum of streptobenzenepropanamide A (**23**)

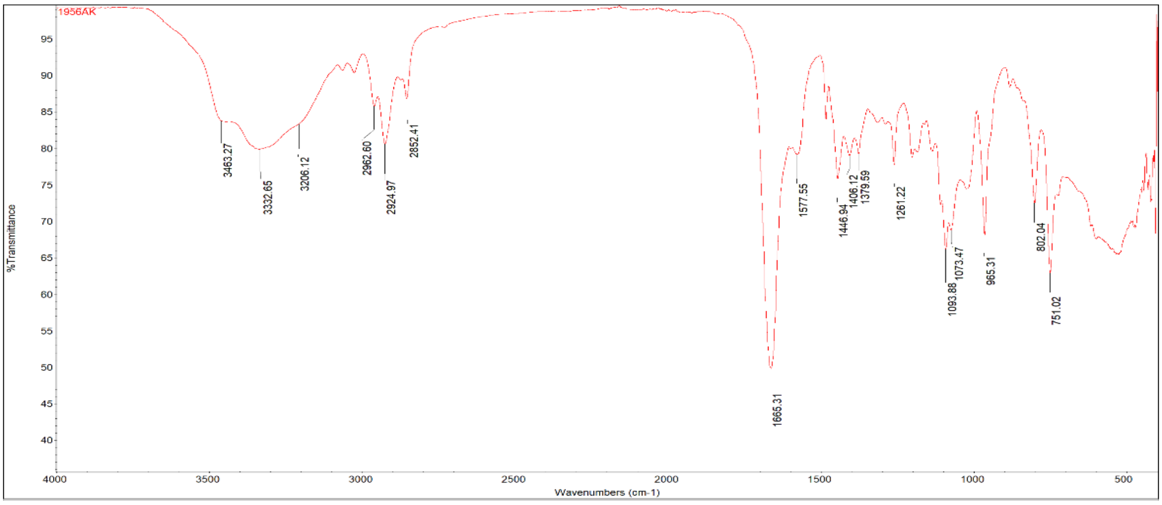

Table S_{16}. Gibbs free energies[a] and equilibrium populations[b] of low-energy conformers of *R*-**23**

Conformers	In MeOH	
	G	*P* (%)
R-**23a**	-671.5804428	6.39%
R-**23b**	-671.5795918	2.59%
R-**23c**	-671.5812962	15.79%
R-**23d**	-671.5811699	13.81%
R-**23e**	-671.5825771	61.41%

[a] B3LYP/6-31+G (d, p), in kcal/mol; [b] from G values at 298.15K.

Table S_{17}. Cartesian coordinates for the low-energy reoptimized MMFF conformers of *R*-**23** at B3LYP/6-311+G (d, p) level of theory in MeOH

R-**23a**			Standard Orientation (Ångstroms)		
No.	Atom	Type	X	Y	Z
1	6	0	-0.853748	2.529083	-1.753232
2	6	0	-1.040582	2.125552	-0.432146
3	6	0	-0.189754	1.182883	0.178676
4	6	0	0.886634	0.646475	-0.573921
5	6	0	1.067772	1.076781	-1.898355
6	6	0	0.210105	2.003886	-2.492371
7	6	0	1.836975	-0.397263	-0.011901
8	6	0	1.365438	-1.866862	-0.131892
9	6	0	1.238977	-2.300149	-1.603338
10	8	0	0.171111	-2.162813	0.594842
11	7	0	0.024178	-2.735604	-1.983740
12	8	0	2.221427	-2.250308	-2.355706
13	6	0	-0.422838	0.785584	1.587119
14	6	0	-1.629740	0.621342	2.159816
15	6	0	-1.853804	0.270912	3.600819
16	1	0	-1.521882	3.262523	-2.195233
17	1	0	-1.843859	2.563237	0.152697
18	1	0	1.896153	0.670026	-2.471506
19	1	0	0.377879	2.320178	-3.517680
20	1	0	2.790733	-0.335955	-0.542648
21	1	0	2.054658	-0.211729	1.044114
22	1	0	2.160379	-2.496741	0.286320
23	1	0	-0.262460	-1.344958	0.888674
24	1	0	-0.120361	-3.074013	-2.924529
25	1	0	-0.717883	-2.801405	-1.301040
26	1	0	0.459802	0.628310	2.207100
27	1	0	-2.525470	0.743125	1.550087
28	1	0	-2.437339	-0.653409	3.694230
29	1	0	-0.909850	0.142096	4.138928
30	1	0	-2.432653	1.056215	4.103816

	R-**23b**		Standard Orientation (Ångstroms)		
No.	Atom	Type	X	Y	Z
1	6	0	-0.909671	2.728589	-1.196484
2	6	0	-0.992300	2.167214	0.075062
3	6	0	-0.245256	1.024310	0.433487
4	6	0	0.589847	0.429005	-0.548893
5	6	0	0.660916	1.011306	-1.826620
6	6	0	-0.074304	2.149086	-2.157257
7	6	0	1.431096	-0.804808	-0.264832
8	6	0	0.796441	-2.131273	-0.745960
9	6	0	0.915922	-2.320446	-2.272547
10	8	0	-0.540965	-2.316992	-0.281401
11	7	0	-0.203186	-2.728153	-2.900988
12	8	0	1.996482	-2.137991	-2.847836
13	6	0	-0.340641	0.480976	1.807744
14	6	0	-0.586863	1.201505	2.915767
15	6	0	-0.706037	0.629507	4.297954
16	1	0	-1.500460	3.607077	-1.439520
17	1	0	-1.661802	2.607938	0.807146
18	1	0	1.318175	0.565288	-2.567892
19	1	0	0.002373	2.577011	-3.152431
20	1	0	2.398431	-0.715169	-0.766895
21	1	0	1.634351	-0.898261	0.803479
22	1	0	1.373135	-2.956690	-0.312980
23	1	0	-1.019123	-1.476664	-0.362693
24	1	0	-0.176956	-2.934540	-3.889702
25	1	0	-1.041144	-2.921410	-2.371155
26	1	0	-0.193049	-0.590400	1.925989
27	1	0	-0.703548	2.282460	2.836096
28	1	0	-1.692566	0.847449	4.727339
29	1	0	-0.562657	-0.455442	4.301616
30	1	0	0.032359	1.078519	4.974406

	R-**23c**		Standard Orientation (Ångstroms)		
No.	Atom	Type	X	Y	Z
1	6	0	-0.971924	2.364810	-2.023652
2	6	0	-1.174026	2.046663	-0.681901
3	6	0	-0.928832	0.751513	-0.181728
4	6	0	-0.478544	-0.248196	-1.084897
5	6	0	-0.293159	0.088163	-2.435271
6	6	0	-0.531450	1.378418	-2.911093
7	6	0	-0.187242	-1.667321	-0.636996
8	6	0	1.083925	-1.864512	0.236168
9	6	0	2.384738	-1.691100	-0.566389
10	8	0	1.082728	-3.173436	0.786436

11	7	0	2.769072	-0.438945	-0.867265
12	8	0	3.027842	-2.698103	-0.898849
13	6	0	-1.168049	0.442005	1.243613
14	6	0	-1.095670	1.312748	2.265623
15	6	0	-1.380635	0.972143	3.698884
16	1	0	-1.175416	3.371443	-2.377330
17	1	0	-1.555280	2.807961	-0.008306
18	1	0	0.047545	-0.680216	-3.124553
19	1	0	-0.383006	1.607492	-3.962180
20	1	0	-1.013579	-2.061150	-0.035562
21	1	0	-0.102828	-2.319683	-1.512526
22	1	0	1.072962	-1.145600	1.063038
23	1	0	1.766302	-3.664948	0.291723
24	1	0	3.586429	-0.293616	-1.444728
25	1	0	2.207372	0.364276	-0.624495
26	1	0	-1.425138	-0.589895	1.478968
27	1	0	-0.802052	2.344225	2.070449
28	1	0	-2.202241	1.585822	4.090645
29	1	0	-1.652491	-0.081146	3.818830
30	1	0	-0.509352	1.179187	4.333343

	***R*-23d**		Standard Orientation (Ångstroms)		
No.	Atom	Type	X	Y	Z
1	6	0	-1.240479	2.015607	-2.695325
2	6	0	-1.004917	2.195565	-1.332855
3	6	0	-0.236439	1.280643	-0.585994
4	6	0	0.321860	0.161131	-1.256967
5	6	0	0.088036	0.004706	-2.631184
6	6	0	-0.688301	0.913511	-3.353476
7	6	0	1.149014	-0.883869	-0.533879
8	6	0	0.324016	-1.830528	0.362323
9	6	0	1.252393	-2.746739	1.181488
10	8	0	-0.662635	-2.569448	-0.360138
11	7	0	0.977969	-4.063645	1.120085
12	8	0	2.171261	-2.267350	1.858753
13	6	0	-0.000481	1.503324	0.855940
14	6	0	-0.822270	2.151283	1.700206
15	6	0	-0.536156	2.393354	3.153226
16	1	0	-1.834559	2.742113	-3.242238
17	1	0	-1.402718	3.076203	-0.837800
18	1	0	0.527563	-0.846228	-3.145939
19	1	0	-0.849681	0.767154	-4.417385
20	1	0	1.688554	-1.490522	-1.272097
21	1	0	1.912724	-0.423596	0.099216
22	1	0	-0.232618	-1.238668	1.097456

23	1	0	-0.339338	-2.757587	-1.253222
24	1	0	1.519196	-4.716560	1.669162
25	1	0	0.178967	-4.389653	0.595018
26	1	0	0.925355	1.100443	1.264285
27	1	0	-1.776018	2.530414	1.332740
28	1	0	-1.317359	1.954419	3.786953
29	1	0	0.428308	1.971191	3.452041
30	1	0	-0.524246	3.468335	3.375607

	R-**23e**		Standard Orientation (Ångstroms)		
No.	Atom	Type	X	Y	Z
1	6	0	-1.238054	1.919406	-2.688575
2	6	0	-0.996845	2.146553	-1.334019
3	6	0	-0.218745	1.262864	-0.560354
4	6	0	0.350713	0.128828	-1.199309
5	6	0	0.105930	-0.078246	-2.566372
6	6	0	-0.683347	0.799656	-3.313211
7	6	0	1.182268	-0.893153	-0.448021
8	6	0	0.324547	-1.856000	0.407839
9	6	0	1.230203	-2.824364	1.172890
10	8	0	-0.593758	-2.611107	-0.383635
11	7	0	1.136726	-4.122609	0.829096
12	8	0	2.001994	-2.395857	2.040866
13	6	0	0.013695	1.530639	0.874186
14	6	0	-0.816424	2.196134	1.696480
15	6	0	-0.537301	2.482384	3.142699
16	1	0	-1.840684	2.623068	-3.255569
17	1	0	-1.399982	3.039667	-0.866629
18	1	0	0.550080	-0.942710	-3.053403
19	1	0	-0.851337	0.616000	-4.370195
20	1	0	1.752301	-1.492117	-1.166325
21	1	0	1.906052	-0.414239	0.217391
22	1	0	-0.220896	-1.279282	1.166032
23	1	0	-1.080478	-2.004351	-0.960700
24	1	0	1.706872	-4.809612	1.302006
25	1	0	0.471054	-4.417167	0.128631
26	1	0	0.940806	1.146814	1.296973
27	1	0	-1.772325	2.555871	1.315469
28	1	0	-1.317130	2.055181	3.786038
29	1	0	0.429599	2.077349	3.456883
30	1	0	-0.535533	3.563400	3.333839

Table S19. Gibbs free energies[a] and equilibrium populations[b] of low-energy conformers of *S*-**23**

Conformers	In MeOH	
	G	*P* (%)
S-**23a**	-671.5804428	5.13%
S-**23b**	-671.5812962	12.67%
S-**23c**	-671.5811699	11.08%
S-**23d**	-671.5825771	49.25%
S-**23e**	-671.5818118	21.88%

[a] B3LYP/6-31+G (d, p), in kcal/mol; [b] from G values at 298.15K.

Table S19. Cartesian coordinates for the low-energy reoptimized MMFF conformers of *S*-**23** at B3LYP/6-311+G (d, p) level of theory in MeOH

S-**23a**			Standard Orientation (Ångstroms)		
No.	Atom	Type	X	Y	Z
1	6	0	0.053011	2.170207	-2.342176
2	6	0	-0.480434	2.107539	-1.055904
3	6	0	-0.725616	0.875156	-0.418096
4	6	0	-0.429833	-0.324704	-1.114927
5	6	0	0.092630	-0.241324	-2.415770
6	6	0	0.339361	0.987442	-3.029954
7	6	0	-0.630739	-1.698825	-0.498594
8	6	0	0.540524	-2.222545	0.367654
9	6	0	1.815433	-2.424835	-0.470546
10	8	0	0.809814	-1.436463	1.530288
11	7	0	2.898203	-1.736286	-0.066006
12	8	0	1.809134	-3.193633	-1.441440
13	6	0	-1.297599	0.855243	0.948752
14	6	0	-1.000146	1.727613	1.929612
15	6	0	-1.625411	1.729894	3.292900
16	1	0	0.225057	3.134813	-2.810831
17	1	0	-0.738445	3.026352	-0.538144
18	1	0	0.312694	-1.160886	-2.950690
19	1	0	0.740733	1.019898	-4.038564
20	1	0	-1.531874	-1.730745	0.121014
21	1	0	-0.774706	-2.431777	-1.296815
22	1	0	0.262482	-3.225271	0.715239
23	1	0	0.340974	-0.587122	1.485501
24	1	0	3.780088	-1.853060	-0.544691
25	1	0	2.845237	-1.170314	0.769422
26	1	0	-2.027883	0.075696	1.165877
27	1	0	-0.249286	2.496200	1.744140
28	1	0	-2.347909	0.916522	3.409648
29	1	0	-0.862394	1.633472	4.075271
30	1	0	-2.143100	2.679743	3.478830

	S-**23b**		Standard Orientation (Ångstroms)		
No.	Atom	Type	X	Y	Z
1	6	0	-1.027605	1.860971	-2.472547
2	6	0	-0.648530	2.072130	-1.148132
3	6	0	0.110232	1.122756	-0.433518
4	6	0	0.503596	-0.068602	-1.099720
5	6	0	0.123113	-0.257498	-2.437840
6	6	0	-0.636786	0.688938	-3.126867
7	6	0	1.318400	-1.146122	-0.410928
8	6	0	0.577730	-1.980241	0.672042
9	6	0	-0.461556	-2.941004	0.069465
10	8	0	1.518924	-2.756448	1.398496
11	7	0	-1.628009	-2.417340	-0.344413
12	8	0	-0.193344	-4.149423	-0.007692
13	6	0	0.509709	1.383633	0.965402
14	6	0	-0.155787	2.147772	1.849225
15	6	0	0.297961	2.420986	3.253119
16	1	0	-1.608043	2.615391	-2.995758
17	1	0	-0.921391	3.003078	-0.660747
18	1	0	0.429258	-1.168371	-2.945957
19	1	0	-0.912427	0.516629	-4.163026
20	1	0	1.719254	-1.838862	-1.158378
21	1	0	2.182191	-0.710132	0.102332
22	1	0	0.077566	-1.303655	1.373993
23	1	0	1.388932	-3.675393	1.094783
24	1	0	-2.307611	-3.011857	-0.799768
25	1	0	-1.808059	-1.424280	-0.312482
26	1	0	1.429197	0.907774	1.303639
27	1	0	-1.100194	2.606482	1.556086
28	1	0	1.242179	1.916045	3.479389
29	1	0	0.436278	3.497656	3.416721
30	1	0	-0.454177	2.090989	3.981081

	S-**23c**		Standard Orientation (Ångstroms)		
No.	Atom	Type	X	Y	Z
1	6	0	-0.215979	2.548439	-2.514393
2	6	0	-0.486450	2.459116	-1.149448
3	6	0	-0.564304	1.217039	-0.488646
4	6	0	-0.379176	0.033547	-1.250413
5	6	0	-0.124081	0.141781	-2.625493
6	6	0	-0.035689	1.381704	-3.261975
7	6	0	-0.429631	-1.346335	-0.623965
8	6	0	0.809176	-1.697991	0.225470
9	6	0	0.603211	-3.039000	0.954655
10	8	0	2.027440	-1.671174	-0.520639

11	7	0	1.591435	-3.942097	0.809353
12	8	0	-0.409838	-3.233317	1.639341
13	6	0	-0.858310	1.156399	0.958498
14	6	0	-0.551291	2.098723	1.867219
15	6	0	-0.897130	2.023889	3.325477
16	1	0	-0.166389	3.521999	-2.993539
17	1	0	-0.666521	3.368345	-0.584011
18	1	0	0.004185	-0.766238	-3.209701
19	1	0	0.160576	1.433589	-4.328820
20	1	0	-1.306224	-1.463197	0.019350
21	1	0	-0.529554	-2.098533	-1.416614
22	1	0	0.931616	-0.947400	1.014264
23	1	0	1.856811	-1.948042	-1.432654
24	1	0	1.542169	-4.824972	1.298021
25	1	0	2.420797	-3.703761	0.284239
26	1	0	-1.368610	0.261978	1.313206
27	1	0	-0.003622	2.987150	1.552347
28	1	0	-1.429688	1.099252	3.568636
29	1	0	0.005452	2.077277	3.947607
30	1	0	-1.528380	2.871830	3.621625

	***S*-23d**		Standard Orientation (Ångstroms)		
No.	Atom	Type	X	Y	Z
1	6	0	-0.059622	2.410569	-2.575152
2	6	0	-0.315367	2.414792	-1.204321
3	6	0	-0.489315	1.220099	-0.478131
4	6	0	-0.426405	-0.012643	-1.181671
5	6	0	-0.178531	0.002753	-2.563689
6	6	0	0.009308	1.196959	-3.263709
7	6	0	-0.576248	-1.352100	-0.486140
8	6	0	0.695700	-1.774778	0.287641
9	6	0	0.455525	-3.107159	1.002019
10	8	0	1.830593	-1.908830	-0.569067
11	7	0	1.185651	-4.152699	0.570572
12	8	0	-0.382614	-3.180733	1.910194
13	6	0	-0.754421	1.259722	0.975067
14	6	0	-0.344205	2.217250	1.825369
15	6	0	-0.658192	2.248047	3.292290
16	1	0	0.066810	3.350753	-3.104166
17	1	0	-0.405550	3.363991	-0.685172
18	1	0	-0.139180	-0.942143	-3.099778
19	1	0	0.194836	1.177746	-4.333464
20	1	0	-1.409004	-1.348003	0.222729
21	1	0	-0.792684	-2.123624	-1.232840
22	1	0	0.904581	-1.036826	1.072983

23	1	0	1.907583	-1.110525	-1.112047
24	1	0	1.073761	-5.057260	1.006387
25	1	0	1.872336	-4.027416	-0.159706
26	1	0	-1.330302	0.432753	1.387487
27	1	0	0.270785	3.036437	1.452478
28	1	0	-1.262674	1.387680	3.595659
29	1	0	0.261285	2.253660	3.891497
30	1	0	-1.205441	3.162530	3.555680

	S-**23e**		Standard Orientation (Ångstroms)		
No.	Atom	Type	X	Y	Z
1	6	0	-0.399995	2.728075	-2.304726
2	6	0	-0.571271	2.538317	-0.934399
3	6	0	-0.596622	1.250473	-0.361748
4	6	0	-0.463891	0.126558	-1.218615
5	6	0	-0.303177	0.336244	-2.596208
6	6	0	-0.266166	1.619272	-3.145479
7	6	0	-0.472078	-1.295235	-0.692573
8	6	0	0.841584	-1.715040	-0.000051
9	6	0	0.649871	-3.073915	0.695241
10	8	0	1.881922	-1.754562	-0.980122
11	7	0	1.320455	-4.108425	0.154134
12	8	0	-0.095039	-3.173743	1.679297
13	6	0	-0.780881	1.084525	1.095425
14	6	0	-0.413294	1.963964	2.043976
15	6	0	-0.648216	1.781099	3.514896
16	1	0	-0.389494	3.733987	-2.714667
17	1	0	-0.713676	3.402717	-0.292961
18	1	0	-0.202203	-0.527348	-3.248217
19	1	0	-0.143526	1.750119	-4.216671
20	1	0	-1.286265	-1.449687	0.021654
21	1	0	-0.644993	-1.987134	-1.524257
22	1	0	1.085682	-0.994486	0.788140
23	1	0	2.721298	-1.512684	-0.565977
24	1	0	1.231270	-5.033260	0.550722
25	1	0	1.913500	-3.959742	-0.650327
26	1	0	-1.254812	0.160631	1.423387
27	1	0	0.102267	2.879695	1.754070
28	1	0	-1.153440	0.834338	3.729720
29	1	0	0.297826	1.802237	4.070916
30	1	0	-1.262637	2.597010	3.917421
